# Interpersonal violence injuries among children and adolescents in the European Union from 1990 to 2023, a Global Burden of Disease study

**DOI:** 10.1093/eurpub/ckag078

**Published:** 2026-06-16

**Authors:** GBD 2023 Italy Interpersonal Violence Collaborators, Giulia Zamagni, Luca Ronfani, Lorenzo Monasta, Angelo Capodici, Giulia Zamagni, Benedetta Armocida, Giulio Castelpietra, Barbara Corso, Giovanni Damiani, Davide Golinelli, Carlo La Vecchia, Luca Ronfani, Francesco Sanmarchi, Dario Tedesco, Eugenio Traini, Giovanni Trisolino, Francesco Violante, Lorenzo Monasta

**Affiliations:** Scuola Superiore Sant’Anna, Pisa, Italy; Institute for Maternal and Child Health—IRCCS “Burlo Garofolo”, Trieste, Italy; Istituto Superiore di Sanità (ISS), Rome, Italy; University of Trieste, Trieste, Italy; National Research Council (CNR), Pisa, Italy; University of Milan, Milan, Italy; Link Campus University, Rome, Italy; University of Milan, Milan, Italy; Institute for Maternal and Child Health—IRCCS “Burlo Garofolo”, Trieste, Italy; University of Bologna, Bologna, Italy; University of Parma, Parma, Italy; TNO, Utrecht, The Netherlands; Rizzoli Orthopaedic Institute (IOR), Bologna, Italy; University of Bologna, Bologna, Italy; Institute for Maternal and Child Health—IRCCS “Burlo Garofolo”, Trieste, Italy

## Abstract

Interpersonal violence against children and adolescents constitutes a significant public health issue associated with substantial morbidity and long-term disability. This study utilizes Global Burden of Disease (GBD) 2023 estimates to quantify the burden of interpersonal violence injuries among the population <20 years of age across the 27 European Union countries (EU27). We analysed incidence and Years Lived with Disability (YLDs) for injury types and age groups (<5, 5–14, and 15–19 years). A ‘Disability Drivers’ analysis compared injury frequency against disability burden to identify priority areas. Long-term (1990–2023), mid-term (2010–2023), and short-term trends (2020–2023) were analysed to distinguish systemic risks from emerging threats. The analysis reveals geographical and temporal heterogeneity. In 2023, Hungary reported the highest incidence rate (1085.7 per 100 000; 95% UI: 917.6–1295.5), while Italy reported the lowest (131.9 per 100 000; 95% UI: 94.3–175.5). Despite low overall incidence, Italy exhibited short-term spikes (2020–2023) in severe trauma among children aged <5, with severe chest injuries rising by 79.1% (95% UI: 66.4%–92.3%). Long-term analysis identified systemic worsening in Eastern Europe, notably in Romania, where poisoning in children <5 years increased by 77.9% (95% UI: 55.5%–96.0%) since 1990. Violence-related injuries among children in the EU27 show a polarized pattern, with chronic burdens in Eastern Europe and emerging acute escalations in Southern Europe. Prevention strategies should consider both injury frequency and disability burden to effectively allocate resources.

## Introduction

Interpersonal violence remains a major determinant of health outcomes for children and adolescents globally, contributing to premature mortality, non-fatal injuries, and long-term disability [1]. Within the European Union (EU27), the epidemiology of paediatric trauma resulting from violence exhibits complex variations driven by socioeconomic factors [2], reporting mechanisms, and regional stability. While aggregate trends may suggest a stabilization of violence indicators in high-income nations [3], granular analyses reveal persistent and emerging risks that disproportionately affect specific age groups and anatomical regions. Understanding the full scope of this burden requires shifting focus from mortality statistics to a comprehensive assessment of morbidity, specifically analysing the interaction between injury frequency and the resulting Years Lived with Disability (YLDs).

Despite the recognized importance of violence-related injuries, no prior study has provided a comprehensive, age-stratified analysis of interpersonal violence injuries across the EU27 using the most recent estimates from the Global Burden of Disease Study (GBD). Previous work has focused on mortality or has examined violence-related morbidity at the global level without differentiating age-specific vulnerability patterns within the European context [1, 3]. Therefore, this gap limits the ability of policymakers to identify at-risk subpopulations and to allocate prevention resources effectively.

This study aims at filling this gap by using GBD 2023 estimates to provide a detailed epidemiological profile of interpersonal violence injuries among individuals under 20 years of age in the EU27. We stratify estimates into three developmental stages, early childhood (<5 years) [4], childhood (5–14 years) [5], and adolescence (15–19 years) [6], to capture age-specific vulnerability patterns, and aligning with authoritative literature.

This paper encompasses a comparative burden analysis by juxtaposing injury incidence against YLD rankings to identify ‘Hidden Burdens’: low-frequency injuries that exert a disproportionate impact on quality of life. Furthermore, it examines temporal trends across three horizons (long-term, mid-term, and short-term) to contextualize recent changes against historical baselines. By integrating anatomical categorization with disability metrics and temporal trending, this paper aims to provide an evidence-based framework for prioritizing public health interventions across the European Union, supporting the objectives of the EU Strategy on the Rights of the Child and the European Child Guarantee [7].

## Methods

### Study overview

This study presents GBD 2023 estimates for interpersonal violence injuries among children and adolescents across the 27 countries of the EU27. The population was stratified into three age groups: <5, 5–14, and 15–19 years, in addition to an overall aggregate analysis for the under-20 population. This stratification was chosen to reflect distinct epidemiological and developmental stages. The <5 group aligns with the standard survival surveillance metrics defined by the UN Inter-agency Group for Child Mortality Estimation [4]. The 5–14 years group was isolated to encompass the ‘neglected’ group in older children and early adolescents, as characterized by Masquelier *et al*. [5], and finally, the 15–19 years group corresponds to the ‘late adolescence’ phase, identified by the second Lancet Commission on Adolescent Health and Wellbeing [6]. Interpersonal violence was defined according to the GBD cause hierarchy (Cause ID 724); its specific ICD codes can be found at https://ghdx.healthdata.org/record/ihme-data/gbd-2023-cause-icd-code-mappings.

GBD 2023 estimates for interpersonal violence are publicly available in the GBD Results Tool (https://vizhub.healthdata.org/gbd-results/) and presented in the capstone papers [8, 9].

### Anatomical and clinical grouping

To facilitate clinical interpretation, specific injury types (GBD Level three causes) were aggregated into seven distinct anatomical and clinical districts (Level two categories) based on the International Classification of Diseases (ICD) codes inherent to the GBD hierarchy, and as already inherent to GBD estimates. Specific groupings can be found in the appendix of Saleib *et al*. publication [10]. The groupings were defined as follows:

1. Head Injuries: minor, moderate, and severe traumatic brain injuries (TBI).
2. Fractures: fractures of the face, skull, spine, pelvis, ribs/sternum, clavicle/scapula/humerus, radius/ulna, femur, patella/tibia/fibula/ankle, foot, and hand.
3. Amputations: unilateral and bilateral amputations of upper and lower limbs, digits (fingers/thumbs), and toes.
4. Spinal Injuries: spinal cord lesions at and below the neck level.
5. Burns: thermal injuries based on total body surface area (<20% and ≥20%) and lower airway burns.
6. Minor Injuries: superficial injuries, open wounds, contusions, foreign bodies in the ear, and muscle/tendon injuries (sprains and strains).
7. Other Injuries: dislocations (shoulder, knee, hip), crushing injuries, nerve injuries, injury to eyes, asphyxiation, drowning/submersion, foreign bodies in respiratory/GI/urogenital systems, poisoning requiring urgent care, and internal haemorrhages.

### Temporal trends and policy analysis

Temporal trends were assessed using the percent change in incidence rates between the baseline year (1990) and the endpoint year (2023). Changes were considered significant if the 95% uncertainty interval (UI) did not cross the null.

To guide public health prioritization, we conducted a rank-discordance comparison by juxtaposing the rank order of injuries based on frequency (incidence) versus disability burden (YLDs), regardless of age and sex.

The rationale for this rank-discordance approach is the following: as YLDs are the product of prevalence and disability weights, an injury that ranks substantially higher in YLD contribution than in incidence indicates that its clinical severity and chronicity outweigh its frequency of occurrence. Specifically, for each of the injury types included in the GBD hierarchy, we computed separate rank orderings based on (i) total incidence counts and (ii) total YLDs across all age groups and both sexes in the EU27 for 2023. Injuries for which the YLD rank exceeded the incidence rank by five or more positions were flagged as ‘Hidden Burdens’: this threshold was chosen to highlight clinically meaningful discrepancies rather than minor rank fluctuations.

### Data sources and processing

Data sources used to produce GBD 2023 estimates are listed in the GBD 2023 Sources Tool (https://sources.healthdata.org/collection/sources-2023). Data on interpersonal violence were available for 15 of the 27 distinct countries considered. Specifically, a 2008 European administrative dataset covers Slovenia, Czechia, Belgium, Croatia, and Finland; notably, this constitutes the exclusive source for Belgium, Croatia, and Finland. The Netherlands exhibits the most extensive data coverage, comprising 17 administrative datasets, 15 registry records, one survey, and three scientific articles. Italy follows with 16 administrative sources. Spain utilizes eight administrative datasets and one survey, while Austria and Portugal rely on three and two administrative sources, respectively. The remaining countries, Bulgaria, Czechia, Denmark, Estonia, Hungary, Slovenia, and Sweden, are limited to a single data source.

For countries lacking primary data, GBD estimates rely more heavily on statistical modelling using covariates and data from neighbouring or epidemiologically similar countries, which may introduce additional uncertainty. This heterogeneity in data availability should be considered when interpreting cross-country comparisons, as countries with fewer data sources may show less precise estimates.

### GBD researching and reporting practices

GBD 2023 complies with the Guidelines for Accurate and Transparent Health Estimates Reporting (GATHER) guidelines [11]. The specific downstream analysis for this study was conducted using R version 4.2.

### Statistical analysis and uncertainty

Incident cases, incidence and YLD rates (per 100 000 population) were analysed for the period 1990–2023. All estimates were presented as the mean value accompanied by 95% UIs derived from the posterior distribution. Specifically, 95% UIs were defined as the 2.5th and 97.5th percentiles of 250 posterior draws.

## Results

**Figure 1.**
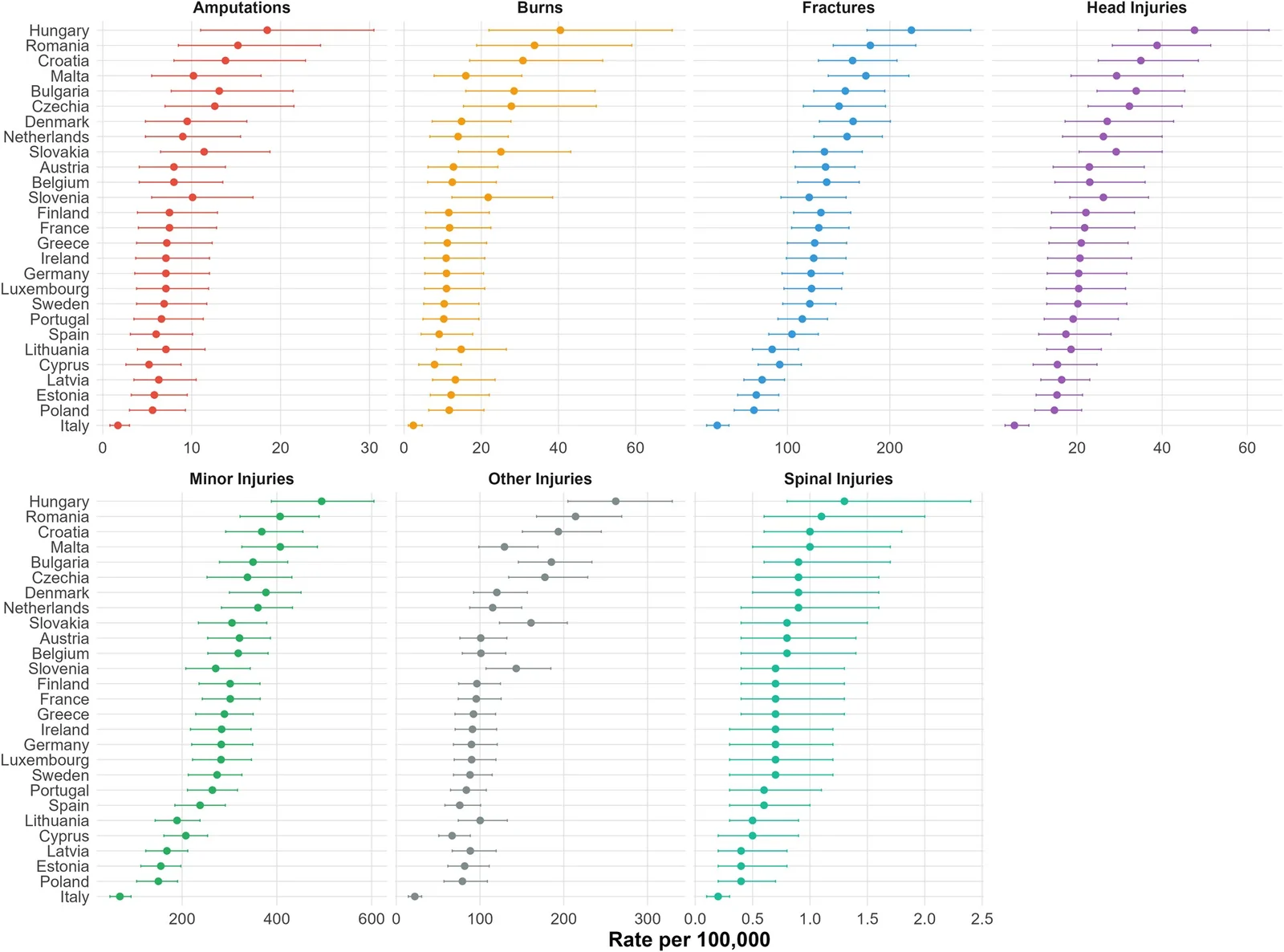
Forest plot illustrating the incidence rates among European Union’s countries in 2023 among individuals under 20 years of age. Rates are presented with 95% uncertainty intervals (UI) and expressed per 100 000 population.

**Figure 2.**
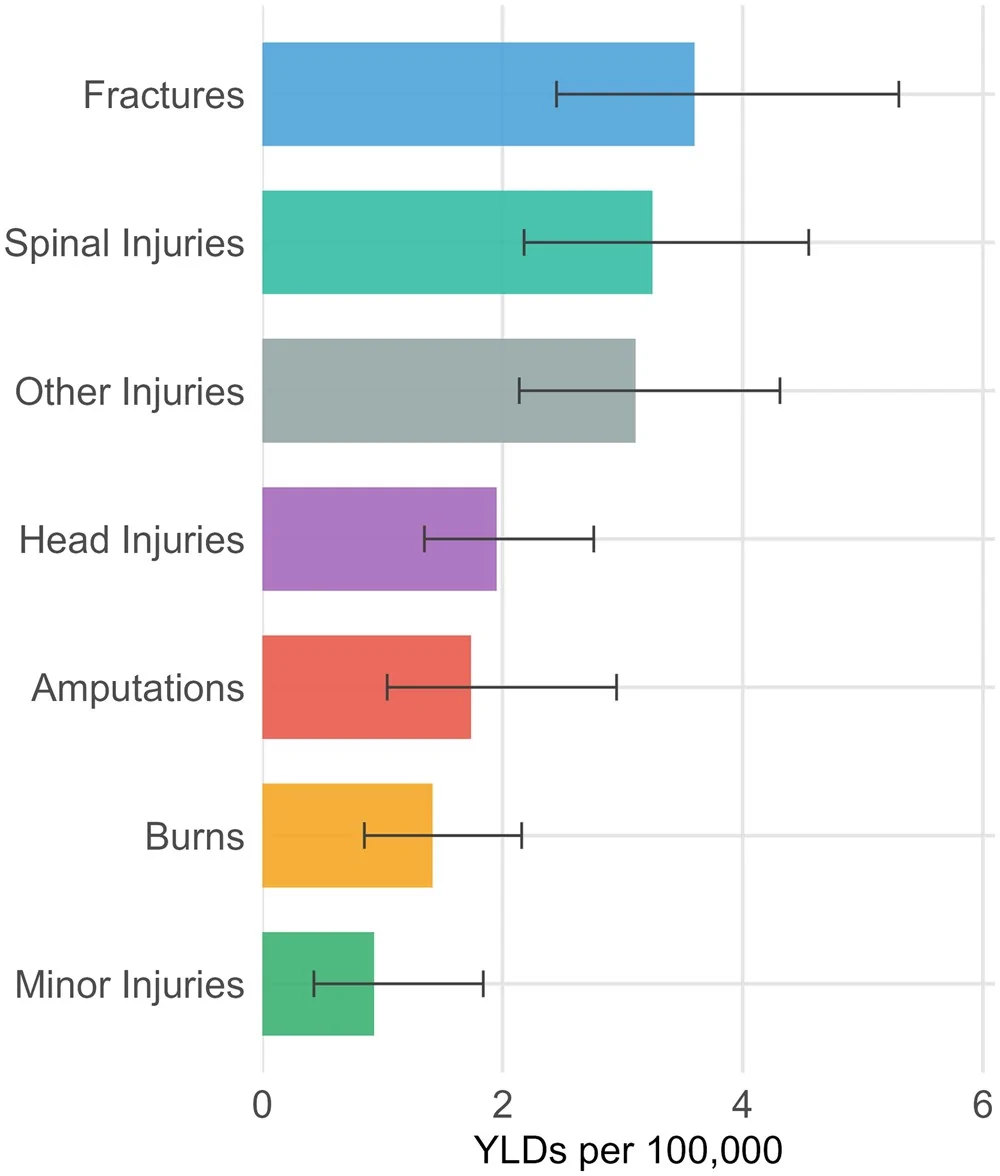
Years Lived with Disability (YLDs) for level-2 injuries in the European Union in 2023 among individuals under 20 years of age. Rates are expressed per 100 000 population and presented with 95% uncertainty intervals (UI).

The analysis of injury burden across EU27 for the population under 20 years of age reveals a complex landscape defined by the interplay between incidence and YLDs (Figs 1 and 2).

Fractures exhibited both a high incidence rate of 115.9 per 100 000 population (95% UI: 92.1–141.9) and a substantial YLD rate of 3.6 (95% UI: 2.45–5.3). Similarly, ‘Other Injuries’ had high values in both categories, with an incidence rate of 97.7 (95% UI: 75.9–128) and a YLD rate of 3.11 (95% UI: 2.14–4.3). Head injuries also fell into this pattern, with an incidence rate of 20.7 (95% UI: 13.8–30.6) and a YLD rate of 1.95 (95% UI: 1.35–2.76). In contrast, Minor Injuries had a high frequency of 264 (95% UI: 208.4–320.5) but a comparatively lower disability burden, with a YLD rate of 0.93 (95% UI: 0.43–1.84).

Supplementary Table S1 shows results for the ‘Hidden Burden’. Despite ranking 41st in incidence with 235 cases (95% UI: 111–480), spinal cord lesions at the neck level ranked first in YLDs, generating 1724 YLDs (95% UI: 1190–2352.8) in 2023. Similarly, spinal cord lesions below the neck level ranked 40th in incidence (362 cases, 95% UI: 151–813) and third in disability burden (1253 YLDs, 95% UI: 810.8–1857.2).

Fractures of the patella, tibia, fibula, or ankle ranked 13th in incidence (10 585 cases, 95% UI: 6181–16 517) but escalated to the second highest rank for YLDs, accounting for 1512.4 YLDs (95% UI: 947.6–2376.8). Moderate to severe traumatic brain injury (TBI) ranked 17th in incidence with 5845 cases (95% UI: 4137–7855) but third in YLDs with 1268.6 (95% UI: 855.2–1844.1). Burns covering less than 20% of the total body surface area presented a hidden burden as well, ranking 12th in incidence (10 971 cases, 95% UI: 5087–21 291), they were the fifth leading cause of YLDs, (919.1, 95% UI: 497.7–1587.9).

Open wounds exhibited the highest incidence rate (121.5, 95% UI: 84.6–159.4) but a relatively lower YLD rate (0.45, 95% UI: 0.21–0.85). Conversely, fractures of the patella, tibia, or fibula showed a much lower rate (11.6, 95% UI: 6.7–18) but a significantly higher YLD rate (1.65, 95% UI: 1.03–2.59). The analysis also identified poisoning requiring urgent care as a pressing issue, with an incidence rate of 10.7 (95% UI: 6–17) and a YLD rate of 0.42 (95% UI: 0.27–0.61).

### Males and females differences in incidence

Injury estimates suggested a sex disparity, with males generally showing higher point estimates of incidence rates and percentage change across several severe injury categories compared with females (Supplementary Table S2). Among children aged 5–14 years in 2023, the estimated number of internal haemorrhage cases in the abdomen and pelvis was 2559 (95% UI: 1364–4083) in males and 1079 (95% UI: 511–1765) in females. Severe chest injuries were estimated at 1803 (95% UI: 930–3126) in males and 715 (95% UI: 341–1373) in females. In the 15–19 age group, open wounds were estimated at 7650 (95% UI: 4476–12 021) in males compared with 3770 (95% UI: 1916–6543) in females. Percentage changes for certain injuries, such as crush injury in the 5–14 age group, were 13.4% (13.4%, 95% UI: 9.6–16.4) in males and 12.2% (95% UI: 8.6–15.7) in females. While males tended to have higher point estimates in several severe trauma categories, females aged 5–14 years showed higher estimated burdens in selected soft tissue and superficial injury categories. For example, superficial injury of any part of the body was estimated at 6458 (95% UI: 2900–11 195) cases in females versus 5912 (95% UI: 3040–9596) in males, and contusion in any part of the body at 5237 (95% UI: 2669–9534) in females compared with 4345 (95% UI: 2464–7517) in males. Poisoning requiring urgent care was also higher in point estimates for females, with 1191 (95% UI: 543–2142) cases compared with 741 (366–1245) in males. Although open wounds were the most frequent injury among females (9715; 95% UI: 5222–15 772), the corresponding estimate was high in males as well (13 599; 95% UI: 7566–21 136). Burns (<20% surface area) were estimated at 1125 cases (95% UI: 439–2412) in females and 939 cases (95% UI: 416–1780) in males. In the 15–19 age group, females also had slightly higher point estimates for poisoning requiring urgent care (437; 95% UI: 196–834) compared with males (384; 95% UI: 202–672), again with overlapping uncertainty intervals.

### Geographical heterogeneity in injury incidence

Most trends exhibited a downward variation. The following results will focus solely on the upward trends, as they represent the opportunity for the most improvement.

The burden of injuries among individuals <20 years of age varied substantially across European nations in 2023 (Fig. 1). Hungary reported the highest total incidence rate of 1085.7 per 100 000 population (95% UI: 917.6–1295.5), positioning it at the top of the burden rankings. This was driven by exceptionally high rates across multiple categories, including ‘Other Injuries’ at 262 (95% UI: 204.9–329.5), fractures at 221.1 (95% UI: 177.7–279), and minor injuries at 494.5 (95% UI: 388.4–604.7). Romania followed with a total rate of 890.7 (95% UI: 756.8–1043.8), characterized by high rates of fractures at 180.9 (95% UI: 144.7–225.4) and burns at 33.9 (95% UI: 18.8–59.1). Malta notably recorded a high rate for fractures at 176.6 (95% UI: 139.8–218.6).

In contrast, Italy’s specific rates were consistently lower than the European average, with fractures at 31.3 (95% UI: 21–42.8), head injuries at 5.3 (95% UI: 3.1–8.7), and burns at 2.4 (95% UI: 1.1–4.7). Even within the lower-burden nations, heterogeneity was observed; for instance, Lithuania reported a fracture rate of 85 (95% UI: 65.8–110.7), which is markedly higher than Italy’s but lower than Hungary’s (Table 1).

**Table 1.** Ranking of European Union countries by incidence rates among individuals under 20 years of age, in 2023 (descending order)^a^

Rank	Country	Total rate (95% UIs)	Spinal injuries	Other injuries	Burns	Head injuries	Fractures	Amputations	Minor injuries
1	Hungary	1085.7 (917.6–1295.5)	1.3 (0.8–2.4)	262 (204.9–329.5)	40.6 (22.4–69.6)	47.6 (34.4–65.1)	221.1 (177.7–279)	18.6 (11.1–30.6)	494.5 (388.4–604.7)
2	Romania	890.7 (756.8–1043.8)	1.1 (0.6–2)	214 (167.5–269.1)	33.9 (18.8–59.1)	38.8 (28.3–51.4)	180.9 (144.7–225.4)	15.2 (8.7–24.5)	406.8 (322.3–489.2)
3	Croatia	805.9 (682.6–970.0)	1 (0.6–1.8)	193.5 (150.5–244.8)	30.8 (17–51.3)	35 (25–48.5)	163.6 (130.2–207)	13.8 (8.2–22.8)	368.1 (292–454.9)
4	Malta	769.4 (658.7–887.8)	1 (0.5–1.7)	129.2 (98.6–169.2)	16.1 (7.7–30.5)	29.3 (18.6–44.9)	176.6 (139.8–218.6)	10.2 (5.6–17.7)	407.1 (326.1–485.5)
5	Bulgaria	767.8 (660.9–901.5)	0.9 (0.6–1.7)	185.2 (145.8–233.6)	28.5 (16.2–49.3)	33.9 (24.7–45.3)	156.5 (125.7–195)	13.1 (7.8–21.4)	349.7 (279–423)
6	Czechia	739.5 (604.0–916.0)	0.9 (0.5–1.6)	177.5 (134.2–228.6)	27.8 (15.4–49.6)	32.3 (22.6–44.7)	150.4 (115.5–195.8)	12.6 (7–21.4)	338 (252.7–431.6)
7	Denmark	713.5 (605.6–843.5)	0.9 (0.5–1.6)	120.1 (92.4–156.5)	14.9 (7.3–27.7)	27.1 (17.2–42.7)	164.1 (131.2–200.5)	9.5 (4.9–16.1)	376.9 (299.8–451)
8	Netherlands	683.4 (571.9–799.7)	0.9 (0.4–1.6)	115.1 (87.7–149.8)	14 (6.7–27)	26.2 (16.6–40)	158.2 (125.8–192.7)	9 (4.8–15.5)	360 (283.1–432.9)
9	Slovakia	669.0 (546.7–804.3)	0.8 (0.4–1.5)	160.9 (123.2–204.2)	25.1 (14.1–43.2)	29.2 (20.5–40)	136.1 (105.6–173)	11.4 (6.6–18.7)	305.4 (234.5–378.7)
10	Austria	603.6 (511.4–699.7)	0.8 (0.4–1.4)	100.9 (76–132.1)	12.8 (6.2–24.3)	22.9 (14.4–35.8)	137.2 (107.5–165.9)	8 (4.2–13.8)	321.1 (254–386.5)
11	Belgium	602.2 (506.3–703.5)	0.8 (0.4–1.4)	101.1 (78.9–130.7)	12.5 (6.1–23.9)	23 (14.8–36)	138.3 (110–170.1)	8 (4.2–13.5)	318.5 (254.4–381.4)
12	Slovenia	594.2 (480.6–727.6)	0.7 (0.4–1.3)	143.2 (107.1–184.6)	21.8 (12.4–38.5)	26.2 (18.3–36.8)	121.2 (93.6–157.3)	10.1 (5.6–16.8)	270.9 (207.8–343.9)
13	Finland	572.3 (488.9–675.4)	0.7 (0.4–1.3)	96.3 (74.6–124.4)	11.6 (5.6–22.1)	22.1 (14–33.5)	132.7 (106–161.8)	7.5 (4–12.9)	301.4 (235.8–364.5)
14	France	569.5 (479.8–668.5)	0.7 (0.4–1.3)	95.5 (74–125.2)	11.8 (5.6–22.5)	21.8 (13.8–33.6)	130.6 (104–160.1)	7.5 (4–12.8)	301.6 (242.6–364.8)
15	Greece	548.4 (454.4–647.7)	0.7 (0.4–1.3)	92.2 (70.1–118.6)	11.2 (5.4–21.4)	21 (13.4–32)	126.6 (99.9–157.8)	7.2 (3.9–12.3)	289.4 (228.8–350)
16	Ireland	539.6 (441.8–642.6)	0.7 (0.3–1.2)	91 (70.3–119.8)	10.9 (5.3–20.9)	20.7 (13.1–32.8)	125.6 (98.9–157.2)	7.1 (3.7–12)	283.5 (217.8–345.5)
17	Germany	534.9 (437.3–658.0)	0.7 (0.3–1.2)	89.8 (68.3–120.6)	11 (5.4–20.6)	20.4 (13–31.7)	123.1 (94.6–154)	7.1 (3.6–12)	282.7 (220.3–349.1)
18	Luxembourg	534.8 (443.1–638.2)	0.7 (0.3–1.2)	90 (69.2–119)	11 (5.3–20.9)	20.4 (12.8–31.4)	123.5 (96.5–153)	7.1 (3.8–11.9)	282.2 (222.1–346.4)
19	Sweden	521.9 (437.5–618.9)	0.7 (0.3–1.2)	88.1 (68.4–114.5)	10.4 (5.1–19.4)	20.2 (12.9–31.7)	121.8 (95.3–147.3)	6.9 (3.8–11.7)	273.9 (213.1–326.2)
20	Portugal	498.9 (427.3–576.2)	0.6 (0.3–1.1)	83.7 (64.9–107.5)	10.3 (4.9–19.4)	19.1 (12.3–29.7)	114.5 (90.7–139.1)	6.6 (3.6–11.3)	263.9 (211.4–317)
21	Spain	451.3 (368.6–543.2)	0.6 (0.3–1)	75.8 (58.1–100.7)	9.1 (4.4–17.8)	17.4 (11–28)	104.4 (81.7–130.1)	6 (3.1–10.1)	238 (184.8–291.3)
22	Lithuania	415.7 (332.4–505.5)	0.5 (0.3–0.9)	100.2 (74.1–132.6)	14.8 (8.4–26.5)	18.6 (12.9–25.7)	85 (65.8–110.7)	7.1 (3.9–11.5)	189.4 (143.2–238)
23	Cyprus	395.9 (316.8–478.1)	0.5 (0.2–0.9)	66.7 (50.8–88.4)	7.9 (3.8–14.8)	15.4 (9.7–24.7)	92.4 (71.3–113.5)	5.2 (2.6–8.8)	207.9 (161.7–253.9)
24	Latvia	367.9 (286.9–455.9)	0.4 (0.2–0.8)	88.4 (66.8–119.3)	13.3 (7.4–23.6)	16.4 (11.5–23)	75.2 (57.4–97.1)	6.3 (3.5–10.5)	168 (123.4–212)
25	Estonia	340.1 (266.3–426.0)	0.4 (0.2–0.8)	81.7 (61.6–111)	12.2 (6.8–22.1)	15.3 (10.4–21.3)	69.5 (51.3–91.5)	5.8 (3.2–9.5)	155.2 (113–197.5)
26	Poland	328.5 (239.4–414.9)	0.4 (0.2–0.7)	79 (57–108.8)	11.7 (6.4–20.7)	14.7 (10.1–21.1)	67.2 (47.9–91.1)	5.6 (3–9.3)	150 (104.2–190.6)
27	Italy	131.9 (94.3–175.5)	0.2 (0.1–0.3)	22.2 (14.4–30.3)	2.4 (1.1–4.7)	5.3 (3.1–8.7)	31.3 (21–42.8)	1.7 (0.8–3)	68.9 (47.8–92.4)

a Rates are presented with 95% uncertainty intervals (UI) and expressed per 100 000 population.

### Long-term systemic increases (1990–2023)

The analysis of injury rates showed statistically significant increases in Eastern Europe between 1990 and 2023, particularly within the under-5 demographic (Supplementary Table S2). Romania exhibits the most severe long-term trajectory among the analysed nations. For children <5 years, the incidence rate of poisoning requiring urgent care rose by 77.9% (95% UI: 55.5%–96.0%). Severe thermal injuries also surged, with burns covering 20% or more of the body surface area increasing by 77.8% (95% UI: 54.5%–96.2%). Estimates for the under-20 demographic indicated a 36.9% increase (95% UI: 22.7%–51.4%) in drowning rates and a 38.9% increase (95% UI: 22.3%–54.9%) in burns involving less than 20% of the body surface area.

### Mid-term trends and accelerations (2010–2023)

The mid-term period highlights accelerating risks in Southern and Central Europe, bridging the gap between historical baselines and recent acute spikes (Supplementary Table S2). For children under 5 years, the rate of severe chest injuries rose by 21.3% (95% UI: 17.1%–26.4%) between 2010 and 2023. This period also saw a 21.2% increase (95% UI: 16.3%–27.1%) in skull fractures and a 21.1% rise (95% UI: 16.3%–26.5%) in internal abdominal/pelvic haemorrhages.

Countries exhibited distinct injury patterns during this timeframe. Portugal showed increases in the 5–14 year age group, specifically regarding poisonings, which rose by 12.7% (95% UI: 3.0%–22.5%) for both sexes combined. Sex disparities were notable, with female rates rising by 23.0% (95% UI: 9.3%–37.2%) compared to a substantial stability for males. Hip fractures in this demographic in Portugal also rose by 12.4% (95% UI: 3.5%–23.5%). Hungary experienced localized mid-term increases in trauma among children under 5 years, with male incidence rates for severe chest injuries rising by 23.4% (95% UI: 9.9%–41.0%). Similarly, male rates for skull fractures increased by 23.4% (95% UI: 9.9%–39.5%), and internal haemorrhages by 23.4% (95% UI: 10.5%–39.9%). Lithuania recorded notable increases in the under-5 demographic, where male incidence rates for head injuries rose by 15.9% (95% UI: 0.7%–30.1%), fractures increased by 16.2% (95% UI: 0.9%–31.2%), and minor injuries rose by 16.2% (95% UI: 0.3%–30.3%). Additionally, Croatia saw a 15.0% (95% UI: 1.8%–29.9%) rise in burns involving less than 20% of the body surface area for females aged 5–14 years.

### Short-term spikes (2020–2023)

Percentage trends from 2020 to 2023 indicate statistically significant increases in injury rates across the European Union aggregates (Supplementary Table S2). For the age group 5–14 years, the overall rate for ‘Other Injuries’ increased by 12.5% (95% UI: 9.3%–15.3%), while fractures rose by 12.4% (95% UI: 9.3%–15.6%). The fracture increase was comparable in males and female (13.6%; 95% UI: 9.7%–17.3% and 10.8%; 95% UI: 6.4%–15.3%, respectively). Head injuries in this demographic increased by 12.4% (95% UI: 9.4%–15.5%), and spinal injuries saw a similar rise of 12.4% (95% UI: 9.2%–15.3%). At a more granular level, the EU observed short-term increases in crush injuries of 13.0% (95% UI: 9.7%–15.4%). Internal haemorrhages in the abdomen and pelvis increased by 12.9% (95% UI: 9.7%–16.1%), severe chest haemorrhages by 12.9% (95% UI: 9.6%–16.0%), and burns covering 20% or more of the body surface area by 12.9% (95% UI: 9.9%–15.3%). Among the older adolescent demographic (15–19 years), EU estimates showed a 7.1% increase (95% UI: 0.7%–11.5%) in burns, a 7.0% increase (95% UI: 0.2%–11.4%) in head injuries, and a 9.2% increase (95% UI: 3.2%–13.6%) in unilateral lower limb amputations.

While EU aggregates show markedly rises, specific national data reveals localized outliers. Italy presented the highest short-term increases for the period 2020–2023 in the <5 age group. Severe chest injuries in this demographic surged by 79.1% (95% UI: 66.4%–92.3%), reaching, in absolute numbers, 77 cases (95% UI: 41–135) in 2023. Similarly, skull fractures increased by 79.0% (95% UI: 65.4%–93.3%) and internal haemorrhages by 78.8% (95% UI: 66.2%–92.5%), indicating a recent escalation in severe trauma.

## Discussion

This study presents, to our knowledge, the first comprehensive analysis of interpersonal violence injuries among the paediatric population in the EU27 using GBD 2023 estimates. The findings reveal a stark epidemiological dichotomy across the European Union, characterized by deep-seated systemic burdens in Eastern Europe and emerging, acute escalations in Southern Europe. By integrating a ‘Disability Drivers’ analysis, we illustrate that traditional incidence-based surveillance masks the true public health cost of violence, which is disproportionately driven by low-frequency, high-severity injuries. These results suggest that the determinants of paediatric safety and the resulting trauma landscapes in the European Union remain profoundly divided. In particular, this geographical heterogeneity may reflect structural disparities in public health and child protection mechanisms, although the relative contributions of true burden differences and surveillance variation remain difficult to disentangle. For example, the persistent high burden in Eastern Europe, particularly in Hungary and Romania, may be partly attributed to inadequate mitigation of violence against vulnerable populations [12]. In this light, the substantial long-term increase in poisoning and burn injuries among Romanian children under 5 years is indicative of chronic neglect or hazardous household environments [13]. These multi-decadal trends suggest that the drivers of violence in these regions are entrenched in socioeconomic factors that require long-term structural interventions rather than transient awareness campaigns. An important consideration is whether the elevated burden in high-ranking nations, such as Hungary and Malta, may partly represent a surveillance artefact driven by superior detection of minor injuries. Indeed, minor injuries comprise a substantial proportion of the total incidence in these regions (45.5% in Hungary). However, several observations argue against a purely artefactual explanation. If high incidence were merely a product of sensitive reporting systems capturing superficial trauma, one might expect a decoupling of minor and severe injury rates. Instead, the analysis reveals a consistent gradient: Hungary’s rate of fractures (221.1 per 100 000) is nearly seven times that of Italy (31.3), and its rate of ‘Other Injuries’, is over ten times higher. This synchronicity between low-severity and high-severity markers is more consistent with genuine variation in violence exposure, although a contribution of differential reporting cannot be definitively excluded. Previous analyses of injury surveillance in European settings have similarly noted that cross-country differences likely reflect a combination of true burden variation and ascertainment differences [14].

Conversely, the exceptionally low incidence rates observed in Italy warrants scrutiny regarding potential misclassification bias. While Italy presents the lowest burden in the EU27 (132.0 per 100 000), this may not solely reflect a protective environment but rather a systemic tendency to classify unwitnessed trauma as unintentional rather than violent. Unlike the potential surveillance artifact of minor injuries discussed above, this could represent a misclassification of intent: severe injuries such as fractures are undoubtedly treated by the healthcare system, but if the violent cause is not explicitly disclosed or investigated, they may be coded as accidental or indeterminate. Such misclassification has been documented in other Southern European settings [15]. Consequently, the Italian estimates should be interpreted as a conservative lower bound, potentially masking a silent prevalence of domestic violence that evades administrative capture due to cultural stigma or less aggressive forensic screening in emergency settings.

This hypothesis of hidden violence in the South is supported by a counter-narrative of acute destabilization. While Italy maintains the lowest overall incidence rate in the region, the alarming surge in severe chest injuries and skull fractures among children aged 5–14 between 2020 and 2023 points to a specific, localized crisis. Unlike the systemic patterns in the East, these injuries are high-specificity markers for severe physical trauma. Occurring in the shadow of the COVID-19 pandemic, this spike likely captures the collateral damage of societal restrictions, where confinement intensified domestic stressors while severing access to school-based protective surveillance [16, 17]. However, other explanations, including changes in healthcare utilization, coding practices during the pandemic, or catch-up reporting, should also be considered. The observation that these rates have not returned to baseline warrants further investigation to determine whether the pandemic catalysed a lasting shift in family safety dynamics.

Beyond the ‘where’ and ‘when’ of violence, this study fundamentally challenges ‘how’ we prioritize injury prevention through the identification of the ‘Hidden Burden’. Public health resource allocation has historically favoured high-incidence conditions. However, our rank-discordance analysis illustrates that injuries such as spinal cord lesions, which rank 41st in incidence but first in Years Lived with Disability, represent the major drivers of the health burden. The lifelong disability associated with these lesions exerts a human and economic toll that far exceeds that of high-frequency, low-severity injuries like superficial wounds. Similarly, the recognized pattern of fractures and traumatic brain injuries underscores the need for prevention strategies that specifically target the mechanisms of blunt force trauma [18], moving beyond simple reduction of event frequency to the mitigation of injury severity.

These findings must be interpreted within the context of several limitations. First, GBD estimates for incidence and YLDs generally exhibit wider uncertainty intervals compared to mortality metrics, owing to variability in the quality and availability of non-fatal data. This is particularly relevant for the present analysis, as only 15 of the 27 EU countries possess primary data sources for interpersonal violence, and data depth varies markedly, from a single dataset in several countries to more than 15 sources in others. For countries with limited or no primary data, GBD estimates rely more heavily on statistical modelling using covariates and regional patterns, which may reduce the precision of country-specific estimates and should be considered when interpreting cross-country comparisons. The wide uncertainty intervals for smaller nations such as Malta and Estonia further reflect this data scarcity. In several EU nations, the absence of centralized data from outpatient services, general practitioners, and family paediatricians limits the accuracy of estimates for injuries that do not result in hospitalization. Second, while the GBD hierarchy identifies injuries as resulting from interpersonal violence, it lacks specific perpetrator data, limiting the ability to distinguish between peer-to-peer violence and maltreatment by caregivers without inference from age and injury patterns. Third, given the sensitive nature of paediatric violence, underreporting remains a pervasive issue [19], suggesting that these estimates likely represent a conservative lower bound of the true burden.

In conclusion, the epidemiology of paediatric violence in the European Union is transitioning from a homogenous concern to a polarized landscape defined by chronic neglect in the East and acute physical trauma in the South. Addressing this requires a paradigm shift in policy that transcends reactive crisis management in favour of proactive, equity-oriented prevention. This necessitates a dual strategy: implementing structural reforms to dismantle the socioeconomic determinants of chronic neglect in high-burden regions and establishing active early-detection systems to preempt the escalating severity of physical abuse in historically ‘safer’ regions where intent misclassification may remain a barrier to protection. Ultimately, prioritizing the ‘Hidden Burdens’ of disability will ensure that health systems address not just the most common injuries, but those that exact the highest price from the lives of children and adolescents [20].

## Supplementary Material

ckag078_Supplementary_Data

## Data Availability

The estimates used in this study are publicly available in the Global Health Data Exchange (GHDx) (https://ghdx.healthdata.org/) and the GBD Results tools (https://vizhub.healthdata.org/gbd-results/). Key points Interpersonal violence injuries in children and adolescents showed substantial variability in incidence rates, with the highest burden in Eastern Europe countries and the lowest in Italy, indicating profound structural and surveillance disparities across countries. In Eastern Europe, violence-related injuries show a long-term, progressive increase. In contrast, Southern Europe has historically low incidence but displays abrupt recent spikes in severe trauma. Disability is driven by low-frequency, high-severity injuries: several injuries with relatively low incidence generated a disproportionate share of long-term disability, demonstrating that incidence alone substantially underestimates the true public-health impact.
