# Supplementary material for: Interpersonal violence injuries among children and adolescents in the European Union from 1990 to 2023, a Global Burden of Disease study
Source: Eur J Public Health. 2026 Jun 16;36(4):ckag078. doi: 10.1093/eurpub/ckag078 (PMC13275158; doi:10.1093/eurpub/ckag078)
Supplement: ckag078_Supplementary_Data [file ckag078_supplementary_data.docx]

# Supplementary Tables

Table S1 3

Table S2, by Country 5

Austria 5

Belgium 8

Bulgaria 10

Croatia 15

Cyprus 19

Czechia 22

Denmark 23

Estonia 26

European Union 29

Finland 33

France 34

Germany 37

Greece 40

Hungary 41

Ireland 51

Italy 54

Latvia 67

Lithuania 70

Luxembourg 76

Malta 79

Netherlands 82

Poland 85

Portugal 94

Romania 98

Slovakia 107

Slovenia 114

Spain 116

Sweden 118

**Table S1**. Top 25 level 3 disability drivers in the European Union in 2023 among individuals aged <20 years. Yellow denotes ‘hidden burden’ (i.e., higher disability than frequency rank). Incidence and Years Lived with Disability (YLDs) are reported with 95% uncertainty intervals (UI).

| **Category** | **Inc Rank** | **Incidence (N) [95% UI]** | **YLD Rank** | **YLDs (N) [95% UI]** | **Hidden Burden** |
| --- | --- | --- | --- | --- | --- |
| Spinal cord lesion at neck level | 41 | 235 (111-480) | 1 | 1,724 (1,190-2,352.8) | YES |
| Fracture of patella, tibia or fibula, or a... | 13 | 10,585 (6,181-16,517) | 2 | 1,512.4 (947.6-2,376.8) | YES |
| Moderate/Severe TBI | 17 | 5,845 (4,137-7,855) | 3 | 1,268.6 (855.2-1,844.1) | YES |
| Spinal cord lesion below neck level | 40 | 362 (151-813) | 4 | 1,253 (810.8-1,857.2) | YES |
| Burns, <20% total burned surface area with... | 12 | 10,971 (5,087-21,291) | 5 | 919.1 (497.7-1,587.9) | YES |
| Internal hemorrhage in abdomen and pelvis | 8 | 17,505 (10,718-25,381) | 6 | 692.7 (439.3-1,055.4) | YES |
| Fracture of pelvis | 34 | 2,178 (749-5,402) | 7 | 669.4 (423.9-997.4) | YES |
| Severe chest Injury | 11 | 11,902 (7,518-18,414) | 8 | 552.4 (298.1-1,012.3) | YES |
| Multiple fractures, dislocations, crashes,... | 19 | 4,686 (2,557-7,845) | 9 | 532.7 (319.5-817.5) | YES |
| Minor TBI | 10 | 13,152 (7,543-21,353) | 10 | 521.4 (350.4-762.1) |  |
| Amputation of thumb | 33 | 2,179 (786-5,392) | 11 | 480.6 (229.3-919.9) | YES |
| Open wound(s) | 1 | 111,325 (77,450-146,024) | 12 | 411.8 (192.6-778) |  |
| Poisoning requiring urgent care | 16 | 9,792 (5,538-15,581) | 13 | 381.9 (251.2-560.4) | YES |
| Burns, >=20% total burned surface area or ... | 38 | 836 (489-1,292) | 14 | 374 (243.9-538.6) | YES |
| Contusion in any part of the body | 3 | 46,512 (29,200-69,840) | 15 | 300.6 (133-611.4) |  |
| Amputation of lower limbs, bilateral | 46 | 121 (38-285) | 16 | 293.8 (184-450) | YES |
| Amputation of upper limbs, bilateral | 44 | 141 (55-303) | 17 | 276.3 (176.6-396.3) | YES |
| Nerve injury | 18 | 5,095 (2,362-10,403) | 18 | 262.2 (169.3-383.9) |  |
| Fracture of skull | 9 | 15,226 (8,540-25,515) | 19 | 245.7 (154-376.3) |  |
| Amputation of fingers (excluding thumb) | 26 | 2,603 (1,099-5,142) | 20 | 236.4 (96.7-523.6) | YES |
| Fracture of face bones | 5 | 22,551 (14,562-34,947) | 21 | 192.4 (106.5-318.2) |  |
| Fracture of femur, other than femoral neck | 20 | 4,638 (2,244-9,185) | 22 | 183.1 (104.9-294.7) |  |
| Amputation of toe/toes | 37 | 1,592 (552-3,613) | 23 | 177.8 (73.2-394.8) | YES |
| Injury to eyes | 7 | 18,329 (9,668-28,940) | 24 | 134.9 (37.4-301.4) |  |
| Muscle and tendon injuries, including spra... | 4 | 22,656 (14,307-34,243) | 25 | 118.7 (53.3-252.2) |  |

**Table S2.** Country-level analysis of significantly increasing trends in level 3 specific injuries, based on incidence rates (1990–2023). Only significant increases are shown (i.e., with lower bound of 95% uncertainty interval >0). Empty cells indicate non-significant trends.

## Austria

| **Age** | **Category** | **Period** | **Male %Change** | **Male Incidence 2023** | **Female %Change** | **Female Incidence 2023** | **Both %Change** | **Both Incidence 2023** |
| --- | --- | --- | --- | --- | --- | --- | --- | --- |
| 5-14 years | Fracture of skull | 2020-2023 | +10.3% (+0.3 to +19.1) | 52 (25-92) |  |  | +10.0% (+2.7 to +16.8) | 73 (34-129) |
|  | Internal hemorrhage in abdomen and pelvis | 2020-2023 | +10.3% (+1.8 to +19.5) | 39 (20-65) |  |  | +9.9% (+3.4 to +17.6) | 55 (29-91) |
|  | Severe chest Injury | 2020-2023 | +10.3% (+0.7 to +20.1) | 31 (16-54) |  |  | +9.9% (+2.8 to +17.6) | 42 (21-75) |
|  | Fracture of face bones | 2020-2023 | +10.3% (+0.6 to +20.3) | 70 (34-118) |  |  | +9.8% (+2.4 to +17.1) | 104 (49-178) |
|  | Fracture of hand (wrist and other distal part of hand) | 2020-2023 | +10.2% (+0.4 to +19.2) | 53 (26-96) |  |  | +9.7% (+1.9 to +17.0) | 87 (42-161) |
|  | Injury to eyes | 2020-2023 | +10.3% (+0.6 to +19.6) | 46 (22-81) |  |  | +9.7% (+2.8 to +16.8) | 79 (37-146) |
|  | Fracture of sternum and/or fracture of one or more ribs | 2020-2023 | +10.2% (+0.9 to +20.1) | 14 (6-29) |  |  | +9.7% (+2.7 to +17.8) | 22 (8-46) |
|  | Amputation of fingers (excluding thumb) | 2020-2023 |  |  |  |  | +9.7% (+2.1 to +17.2) | 8 (3-18) |
|  | Open wound(s) | 2020-2023 | +10.3% (+0.8 to +19.1) | 245 (136-386) |  |  | +9.6% (+2.7 to +17.4) | 414 (229-674) |
|  | Moderate/Severe TBI | 2020-2023 | +10.3% (+0.0 to +19.7) | 9 (5-14) |  |  | +9.6% (+3.0 to +16.7) | 14 (8-23) |
|  | Nerve injury | 2020-2023 | +10.3% (+1.2 to +20.7) | 12 (5-24) |  |  | +9.6% (+2.8 to +18.5) | 21 (8-47) |
|  | Amputation of thumb | 2020-2023 | +10.2% (+0.0 to +21.1) | 5 (1-11) |  |  | +9.6% (+1.5 to +18.6) | 9 (3-23) |
|  | Minor TBI | 2020-2023 | +10.3% (+1.9 to +19.8) | 34 (17-61) |  |  | +9.6% (+2.2 to +17.1) | 61 (30-108) |
|  | Multiple fractures, dislocations, crashes, wounds, pains, and strains | 2020-2023 | +10.3% (+0.7 to +19.0) | 7 (3-14) |  |  | +9.6% (+2.6 to +17.4) | 13 (6-25) |
|  | Crush injury | 2020-2023 | +10.2% (+1.0 to +20.4) | 0 (0-0) |  |  | +9.6% (+2.2 to +17.3) | 0 (0-1) |
|  | Fracture of foot bones except ankle | 2020-2023 | +10.2% (+0.2 to +20.1) | 10 (4-21) |  |  | +9.6% (+2.2 to +17.8) | 17 (6-36) |
|  | Spinal cord lesion at neck level | 2020-2023 | +10.2% (+1.2 to +19.7) | 0 (0-1) |  |  | +9.6% (+2.7 to +16.8) | 1 (0-2) |
|  | Spinal cord lesion below neck level | 2020-2023 | +10.3% (+0.1 to +18.9) | 1 (0-2) |  |  | +9.6% (+2.2 to +17.3) | 1 (0-3) |
|  | Burns, <20% total burned surface area without lower airway burns | 2020-2023 |  |  |  |  | +9.6% (+2.0 to +18.5) | 34 (14-67) |
|  | Fracture of patella, tibia or fibula, or ankle | 2020-2023 |  |  |  |  | +9.6% (+2.3 to +17.0) | 36 (16-64) |
|  | Asphyxiation | 2020-2023 |  |  |  |  | +9.5% (+1.4 to +18.8) | 12 (3-32) |
|  | Fracture of vertebral column | 2020-2023 |  |  |  |  | +9.5% (+1.4 to +18.0) | 14 (6-29) |
|  | Amputation of upper limbs, bilateral | 2020-2023 | +10.2% (+0.6 to +20.5) | 0 (0-0) |  |  | +9.5% (+2.3 to +17.7) | 0 (0-1) |
|  | Drowning and nonfatal submersion | 2020-2023 | +10.3% (+0.7 to +20.0) | 5 (2-13) |  |  | +9.5% (+2.8 to +17.7) | 11 (3-29) |
|  | Fracture of femur, other than femoral neck | 2020-2023 | +10.2% (+1.2 to +19.8) | 7 (3-14) |  |  | +9.5% (+2.8 to +17.4) | 12 (5-26) |
|  | Amputation of toe/toes | 2020-2023 | +10.2% (+1.2 to +21.3) | 3 (1-8) |  |  | +9.5% (+2.0 to +18.3) | 7 (2-16) |
|  | Amputation of upper limb, unilateral | 2020-2023 |  |  |  |  | +9.5% (+1.6 to +17.7) | 0 (0-1) |
|  | Dislocation of knee | 2020-2023 |  |  |  |  | +9.5% (+2.2 to +17.6) | 10 (3-26) |
|  | Muscle and tendon injuries, including sprains and strains lesser dislocations | 2020-2023 | +10.3% (+1.0 to +20.4) | 36 (18-63) |  |  | +9.5% (+2.7 to +17.3) | 72 (36-129) |
|  | Lower airway burns | 2020-2023 |  |  |  |  | +9.5% (+1.4 to +17.8) | 1 (0-1) |
|  | Fracture of clavicle, scapula, or humerus | 2020-2023 | +10.3% (+0.2 to +20.6) | 21 (9-40) |  |  | +9.5% (+1.6 to +18.4) | 41 (17-80) |
|  | Dislocation of shoulder | 2020-2023 | +10.3% (+0.2 to +21.3) | 7 (3-18) |  |  | +9.4% (+1.2 to +18.1) | 15 (5-36) |
|  | Fracture of radius and/or ulna | 2020-2023 |  |  |  |  | +9.4% (+1.5 to +18.0) | 30 (13-57) |
|  | Contusion in any part of the body | 2020-2023 | +10.3% (+0.8 to +19.8) | 106 (55-187) |  |  | +9.4% (+1.6 to +18.0) | 230 (121-420) |
|  | Superficial injury of any part of the body | 2020-2023 | +10.3% (+0.7 to +19.5) | 138 (69-228) |  |  | +9.4% (+1.6 to +17.3) | 284 (133-485) |
|  | Dislocation of hip | 2020-2023 |  |  |  |  | +9.4% (+1.2 to +18.5) | 7 (2-17) |
|  | Foreign body in GI and urogenital system | 2020-2023 | +10.2% (+0.9 to +21.2) | 5 (2-12) |  |  | +9.4% (+1.3 to +17.7) | 9 (3-22) |
|  | Foreign body in ear | 2020-2023 | +10.2% (+0.3 to +19.4) | 6 (2-13) |  |  | +9.4% (+1.5 to +17.4) | 11 (3-27) |
|  | Effect of different environmental factors | 2020-2023 |  |  |  |  | +9.4% (+1.1 to +16.9) | 12 (4-29) |
|  | Foreign body in respiratory system | 2020-2023 | +10.2% (+0.1 to +20.5) | 3 (1-8) |  |  | +9.3% (+2.2 to +17.7) | 7 (2-17) |
|  | Complications following therapeutic procedures | 2020-2023 | +10.2% (+0.5 to +19.1) | 2 (1-3) |  |  | +9.3% (+1.2 to +17.0) | 3 (2-7) |
|  | Poisoning requiring urgent care | 2020-2023 | +10.3% (+0.9 to +19.8) | 9 (4-15) |  |  | +9.2% (+1.4 to +18.8) | 23 (11-42) |
|  | Amputation of lower limbs, bilateral | 2020-2023 | +10.2% (+0.3 to +19.5) | 0 (0-1) |  |  | +9.2% (+0.9 to +19.0) | 0 (0-1) |
|  | Fracture of pelvis | 2020-2023 |  |  |  |  | +9.2% (+0.1 to +17.7) | 10 (3-27) |
|  | Burns, >=20% total burned surface area or >= 10% burned surface area if head/neck or hands/wrist involved w/o lower airway burns | 2020-2023 | +10.2% (+1.0 to +19.4) | 0 (0-1) |  |  | +9.2% (+0.8 to +17.7) | 1 (0-2) |
|  | Amputation of lower limb, unilateral | 2020-2023 |  |  |  |  | +9.1% (+0.4 to +17.9) | 0 (0-0) |
|  | Fracture of hip | 2020-2023 | +10.2% (+1.0 to +19.7) | 1 (0-2) |  |  | +8.9% (+0.2 to +17.5) | 2 (1-5) |

## Belgium

| **Age** | **Category** | **Period** | **Male %Change** | **Male Incidence 2023** | **Female %Change** | **Female Incidence 2023** | **Both %Change** | **Both Incidence 2023** |
| --- | --- | --- | --- | --- | --- | --- | --- | --- |
| 5-14 years | Amputation of lower limb, unilateral | 2020-2023 |  |  |  |  | +11.3% (+0.8 to +23.8) | 0 (0-0) |
|  | Dislocation of knee | 2020-2023 |  |  |  |  | +11.2% (+1.7 to +23.1) | 15 (5-37) |
|  | Fracture of hip | 2020-2023 |  |  |  |  | +11.2% (+1.4 to +21.9) | 3 (1-7) |
|  | Amputation of upper limbs, bilateral | 2020-2023 |  |  |  |  | +11.2% (+0.3 to +23.5) | 1 (0-1) |
|  | Fracture of patella, tibia or fibula, or ankle | 2020-2023 |  |  |  |  | +11.2% (+0.1 to +22.7) | 53 (25-95) |
|  | Multiple fractures, dislocations, crashes, wounds, pains, and strains | 2020-2023 |  |  |  |  | +11.1% (+2.0 to +21.5) | 19 (8-37) |
|  | Spinal cord lesion below neck level | 2020-2023 |  |  |  |  | +11.1% (+0.6 to +23.9) | 2 (1-5) |
|  | Open wound(s) | 2020-2023 |  |  |  |  | +11.1% (+0.2 to +23.8) | 606 (339-988) |
|  | Fracture of vertebral column | 2020-2023 |  |  |  |  | +11.1% (+0.7 to +22.5) | 20 (8-42) |
|  | Complications following therapeutic procedures | 2020-2023 |  |  |  |  | +11.1% (+0.2 to +22.9) | 5 (2-10) |
|  | Foreign body in ear | 2020-2023 |  |  |  |  | +11.1% (+0.0 to +22.6) | 16 (5-39) |
|  | Amputation of upper limb, unilateral | 2020-2023 |  |  |  |  | +11.1% (+0.3 to +24.7) | 1 (0-2) |
|  | Burns, >=20% total burned surface area or >= 10% burned surface area if head/neck or hands/wrist involved w/o lower airway burns | 2020-2023 |  |  |  |  | +11.0% (+1.2 to +21.5) | 2 (1-3) |
|  | Dislocation of hip | 2020-2023 |  |  |  |  | +11.0% (+0.9 to +22.7) | 10 (3-26) |
|  | Minor TBI | 2020-2023 |  |  |  |  | +11.0% (+0.2 to +22.5) | 89 (44-157) |
|  | Contusion in any part of the body | 2020-2023 |  |  |  |  | +11.0% (+1.2 to +23.2) | 341 (182-604) |
|  | Foreign body in GI and urogenital system | 2020-2023 |  |  |  |  | +11.0% (+0.1 to +22.1) | 13 (4-33) |
|  | Asphyxiation | 2020-2023 |  |  |  |  | +11.0% (+0.1 to +22.0) | 17 (4-46) |
|  | Superficial injury of any part of the body | 2020-2023 |  |  |  |  | +11.0% (+0.5 to +21.5) | 421 (203-697) |
|  | Muscle and tendon injuries, including sprains and strains lesser dislocations | 2020-2023 |  |  |  |  | +11.0% (+1.1 to +21.6) | 107 (54-185) |
|  | Lower airway burns | 2020-2023 |  |  |  |  | +11.0% (+0.2 to +22.7) | 1 (0-2) |
|  | Injury to eyes | 2020-2023 |  |  |  |  | +11.0% (+0.6 to +22.6) | 116 (55-215) |
|  | Fracture of radius and/or ulna | 2020-2023 |  |  |  |  | +11.0% (+1.5 to +23.0) | 45 (20-84) |
|  | Dislocation of shoulder | 2020-2023 |  |  |  |  | +11.0% (+1.0 to +21.7) | 21 (7-53) |
|  | Poisoning requiring urgent care | 2020-2023 |  |  |  |  | +10.9% (+1.1 to +22.1) | 34 (16-60) |
|  | Burns, <20% total burned surface area without lower airway burns | 2020-2023 |  |  |  |  | +10.9% (+0.8 to +23.6) | 50 (21-97) |

## Bulgaria

| **Age** | **Category** | **Period** | **Male %Change** | **Male Incidence 2023** | **Female %Change** | **Female Incidence 2023** | **Both %Change** | **Both Incidence 2023** |
| --- | --- | --- | --- | --- | --- | --- | --- | --- |
| <5 years | Severe chest Injury | 1990-2023 | +29.1% (+12.6 to +46.2) | 193 (118-288) | +17.0% (+1.7 to +33.9) | 84 (46-146) | +25.5% (+12.1 to +38.1) | 278 (167-423) |
|  | Fracture of skull | 1990-2023 | +29.1% (+12.5 to +47.8) | 97 (55-158) | +16.9% (+3.0 to +32.0) | 44 (21-86) | +25.3% (+11.7 to +38.4) | 141 (79-240) |
|  | Internal hemorrhage in abdomen and pelvis | 1990-2023 | +29.0% (+13.4 to +45.9) | 325 (215-454) | +16.9% (+3.1 to +32.6) | 151 (91-237) | +25.2% (+13.7 to +38.3) | 475 (308-693) |
|  | Fracture of face bones | 1990-2023 | +29.0% (+12.4 to +44.6) | 167 (105-257) | +17.1% (+3.0 to +32.7) | 95 (54-158) | +24.6% (+11.9 to +37.2) | 262 (163-409) |
|  | Fracture of sternum and/or fracture of one or more ribs | 1990-2023 | +29.2% (+12.8 to +46.7) | 14 (7-27) | +17.0% (+2.2 to +32.7) | 8 (3-19) | +24.6% (+11.5 to +37.2) | 22 (10-46) |
|  | Moderate/Severe TBI | 1990-2023 | +28.9% (+11.9 to +45.8) | 115 (87-151) | +17.0% (+1.6 to +32.0) | 76 (54-107) | +24.0% (+11.2 to +36.7) | 191 (145-256) |
|  | Crush injury | 1990-2023 | +28.8% (+12.7 to +45.4) | 6 (3-9) | +17.0% (+2.5 to +31.5) | 4 (2-7) | +23.7% (+11.6 to +37.2) | 9 (5-17) |
|  | Amputation of upper limbs, bilateral | 1990-2023 | +29.0% (+12.8 to +47.7) | 2 (1-5) | +17.1% (+1.8 to +32.8) | 2 (1-4) | +23.7% (+11.3 to +36.8) | 4 (2-9) |
|  | Fracture of hand (wrist and other distal part of hand) | 1990-2023 | +29.0% (+12.0 to +45.8) | 106 (65-158) | +17.1% (+2.5 to +33.5) | 79 (43-133) | +23.7% (+11.4 to +35.3) | 185 (107-286) |
|  | Amputation of fingers (excluding thumb) | 1990-2023 | +29.1% (+11.3 to +46.8) | 39 (20-71) | +17.2% (+1.1 to +33.4) | 32 (13-67) | +23.5% (+9.8 to +36.5) | 71 (34-136) |
|  | Open wound(s) | 1990-2023 | +29.1% (+11.8 to +45.3) | 1,275 (938-1,646) | +17.1% (+2.3 to +32.8) | 1,026 (717-1,377) | +23.5% (+10.2 to +35.4) | 2,301 (1,666-3,028) |
|  | Injury to eyes | 1990-2023 | +29.1% (+12.9 to +46.0) | 134 (83-208) | +17.2% (+1.3 to +33.9) | 111 (56-189) | +23.4% (+11.2 to +36.6) | 246 (140-394) |
|  | Spinal cord lesion below neck level | 1990-2023 | +28.8% (+12.8 to +43.9) | 3 (1-6) | +17.0% (+1.9 to +31.1) | 2 (1-6) | +23.3% (+10.7 to +34.3) | 6 (2-11) |
|  | Fracture of patella, tibia or fibula, or ankle | 1990-2023 | +29.1% (+12.9 to +44.3) | 134 (82-204) | +17.1% (+2.9 to +33.1) | 118 (65-196) | +23.2% (+11.6 to +34.6) | 251 (147-396) |
|  | Multiple fractures, dislocations, crashes, wounds, pains, and strains | 1990-2023 | +29.0% (+12.7 to +45.4) | 76 (44-125) | +17.0% (+2.8 to +32.4) | 67 (35-118) | +23.1% (+11.2 to +35.4) | 143 (78-242) |
|  | Minor TBI | 1990-2023 | +29.1% (+12.3 to +45.5) | 81 (49-131) | +17.0% (+1.1 to +33.6) | 72 (37-124) | +23.1% (+8.6 to +35.6) | 153 (86-255) |
|  | Spinal cord lesion at neck level | 1990-2023 | +29.0% (+11.9 to +44.6) | 2 (1-4) | +16.7% (+1.9 to +32.5) | 2 (1-5) | +23.1% (+8.9 to +35.2) | 4 (2-9) |
|  | Nerve injury | 1990-2023 | +29.0% (+11.5 to +45.9) | 38 (20-69) | +17.2% (+1.9 to +33.1) | 35 (15-78) | +23.0% (+10.2 to +35.6) | 73 (34-147) |
|  | Amputation of upper limb, unilateral | 1990-2023 | +29.2% (+12.0 to +46.4) | 2 (1-3) | +16.8% (+1.2 to +34.0) | 1 (0-3) | +23.0% (+9.0 to +36.8) | 3 (1-6) |
|  | Fracture of foot bones except ankle | 1990-2023 | +28.8% (+12.0 to +45.6) | 46 (24-83) | +16.9% (+2.9 to +32.8) | 40 (17-87) | +22.9% (+10.9 to +35.3) | 87 (42-169) |
|  | Fracture of femur, other than femoral neck | 1990-2023 | +29.1% (+11.5 to +46.3) | 74 (39-130) | +17.1% (+1.9 to +32.0) | 74 (34-144) | +22.7% (+9.4 to +35.2) | 148 (73-271) |
|  | Foreign body in GI and urogenital system | 1990-2023 | +29.0% (+12.1 to +46.4) | 22 (10-43) | +17.2% (+2.0 to +33.0) | 23 (8-56) | +22.7% (+10.9 to +36.0) | 45 (18-99) |
|  | Amputation of thumb | 1990-2023 | +29.0% (+12.3 to +45.8) | 15 (6-32) | +17.2% (+1.3 to +33.7) | 16 (5-41) | +22.6% (+8.4 to +35.6) | 32 (12-72) |
|  | Amputation of toe/toes | 1990-2023 | +29.2% (+14.1 to +45.1) | 12 (5-26) | +17.0% (+0.8 to +32.3) | 13 (4-32) | +22.5% (+7.9 to +36.0) | 25 (10-55) |
|  | Fracture of vertebral column | 1990-2023 | +29.0% (+12.4 to +45.8) | 30 (14-57) | +17.0% (+2.1 to +33.5) | 32 (12-66) | +22.5% (+9.2 to +35.8) | 62 (27-125) |
|  | Foreign body in ear | 1990-2023 | +28.8% (+10.5 to +48.7) | 14 (6-28) | +17.1% (+1.7 to +34.0) | 15 (5-36) | +22.3% (+10.8 to +36.9) | 29 (11-61) |
|  | Dislocation of shoulder | 1990-2023 | +29.2% (+11.0 to +45.9) | 16 (7-33) | +17.0% (+2.2 to +32.1) | 18 (7-43) | +22.3% (+9.0 to +35.6) | 34 (15-72) |
|  | Foreign body in respiratory system | 1990-2023 | +29.1% (+12.9 to +46.9) | 17 (8-35) | +17.2% (+1.8 to +33.1) | 20 (7-48) | +22.2% (+8.6 to +36.0) | 38 (15-84) |
|  | Fracture of clavicle, scapula, or humerus | 1990-2023 | +29.1% (+13.1 to +44.6) | 75 (41-129) | +17.0% (+1.9 to +35.0) | 86 (44-157) | +22.2% (+9.6 to +34.2) | 161 (86-285) |
|  | Muscle and tendon injuries, including sprains and strains lesser dislocations | 1990-2023 | +29.1% (+11.4 to +45.2) | 265 (173-384) | +17.1% (+1.4 to +32.5) | 310 (187-475) | +22.2% (+8.3 to +34.7) | 575 (354-855) |
|  | Amputation of lower limb, unilateral | 1990-2023 | +29.0% (+13.6 to +46.7) | 1 (0-1) | +17.0% (+1.5 to +33.6) | 1 (0-1) | +22.1% (+10.8 to +34.9) | 2 (1-3) |
|  | Asphyxiation | 1990-2023 | +29.0% (+11.9 to +47.4) | 11 (4-24) | +17.0% (+1.8 to +33.0) | 13 (4-34) | +22.1% (+7.0 to +35.6) | 24 (9-59) |
|  | Fracture of radius and/or ulna | 1990-2023 | +29.0% (+12.8 to +46.1) | 125 (75-186) | +16.9% (+1.7 to +33.3) | 148 (81-241) | +22.0% (+8.5 to +33.9) | 273 (160-427) |
|  | Dislocation of hip | 1990-2023 | +29.0% (+13.1 to +46.3) | 12 (5-25) | +17.1% (+2.9 to +31.6) | 15 (5-35) | +22.0% (+10.5 to +35.7) | 27 (10-60) |
|  | Lower airway burns | 1990-2023 | +28.7% (+11.8 to +47.1) | 1 (0-2) | +17.0% (+3.5 to +33.5) | 1 (0-3) | +22.0% (+10.0 to +35.7) | 2 (1-5) |
|  | Superficial injury of any part of the body | 1990-2023 | +29.0% (+13.3 to +45.7) | 210 (139-290) | +17.1% (+2.0 to +33.5) | 260 (153-389) | +22.0% (+8.8 to +35.5) | 470 (292-674) |
|  | Dislocation of knee | 1990-2023 | +29.1% (+12.1 to +44.6) | 13 (6-26) | +17.0% (+2.0 to +32.8) | 17 (5-41) | +21.9% (+9.1 to +33.8) | 30 (11-66) |
|  | Amputation of lower limbs, bilateral | 1990-2023 | +28.9% (+11.6 to +45.7) | 1 (0-2) | +16.8% (+1.1 to +33.4) | 1 (0-2) | +21.9% (+9.1 to +34.8) | 2 (1-4) |
|  | Drowning and nonfatal submersion | 1990-2023 | +29.0% (+12.7 to +46.9) | 13 (5-26) | +16.7% (+1.2 to +34.6) | 16 (5-40) | +21.8% (+8.9 to +36.2) | 29 (11-69) |
|  | Contusion in any part of the body | 1990-2023 | +29.1% (+13.4 to +45.1) | 112 (70-170) | +17.1% (+4.0 to +32.5) | 150 (84-258) | +21.7% (+10.4 to +33.8) | 262 (156-419) |
|  | Effect of different environmental factors | 1990-2023 | +29.0% (+11.8 to +45.9) | 22 (9-44) | +17.0% (+3.7 to +33.1) | 29 (11-67) | +21.7% (+9.4 to +34.1) | 51 (21-115) |
|  | Burns, <20% total burned surface area without lower airway burns | 1990-2023 | +28.9% (+11.1 to +47.2) | 114 (63-191) | +17.1% (+1.0 to +35.1) | 153 (69-266) | +21.6% (+7.1 to +35.9) | 266 (132-466) |
|  | Complications following therapeutic procedures | 1990-2023 | +29.1% (+13.0 to +47.1) | 38 (23-60) | +17.0% (+2.4 to +33.0) | 53 (28-91) | +21.6% (+8.8 to +33.7) | 91 (51-157) |
|  | Fracture of pelvis | 1990-2023 | +29.3% (+13.0 to +46.1) | 9 (4-22) | +17.0% (+4.0 to +31.5) | 13 (4-36) | +21.5% (+9.0 to +34.4) | 23 (8-56) |
|  | Fracture of hip | 1990-2023 | +29.1% (+13.4 to +46.1) | 3 (1-6) | +17.1% (+1.4 to +34.5) | 5 (2-11) | +21.1% (+6.9 to +35.1) | 8 (4-17) |
|  | Burns, >=20% total burned surface area or >= 10% burned surface area if head/neck or hands/wrist involved w/o lower airway burns | 1990-2023 | +28.7% (+12.1 to +44.8) | 14 (9-21) | +16.9% (+2.0 to +31.8) | 23 (13-37) | +20.9% (+7.3 to +32.2) | 38 (23-58) |
|  | Poisoning requiring urgent care | 1990-2023 | +29.1% (+13.0 to +44.9) | 120 (71-183) | +17.2% (+2.4 to +32.9) | 214 (117-364) | +20.9% (+7.6 to +33.3) | 334 (190-544) |
| 5-14 years | Severe chest Injury | 2020-2023 |  |  |  |  | +11.2% (+2.1 to +19.6) | 51 (28-93) |
|  | Fracture of skull | 2020-2023 | +11.4% (+1.3 to +22.2) | 18 (9-32) |  |  | +11.1% (+2.8 to +20.9) | 25 (12-46) |
|  | Internal hemorrhage in abdomen and pelvis | 2020-2023 | +11.4% (+0.6 to +21.9) | 62 (33-99) |  |  | +11.1% (+2.7 to +19.1) | 86 (44-139) |
|  | Fracture of face bones | 2020-2023 | +11.4% (+0.1 to +21.3) | 32 (16-52) | +10.2% (+0.0 to +23.0) | 16 (7-27) | +11.0% (+2.4 to +19.8) | 47 (23-79) |
|  | Fracture of femur, other than femoral neck | 2020-2023 | +11.6% (+0.7 to +22.5) | 15 (7-28) |  |  | +11.0% (+2.2 to +19.3) | 27 (12-58) |
|  | Fracture of sternum and/or fracture of one or more ribs | 2020-2023 |  |  |  |  | +11.0% (+2.3 to +19.2) | 4 (2-10) |
|  | Fracture of hand (wrist and other distal part of hand) | 2020-2023 | +11.5% (+0.3 to +23.7) | 20 (10-36) |  |  | +11.0% (+2.7 to +19.6) | 33 (17-59) |
|  | Injury to eyes | 2020-2023 | +11.4% (+1.0 to +23.1) | 26 (14-44) |  |  | +11.0% (+2.4 to +20.2) | 44 (22-79) |
|  | Open wound(s) | 2020-2023 |  |  |  |  | +11.0% (+2.9 to +19.4) | 423 (242-641) |
|  | Spinal cord lesion at neck level | 2020-2023 |  |  |  |  | +10.9% (+3.2 to +19.0) | 1 (0-2) |
|  | Amputation of upper limbs, bilateral | 2020-2023 |  |  |  |  | +10.9% (+2.4 to +19.3) | 1 (0-2) |
|  | Moderate/Severe TBI | 2020-2023 | +11.3% (+0.3 to +22.3) | 23 (14-35) |  |  | +10.9% (+2.8 to +18.7) | 35 (22-54) |
|  | Nerve injury | 2020-2023 | +11.5% (+0.4 to +21.9) | 7 (3-14) |  |  | +10.9% (+2.7 to +19.7) | 12 (5-27) |
|  | Dislocation of shoulder | 2020-2023 | +11.5% (+0.4 to +22.9) | 3 (1-7) |  |  | +10.9% (+2.5 to +19.2) | 6 (2-15) |
|  | Crush injury | 2020-2023 | +11.4% (+0.5 to +23.2) | 1 (1-2) |  |  | +10.9% (+2.8 to +18.6) | 2 (1-3) |
|  | Fracture of foot bones except ankle | 2020-2023 |  |  |  |  | +10.9% (+1.9 to +19.7) | 15 (6-30) |
|  | Amputation of fingers (excluding thumb) | 2020-2023 | +11.4% (+0.1 to +23.4) | 7 (3-14) |  |  | +10.9% (+1.7 to +19.6) | 13 (5-26) |
|  | Effect of different environmental factors | 2020-2023 | +11.5% (+1.0 to +22.9) | 4 (2-9) |  |  | +10.9% (+2.2 to +19.9) | 9 (3-19) |
|  | Amputation of upper limb, unilateral | 2020-2023 |  |  |  |  | +10.9% (+2.9 to +19.1) | 0 (0-1) |
|  | Spinal cord lesion below neck level | 2020-2023 | +11.4% (+0.8 to +22.3) | 1 (0-1) |  |  | +10.9% (+2.2 to +19.5) | 1 (0-2) |
|  | Multiple fractures, dislocations, crashes, wounds, pains, and strains | 2020-2023 | +11.4% (+0.6 to +22.6) | 15 (8-28) |  |  | +10.9% (+2.6 to +19.6) | 27 (13-49) |
|  | Foreign body in ear | 2020-2023 |  |  |  |  | +10.9% (+2.6 to +18.9) | 5 (2-11) |
|  | Foreign body in respiratory system | 2020-2023 | +11.5% (+0.3 to +21.1) | 3 (1-7) |  |  | +10.9% (+2.5 to +20.1) | 7 (3-15) |
|  | Foreign body in GI and urogenital system | 2020-2023 |  |  |  |  | +10.9% (+1.9 to +19.6) | 8 (3-17) |
|  | Fracture of vertebral column | 2020-2023 | +11.4% (+1.3 to +23.7) | 6 (3-12) |  |  | +10.9% (+2.6 to +19.2) | 12 (5-23) |
|  | Amputation of thumb | 2020-2023 |  |  |  |  | +10.9% (+2.5 to +19.7) | 5 (2-13) |
|  | Fracture of patella, tibia or fibula, or ankle | 2020-2023 | +11.3% (+1.2 to +22.6) | 26 (14-43) |  |  | +10.8% (+2.6 to +20.0) | 46 (23-77) |
|  | Minor TBI | 2020-2023 | +11.4% (+0.4 to +22.7) | 17 (8-28) |  |  | +10.8% (+2.4 to +19.3) | 29 (14-53) |
|  | Muscle and tendon injuries, including sprains and strains lesser dislocations | 2020-2023 | +11.4% (+1.2 to +22.5) | 51 (28-85) |  |  | +10.8% (+3.2 to +19.0) | 103 (56-173) |
|  | Amputation of toe/toes | 2020-2023 | +11.3% (+0.9 to +22.6) | 2 (1-5) |  |  | +10.8% (+2.2 to +19.6) | 4 (2-10) |
|  | Dislocation of hip | 2020-2023 |  |  |  |  | +10.8% (+1.5 to +19.9) | 5 (2-11) |
|  | Lower airway burns | 2020-2023 |  |  |  |  | +10.8% (+1.7 to +18.9) | 0 (0-1) |
|  | Asphyxiation | 2020-2023 |  |  |  |  | +10.8% (+2.6 to +20.2) | 4 (1-9) |
|  | Fracture of clavicle, scapula, or humerus | 2020-2023 | +11.4% (+0.3 to +22.7) | 15 (7-28) |  |  | +10.8% (+2.9 to +19.1) | 30 (13-58) |
|  | Drowning and nonfatal submersion | 2020-2023 | +11.4% (+1.3 to +21.5) | 2 (1-5) |  |  | +10.8% (+2.6 to +18.6) | 5 (2-12) |
|  | Amputation of lower limbs, bilateral | 2020-2023 |  |  |  |  | +10.8% (+2.4 to +19.6) | 0 (0-1) |
|  | Dislocation of knee | 2020-2023 | +11.4% (+0.5 to +23.4) | 2 (1-6) |  |  | +10.8% (+2.2 to +20.3) | 5 (2-12) |
|  | Superficial injury of any part of the body | 2020-2023 | +11.4% (+0.1 to +22.7) | 42 (21-68) |  |  | +10.7% (+1.8 to +19.4) | 87 (43-146) |
|  | Contusion in any part of the body | 2020-2023 |  |  |  |  | +10.7% (+1.8 to +20.1) | 47 (24-79) |
|  | Fracture of radius and/or ulna | 2020-2023 | +11.3% (+0.1 to +23.2) | 25 (13-45) |  |  | +10.7% (+2.7 to +19.6) | 51 (24-91) |
|  | Fracture of pelvis | 2020-2023 |  |  |  |  | +10.7% (+1.4 to +20.4) | 4 (1-11) |
|  | Burns, <20% total burned surface area without lower airway burns | 2020-2023 | +11.4% (+0.8 to +22.5) | 20 (9-36) |  |  | +10.7% (+1.5 to +19.2) | 42 (19-82) |
|  | Complications following therapeutic procedures | 2020-2023 | +11.3% (+0.7 to +21.5) | 9 (4-15) | +10.2% (+0.0 to +22.6) | 10 (5-19) | +10.7% (+3.1 to +19.0) | 19 (9-35) |
|  | Amputation of lower limb, unilateral | 2020-2023 |  |  |  |  | +10.6% (+2.2 to +19.2) | 0 (0-1) |
|  | Burns, >=20% total burned surface area or >= 10% burned surface area if head/neck or hands/wrist involved w/o lower airway burns | 2020-2023 |  |  |  |  | +10.6% (+1.3 to +18.8) | 6 (3-10) |
|  | Poisoning requiring urgent care | 2020-2023 | +11.4% (+1.0 to +21.4) | 23 (12-40) |  |  | +10.6% (+2.2 to +19.3) | 59 (29-102) |
|  | Fracture of hip | 2020-2023 |  |  |  |  | +10.5% (+0.3 to +19.8) | 2 (1-5) |

## Croatia

| **Age** | **Category** | **Period** | **Male %Change** | **Male Incidence 2023** | **Female %Change** | **Female Incidence 2023** | **Both %Change** | **Both Incidence 2023** |
| --- | --- | --- | --- | --- | --- | --- | --- | --- |
| 5-14 years | Poisoning requiring urgent care | 2020-2023 |  |  | +16.0% (+1.6 to +31.3) | 18 (8-32) | +13.3% (+1.7 to +24.7) | 29 (14-51) |
|  | Burns, >=20% total burned surface area or >= 10% burned surface area if head/neck or hands/wrist involved w/o lower airway burns | 2020-2023 |  |  | +16.0% (+2.1 to +30.7) | 2 (1-3) | +13.1% (+1.9 to +22.9) | 3 (1-5) |
|  | Fracture of pelvis | 2020-2023 |  |  | +16.2% (+1.2 to +32.3) | 1 (0-3) | +13.0% (+2.9 to +23.3) | 2 (1-5) |
|  | Dislocation of knee | 2020-2023 |  |  | +16.1% (+0.7 to +32.8) | 1 (0-3) | +12.9% (+1.8 to +23.0) | 3 (1-6) |
|  | Burns, <20% total burned surface area without lower airway burns | 2020-2023 |  |  | +15.9% (+1.1 to +31.9) | 11 (5-23) | +12.9% (+1.4 to +22.8) | 21 (9-41) |
|  | Effect of different environmental factors | 2020-2023 |  |  | +15.9% (+0.9 to +31.0) | 2 (1-6) | +12.9% (+0.5 to +22.9) | 4 (2-9) |
|  | Contusion in any part of the body | 2020-2023 |  |  | +16.0% (+0.3 to +32.9) | 12 (6-22) | +12.9% (+0.9 to +23.4) | 23 (12-39) |
|  | Fracture of hip | 2020-2023 |  |  | +15.8% (+1.6 to +31.7) | 1 (0-1) | +12.9% (+0.7 to +23.5) | 1 (0-2) |
|  | Superficial injury of any part of the body | 2020-2023 |  |  | +16.0% (+0.4 to +31.9) | 22 (10-37) | +12.7% (+1.9 to +23.1) | 43 (20-72) |
|  | Drowning and nonfatal submersion | 2020-2023 |  |  | +16.1% (+0.3 to +32.0) | 1 (0-3) | +12.7% (+1.5 to +22.2) | 2 (1-6) |
|  | Complications following therapeutic procedures | 2020-2023 |  |  | +15.8% (+1.4 to +29.7) | 5 (2-10) | +12.7% (+3.0 to +22.0) | 9 (5-17) |
|  | Fracture of radius and/or ulna | 2020-2023 |  |  | +16.0% (+1.1 to +30.4) | 13 (6-23) | +12.7% (+1.2 to +22.0) | 25 (12-46) |
|  | Lower airway burns | 2020-2023 |  |  | +15.9% (+1.1 to +32.7) | 0 (0-0) | +12.7% (+0.9 to +23.1) | 0 (0-0) |
|  | Asphyxiation | 2020-2023 |  |  | +15.9% (+0.3 to +30.7) | 1 (0-3) | +12.7% (+1.0 to +22.4) | 2 (1-5) |
|  | Amputation of thumb | 2020-2023 |  |  | +16.2% (+1.0 to +32.7) | 1 (0-3) | +12.6% (+0.6 to +23.0) | 3 (1-7) |
|  | Muscle and tendon injuries, including sprains and strains lesser dislocations | 2020-2023 |  |  | +15.9% (+1.9 to +28.9) | 25 (13-45) | +12.6% (+1.9 to +22.0) | 51 (27-86) |
|  | Fracture of clavicle, scapula, or humerus | 2020-2023 |  |  | +16.0% (+2.1 to +31.3) | 7 (3-15) | +12.6% (+1.4 to +22.8) | 15 (6-29) |
|  | Dislocation of hip | 2020-2023 |  |  |  |  | +12.6% (+2.2 to +23.6) | 2 (1-6) |
|  | Foreign body in ear | 2020-2023 |  |  | +15.8% (+1.7 to +32.7) | 1 (0-3) | +12.5% (+1.1 to +22.9) | 2 (1-6) |
|  | Foreign body in respiratory system | 2020-2023 |  |  | +15.8% (+0.4 to +31.2) | 2 (1-4) | +12.5% (+2.1 to +22.0) | 3 (1-8) |
|  | Dislocation of shoulder | 2020-2023 |  |  | +16.0% (+1.3 to +30.9) | 2 (0-4) | +12.5% (+1.1 to +22.0) | 3 (1-7) |
|  | Amputation of toe/toes | 2020-2023 |  |  | +16.0% (+0.8 to +31.2) | 1 (0-3) | +12.5% (+0.2 to +22.2) | 2 (1-5) |
|  | Fracture of femur, other than femoral neck | 2020-2023 |  |  | +16.0% (+0.7 to +32.6) | 6 (3-14) | +12.4% (+1.6 to +22.5) | 14 (6-29) |
|  | Fracture of vertebral column | 2020-2023 |  |  | +15.8% (+0.9 to +30.7) | 3 (1-6) | +12.3% (+1.0 to +21.7) | 6 (2-11) |
|  | Foreign body in GI and urogenital system | 2020-2023 |  |  | +15.7% (+0.7 to +31.0) | 2 (1-5) | +12.3% (+1.1 to +22.7) | 4 (1-9) |
|  | Nerve injury | 2020-2023 |  |  | +15.9% (+1.3 to +29.9) | 3 (1-6) | +12.3% (+1.0 to +22.0) | 6 (2-13) |
|  | Minor TBI | 2020-2023 |  |  | +16.1% (+0.5 to +32.8) | 6 (3-11) |  |  |
|  | Fracture of foot bones except ankle | 2020-2023 |  |  | +15.9% (+1.3 to +31.1) | 3 (1-7) | +12.2% (+1.3 to +22.3) | 7 (3-15) |
|  | Amputation of lower limb, unilateral | 2020-2023 |  |  | +15.8% (+1.0 to +32.4) | 0 (0-0) | +12.2% (+1.6 to +22.4) | 0 (0-0) |
|  | Amputation of fingers (excluding thumb) | 2020-2023 |  |  | +16.1% (+1.2 to +32.0) | 3 (1-6) | +12.2% (+0.6 to +22.7) | 6 (2-13) |
|  | Fracture of patella, tibia or fibula, or ankle | 2020-2023 |  |  | +15.9% (+1.6 to +31.5) | 10 (5-18) | +12.1% (+1.0 to +22.3) | 23 (11-39) |
|  | Multiple fractures, dislocations, crashes, wounds, pains, and strains | 2020-2023 |  |  | +15.9% (+1.8 to +32.9) | 6 (2-11) | +12.1% (+1.5 to +21.6) | 13 (6-24) |
|  | Amputation of upper limb, unilateral | 2020-2023 |  |  | +15.8% (+0.9 to +31.6) | 0 (0-0) | +12.1% (+1.4 to +21.9) | 0 (0-1) |
|  | Injury to eyes | 2020-2023 |  |  | +16.0% (+1.6 to +30.9) | 9 (4-18) | +12.1% (+0.5 to +22.1) | 22 (11-39) |
|  | Open wound(s) | 2020-2023 |  |  | +16.0% (+1.1 to +31.1) | 85 (47-134) | +12.0% (+1.6 to +21.9) | 209 (115-330) |
|  | Amputation of upper limbs, bilateral | 2020-2023 |  |  | +16.0% (+2.0 to +30.1) | 0 (0-0) | +12.0% (+0.0 to +22.7) | 0 (0-1) |
|  | Spinal cord lesion at neck level | 2020-2023 |  |  | +16.0% (+2.0 to +30.7) | 0 (0-0) |  |  |
|  | Fracture of hand (wrist and other distal part of hand) | 2020-2023 |  |  | +16.0% (+0.9 to +31.9) | 6 (3-12) |  |  |
|  | Spinal cord lesion below neck level | 2020-2023 |  |  | +15.7% (+1.9 to +30.8) | 0 (0-0) | +11.8% (+1.2 to +22.2) | 0 (0-1) |
|  | Crush injury | 2020-2023 |  |  | +15.9% (+1.6 to +31.0) | 0 (0-1) | +11.7% (+0.7 to +21.4) | 1 (0-2) |
|  | Moderate/Severe TBI | 2020-2023 |  |  | +15.9% (+2.4 to +30.8) | 6 (4-10) | +11.5% (+0.2 to +21.3) | 17 (10-27) |
|  | Fracture of sternum and/or fracture of one or more ribs | 2020-2023 |  |  | +16.0% (+2.5 to +31.3) | 1 (0-2) |  |  |
|  | Fracture of face bones | 2020-2023 |  |  | +15.8% (+1.6 to +30.8) | 8 (4-14) |  |  |
|  | Fracture of skull | 2020-2023 |  |  | +16.0% (+1.9 to +30.7) | 3 (1-7) |  |  |
|  | Internal hemorrhage in abdomen and pelvis | 2020-2023 |  |  | +16.0% (+1.1 to +31.1) | 12 (6-20) |  |  |
|  | Severe chest Injury | 2020-2023 |  |  | +15.9% (+2.4 to +30.6) | 7 (3-13) |  |  |
|  | Burns, <20% total burned surface area without lower airway burns | 2010-2023 |  |  | +15.0% (+1.8 to +29.9) | 11 (5-23) |  |  |
|  | Drowning and nonfatal submersion | 2010-2023 |  |  | +15.2% (+2.2 to +29.9) | 1 (0-3) |  |  |
|  | Dislocation of knee | 2010-2023 |  |  | +14.8% (+0.9 to +30.8) | 1 (0-3) |  |  |
|  | Fracture of pelvis | 2010-2023 |  |  | +14.1% (+0.2 to +29.8) | 1 (0-3) |  |  |
|  | Superficial injury of any part of the body | 2010-2023 |  |  | +14.4% (+0.0 to +30.4) | 22 (10-37) |  |  |
|  | Foreign body in ear | 2010-2023 |  |  | +15.0% (+0.4 to +30.9) | 1 (0-3) |  |  |
|  | Muscle and tendon injuries, including sprains and strains lesser dislocations | 2010-2023 |  |  | +14.5% (+0.5 to +28.5) | 25 (13-45) |  |  |
|  | Amputation of thumb | 2010-2023 |  |  | +14.8% (+1.0 to +30.3) | 1 (0-3) |  |  |
|  | Fracture of radius and/or ulna | 2010-2023 |  |  | +14.2% (+0.4 to +28.1) | 13 (6-23) |  |  |
|  | Lower airway burns | 2010-2023 |  |  | +14.2% (+0.1 to +29.9) | 0 (0-0) |  |  |
|  | Complications following therapeutic procedures | 2010-2023 |  |  | +13.5% (+0.5 to +27.0) | 5 (2-10) |  |  |
|  | Fracture of clavicle, scapula, or humerus | 2010-2023 |  |  | +14.4% (+0.4 to +29.2) | 7 (3-15) |  |  |
|  | Dislocation of shoulder | 2010-2023 |  |  | +14.2% (+0.5 to +28.4) | 2 (0-4) |  |  |
|  | Foreign body in GI and urogenital system | 2010-2023 |  |  | +14.7% (+0.6 to +31.4) | 2 (1-5) |  |  |
|  | Nerve injury | 2010-2023 |  |  | +14.7% (+1.7 to +30.0) | 3 (1-6) |  |  |
|  | Fracture of femur, other than femoral neck | 2010-2023 |  |  | +14.3% (+0.5 to +29.5) | 6 (3-14) |  |  |
|  | Fracture of vertebral column | 2010-2023 |  |  | +14.3% (+1.1 to +29.2) | 3 (1-6) |  |  |
|  | Fracture of foot bones except ankle | 2010-2023 |  |  | +14.8% (+0.2 to +30.7) | 3 (1-7) |  |  |
|  | Amputation of fingers (excluding thumb) | 2010-2023 |  |  | +14.7% (+0.4 to +30.6) | 3 (1-6) |  |  |
|  | Injury to eyes | 2010-2023 |  |  | +14.6% (+1.3 to +30.1) | 9 (4-18) |  |  |
|  | Multiple fractures, dislocations, crashes, wounds, pains, and strains | 2010-2023 |  |  | +14.0% (+0.5 to +29.3) | 6 (2-11) |  |  |
|  | Open wound(s) | 2010-2023 |  |  | +14.4% (+1.0 to +30.1) | 85 (47-134) |  |  |
|  | Fracture of face bones | 2010-2023 |  |  | +14.5% (+0.2 to +30.3) | 8 (4-14) |  |  |
|  | Fracture of sternum and/or fracture of one or more ribs | 2010-2023 |  |  | +14.3% (+0.5 to +28.0) | 1 (0-2) |  |  |
|  | Moderate/Severe TBI | 2010-2023 |  |  | +13.3% (+0.2 to +28.3) | 6 (4-10) |  |  |
|  | Internal hemorrhage in abdomen and pelvis | 2010-2023 |  |  | +14.6% (+2.2 to +30.0) | 12 (6-20) |  |  |
|  | Fracture of skull | 2010-2023 |  |  | +14.6% (+1.8 to +29.3) | 3 (1-7) |  |  |
|  | Severe chest Injury | 2010-2023 |  |  | +14.5% (+0.7 to +29.5) | 7 (3-13) |  |  |

## Cyprus

| **Age** | **Category** | **Period** | **Male %Change** | **Male Incidence 2023** | **Female %Change** | **Female Incidence 2023** | **Both %Change** | **Both Incidence 2023** |
| --- | --- | --- | --- | --- | --- | --- | --- | --- |
| 5-14 years | Amputation of toe/toes | 2020-2023 |  |  |  |  | +10.9% (+1.3 to +19.6) | 1 (0-2) |
|  | Asphyxiation | 2020-2023 |  |  |  |  | +10.8% (+1.5 to +18.4) | 2 (0-5) |
|  | Dislocation of knee | 2020-2023 |  |  |  |  | +10.8% (+1.1 to +18.4) | 2 (0-4) |
|  | Nerve injury | 2020-2023 |  |  |  |  | +10.8% (+1.9 to +18.3) | 3 (1-7) |
|  | Fracture of skull | 2020-2023 |  |  |  |  | +10.7% (+1.5 to +19.1) | 11 (5-20) |
|  | Severe chest Injury | 2020-2023 |  |  |  |  | +10.7% (+1.7 to +19.5) | 7 (3-12) |
|  | Amputation of thumb | 2020-2023 |  |  |  |  | +10.7% (+1.3 to +19.5) | 1 (0-3) |
|  | Foreign body in ear | 2020-2023 |  |  |  |  | +10.7% (+1.4 to +19.1) | 2 (1-4) |
|  | Fracture of foot bones except ankle | 2020-2023 |  |  |  |  | +10.7% (+1.3 to +19.1) | 3 (1-6) |
|  | Internal hemorrhage in abdomen and pelvis | 2020-2023 |  |  |  |  | +10.7% (+2.3 to +19.6) | 9 (4-15) |
|  | Fracture of sternum and/or fracture of one or more ribs | 2020-2023 |  |  |  |  | +10.7% (+1.1 to +20.2) | 3 (1-7) |
|  | Burns, <20% total burned surface area without lower airway burns | 2020-2023 |  |  |  |  | +10.7% (+1.3 to +18.0) | 5 (2-10) |
|  | Dislocation of shoulder | 2020-2023 |  |  |  |  | +10.7% (+1.5 to +18.9) | 2 (1-6) |
|  | Fracture of hand (wrist and other distal part of hand) | 2020-2023 |  |  |  |  | +10.7% (+1.8 to +18.6) | 13 (6-25) |
|  | Lower airway burns | 2020-2023 |  |  |  |  | +10.7% (+1.6 to +19.9) | 0 (0-0) |
|  | Fracture of face bones | 2020-2023 |  |  |  |  | +10.7% (+2.2 to +18.8) | 16 (8-27) |
|  | Amputation of fingers (excluding thumb) | 2020-2023 |  |  |  |  | +10.6% (+1.8 to +19.6) | 1 (0-3) |
|  | Dislocation of hip | 2020-2023 |  |  |  |  | +10.6% (+1.0 to +19.6) | 1 (0-3) |
|  | Spinal cord lesion below neck level | 2020-2023 |  |  |  |  | +10.6% (+1.8 to +18.3) | 0 (0-0) |
|  | Open wound(s) | 2020-2023 |  |  |  |  | +10.6% (+2.2 to +18.3) | 64 (36-102) |
|  | Effect of different environmental factors | 2020-2023 |  |  |  |  | +10.6% (+0.8 to +19.9) | 2 (1-4) |
|  | Foreign body in GI and urogenital system | 2020-2023 |  |  |  |  | +10.6% (+1.2 to +20.0) | 1 (0-3) |
|  | Drowning and nonfatal submersion | 2020-2023 |  |  |  |  | +10.6% (+2.1 to +18.8) | 2 (0-4) |
|  | Fracture of patella, tibia or fibula, or ankle | 2020-2023 |  |  |  |  | +10.6% (+1.8 to +18.8) | 6 (3-10) |
|  | Superficial injury of any part of the body | 2020-2023 |  |  |  |  | +10.6% (+0.5 to +18.3) | 44 (21-72) |
|  | Injury to eyes | 2020-2023 |  |  |  |  | +10.6% (+2.1 to +18.5) | 12 (6-22) |
|  | Foreign body in respiratory system | 2020-2023 |  |  |  |  | +10.5% (+2.3 to +17.8) | 1 (0-2) |
|  | Muscle and tendon injuries, including sprains and strains lesser dislocations | 2020-2023 |  |  |  |  | +10.5% (+1.6 to +18.7) | 11 (6-20) |
|  | Fracture of femur, other than femoral neck | 2020-2023 |  |  |  |  | +10.5% (+1.5 to +18.6) | 2 (1-4) |
|  | Fracture of pelvis | 2020-2023 |  |  |  |  | +10.5% (+1.2 to +19.3) | 2 (0-4) |
|  | Spinal cord lesion at neck level | 2020-2023 |  |  |  |  | +10.5% (+1.9 to +18.5) | 0 (0-0) |
|  | Amputation of upper limbs, bilateral | 2020-2023 |  |  |  |  | +10.5% (+1.4 to +18.9) | 0 (0-0) |
|  | Fracture of radius and/or ulna | 2020-2023 |  |  |  |  | +10.5% (+1.5 to +17.8) | 5 (2-9) |
|  | Fracture of clavicle, scapula, or humerus | 2020-2023 |  |  |  |  | +10.5% (+1.4 to +19.3) | 6 (3-12) |
|  | Poisoning requiring urgent care | 2020-2023 |  |  |  |  | +10.5% (+0.4 to +18.4) | 3 (2-6) |
|  | Multiple fractures, dislocations, crashes, wounds, pains, and strains | 2020-2023 |  |  |  |  | +10.5% (+2.1 to +18.9) | 2 (1-4) |
|  | Amputation of lower limbs, bilateral | 2020-2023 |  |  |  |  | +10.5% (+0.9 to +18.0) | 0 (0-0) |
|  | Contusion in any part of the body | 2020-2023 |  |  |  |  | +10.5% (+1.2 to +19.4) | 35 (19-62) |
|  | Minor TBI | 2020-2023 |  |  |  |  | +10.5% (+1.8 to +19.2) | 9 (5-17) |
|  | Amputation of upper limb, unilateral | 2020-2023 |  |  |  |  | +10.5% (+1.5 to +19.4) | 0 (0-0) |
|  | Burns, >=20% total burned surface area or >= 10% burned surface area if head/neck or hands/wrist involved w/o lower airway burns | 2020-2023 |  |  |  |  | +10.5% (+0.7 to +19.1) | 0 (0-0) |
|  | Moderate/Severe TBI | 2020-2023 |  |  |  |  | +10.5% (+1.6 to +18.3) | 2 (1-4) |
|  | Crush injury | 2020-2023 |  |  |  |  | +10.5% (+1.7 to +18.5) | 0 (0-0) |
|  | Fracture of vertebral column | 2020-2023 |  |  |  |  | +10.4% (+1.6 to +17.8) | 2 (1-4) |
|  | Complications following therapeutic procedures | 2020-2023 |  |  |  |  | +10.3% (+1.8 to +17.9) | 1 (0-1) |
|  | Amputation of lower limb, unilateral | 2020-2023 |  |  |  |  | +10.2% (+0.4 to +18.8) | 0 (0-0) |
|  | Fracture of hip | 2020-2023 |  |  |  |  | +10.2% (+0.1 to +18.5) | 0 (0-1) |

## Czechia

| **Age** | **Category** | **Period** | **Male %Change** | **Male Incidence 2023** | **Female %Change** | **Female Incidence 2023** | **Both %Change** | **Both Incidence 2023** |
| --- | --- | --- | --- | --- | --- | --- | --- | --- |
| 5-14 years | Internal hemorrhage in abdomen and pelvis | 2020-2023 |  |  |  |  | +10.1% (+0.2 to +21.7) | 200 (105-330) |
|  | Fracture of hand (wrist and other distal part of hand) | 2020-2023 |  |  |  |  | +10.0% (+0.0 to +20.3) | 78 (37-138) |
|  | Crush injury | 2020-2023 |  |  |  |  | +10.0% (+0.3 to +20.3) | 4 (2-7) |
|  | Asphyxiation | 2020-2023 |  |  |  |  | +10.0% (+0.8 to +21.2) | 9 (3-22) |
|  | Moderate/Severe TBI | 2020-2023 |  |  |  |  | +9.9% (+0.2 to +20.7) | 82 (47-125) |
|  | Nerve injury | 2020-2023 |  |  |  |  | +9.9% (+0.7 to +20.6) | 29 (11-63) |
|  | Fracture of femur, other than femoral neck | 2020-2023 |  |  |  |  | +9.9% (+0.7 to +21.1) | 63 (28-138) |
| 15-19 years | Minor TBI | 2020-2023 |  |  |  |  | +10.1% (+0.2 to +23.0) | 26 (12-46) |
|  | Multiple fractures, dislocations, crashes, wounds, pains, and strains | 2020-2023 |  |  |  |  | +10.1% (+0.2 to +22.0) | 24 (11-47) |
|  | Fracture of vertebral column | 2020-2023 |  |  |  |  | +9.9% (+0.6 to +21.0) | 10 (4-21) |
|  | Fracture of clavicle, scapula, or humerus | 2020-2023 |  |  |  |  | +9.9% (+0.0 to +22.0) | 25 (11-47) |
|  | Drowning and nonfatal submersion | 2020-2023 |  |  |  |  | +9.8% (+0.8 to +20.0) | 4 (1-8) |
|  | Contusion in any part of the body | 2020-2023 |  |  |  |  | +9.7% (+0.1 to +20.5) | 37 (18-63) |
|  | Effect of different environmental factors | 2020-2023 |  |  |  |  | +9.6% (+0.6 to +19.9) | 7 (2-15) |
|  | Burns, >=20% total burned surface area or >= 10% burned surface area if head/neck or hands/wrist involved w/o lower airway burns | 2020-2023 |  |  |  |  | +9.6% (+0.4 to +20.2) | 6 (3-11) |
|  | Fracture of hip | 2020-2023 |  |  |  |  | +9.5% (+0.2 to +21.5) | 3 (1-6) |
|  | Poisoning requiring urgent care | 2020-2023 |  |  |  |  | +9.5% (+1.6 to +21.6) | 48 (23-89) |

## Denmark

| **Age** | **Category** | **Period** | **Male %Change** | **Male Incidence 2023** | **Female %Change** | **Female Incidence 2023** | **Both %Change** | **Both Incidence 2023** |
| --- | --- | --- | --- | --- | --- | --- | --- | --- |
| 5-14 years | Fracture of skull | 2020-2023 | +12.3% (+0.6 to +26.9) | 37 (18-64) |  |  | +11.4% (+1.1 to +23.0) | 52 (24-92) |
|  | Internal hemorrhage in abdomen and pelvis | 2020-2023 | +12.3% (+0.8 to +25.5) | 28 (14-48) |  |  | +11.4% (+2.6 to +23.0) | 40 (20-68) |
|  | Severe chest Injury | 2020-2023 | +12.2% (+1.0 to +28.5) | 22 (11-39) |  |  | +11.4% (+2.1 to +24.0) | 30 (15-54) |
|  | Fracture of face bones | 2020-2023 | +12.2% (+1.2 to +27.9) | 50 (24-83) |  |  | +11.2% (+1.4 to +22.8) | 74 (35-125) |
|  | Fracture of hand (wrist and other distal part of hand) | 2020-2023 |  |  |  |  | +11.1% (+1.9 to +20.7) | 63 (29-118) |
|  | Fracture of sternum and/or fracture of one or more ribs | 2020-2023 | +12.1% (+0.7 to +27.2) | 10 (4-21) |  |  | +11.0% (+1.6 to +21.3) | 16 (6-34) |
|  | Spinal cord lesion below neck level | 2020-2023 | +12.4% (+0.2 to +27.6) | 1 (0-1) |  |  | +11.0% (+1.1 to +21.7) | 1 (0-2) |
|  | Moderate/Severe TBI | 2020-2023 | +12.2% (+0.7 to +27.3) | 7 (3-11) |  |  | +11.0% (+1.3 to +21.6) | 10 (6-18) |
|  | Fracture of foot bones except ankle | 2020-2023 | +12.4% (+0.7 to +27.6) | 7 (3-14) |  |  | +11.0% (+1.6 to +19.9) | 12 (5-26) |
|  | Injury to eyes | 2020-2023 | +12.3% (+1.1 to +27.9) | 33 (16-59) |  |  | +11.0% (+1.7 to +22.4) | 57 (27-103) |
|  | Open wound(s) | 2020-2023 | +12.2% (+0.4 to +26.4) | 175 (94-283) |  |  | +10.9% (+1.6 to +21.7) | 298 (159-487) |
|  | Amputation of fingers (excluding thumb) | 2020-2023 | +12.3% (+0.2 to +26.8) | 3 (1-7) |  |  | +10.9% (+1.7 to +22.7) | 6 (2-13) |
|  | Amputation of upper limbs, bilateral | 2020-2023 | +12.2% (+1.4 to +27.3) | 0 (0-0) |  |  | +10.9% (+0.8 to +20.8) | 0 (0-1) |
|  | Crush injury | 2020-2023 | +12.1% (+0.4 to +26.1) | 0 (0-0) |  |  | +10.9% (+1.8 to +21.2) | 0 (0-1) |
|  | Foreign body in ear | 2020-2023 | +12.5% (+0.7 to +26.3) | 4 (1-9) |  |  | +10.8% (+0.9 to +23.6) | 8 (2-19) |
|  | Minor TBI | 2020-2023 | +12.3% (+1.2 to +29.1) | 25 (12-43) |  |  | +10.8% (+2.0 to +21.0) | 44 (21-79) |
|  | Nerve injury | 2020-2023 | +12.2% (+0.4 to +26.8) | 8 (3-17) |  |  | +10.8% (+1.0 to +20.5) | 15 (6-34) |
|  | Amputation of thumb | 2020-2023 |  |  |  |  | +10.8% (+1.2 to +23.0) | 6 (2-16) |
|  | Multiple fractures, dislocations, crashes, wounds, pains, and strains | 2020-2023 | +12.2% (+0.1 to +26.1) | 5 (2-10) |  |  | +10.8% (+1.4 to +21.5) | 9 (4-18) |
|  | Spinal cord lesion at neck level | 2020-2023 | +12.2% (+0.3 to +26.3) | 0 (0-1) |  |  | +10.8% (+0.5 to +21.2) | 1 (0-1) |
|  | Fracture of patella, tibia or fibula, or ankle | 2020-2023 | +12.3% (+0.1 to +25.9) | 15 (7-26) |  |  | +10.8% (+1.1 to +21.1) | 26 (12-46) |
|  | Foreign body in respiratory system | 2020-2023 |  |  |  |  | +10.7% (+1.7 to +19.4) | 5 (2-11) |
|  | Fracture of femur, other than femoral neck | 2020-2023 | +12.2% (+0.7 to +27.8) | 5 (2-10) |  |  | +10.7% (+0.5 to +21.1) | 9 (4-20) |
|  | Fracture of vertebral column | 2020-2023 | +12.3% (+0.3 to +28.3) | 5 (2-11) |  |  | +10.7% (+0.9 to +21.7) | 10 (4-21) |
|  | Fracture of clavicle, scapula, or humerus | 2020-2023 | +12.3% (+0.3 to +26.4) | 15 (7-28) |  |  | +10.7% (+1.2 to +21.4) | 30 (13-59) |
|  | Amputation of lower limbs, bilateral | 2020-2023 |  |  |  |  | +10.7% (+0.7 to +21.2) | 0 (0-1) |
|  | Asphyxiation | 2020-2023 | +12.3% (+0.2 to +29.6) | 4 (1-10) |  |  | +10.7% (+1.2 to +23.1) | 8 (2-23) |
|  | Foreign body in GI and urogenital system | 2020-2023 | +12.1% (+0.4 to +28.0) | 3 (1-8) |  |  | +10.6% (+0.8 to +23.3) | 7 (2-15) |
|  | Muscle and tendon injuries, including sprains and strains lesser dislocations | 2020-2023 | +12.3% (+0.9 to +25.1) | 26 (13-45) |  |  | +10.6% (+1.2 to +20.3) | 52 (27-92) |
|  | Fracture of radius and/or ulna | 2020-2023 | +12.2% (+0.2 to +29.0) | 11 (5-20) |  |  | +10.6% (+0.9 to +21.6) | 22 (9-41) |
|  | Drowning and nonfatal submersion | 2020-2023 | +12.2% (+1.2 to +27.5) | 4 (1-9) |  |  | +10.6% (+1.6 to +21.4) | 8 (2-21) |
|  | Effect of different environmental factors | 2020-2023 |  |  |  |  | +10.6% (+0.2 to +19.3) | 9 (3-21) |
|  | Amputation of toe/toes | 2020-2023 |  |  |  |  | +10.6% (+1.9 to +19.8) | 5 (1-11) |
|  | Dislocation of shoulder | 2020-2023 | +12.1% (+0.1 to +26.7) | 5 (2-13) |  |  | +10.6% (+0.7 to +21.8) | 10 (3-26) |
|  | Contusion in any part of the body | 2020-2023 | +12.2% (+0.4 to +26.3) | 75 (41-138) |  |  | +10.5% (+1.5 to +20.7) | 166 (87-302) |
|  | Amputation of upper limb, unilateral | 2020-2023 |  |  |  |  | +10.5% (+1.2 to +24.8) | 0 (0-1) |
|  | Dislocation of hip | 2020-2023 | +12.2% (+0.5 to +25.8) | 2 (1-6) |  |  | +10.5% (+0.9 to +22.5) | 5 (2-12) |
|  | Superficial injury of any part of the body | 2020-2023 | +12.2% (+0.1 to +28.8) | 99 (49-161) |  |  | +10.5% (+0.3 to +22.5) | 205 (97-341) |
|  | Burns, <20% total burned surface area without lower airway burns | 2020-2023 |  |  |  |  | +10.5% (+0.4 to +24.6) | 25 (10-48) |
|  | Dislocation of knee | 2020-2023 | +12.3% (+0.3 to +27.4) | 4 (1-8) |  |  | +10.5% (+1.0 to +21.1) | 8 (2-19) |
|  | Lower airway burns | 2020-2023 | +12.0% (+0.1 to +28.1) | 0 (0-0) |  |  | +10.4% (+1.3 to +21.7) | 0 (0-1) |
|  | Complications following therapeutic procedures | 2020-2023 | +12.2% (+1.7 to +25.9) | 1 (1-2) |  |  | +10.4% (+1.2 to +20.3) | 3 (1-5) |
|  | Fracture of pelvis | 2020-2023 | +12.1% (+1.0 to +28.7) | 3 (1-9) |  |  | +10.3% (+0.1 to +22.8) | 7 (2-20) |
|  | Burns, >=20% total burned surface area or >= 10% burned surface area if head/neck or hands/wrist involved w/o lower airway burns | 2020-2023 | +12.1% (+1.5 to +25.6) | 0 (0-1) |  |  | +10.3% (+0.7 to +20.0) | 1 (0-2) |
|  | Poisoning requiring urgent care | 2020-2023 | +12.2% (+0.9 to +27.1) | 6 (3-11) |  |  | +10.2% (+0.5 to +20.0) | 16 (8-31) |
|  | Amputation of lower limb, unilateral | 2020-2023 |  |  |  |  | +10.1% (+1.0 to +21.0) | 0 (0-0) |
|  | Fracture of hip | 2020-2023 |  |  |  |  | +9.9% (+0.2 to +20.6) | 2 (1-3) |

## Estonia

| **Age** | **Category** | **Period** | **Male %Change** | **Male Incidence 2023** | **Female %Change** | **Female Incidence 2023** | **Both %Change** | **Both Incidence 2023** |
| --- | --- | --- | --- | --- | --- | --- | --- | --- |
| 5-14 years | Severe chest Injury | 2020-2023 | +13.7% (+1.2 to +29.4) | 7 (4-12) | +12.2% (+0.1 to +25.8) | 3 (2-7) | +13.2% (+4.1 to +24.3) | 10 (5-19) |
|  | Moderate/Severe TBI | 2020-2023 | +13.7% (+1.0 to +29.3) | 4 (2-6) | +12.5% (+0.5 to +26.2) | 3 (2-5) | +13.2% (+4.7 to +22.6) | 7 (4-11) |
|  | Internal hemorrhage in abdomen and pelvis | 2020-2023 | +13.6% (+1.9 to +29.0) | 11 (6-18) |  |  | +13.1% (+4.5 to +23.6) | 17 (9-28) |
|  | Fracture of skull | 2020-2023 | +13.6% (+1.0 to +31.6) | 3 (2-6) | +12.3% (+1.1 to +25.4) | 2 (1-4) | +13.1% (+3.7 to +24.9) | 5 (2-9) |
|  | Amputation of lower limb, unilateral | 2020-2023 | +13.7% (+0.5 to +30.6) | 0 (0-0) | +12.7% (+0.7 to +28.1) | 0 (0-0) | +13.1% (+4.6 to +23.8) | 0 (0-0) |
|  | Fracture of sternum and/or fracture of one or more ribs | 2020-2023 | +13.6% (+1.6 to +27.7) | 1 (0-1) | +12.4% (+0.4 to +25.8) | 0 (0-1) | +13.1% (+4.8 to +24.0) | 1 (0-2) |
|  | Crush injury | 2020-2023 | +13.7% (+0.0 to +29.9) | 0 (0-0) |  |  | +13.1% (+4.1 to +23.6) | 0 (0-1) |
|  | Spinal cord lesion at neck level | 2020-2023 | +13.6% (+1.4 to +29.3) | 0 (0-0) | +12.6% (+0.0 to +26.4) | 0 (0-0) | +13.1% (+4.6 to +23.0) | 0 (0-0) |
|  | Fracture of face bones | 2020-2023 |  |  | +12.3% (+0.1 to +26.7) | 4 (2-7) | +13.1% (+3.7 to +24.1) | 10 (5-16) |
|  | Multiple fractures, dislocations, crashes, wounds, pains, and strains | 2020-2023 | +13.8% (+2.0 to +29.9) | 3 (1-5) |  |  | +13.0% (+4.8 to +22.6) | 6 (3-10) |
|  | Amputation of fingers (excluding thumb) | 2020-2023 | +13.7% (+0.5 to +31.1) | 1 (1-3) |  |  | +13.0% (+3.6 to +23.3) | 3 (1-5) |
|  | Open wound(s) | 2020-2023 | +13.7% (+1.3 to +29.1) | 45 (26-71) | +12.3% (+0.2 to +25.7) | 43 (24-69) | +13.0% (+4.8 to +22.4) | 88 (50-138) |
|  | Fracture of hip | 2020-2023 | +13.8% (+1.0 to +32.0) | 0 (0-0) |  |  | +13.0% (+3.2 to +23.6) | 0 (0-1) |
|  | Fracture of hand (wrist and other distal part of hand) | 2020-2023 | +13.6% (+1.9 to +30.3) | 4 (2-7) | +12.3% (+0.9 to +25.7) | 3 (1-6) | +13.0% (+4.2 to +23.2) | 7 (3-13) |
|  | Injury to eyes | 2020-2023 | +13.6% (+1.0 to +29.7) | 5 (2-8) |  |  | +13.0% (+4.0 to +23.6) | 9 (4-17) |
|  | Minor TBI | 2020-2023 | +13.6% (+0.3 to +29.9) | 3 (2-5) |  |  | +13.0% (+3.7 to +22.8) | 6 (3-11) |
|  | Fracture of patella, tibia or fibula, or ankle | 2020-2023 | +13.7% (+0.3 to +28.4) | 5 (2-8) |  |  | +13.0% (+4.3 to +21.7) | 10 (5-17) |
|  | Amputation of upper limbs, bilateral | 2020-2023 |  |  |  |  | +13.0% (+3.1 to +23.6) | 0 (0-0) |
|  | Amputation of toe/toes | 2020-2023 | +13.9% (+1.2 to +30.1) | 0 (0-1) |  |  | +12.9% (+4.3 to +22.1) | 1 (0-2) |
|  | Spinal cord lesion below neck level | 2020-2023 | +13.7% (+1.4 to +28.8) | 0 (0-0) |  |  | +12.9% (+4.3 to +21.5) | 0 (0-0) |
|  | Fracture of radius and/or ulna | 2020-2023 | +13.6% (+1.1 to +29.9) | 5 (2-8) | +12.4% (+0.1 to +25.5) | 6 (3-12) | +12.9% (+3.6 to +22.7) | 11 (5-20) |
|  | Fracture of vertebral column | 2020-2023 | +13.7% (+1.3 to +28.8) | 1 (0-2) |  |  | +12.9% (+4.0 to +22.0) | 2 (1-5) |
|  | Fracture of pelvis | 2020-2023 | +13.6% (+1.5 to +29.7) | 0 (0-1) |  |  | +12.9% (+3.8 to +22.5) | 1 (0-2) |
|  | Fracture of femur, other than femoral neck | 2020-2023 | +13.6% (+0.6 to +30.3) | 3 (1-5) |  |  | +12.9% (+3.5 to +22.9) | 6 (3-12) |
|  | Lower airway burns | 2020-2023 | +13.6% (+2.0 to +31.4) | 0 (0-0) |  |  | +12.9% (+3.8 to +24.0) | 0 (0-0) |
|  | Dislocation of shoulder | 2020-2023 | +13.6% (+1.3 to +30.7) | 1 (0-1) |  |  | +12.9% (+3.7 to +22.1) | 1 (0-3) |
|  | Muscle and tendon injuries, including sprains and strains lesser dislocations | 2020-2023 | +13.7% (+1.4 to +29.8) | 9 (5-15) |  |  | +12.9% (+4.0 to +22.0) | 22 (12-38) |
|  | Foreign body in ear | 2020-2023 | +13.6% (+1.5 to +30.5) | 0 (0-1) |  |  | +12.9% (+4.1 to +23.3) | 1 (0-2) |
|  | Fracture of clavicle, scapula, or humerus | 2020-2023 | +13.6% (+0.6 to +30.6) | 3 (1-5) | +12.3% (+0.5 to +27.4) | 4 (2-8) | +12.9% (+3.9 to +22.1) | 6 (3-13) |
|  | Foreign body in respiratory system | 2020-2023 |  |  |  |  | +12.8% (+4.1 to +22.3) | 1 (1-3) |
|  | Amputation of upper limb, unilateral | 2020-2023 | +13.4% (+0.4 to +29.3) | 0 (0-0) |  |  | +12.8% (+3.8 to +23.7) | 0 (0-0) |
|  | Amputation of thumb | 2020-2023 | +13.5% (+0.0 to +30.6) | 1 (0-1) |  |  | +12.8% (+4.2 to +23.2) | 1 (0-3) |
|  | Nerve injury | 2020-2023 | +13.6% (+1.6 to +29.6) | 1 (1-3) | +12.1% (+0.1 to +25.2) | 1 (0-3) | +12.8% (+4.2 to +22.4) | 3 (1-6) |
|  | Dislocation of knee | 2020-2023 | +13.8% (+0.9 to +30.0) | 0 (0-1) |  |  | +12.8% (+4.1 to +22.0) | 1 (0-3) |
|  | Superficial injury of any part of the body | 2020-2023 | +13.6% (+0.3 to +29.8) | 8 (4-12) |  |  | +12.8% (+3.8 to +22.0) | 19 (9-32) |
|  | Foreign body in GI and urogenital system | 2020-2023 |  |  |  |  | +12.8% (+3.4 to +23.3) | 2 (1-4) |
|  | Dislocation of hip | 2020-2023 | +13.6% (+0.7 to +29.9) | 0 (0-1) |  |  | +12.8% (+3.9 to +23.5) | 1 (0-2) |
|  | Amputation of lower limbs, bilateral | 2020-2023 | +13.6% (+1.6 to +29.1) | 0 (0-0) | +12.2% (+0.4 to +26.0) | 0 (0-0) | +12.8% (+5.0 to +22.4) | 0 (0-0) |
|  | Contusion in any part of the body | 2020-2023 | +13.6% (+1.6 to +30.6) | 4 (2-6) |  |  | +12.8% (+3.1 to +22.2) | 10 (5-18) |
|  | Complications following therapeutic procedures | 2020-2023 | +13.6% (+1.4 to +29.6) | 2 (1-3) | +12.3% (+0.5 to +26.4) | 3 (1-5) | +12.8% (+4.4 to +22.3) | 4 (2-8) |
|  | Burns, >=20% total burned surface area or >= 10% burned surface area if head/neck or hands/wrist involved w/o lower airway burns | 2020-2023 | +13.7% (+2.0 to +29.7) | 0 (0-1) |  |  | +12.8% (+3.2 to +22.6) | 1 (1-2) |
|  | Poisoning requiring urgent care | 2020-2023 | +13.7% (+0.3 to +29.8) | 4 (2-7) |  |  | +12.8% (+3.7 to +23.3) | 13 (6-23) |
|  | Fracture of foot bones except ankle | 2020-2023 | +13.4% (+0.4 to +30.4) | 2 (1-3) |  |  | +12.8% (+3.6 to +22.8) | 3 (1-6) |
|  | Asphyxiation | 2020-2023 | +13.6% (+0.7 to +29.2) | 0 (0-1) |  |  | +12.7% (+4.2 to +23.2) | 1 (0-2) |
|  | Effect of different environmental factors | 2020-2023 | +13.5% (+0.7 to +30.3) | 1 (0-2) |  |  | +12.7% (+3.6 to +22.3) | 2 (1-4) |
|  | Drowning and nonfatal submersion | 2020-2023 | +13.5% (+1.2 to +29.8) | 0 (0-1) |  |  | +12.7% (+3.9 to +22.4) | 1 (0-3) |
|  | Burns, <20% total burned surface area without lower airway burns | 2020-2023 | +13.7% (+0.3 to +30.5) | 4 (2-6) |  |  | +12.7% (+3.6 to +23.3) | 9 (4-18) |

## European Union

| **Age** | **Category** | **Period** | **Male %Change** | **Male Incidence 2023** | **Female %Change** | **Female Incidence 2023** | **Both %Change** | **Both Incidence 2023** |
| --- | --- | --- | --- | --- | --- | --- | --- | --- |
| 5-14 years | Crush injury | 2020-2023 | +13.4% (+9.6 to +16.4) | 27 (13-49) | +12.2% (+8.6 to +15.7) | 17 (7-36) | +13.0% (+9.7 to +15.4) | 44 (21-83) |
|  | Amputation of lower limb, unilateral | 2020-2023 | +13.1% (+8.7 to +16.2) | 4 (2-7) | +12.8% (+9.2 to +16.3) | 4 (2-8) | +12.9% (+9.9 to +15.8) | 8 (3-15) |
|  | Internal hemorrhage in abdomen and pelvis | 2020-2023 | +13.6% (+10.0 to +17.3) | 2,559 (1,364-4,083) | +11.4% (+8.0 to +15.5) | 1,079 (511-1,765) | +12.9% (+9.7 to +16.1) | 3,638 (1,871-5,827) |
|  | Severe chest Injury | 2020-2023 | +13.6% (+9.8 to +17.1) | 1,803 (930-3,126) | +11.1% (+7.3 to +15.8) | 715 (341-1,373) | +12.9% (+9.6 to +16.0) | 2,518 (1,286-4,509) |
|  | Burns, >=20% total burned surface area or >= 10% burned surface area if head/neck or hands/wrist involved w/o lower airway burns | 2020-2023 | +13.4% (+9.4 to +16.2) | 62 (30-105) | +12.5% (+8.8 to +15.9) | 90 (40-165) | +12.9% (+9.9 to +15.3) | 152 (71-271) |
|  | Complications following therapeutic procedures | 2020-2023 | +13.4% (+9.7 to +16.0) | 213 (105-377) | +12.4% (+9.1 to +15.7) | 264 (126-499) | +12.8% (+10.0 to +15.1) | 477 (234-897) |
|  | Moderate/Severe TBI | 2020-2023 | +13.4% (+9.7 to +16.5) | 739 (441-1,165) | +11.7% (+8.2 to +15.8) | 443 (257-709) | +12.8% (+9.7 to +15.5) | 1,183 (703-1,886) |
|  | Fracture of femur, other than femoral neck | 2020-2023 | +13.5% (+10.0 to +16.9) | 502 (238-1,033) | +11.8% (+8.1 to +15.8) | 456 (188-1,024) | +12.7% (+9.5 to +15.4) | 958 (437-2,029) |
|  | Fracture of skull | 2020-2023 | +13.6% (+9.7 to +17.5) | 2,278 (1,070-3,939) | +10.4% (+5.3 to +15.5) | 920 (370-1,809) | +12.7% (+9.1 to +16.4) | 3,198 (1,448-5,667) |
|  | Amputation of upper limbs, bilateral | 2020-2023 | +13.5% (+9.5 to +17.4) | 16 (6-35) | +11.4% (+6.6 to +15.7) | 11 (3-27) | +12.7% (+9.2 to +15.4) | 27 (9-61) |
|  | Multiple fractures, dislocations, crashes, wounds, pains, and strains | 2020-2023 | +13.5% (+9.8 to +16.6) | 548 (261-979) | +11.6% (+8.1 to +15.8) | 436 (195-795) | +12.7% (+9.6 to +15.4) | 984 (464-1,771) |
|  | Amputation of fingers (excluding thumb) | 2020-2023 | +13.6% (+9.8 to +17.1) | 308 (126-618) | +11.4% (+7.6 to +15.6) | 226 (74-519) | +12.6% (+9.7 to +15.5) | 534 (206-1,126) |
|  | Fracture of face bones | 2020-2023 | +13.6% (+9.3 to +17.7) | 3,177 (1,578-5,227) | +10.5% (+5.7 to +15.6) | 1,587 (726-2,822) | +12.5% (+9.0 to +16.0) | 4,764 (2,340-8,008) |
|  | Fracture of patella, tibia or fibula, or ankle | 2020-2023 | +13.5% (+9.9 to +16.8) | 1,233 (616-2,170) | +11.2% (+7.1 to +15.4) | 971 (433-1,785) | +12.5% (+9.6 to +15.3) | 2,204 (1,051-3,844) |
|  | Open wound(s) | 2020-2023 | +13.6% (+10.0 to +17.1) | 13,599 (7,566-21,136) | +11.0% (+6.7 to +15.7) | 9,715 (5,222-15,772) | +12.5% (+9.7 to +15.6) | 23,315 (12,910-37,369) |
|  | Poisoning requiring urgent care | 2020-2023 | +13.5% (+10.0 to +16.8) | 741 (366-1,245) | +11.8% (+8.3 to +15.4) | 1,191 (543-2,142) | +12.5% (+9.6 to +15.3) | 1,932 (909-3,355) |
|  | Fracture of radius and/or ulna | 2020-2023 | +13.5% (+9.7 to +17.5) | 1,016 (481-1,844) | +11.4% (+7.5 to +15.4) | 1,064 (464-1,970) | +12.4% (+9.1 to +15.6) | 2,080 (926-3,827) |
|  | Amputation of upper limb, unilateral | 2020-2023 | +13.5% (+9.6 to +17.6) | 13 (4-30) | +11.1% (+6.6 to +15.7) | 11 (3-28) | +12.4% (+9.2 to +15.7) | 24 (7-58) |
|  | Muscle and tendon injuries, including sprains and strains lesser dislocations | 2020-2023 | +13.5% (+10.0 to +16.8) | 2,253 (1,249-3,703) | +11.3% (+7.3 to +15.6) | 2,348 (1,185-4,169) | +12.4% (+9.5 to +15.3) | 4,601 (2,538-7,884) |
|  | Fracture of foot bones except ankle | 2020-2023 | +13.6% (+9.6 to +17.3) | 507 (229-1,030) | +10.9% (+6.4 to +15.1) | 395 (130-894) | +12.4% (+9.3 to +15.7) | 902 (373-1,937) |
|  | Spinal cord lesion at neck level | 2020-2023 | +13.5% (+9.7 to +17.0) | 27 (12-56) | +10.9% (+6.4 to +15.2) | 21 (8-48) | +12.4% (+9.2 to +15.3) | 47 (19-102) |
|  | Spinal cord lesion below neck level | 2020-2023 | +13.6% (+10.0 to +17.5) | 40 (15-84) | +10.7% (+5.5 to +15.6) | 29 (9-75) | +12.4% (+9.1 to +15.6) | 68 (25-163) |
|  | Fracture of sternum and/or fracture of one or more ribs | 2020-2023 | +13.5% (+8.9 to +17.8) | 590 (237-1,214) | +10.1% (+4.4 to +15.7) | 311 (105-772) | +12.3% (+9.0 to +16.0) | 901 (359-1,982) |
|  | Fracture of hand (wrist and other distal part of hand) | 2020-2023 | +13.6% (+9.5 to +17.7) | 2,352 (1,148-4,091) | +10.4% (+5.2 to +16.0) | 1,536 (652-3,021) | +12.3% (+8.8 to +15.7) | 3,888 (1,873-7,100) |
|  | Burns, <20% total burned surface area without lower airway burns | 2020-2023 | +13.6% (+9.8 to +16.9) | 939 (416-1,780) | +11.2% (+7.2 to +16.3) | 1,125 (439-2,412) | +12.3% (+8.8 to +15.5) | 2,064 (845-4,080) |
|  | Injury to eyes | 2020-2023 | +13.6% (+9.3 to +17.5) | 2,186 (1,067-3,761) | +10.6% (+5.8 to +15.7) | 1,609 (729-3,167) | +12.3% (+8.8 to +15.5) | 3,795 (1,789-6,851) |
|  | Foreign body in GI and urogenital system | 2020-2023 | +13.6% (+10.1 to +17.7) | 254 (98-602) | +10.9% (+5.8 to +16.3) | 232 (64-665) | +12.3% (+9.0 to +15.4) | 486 (157-1,237) |
|  | Nerve injury | 2020-2023 | +13.6% (+9.5 to +17.4) | 559 (233-1,136) | +10.7% (+6.1 to +15.8) | 461 (164-1,141) | +12.2% (+9.0 to +15.6) | 1,020 (395-2,230) |
|  | Minor TBI | 2020-2023 | +13.6% (+9.4 to +17.5) | 1,580 (805-2,808) | +10.5% (+5.3 to +15.8) | 1,241 (587-2,327) | +12.2% (+8.9 to +15.6) | 2,821 (1,405-5,187) |
|  | Foreign body in respiratory system | 2020-2023 | +13.5% (+9.7 to +17.4) | 188 (70-399) | +10.9% (+6.7 to +15.7) | 194 (62-507) | +12.2% (+9.3 to +15.4) | 381 (138-897) |
|  | Amputation of toe/toes | 2020-2023 | +13.6% (+9.5 to +17.5) | 171 (60-394) | +10.7% (+6.3 to +15.7) | 159 (42-408) | +12.2% (+9.1 to +15.5) | 330 (101-765) |
|  | Fracture of vertebral column | 2020-2023 | +13.5% (+9.3 to +17.1) | 376 (164-734) | +10.8% (+6.7 to +15.8) | 353 (143-752) | +12.2% (+8.9 to +15.6) | 730 (312-1,462) |
|  | Fracture of clavicle, scapula, or humerus | 2020-2023 | +13.6% (+10.0 to +17.7) | 1,048 (473-1,964) | +10.8% (+6.4 to +16.3) | 1,055 (426-2,139) | +12.2% (+9.0 to +15.6) | 2,103 (911-4,098) |
|  | Amputation of thumb | 2020-2023 | +13.6% (+9.4 to +18.2) | 227 (77-504) | +10.6% (+5.6 to +15.8) | 212 (61-593) | +12.1% (+8.6 to +15.9) | 439 (140-1,080) |
|  | Effect of different environmental factors | 2020-2023 | +13.6% (+9.2 to +17.9) | 280 (107-602) | +10.9% (+5.9 to +15.8) | 332 (118-819) | +12.1% (+8.6 to +15.3) | 612 (226-1,430) |
|  | Dislocation of hip | 2020-2023 | +13.6% (+9.7 to +17.7) | 170 (59-383) | +10.7% (+6.0 to +16.5) | 183 (49-478) | +12.1% (+8.6 to +15.7) | 353 (111-887) |
|  | Lower airway burns | 2020-2023 | +13.6% (+9.5 to +18.3) | 13 (4-30) | +10.7% (+5.7 to +15.7) | 14 (4-38) | +12.1% (+8.6 to +16.0) | 28 (8-68) |
|  | Foreign body in ear | 2020-2023 | +13.6% (+9.6 to +17.5) | 253 (91-573) | +10.4% (+5.3 to +15.6) | 242 (68-649) | +12.0% (+8.6 to +15.8) | 495 (160-1,222) |
|  | Dislocation of knee | 2020-2023 | +13.6% (+9.2 to +17.8) | 233 (78-530) | +10.6% (+5.6 to +15.5) | 255 (68-669) | +12.0% (+8.5 to +15.8) | 488 (155-1,192) |
|  | Amputation of lower limbs, bilateral | 2020-2023 | +13.6% (+9.6 to +18.0) | 11 (3-26) | +10.6% (+5.5 to +15.8) | 12 (3-33) | +12.0% (+9.0 to +15.7) | 23 (6-58) |
|  | Asphyxiation | 2020-2023 | +13.7% (+9.2 to +17.6) | 250 (78-608) | +10.4% (+5.4 to +15.8) | 262 (63-786) | +12.0% (+8.5 to +15.5) | 512 (141-1,423) |
|  | Dislocation of shoulder | 2020-2023 | +13.6% (+9.5 to +17.4) | 333 (120-785) | +10.3% (+5.9 to +15.2) | 334 (98-860) | +11.9% (+8.8 to +15.3) | 667 (221-1,566) |
|  | Drowning and nonfatal submersion | 2020-2023 | +13.6% (+9.4 to +17.9) | 238 (73-545) | +10.4% (+5.3 to +15.7) | 257 (67-765) | +11.9% (+8.4 to +15.8) | 495 (141-1,310) |
|  | Fracture of hip | 2020-2023 | +13.4% (+9.5 to +17.2) | 49 (19-104) | +10.8% (+6.7 to +14.8) | 71 (28-156) | +11.9% (+8.8 to +15.0) | 120 (48-260) |
|  | Superficial injury of any part of the body | 2020-2023 | +13.6% (+9.5 to +18.1) | 5,912 (3,040-9,596) | +10.3% (+4.9 to +15.9) | 6,458 (2,900-11,195) | +11.9% (+8.1 to +15.8) | 12,370 (5,964-20,840) |
|  | Fracture of pelvis | 2020-2023 | +13.5% (+9.0 to +18.0) | 208 (68-521) | +10.4% (+5.0 to +15.3) | 266 (74-773) | +11.7% (+8.0 to +15.9) | 473 (137-1,246) |
|  | Contusion in any part of the body | 2020-2023 | +13.6% (+9.2 to +17.9) | 4,345 (2,464-7,517) | +10.1% (+4.5 to +16.1) | 5,237 (2,669-9,534) | +11.7% (+7.9 to +15.8) | 9,581 (5,190-16,891) |
| 15-19 years | Amputation of lower limb, unilateral | 2020-2023 | +8.6% (+2.1 to +14.5) | 3 (1-5) | +9.9% (+4.4 to +14.3) | 2 (1-5) | +9.2% (+3.2 to +13.6) | 5 (2-10) |
|  | Burns, >=20% total burned surface area or >= 10% burned surface area if head/neck or hands/wrist involved w/o lower airway burns | 2020-2023 | +8.2% (+1.3 to +14.2) | 35 (19-59) | +8.7% (+3.8 to +12.9) | 37 (18-68) | +8.5% (+3.3 to +12.6) | 72 (37-122) |
|  | Complications following therapeutic procedures | 2020-2023 | +8.2% (+1.8 to +13.1) | 125 (65-212) | +8.7% (+3.6 to +12.6) | 112 (53-212) | +8.4% (+3.4 to +12.5) | 237 (121-427) |
|  | Crush injury | 2020-2023 | +8.2% (+0.9 to +13.2) | 17 (8-31) | +8.4% (+3.2 to +12.1) | 8 (3-17) | +8.2% (+2.1 to +12.0) | 25 (12-47) |
|  | Moderate/Severe TBI | 2020-2023 | +7.9% (+0.4 to +13.0) | 513 (303-819) | +7.5% (+2.3 to +11.3) | 217 (113-388) | +7.8% (+1.4 to +11.9) | 731 (422-1,209) |
|  | Poisoning requiring urgent care | 2020-2023 | +7.9% (+0.3 to +13.4) | 384 (202-672) | +7.5% (+2.4 to +11.8) | 437 (196-834) | +7.7% (+2.4 to +11.6) | 820 (397-1,497) |
|  | Fracture of femur, other than femoral neck | 2020-2023 |  |  | +7.3% (+1.7 to +11.9) | 172 (65-344) | +7.6% (+1.1 to +12.1) | 440 (197-844) |
|  | Multiple fractures, dislocations, crashes, wounds, pains, and strains | 2020-2023 | +7.8% (+0.7 to +13.2) | 309 (153-584) | +7.2% (+2.0 to +11.4) | 173 (74-365) | +7.6% (+2.0 to +11.8) | 482 (229-942) |
|  | Internal hemorrhage in abdomen and pelvis | 2020-2023 | +7.6% (+0.6 to +13.2) | 1,375 (773-2,151) | +6.8% (+1.9 to +11.2) | 406 (198-719) | +7.5% (+1.0 to +12.0) | 1,782 (982-2,863) |
|  | Amputation of fingers (excluding thumb) | 2020-2023 |  |  | +6.8% (+1.3 to +11.0) | 83 (29-205) | +7.4% (+0.4 to +12.1) | 247 (108-529) |
|  | Amputation of upper limbs, bilateral | 2020-2023 | +7.6% (+0.5 to +14.0) | 10 (4-21) | +6.9% (+1.5 to +11.8) | 5 (1-12) | +7.4% (+1.4 to +12.0) | 15 (6-33) |
|  | Fracture of radius and/or ulna | 2020-2023 |  |  | +6.9% (+1.4 to +11.0) | 418 (184-822) | +7.4% (+0.9 to +11.2) | 985 (437-1,825) |
|  | Severe chest Injury | 2020-2023 |  |  | +6.4% (+0.8 to +10.5) | 280 (113-527) |  |  |
|  | Muscle and tendon injuries, including sprains and strains lesser dislocations | 2020-2023 |  |  | +6.7% (+0.7 to +11.5) | 875 (416-1,586) | +7.2% (+0.4 to +11.5) | 2,084 (1,075-3,511) |
|  | Fracture of patella, tibia or fibula, or ankle | 2020-2023 |  |  | +6.5% (+0.5 to +10.9) | 382 (175-751) | +7.2% (+0.5 to +11.3) | 1,078 (553-1,883) |
|  | Open wound(s) | 2020-2023 | +7.5% (+0.2 to +13.2) | 7,650 (4,476-12,021) | +6.3% (+1.1 to +10.4) | 3,770 (1,916-6,543) | +7.1% (+0.7 to +11.3) | 11,421 (6,491-18,606) |
|  | Amputation of upper limb, unilateral | 2020-2023 |  |  | +6.4% (+0.2 to +11.4) | 5 (1-14) | +7.1% (+0.3 to +11.8) | 14 (5-34) |
|  | Burns, <20% total burned surface area without lower airway burns | 2020-2023 |  |  | +6.5% (+1.1 to +11.3) | 376 (147-841) | +7.0% (+0.4 to +11.5) | 828 (382-1,676) |
|  | Foreign body in respiratory system | 2020-2023 |  |  | +6.2% (+0.0 to +10.6) | 75 (21-196) |  |  |
|  | Fracture of vertebral column | 2020-2023 |  |  | +6.0% (+0.4 to +10.7) | 148 (53-344) | +6.9% (+0.2 to +11.0) | 374 (153-756) |
|  | Fracture of hip | 2020-2023 |  |  | +6.2% (+0.4 to +10.8) | 43 (18-94) | +6.9% (+1.0 to +10.9) | 85 (36-183) |
|  | Foreign body in GI and urogenital system | 2020-2023 | +7.4% (+0.0 to +13.0) | 140 (54-306) | +6.0% (+0.3 to +10.6) | 88 (25-233) | +6.9% (+0.8 to +11.3) | 228 (79-544) |
|  | Amputation of toe/toes | 2020-2023 |  |  |  |  | +6.8% (+0.1 to +11.2) | 161 (49-413) |
|  | Injury to eyes | 2020-2023 |  |  |  |  | +6.8% (+0.0 to +11.1) | 1,860 (902-3,213) |
|  | Effect of different environmental factors | 2020-2023 |  |  |  |  | +6.7% (+0.7 to +11.5) | 276 (102-642) |
|  | Dislocation of hip | 2020-2023 |  |  | +5.9% (+0.3 to +11.1) | 72 (19-197) | +6.7% (+0.4 to +11.1) | 168 (56-417) |
|  | Lower airway burns | 2020-2023 |  |  |  |  | +6.7% (+0.1 to +11.2) | 15 (5-37) |
|  | Amputation of thumb | 2020-2023 |  |  |  |  | +6.6% (+0.2 to +11.3) | 204 (68-512) |
|  | Dislocation of shoulder | 2020-2023 |  |  |  |  | +6.6% (+0.2 to +11.3) | 340 (112-845) |
|  | Fracture of sternum and/or fracture of one or more ribs | 2020-2023 |  |  |  |  | +6.5% (+0.6 to +11.4) | 518 (202-1,112) |

## Finland

| **Age** | **Category** | **Period** | **Male %Change** | **Male Incidence 2023** | **Female %Change** | **Female Incidence 2023** | **Both %Change** | **Both Incidence 2023** |
| --- | --- | --- | --- | --- | --- | --- | --- | --- |
| 5-14 years | Fracture of skull | 2020-2023 |  |  |  |  | +9.1% (+0.6 to +19.5) | 51 (23-92) |
|  | Fracture of patella, tibia or fibula, or ankle | 2020-2023 |  |  |  |  | +8.6% (+0.2 to +17.3) | 25 (12-45) |
|  | Injury to eyes | 2020-2023 | +10.3% (+0.1 to +23.6) | 33 (16-57) |  |  |  |  |

## France

| **Age** | **Category** | **Period** | **Male %Change** | **Male Incidence 2023** | **Female %Change** | **Female Incidence 2023** | **Both %Change** | **Both Incidence 2023** |
| --- | --- | --- | --- | --- | --- | --- | --- | --- |
| 5-14 years | Severe chest Injury | 2020-2023 | +11.4% (+1.1 to +23.3) | 279 (146-484) |  |  | +10.4% (+1.9 to +18.2) | 394 (199-704) |
|  | Fracture of skull | 2020-2023 | +11.4% (+0.7 to +22.4) | 478 (225-861) |  |  | +10.4% (+2.0 to +17.6) | 678 (306-1,226) |
|  | Internal hemorrhage in abdomen and pelvis | 2020-2023 | +11.4% (+0.4 to +23.4) | 359 (191-604) |  |  | +10.4% (+2.2 to +17.9) | 515 (264-865) |
|  | Fracture of face bones | 2020-2023 |  |  |  |  | +10.2% (+1.5 to +17.9) | 966 (456-1,648) |
|  | Crush injury | 2020-2023 | +11.6% (+1.0 to +23.9) | 2 (1-4) |  |  | +10.2% (+2.4 to +17.3) | 4 (1-7) |
|  | Moderate/Severe TBI | 2020-2023 | +11.5% (+0.1 to +21.8) | 84 (46-136) |  |  | +10.2% (+1.7 to +16.8) | 136 (75-221) |
|  | Fracture of sternum and/or fracture of one or more ribs | 2020-2023 | +11.3% (+2.0 to +23.0) | 131 (53-273) |  |  | +10.2% (+2.6 to +17.7) | 203 (81-460) |
|  | Spinal cord lesion below neck level | 2020-2023 | +11.5% (+0.2 to +23.6) | 7 (3-16) |  |  | +10.0% (+1.8 to +17.0) | 13 (4-31) |
|  | Spinal cord lesion at neck level | 2020-2023 | +11.4% (+1.3 to +23.8) | 5 (2-10) |  |  | +10.0% (+2.2 to +17.1) | 8 (3-18) |
|  | Fracture of hand (wrist and other distal part of hand) | 2020-2023 | +11.3% (+1.1 to +23.5) | 486 (240-870) |  |  | +10.0% (+1.8 to +16.6) | 813 (393-1,493) |
|  | Amputation of upper limbs, bilateral | 2020-2023 |  |  |  |  | +10.0% (+1.5 to +17.2) | 4 (1-9) |
|  | Open wound(s) | 2020-2023 | +11.4% (+0.1 to +22.6) | 2,241 (1,264-3,596) |  |  | +10.0% (+2.0 to +16.7) | 3,884 (2,080-6,396) |
|  | Multiple fractures, dislocations, crashes, wounds, pains, and strains | 2020-2023 |  |  |  |  | +9.9% (+1.6 to +17.3) | 123 (55-229) |
|  | Minor TBI | 2020-2023 | +11.4% (+0.6 to +23.1) | 314 (161-549) |  |  | +9.9% (+1.8 to +17.5) | 569 (283-1,029) |
|  | Amputation of fingers (excluding thumb) | 2020-2023 |  |  |  |  | +9.9% (+1.2 to +16.7) | 75 (28-172) |
|  | Fracture of patella, tibia or fibula, or ankle | 2020-2023 | +11.4% (+1.0 to +21.8) | 187 (91-346) |  |  | +9.9% (+1.5 to +16.9) | 339 (156-609) |
|  | Injury to eyes | 2020-2023 | +11.3% (+0.3 to +21.9) | 423 (210-724) |  |  | +9.9% (+2.1 to +16.1) | 745 (357-1,348) |
|  | Amputation of upper limb, unilateral | 2020-2023 |  |  |  |  | +9.9% (+0.5 to +17.7) | 4 (1-10) |
|  | Nerve injury | 2020-2023 | +11.3% (+0.9 to +22.5) | 106 (43-225) |  |  | +9.8% (+1.5 to +17.2) | 197 (76-437) |
|  | Amputation of lower limb, unilateral | 2020-2023 |  |  |  |  | +9.8% (+1.9 to +17.8) | 0 (0-0) |
|  | Fracture of foot bones except ankle | 2020-2023 | +11.3% (+0.1 to +23.8) | 87 (38-184) |  |  | +9.8% (+1.0 to +17.7) | 157 (63-342) |
|  | Fracture of femur, other than femoral neck | 2020-2023 | +11.4% (+0.1 to +23.8) | 60 (26-123) |  |  | +9.8% (+1.5 to +16.9) | 115 (49-252) |
|  | Fracture of clavicle, scapula, or humerus | 2020-2023 | +11.3% (+0.2 to +22.9) | 191 (85-368) |  |  | +9.7% (+1.3 to +17.8) | 389 (162-754) |
|  | Amputation of lower limbs, bilateral | 2020-2023 |  |  |  |  | +9.7% (+1.1 to +16.7) | 4 (1-12) |
|  | Fracture of radius and/or ulna | 2020-2023 | +11.5% (+0.2 to +22.4) | 139 (61-254) |  |  | +9.7% (+1.6 to +16.8) | 287 (119-539) |
|  | Lower airway burns | 2020-2023 | +11.4% (+1.1 to +24.3) | 3 (1-6) |  |  | +9.6% (+1.1 to +17.3) | 5 (1-13) |
|  | Foreign body in respiratory system | 2020-2023 | +11.3% (+0.8 to +21.3) | 31 (11-72) |  |  | +9.6% (+2.1 to +17.0) | 64 (21-148) |
|  | Amputation of toe/toes | 2020-2023 | +11.3% (+1.1 to +23.5) | 32 (10-73) |  |  | +9.6% (+1.4 to +18.1) | 62 (18-146) |
|  | Superficial injury of any part of the body | 2020-2023 | +11.4% (+0.5 to +23.5) | 1,262 (641-2,042) |  |  | +9.6% (+1.1 to +17.1) | 2,687 (1,290-4,536) |
|  | Amputation of thumb | 2020-2023 |  |  |  |  | +9.6% (+0.7 to +17.2) | 84 (26-208) |
|  | Dislocation of hip | 2020-2023 |  |  |  |  | +9.6% (+1.0 to +18.1) | 66 (20-166) |
|  | Complications following therapeutic procedures | 2020-2023 | +11.5% (+0.5 to +22.5) | 15 (7-26) |  |  | +9.6% (+1.1 to +16.5) | 33 (15-61) |
|  | Muscle and tendon injuries, including sprains and strains lesser dislocations | 2020-2023 | +11.4% (+1.4 to +22.5) | 329 (171-569) |  |  | +9.6% (+2.3 to +16.7) | 681 (345-1,204) |
|  | Dislocation of shoulder | 2020-2023 | +11.5% (+1.4 to +22.9) | 67 (24-172) |  |  | +9.6% (+1.7 to +16.8) | 137 (44-336) |
|  | Asphyxiation | 2020-2023 | +11.4% (+0.4 to +22.1) | 52 (16-134) |  |  | +9.6% (+1.6 to +17.7) | 109 (29-306) |
|  | Fracture of hip | 2020-2023 | +11.7% (+0.6 to +23.6) | 8 (3-17) |  |  | +9.6% (+1.0 to +17.2) | 20 (8-45) |
|  | Fracture of pelvis | 2020-2023 |  |  |  |  | +9.6% (+1.1 to +18.2) | 98 (27-251) |
|  | Fracture of vertebral column | 2020-2023 | +11.3% (+0.2 to +21.7) | 65 (28-129) |  |  | +9.6% (+1.0 to +16.8) | 129 (53-273) |
|  | Foreign body in ear | 2020-2023 | +11.3% (+0.4 to +24.6) | 51 (17-120) |  |  | +9.5% (+0.8 to +17.3) | 101 (31-254) |
|  | Effect of different environmental factors | 2020-2023 | +11.2% (+0.3 to +23.8) | 51 (18-115) |  |  | +9.5% (+0.5 to +16.9) | 113 (40-274) |
|  | Drowning and nonfatal submersion | 2020-2023 |  |  |  |  | +9.5% (+1.5 to +17.4) | 102 (27-270) |
|  | Dislocation of knee | 2020-2023 | +11.3% (+0.1 to +22.4) | 46 (14-104) |  |  | +9.5% (+0.2 to +17.6) | 98 (30-241) |
|  | Contusion in any part of the body | 2020-2023 | +11.4% (+0.4 to +22.2) | 967 (547-1,694) |  |  | +9.5% (+1.2 to +17.7) | 2,175 (1,199-3,851) |
|  | Foreign body in GI and urogenital system | 2020-2023 |  |  |  |  | +9.5% (+0.0 to +17.2) | 85 (28-224) |
|  | Burns, >=20% total burned surface area or >= 10% burned surface area if head/neck or hands/wrist involved w/o lower airway burns | 2020-2023 | +11.4% (+0.3 to +21.5) | 4 (2-8) |  |  | +9.4% (+1.0 to +17.1) | 10 (4-21) |
|  | Burns, <20% total burned surface area without lower airway burns | 2020-2023 | +11.2% (+0.0 to +22.3) | 144 (64-275) |  |  |  |  |
|  | Poisoning requiring urgent care | 2020-2023 | +11.4% (+0.3 to +21.8) | 81 (40-140) |  |  | +9.2% (+1.1 to +17.2) | 214 (99-395) |

## Germany

| **Age** | **Category** | **Period** | **Male %Change** | **Male Incidence 2023** | **Female %Change** | **Female Incidence 2023** | **Both %Change** | **Both Incidence 2023** |
| --- | --- | --- | --- | --- | --- | --- | --- | --- |
| 5-14 years | Fracture of skull | 2020-2023 |  |  |  |  | +12.1% (+2.5 to +23.6) | 680 (318-1,221) |
|  | Severe chest Injury | 2020-2023 |  |  |  |  | +12.1% (+1.3 to +22.9) | 395 (196-718) |
|  | Internal hemorrhage in abdomen and pelvis | 2020-2023 |  |  |  |  | +12.0% (+1.9 to +22.7) | 516 (264-863) |
|  | Fracture of face bones | 2020-2023 |  |  |  |  | +11.9% (+2.8 to +21.9) | 966 (458-1,684) |
|  | Fracture of hand (wrist and other distal part of hand) | 2020-2023 |  |  |  |  | +11.6% (+1.6 to +21.8) | 810 (368-1,502) |
|  | Fracture of sternum and/or fracture of one or more ribs | 2020-2023 |  |  |  |  | +11.6% (+2.3 to +22.8) | 203 (79-449) |
|  | Moderate/Severe TBI | 2020-2023 | +13.6% (+0.3 to +25.8) | 85 (47-136) |  |  | +11.4% (+1.6 to +21.9) | 134 (75-224) |
|  | Crush injury | 2020-2023 |  |  |  |  | +11.4% (+2.6 to +21.1) | 4 (1-7) |
|  | Spinal cord lesion below neck level | 2020-2023 | +13.7% (+0.3 to +27.3) | 8 (3-17) |  |  | +11.4% (+1.4 to +23.0) | 13 (4-30) |
|  | Open wound(s) | 2020-2023 | +13.7% (+0.9 to +26.9) | 2,293 (1,268-3,643) |  |  | +11.4% (+2.1 to +20.7) | 3,858 (2,121-6,274) |
|  | Amputation of fingers (excluding thumb) | 2020-2023 |  |  |  |  | +11.4% (+1.5 to +21.8) | 75 (27-167) |
|  | Amputation of upper limbs, bilateral | 2020-2023 |  |  |  |  | +11.4% (+1.6 to +22.6) | 4 (1-8) |
|  | Fracture of foot bones except ankle | 2020-2023 |  |  |  |  | +11.4% (+1.7 to +21.8) | 156 (61-346) |
|  | Injury to eyes | 2020-2023 |  |  |  |  | +11.3% (+0.8 to +24.1) | 740 (347-1,387) |
|  | Nerve injury | 2020-2023 |  |  |  |  | +11.2% (+1.2 to +22.7) | 196 (76-437) |
|  | Minor TBI | 2020-2023 |  |  |  |  | +11.2% (+2.0 to +23.4) | 564 (277-1,062) |
|  | Fracture of patella, tibia or fibula, or ankle | 2020-2023 |  |  |  |  | +11.2% (+2.5 to +21.3) | 336 (159-591) |
|  | Multiple fractures, dislocations, crashes, wounds, pains, and strains | 2020-2023 |  |  |  |  | +11.1% (+0.9 to +23.4) | 121 (54-232) |
|  | Amputation of upper limb, unilateral | 2020-2023 |  |  |  |  | +11.1% (+1.5 to +24.2) | 4 (1-10) |
|  | Spinal cord lesion at neck level | 2020-2023 | +13.6% (+0.5 to +26.4) | 5 (2-10) |  |  | +11.1% (+3.0 to +22.1) | 8 (3-18) |
|  | Amputation of thumb | 2020-2023 |  |  |  |  | +11.1% (+1.3 to +23.5) | 84 (25-210) |
|  | Foreign body in GI and urogenital system | 2020-2023 |  |  |  |  | +11.1% (+1.3 to +22.3) | 84 (27-215) |
|  | Fracture of femur, other than femoral neck | 2020-2023 |  |  |  |  | +11.0% (+1.4 to +21.7) | 114 (48-244) |
|  | Amputation of toe/toes | 2020-2023 |  |  |  |  | +11.0% (+2.1 to +21.9) | 61 (18-148) |
|  | Foreign body in ear | 2020-2023 |  |  |  |  | +11.0% (+1.0 to +22.3) | 100 (30-246) |
|  | Asphyxiation | 2020-2023 |  |  |  |  | +11.0% (+2.4 to +20.7) | 108 (29-300) |
|  | Effect of different environmental factors | 2020-2023 |  |  |  |  | +11.0% (+1.8 to +22.5) | 112 (40-264) |
|  | Fracture of clavicle, scapula, or humerus | 2020-2023 |  |  |  |  | +10.9% (+1.0 to +22.5) | 384 (163-749) |
|  | Muscle and tendon injuries, including sprains and strains lesser dislocations | 2020-2023 |  |  |  |  | +10.9% (+2.4 to +21.3) | 672 (347-1,210) |
|  | Fracture of radius and/or ulna | 2020-2023 |  |  |  |  | +10.9% (+0.9 to +22.1) | 283 (121-527) |
|  | Drowning and nonfatal submersion | 2020-2023 |  |  |  |  | +10.9% (+1.9 to +23.1) | 101 (29-272) |
|  | Burns, <20% total burned surface area without lower airway burns | 2020-2023 |  |  |  |  | +10.9% (+1.6 to +22.3) | 318 (123-637) |
|  | Fracture of vertebral column | 2020-2023 |  |  |  |  | +10.9% (+1.9 to +21.9) | 127 (53-265) |
|  | Dislocation of knee | 2020-2023 |  |  |  |  | +10.9% (+1.9 to +22.9) | 97 (30-243) |
|  | Superficial injury of any part of the body | 2020-2023 |  |  |  |  | +10.8% (+1.6 to +23.4) | 2,649 (1,250-4,456) |
|  | Amputation of lower limbs, bilateral | 2020-2023 |  |  |  |  | +10.8% (+1.1 to +22.3) | 4 (1-11) |
|  | Dislocation of hip | 2020-2023 |  |  |  |  | +10.8% (+1.2 to +20.6) | 65 (19-163) |
|  | Lower airway burns | 2020-2023 | +13.7% (+0.3 to +27.6) | 3 (1-6) |  |  | +10.8% (+1.1 to +22.3) | 5 (1-12) |
|  | Dislocation of shoulder | 2020-2023 |  |  |  |  | +10.8% (+2.1 to +21.7) | 135 (44-332) |
|  | Foreign body in respiratory system | 2020-2023 |  |  |  |  | +10.7% (+1.5 to +21.9) | 63 (21-145) |
|  | Contusion in any part of the body | 2020-2023 |  |  |  |  | +10.7% (+1.7 to +22.3) | 2,141 (1,151-3,825) |
|  | Complications following therapeutic procedures | 2020-2023 | +13.6% (+0.4 to +26.0) | 15 (7-27) |  |  | +10.5% (+2.0 to +22.0) | 32 (16-61) |
|  | Poisoning requiring urgent care | 2020-2023 |  |  |  |  | +10.3% (+1.5 to +22.3) | 210 (96-389) |
|  | Burns, >=20% total burned surface area or >= 10% burned surface area if head/neck or hands/wrist involved w/o lower airway burns | 2020-2023 |  |  |  |  | +10.3% (+1.0 to +21.1) | 10 (4-21) |
|  | Fracture of pelvis | 2020-2023 |  |  |  |  | +10.3% (+0.8 to +22.0) | 96 (27-255) |
|  | Amputation of lower limb, unilateral | 2020-2023 |  |  |  |  | +10.2% (+0.6 to +22.1) | 0 (0-0) |
|  | Fracture of hip | 2020-2023 |  |  |  |  | +10.1% (+0.7 to +22.4) | 19 (8-45) |

## Greece

| **Age** | **Category** | **Period** | **Male %Change** | **Male Incidence 2023** | **Female %Change** | **Female Incidence 2023** | **Both %Change** | **Both Incidence 2023** |
| --- | --- | --- | --- | --- | --- | --- | --- | --- |
| 5-14 years | Internal hemorrhage in abdomen and pelvis | 2020-2023 |  |  |  |  | +10.7% (+0.9 to +19.9) | 63 (32-104) |
|  | Severe chest Injury | 2020-2023 |  |  |  |  | +10.6% (+0.2 to +21.1) | 48 (24-85) |
|  | Fracture of face bones | 2020-2023 |  |  |  |  | +10.6% (+0.4 to +19.6) | 117 (56-202) |
|  | Amputation of fingers (excluding thumb) | 2020-2023 |  |  |  |  | +10.5% (+0.2 to +19.1) | 9 (3-21) |
|  | Muscle and tendon injuries, including sprains and strains lesser dislocations | 2020-2023 |  |  |  |  | +10.4% (+0.2 to +18.7) | 83 (42-145) |
|  | Spinal cord lesion below neck level | 2020-2023 |  |  |  |  | +10.3% (+0.1 to +20.1) | 2 (1-4) |
|  | Fracture of patella, tibia or fibula, or ankle | 2020-2023 |  |  |  |  | +10.3% (+0.1 to +18.8) | 41 (19-74) |
|  | Foreign body in respiratory system | 2020-2023 |  |  |  |  | +10.3% (+0.1 to +19.9) | 8 (3-19) |

## Hungary

| **Age** | **Category** | **Period** | **Male %Change** | **Male Incidence 2023** | **Female %Change** | **Female Incidence 2023** | **Both %Change** | **Both Incidence 2023** |
| --- | --- | --- | --- | --- | --- | --- | --- | --- |
| <5 years | Severe chest Injury | 2020-2023 | +12.5% (+0.2 to +27.2) | 421 (259-634) |  |  |  |  |
|  | Fracture of skull | 2020-2023 | +12.5% (+0.6 to +27.0) | 211 (121-353) |  |  |  |  |
|  | Internal hemorrhage in abdomen and pelvis | 2020-2023 | +12.5% (+0.2 to +26.2) | 707 (459-994) |  |  |  |  |
|  | Fracture of face bones | 2020-2023 | +12.4% (+1.4 to +25.5) | 364 (232-571) |  |  |  |  |
|  | Fracture of sternum and/or fracture of one or more ribs | 2020-2023 | +12.5% (+0.9 to +26.5) | 30 (14-58) |  |  |  |  |
|  | Crush injury | 2020-2023 | +12.5% (+0.6 to +25.9) | 12 (6-20) |  |  |  |  |
|  | Fracture of hand (wrist and other distal part of hand) | 2020-2023 | +12.4% (+1.0 to +26.4) | 230 (140-360) |  |  |  |  |
|  | Severe chest Injury | 2010-2023 | +23.4% (+9.9 to +41.0) | 421 (259-634) |  |  |  |  |
|  | Amputation of upper limbs, bilateral | 2020-2023 | +12.3% (+0.3 to +26.2) | 5 (2-10) |  |  |  |  |
|  | Open wound(s) | 2020-2023 | +12.5% (+0.4 to +26.2) | 2,778 (2,017-3,619) |  |  |  |  |
|  | Amputation of fingers (excluding thumb) | 2020-2023 | +12.6% (+0.2 to +28.2) | 84 (44-155) |  |  |  |  |
|  | Spinal cord lesion below neck level | 2020-2023 | +12.5% (+0.2 to +25.0) | 7 (3-13) |  |  |  |  |
|  | Injury to eyes | 2020-2023 | +12.5% (+0.3 to +26.7) | 292 (177-447) |  |  |  |  |
|  | Fracture of skull | 2010-2023 | +23.4% (+9.9 to +39.5) | 211 (121-353) |  |  |  |  |
|  | Multiple fractures, dislocations, crashes, wounds, pains, and strains | 2020-2023 | +12.5% (+0.6 to +26.0) | 166 (95-275) |  |  |  |  |
|  | Fracture of patella, tibia or fibula, or ankle | 2020-2023 | +12.4% (+1.0 to +26.3) | 291 (178-439) |  |  |  |  |
|  | Fracture of foot bones except ankle | 2020-2023 | +12.4% (+0.3 to +25.4) | 101 (50-177) |  |  |  |  |
|  | Minor TBI | 2020-2023 | +12.5% (+1.1 to +25.5) | 177 (109-279) |  |  |  |  |
|  | Internal hemorrhage in abdomen and pelvis | 2010-2023 | +23.4% (+10.5 to +39.9) | 707 (459-994) |  |  |  |  |
|  | Amputation of upper limb, unilateral | 2020-2023 | +12.5% (+0.2 to +26.2) | 3 (1-7) |  |  |  |  |
|  | Nerve injury | 2020-2023 | +12.4% (+1.8 to +23.7) | 83 (42-153) |  |  |  |  |
|  | Fracture of femur, other than femoral neck | 2020-2023 | +12.5% (+1.1 to +25.4) | 161 (82-287) |  |  |  |  |
|  | Fracture of vertebral column | 2020-2023 | +12.5% (+0.3 to +26.7) | 66 (32-124) |  |  |  |  |
|  | Foreign body in GI and urogenital system | 2020-2023 | +12.3% (+0.0 to +26.8) | 49 (22-95) |  |  |  |  |
|  | Amputation of toe/toes | 2020-2023 | +12.5% (+0.9 to +25.4) | 26 (12-54) |  |  |  |  |
|  | Foreign body in ear | 2020-2023 | +12.6% (+0.4 to +24.7) | 31 (13-62) |  |  |  |  |
|  | Fracture of clavicle, scapula, or humerus | 2020-2023 | +12.4% (+0.5 to +25.4) | 164 (88-277) |  |  |  |  |
|  | Foreign body in respiratory system | 2020-2023 | +12.5% (+1.2 to +25.7) | 38 (18-76) |  |  |  |  |
|  | Fracture of radius and/or ulna | 2020-2023 | +12.6% (+1.3 to +26.8) | 273 (170-419) |  |  |  |  |
|  | Amputation of lower limb, unilateral | 2020-2023 | +12.5% (+0.8 to +26.8) | 2 (1-2) |  |  |  |  |
|  | Asphyxiation | 2020-2023 | +12.5% (+1.0 to +25.8) | 24 (9-55) |  |  |  |  |
|  | Dislocation of hip | 2020-2023 | +12.5% (+0.5 to +25.7) | 27 (11-52) |  |  |  |  |
|  | Superficial injury of any part of the body | 2020-2023 | +12.5% (+1.3 to +26.2) | 457 (301-664) |  |  |  |  |
|  | Drowning and nonfatal submersion | 2020-2023 | +12.4% (+0.9 to +25.4) | 28 (12-55) |  |  |  |  |
|  | Lower airway burns | 2020-2023 | +12.2% (+0.1 to +25.2) | 2 (1-5) |  |  |  |  |
|  | Contusion in any part of the body | 2020-2023 | +12.5% (+0.7 to +25.7) | 243 (159-368) |  |  |  |  |
|  | Burns, <20% total burned surface area without lower airway burns | 2020-2023 | +12.5% (+0.7 to +27.2) | 248 (138-416) |  |  |  |  |
|  | Fracture of pelvis | 2020-2023 | +12.7% (+1.0 to +27.2) | 20 (8-46) |  |  |  |  |
|  | Fracture of face bones | 2010-2023 | +23.4% (+10.6 to +39.1) | 364 (232-571) |  |  |  |  |
|  | Fracture of sternum and/or fracture of one or more ribs | 2010-2023 | +23.4% (+11.1 to +38.4) | 30 (14-58) |  |  |  |  |
|  | Fracture of hip | 2020-2023 | +12.7% (+0.6 to +25.5) | 7 (3-14) |  |  |  |  |
|  | Burns, >=20% total burned surface area or >= 10% burned surface area if head/neck or hands/wrist involved w/o lower airway burns | 2020-2023 | +12.3% (+0.6 to +27.1) | 32 (19-47) |  |  |  |  |
|  | Poisoning requiring urgent care | 2020-2023 | +12.5% (+0.7 to +26.0) | 262 (159-414) |  |  |  |  |
|  | Moderate/Severe TBI | 2010-2023 | +23.7% (+10.3 to +41.6) | 252 (191-331) |  |  |  |  |
|  | Crush injury | 2010-2023 | +23.7% (+11.2 to +39.6) | 12 (6-20) |  |  |  |  |
|  | Fracture of hand (wrist and other distal part of hand) | 2010-2023 | +23.4% (+11.6 to +39.6) | 230 (140-360) |  |  |  |  |
|  | Amputation of upper limbs, bilateral | 2010-2023 | +23.7% (+10.5 to +40.6) | 5 (2-10) |  |  |  |  |
|  | Spinal cord lesion below neck level | 2010-2023 | +23.8% (+10.7 to +42.5) | 7 (3-13) |  |  |  |  |
|  | Open wound(s) | 2010-2023 | +23.4% (+11.1 to +41.1) | 2,778 (2,017-3,619) |  |  |  |  |
|  | Amputation of fingers (excluding thumb) | 2010-2023 | +23.4% (+9.3 to +41.6) | 84 (44-155) |  |  |  |  |
|  | Injury to eyes | 2010-2023 | +23.3% (+11.7 to +40.9) | 292 (177-447) |  |  |  |  |
|  | Spinal cord lesion at neck level | 2010-2023 | +23.6% (+9.7 to +41.8) | 5 (3-9) |  |  |  |  |
|  | Multiple fractures, dislocations, crashes, wounds, pains, and strains | 2010-2023 | +23.3% (+11.5 to +38.2) | 166 (95-275) |  |  |  |  |
|  | Fracture of patella, tibia or fibula, or ankle | 2010-2023 | +23.4% (+10.5 to +38.1) | 291 (178-439) |  |  |  |  |
|  | Fracture of foot bones except ankle | 2010-2023 | +23.4% (+10.5 to +41.1) | 101 (50-177) |  |  |  |  |
|  | Minor TBI | 2010-2023 | +23.3% (+9.4 to +39.4) | 177 (109-279) |  |  |  |  |
|  | Amputation of upper limb, unilateral | 2010-2023 | +23.9% (+9.1 to +41.7) | 3 (1-7) |  |  |  |  |
|  | Nerve injury | 2010-2023 | +23.4% (+12.7 to +38.2) | 83 (42-153) |  |  |  |  |
|  | Fracture of femur, other than femoral neck | 2010-2023 | +23.3% (+11.2 to +38.8) | 161 (82-287) |  |  |  |  |
|  | Foreign body in GI and urogenital system | 2010-2023 | +23.3% (+9.6 to +41.0) | 49 (22-95) |  |  |  |  |
|  | Fracture of vertebral column | 2010-2023 | +23.5% (+10.9 to +40.3) | 66 (32-124) |  |  |  |  |
|  | Amputation of thumb | 2010-2023 | +23.3% (+11.1 to +40.5) | 34 (14-73) |  |  |  |  |
|  | Amputation of toe/toes | 2010-2023 | +23.4% (+10.1 to +42.1) | 26 (12-54) |  |  |  |  |
|  | Foreign body in ear | 2010-2023 | +23.6% (+10.4 to +39.2) | 31 (13-62) |  |  |  |  |
|  | Dislocation of shoulder | 2010-2023 | +23.4% (+9.5 to +39.6) | 35 (16-69) |  |  |  |  |
|  | Fracture of clavicle, scapula, or humerus | 2010-2023 | +23.3% (+10.3 to +38.4) | 164 (88-277) |  |  |  |  |
|  | Foreign body in respiratory system | 2010-2023 | +23.5% (+11.3 to +38.3) | 38 (18-76) |  |  |  |  |
|  | Muscle and tendon injuries, including sprains and strains lesser dislocations | 2010-2023 | +23.3% (+11.3 to +39.6) | 578 (376-833) |  |  |  |  |
|  | Amputation of lower limb, unilateral | 2010-2023 | +23.4% (+10.6 to +40.4) | 2 (1-2) |  |  |  |  |
|  | Fracture of radius and/or ulna | 2010-2023 | +23.4% (+10.7 to +39.0) | 273 (170-419) |  |  |  |  |
|  | Asphyxiation | 2010-2023 | +23.4% (+10.7 to +38.2) | 24 (9-55) |  |  |  |  |
|  | Lower airway burns | 2010-2023 | +23.7% (+11.3 to +39.4) | 2 (1-5) |  |  |  |  |
|  | Dislocation of hip | 2010-2023 | +23.3% (+10.3 to +39.0) | 27 (11-52) |  |  |  |  |
|  | Amputation of lower limbs, bilateral | 2010-2023 | +23.6% (+10.1 to +43.4) | 2 (1-3) |  |  |  |  |
|  | Drowning and nonfatal submersion | 2010-2023 | +23.4% (+8.7 to +39.4) | 28 (12-55) |  |  |  |  |
|  | Superficial injury of any part of the body | 2010-2023 | +23.3% (+11.2 to +38.5) | 457 (301-664) |  |  |  |  |
|  | Dislocation of knee | 2010-2023 | +23.2% (+10.7 to +40.5) | 29 (12-58) |  |  |  |  |
|  | Effect of different environmental factors | 2010-2023 | +23.5% (+10.8 to +39.9) | 48 (21-97) |  |  |  |  |
|  | Contusion in any part of the body | 2010-2023 | +23.4% (+10.1 to +39.1) | 243 (159-368) |  |  |  |  |
|  | Burns, <20% total burned surface area without lower airway burns | 2010-2023 | +23.4% (+11.0 to +39.3) | 248 (138-416) |  |  |  |  |
|  | Complications following therapeutic procedures | 2010-2023 | +23.3% (+11.4 to +38.0) | 83 (49-133) |  |  |  |  |
|  | Fracture of pelvis | 2010-2023 | +23.3% (+10.8 to +40.4) | 20 (8-46) |  |  |  |  |
|  | Fracture of hip | 2010-2023 | +23.2% (+11.0 to +37.6) | 7 (3-14) |  |  |  |  |
|  | Burns, >=20% total burned surface area or >= 10% burned surface area if head/neck or hands/wrist involved w/o lower airway burns | 2010-2023 | +23.7% (+11.4 to +39.8) | 32 (19-47) |  |  |  |  |
|  | Poisoning requiring urgent care | 2010-2023 | +23.4% (+11.6 to +40.0) | 262 (159-414) |  |  |  |  |
| 5-14 years | Drowning and nonfatal submersion | 2020-2023 | +12.2% (+1.4 to +25.9) | 4 (1-8) |  |  | +12.2% (+2.8 to +22.8) | 8 (2-20) |
|  | Asphyxiation | 2020-2023 | +12.2% (+0.2 to +27.5) | 3 (1-7) |  |  | +12.2% (+4.0 to +21.7) | 7 (2-16) |
|  | Amputation of thumb | 2020-2023 | +12.3% (+0.7 to +28.6) | 5 (2-10) |  |  | +12.2% (+2.3 to +22.9) | 9 (3-21) |
|  | Dislocation of knee | 2020-2023 | +12.2% (+1.0 to +27.7) | 4 (1-9) |  |  | +12.2% (+2.7 to +22.7) | 8 (3-19) |
|  | Burns, <20% total burned surface area without lower airway burns | 2020-2023 | +12.4% (+1.5 to +25.9) | 31 (14-62) |  |  | +12.2% (+3.7 to +21.5) | 68 (29-137) |
|  | Nerve injury | 2020-2023 | +12.3% (+0.5 to +27.0) | 11 (4-23) |  |  | +12.2% (+3.5 to +23.1) | 20 (8-43) |
|  | Foreign body in ear | 2020-2023 | +12.2% (+1.3 to +30.7) | 4 (1-9) |  |  | +12.1% (+3.9 to +22.2) | 8 (3-18) |
|  | Fracture of skull | 2020-2023 | +12.2% (+0.6 to +27.0) | 29 (14-52) | +12.0% (+0.1 to +25.9) | 11 (4-23) | +12.1% (+4.2 to +22.4) | 40 (18-73) |
|  | Internal hemorrhage in abdomen and pelvis | 2020-2023 | +12.1% (+1.7 to +27.6) | 98 (54-159) |  |  | +12.1% (+5.2 to +21.3) | 138 (72-225) |
|  | Fracture of pelvis | 2020-2023 |  |  |  |  | +12.1% (+3.2 to +22.6) | 7 (2-18) |
|  | Severe chest Injury | 2020-2023 | +12.1% (+1.9 to +29.0) | 60 (32-105) |  |  | +12.1% (+5.1 to +23.8) | 82 (42-153) |
|  | Injury to eyes | 2020-2023 | +12.2% (+1.6 to +25.5) | 41 (21-71) | +12.0% (+0.0 to +24.3) | 30 (13-57) | +12.1% (+4.5 to +20.9) | 70 (34-126) |
|  | Effect of different environmental factors | 2020-2023 |  |  |  |  | +12.1% (+3.4 to +23.1) | 14 (5-31) |
|  | Poisoning requiring urgent care | 2020-2023 | +12.1% (+0.6 to +28.7) | 37 (18-64) | +12.1% (+0.2 to +24.9) | 58 (27-106) | +12.1% (+2.9 to +21.5) | 95 (45-167) |
|  | Contusion in any part of the body | 2020-2023 | +12.2% (+1.3 to +28.5) | 34 (18-59) |  |  | +12.1% (+3.0 to +21.5) | 75 (38-134) |
|  | Foreign body in GI and urogenital system | 2020-2023 | +12.1% (+0.1 to +30.0) | 7 (3-13) |  |  | +12.1% (+2.7 to +22.1) | 13 (4-29) |
|  | Amputation of fingers (excluding thumb) | 2020-2023 |  |  |  |  | +12.1% (+5.1 to +22.3) | 20 (8-42) |
|  | Open wound(s) | 2020-2023 | +12.1% (+0.8 to +28.9) | 398 (220-615) |  |  | +12.1% (+4.7 to +22.3) | 678 (376-1,061) |
|  | Fracture of hand (wrist and other distal part of hand) | 2020-2023 | +12.1% (+0.5 to +29.6) | 32 (16-58) |  |  | +12.1% (+3.7 to +22.3) | 54 (26-99) |
|  | Amputation of upper limbs, bilateral | 2020-2023 | +12.1% (+0.9 to +29.6) | 1 (0-1) |  |  | +12.0% (+4.4 to +22.3) | 1 (0-2) |
|  | Spinal cord lesion below neck level | 2020-2023 | +12.1% (+1.3 to +26.6) | 1 (0-2) | +11.9% (+0.1 to +25.7) | 1 (0-1) | +12.0% (+4.5 to +21.0) | 1 (1-3) |
|  | Fracture of face bones | 2020-2023 |  |  | +11.9% (+0.1 to +26.7) | 25 (12-44) | +12.0% (+4.4 to +22.2) | 76 (37-127) |
|  | Muscle and tendon injuries, including sprains and strains lesser dislocations | 2020-2023 | +12.2% (+1.0 to +30.9) | 81 (45-136) |  |  | +12.0% (+4.0 to +22.1) | 165 (88-274) |
|  | Minor TBI | 2020-2023 |  |  |  |  | +12.0% (+4.1 to +21.5) | 47 (22-85) |
|  | Fracture of sternum and/or fracture of one or more ribs | 2020-2023 | +11.9% (+0.6 to +25.8) | 5 (2-10) |  |  | +12.0% (+4.6 to +23.8) | 7 (3-16) |
|  | Fracture of patella, tibia or fibula, or ankle | 2020-2023 | +12.0% (+1.6 to +30.0) | 42 (20-72) |  |  | +12.0% (+4.5 to +21.9) | 74 (35-125) |
|  | Fracture of femur, other than femoral neck | 2020-2023 | +12.1% (+0.1 to +27.4) | 23 (11-48) |  |  | +12.0% (+4.3 to +20.9) | 44 (20-92) |
|  | Superficial injury of any part of the body | 2020-2023 | +12.0% (+1.4 to +27.3) | 67 (33-109) |  |  | +12.0% (+3.1 to +22.1) | 139 (65-238) |
|  | Fracture of foot bones except ankle | 2020-2023 |  |  |  |  | +12.0% (+3.4 to +24.2) | 24 (9-48) |
|  | Dislocation of shoulder | 2020-2023 | +12.0% (+1.6 to +26.7) | 5 (2-10) | +11.9% (+0.1 to +25.3) | 5 (2-12) | +12.0% (+3.4 to +21.0) | 10 (4-22) |
|  | Amputation of toe/toes | 2020-2023 | +11.9% (+1.1 to +27.0) | 4 (1-8) |  |  | +12.0% (+3.2 to +22.1) | 7 (2-16) |
|  | Fracture of radius and/or ulna | 2020-2023 | +12.0% (+0.6 to +30.9) | 40 (19-75) |  |  | +12.0% (+3.8 to +22.5) | 82 (37-149) |
|  | Fracture of clavicle, scapula, or humerus | 2020-2023 | +12.0% (+1.4 to +26.0) | 24 (11-44) |  |  | +11.9% (+3.7 to +21.2) | 48 (21-94) |
|  | Lower airway burns | 2020-2023 |  |  |  |  | +11.9% (+3.0 to +23.1) | 1 (0-1) |
|  | Spinal cord lesion at neck level | 2020-2023 | +12.1% (+1.4 to +26.3) | 1 (0-1) | +11.8% (+0.1 to +26.3) | 1 (0-1) | +11.9% (+5.3 to +21.4) | 1 (0-2) |
|  | Burns, >=20% total burned surface area or >= 10% burned surface area if head/neck or hands/wrist involved w/o lower airway burns | 2020-2023 | +11.9% (+1.0 to +27.9) | 4 (2-7) |  |  | +11.9% (+3.2 to +21.7) | 10 (5-17) |
|  | Fracture of vertebral column | 2020-2023 |  |  |  |  | +11.9% (+4.1 to +22.0) | 19 (8-37) |
|  | Amputation of upper limb, unilateral | 2020-2023 | +12.0% (+1.4 to +30.1) | 0 (0-1) |  |  | +11.9% (+3.9 to +21.8) | 1 (0-2) |
|  | Multiple fractures, dislocations, crashes, wounds, pains, and strains | 2020-2023 | +12.0% (+1.2 to +28.9) | 25 (12-42) |  |  | +11.9% (+4.0 to +22.3) | 43 (20-74) |
|  | Foreign body in respiratory system | 2020-2023 | +12.2% (+1.7 to +28.8) | 5 (2-12) |  |  | +11.9% (+4.0 to +22.1) | 11 (4-24) |
|  | Dislocation of hip | 2020-2023 |  |  |  |  | +11.8% (+2.2 to +22.0) | 8 (3-18) |
|  | Crush injury | 2020-2023 | +11.9% (+0.4 to +27.7) | 2 (1-3) |  |  | +11.8% (+3.9 to +21.2) | 3 (1-5) |
|  | Moderate/Severe TBI | 2020-2023 | +11.8% (+0.6 to +26.3) | 36 (21-58) |  |  | +11.8% (+4.3 to +21.8) | 56 (33-86) |
|  | Amputation of lower limbs, bilateral | 2020-2023 | +12.0% (+1.2 to +28.6) | 0 (0-0) | +11.6% (+0.2 to +28.4) | 0 (0-1) | +11.8% (+3.5 to +22.3) | 0 (0-1) |
|  | Complications following therapeutic procedures | 2020-2023 | +11.9% (+2.1 to +29.0) | 14 (7-25) |  |  | +11.8% (+2.5 to +20.9) | 30 (15-57) |
|  | Fracture of hip | 2020-2023 |  |  |  |  | +11.5% (+2.0 to +20.5) | 3 (1-8) |
|  | Amputation of lower limb, unilateral | 2020-2023 | +11.4% (+0.5 to +27.0) | 0 (0-1) |  |  | +11.5% (+2.9 to +21.1) | 1 (0-1) |
| <20 years | Severe chest Injury | 2020-2023 | +11.5% (+0.6 to +24.0) | 506 (324-749) |  |  |  |  |
|  | Fracture of skull | 2020-2023 | +11.5% (+0.6 to +24.2) | 252 (143-414) |  |  |  |  |
|  | Internal hemorrhage in abdomen and pelvis | 2020-2023 | +11.5% (+0.5 to +23.6) | 846 (555-1,189) |  |  |  |  |
|  | Fracture of sternum and/or fracture of one or more ribs | 2020-2023 | +11.5% (+0.8 to +22.9) | 37 (17-71) |  |  |  |  |
|  | Fracture of face bones | 2020-2023 | +11.4% (+1.4 to +22.3) | 437 (284-671) |  |  |  |  |
|  | Severe chest Injury | 2010-2023 | +21.0% (+10.8 to +35.6) | 506 (324-749) |  |  |  |  |
|  | Fracture of skull | 2010-2023 | +21.4% (+10.4 to +36.2) | 252 (143-414) |  |  |  |  |
|  | Moderate/Severe TBI | 2020-2023 | +11.3% (+0.5 to +23.0) | 308 (229-401) |  |  |  |  |
|  | Internal hemorrhage in abdomen and pelvis | 2010-2023 | +21.1% (+9.8 to +35.1) | 846 (555-1,189) |  |  |  |  |
|  | Crush injury | 2020-2023 | +11.4% (+0.7 to +23.2) | 15 (8-25) |  |  |  |  |
|  | Fracture of hand (wrist and other distal part of hand) | 2020-2023 | +11.4% (+0.3 to +23.4) | 277 (168-425) |  |  |  |  |
|  | Amputation of upper limbs, bilateral | 2020-2023 | +11.3% (+0.3 to +22.8) | 6 (3-13) |  |  |  |  |
|  | Open wound(s) | 2020-2023 | +11.4% (+0.7 to +23.1) | 3,346 (2,463-4,321) |  |  |  |  |
|  | Amputation of fingers (excluding thumb) | 2020-2023 | +11.6% (+0.3 to +25.2) | 101 (52-184) |  |  |  |  |
|  | Injury to eyes | 2020-2023 | +11.5% (+0.5 to +23.8) | 350 (211-553) |  |  |  |  |
|  | Spinal cord lesion below neck level | 2020-2023 | +11.5% (+0.3 to +22.3) | 8 (4-15) |  |  |  |  |
|  | Multiple fractures, dislocations, crashes, wounds, pains, and strains | 2020-2023 | +11.5% (+0.7 to +23.0) | 201 (115-340) |  |  |  |  |
|  | Fracture of patella, tibia or fibula, or ankle | 2020-2023 | +11.4% (+1.7 to +23.6) | 352 (213-521) |  |  |  |  |
|  | Minor TBI | 2020-2023 | +11.4% (+0.6 to +22.3) | 215 (129-335) |  |  |  |  |
|  | Fracture of foot bones except ankle | 2020-2023 | +11.4% (+0.6 to +22.2) | 120 (62-210) |  |  |  |  |
|  | Amputation of upper limb, unilateral | 2020-2023 | +11.4% (+0.5 to +22.1) | 4 (2-8) |  |  |  |  |
|  | Nerve injury | 2020-2023 | +11.4% (+2.0 to +22.1) | 98 (50-188) |  |  |  |  |
|  | Fracture of femur, other than femoral neck | 2020-2023 | +11.5% (+1.8 to +22.6) | 194 (101-343) |  |  |  |  |
|  | Amputation of lower limb, unilateral | 2020-2023 | +11.2% (+1.6 to +23.4) | 2 (1-3) |  |  |  |  |
|  | Fracture of vertebral column | 2020-2023 | +11.5% (+0.8 to +23.6) | 80 (38-150) |  |  |  |  |
|  | Foreign body in GI and urogenital system | 2020-2023 | +11.4% (+0.5 to +23.7) | 58 (27-112) |  |  |  |  |
|  | Amputation of toe/toes | 2020-2023 | +11.4% (+1.5 to +22.7) | 31 (14-65) |  |  |  |  |
|  | Fracture of face bones | 2010-2023 | +21.1% (+9.7 to +33.6) | 437 (284-671) |  |  |  |  |
|  | Dislocation of shoulder | 2020-2023 | +11.5% (+0.1 to +23.9) | 43 (20-83) |  |  |  |  |
|  | Foreign body in ear | 2020-2023 | +11.7% (+0.3 to +22.3) | 36 (15-71) |  |  |  |  |
|  | Fracture of clavicle, scapula, or humerus | 2020-2023 | +11.4% (+0.9 to +22.7) | 198 (113-334) |  |  |  |  |
|  | Foreign body in respiratory system | 2020-2023 | +11.5% (+0.8 to +22.1) | 46 (22-93) |  |  |  |  |
|  | Fracture of radius and/or ulna | 2020-2023 | +11.5% (+1.3 to +23.8) | 331 (202-510) |  |  |  |  |
|  | Muscle and tendon injuries, including sprains and strains lesser dislocations | 2020-2023 | +11.5% (+0.4 to +23.2) | 694 (471-1,007) |  |  |  |  |
|  | Fracture of sternum and/or fracture of one or more ribs | 2010-2023 | +20.3% (+10.3 to +32.0) | 37 (17-71) |  |  |  |  |
|  | Superficial injury of any part of the body | 2020-2023 | +11.5% (+1.2 to +23.4) | 553 (365-797) |  |  |  |  |
|  | Dislocation of hip | 2020-2023 | +11.5% (+0.2 to +23.6) | 32 (13-62) |  |  |  |  |
|  | Asphyxiation | 2020-2023 | +11.5% (+1.2 to +23.7) | 29 (11-63) |  |  |  |  |
|  | Drowning and nonfatal submersion | 2020-2023 | +11.4% (+1.6 to +23.7) | 33 (14-64) |  |  |  |  |
|  | Dislocation of knee | 2020-2023 | +11.4% (+0.5 to +23.5) | 35 (15-71) |  |  |  |  |
|  | Complications following therapeutic procedures | 2020-2023 | +11.4% (+0.8 to +22.7) | 105 (63-165) |  |  |  |  |
|  | Fracture of hip | 2020-2023 | +11.4% (+1.9 to +20.8) | 9 (4-18) |  |  |  |  |
|  | Fracture of pelvis | 2020-2023 | +11.6% (+1.2 to +23.8) | 25 (10-58) |  |  |  |  |
|  | Contusion in any part of the body | 2020-2023 | +11.5% (+1.3 to +22.9) | 292 (188-449) |  |  |  |  |
|  | Effect of different environmental factors | 2020-2023 | +11.5% (+0.9 to +23.9) | 57 (24-116) |  |  |  |  |
|  | Burns, <20% total burned surface area without lower airway burns | 2020-2023 | +11.5% (+0.8 to +23.7) | 291 (169-485) |  |  |  |  |
|  | Burns, >=20% total burned surface area or >= 10% burned surface area if head/neck or hands/wrist involved w/o lower airway burns | 2020-2023 | +11.3% (+0.6 to +23.7) | 38 (23-56) |  |  |  |  |
|  | Poisoning requiring urgent care | 2020-2023 | +11.5% (+0.9 to +23.6) | 315 (196-489) |  |  |  |  |
|  | Moderate/Severe TBI | 2010-2023 | +20.1% (+9.3 to +33.4) | 308 (229-401) |  |  |  |  |
|  | Crush injury | 2010-2023 | +20.6% (+10.4 to +33.5) | 15 (8-25) |  |  |  |  |
|  | Fracture of hand (wrist and other distal part of hand) | 2010-2023 | +21.0% (+11.1 to +34.2) | 277 (168-425) |  |  |  |  |
|  | Amputation of upper limbs, bilateral | 2010-2023 | +21.3% (+11.1 to +34.3) | 6 (3-13) |  |  |  |  |
|  | Spinal cord lesion below neck level | 2010-2023 | +21.8% (+11.6 to +36.7) | 8 (4-15) |  |  |  |  |
|  | Open wound(s) | 2010-2023 | +20.9% (+10.9 to +33.8) | 3,346 (2,463-4,321) |  |  |  |  |
|  | Amputation of fingers (excluding thumb) | 2010-2023 | +21.1% (+9.3 to +35.2) | 101 (52-184) |  |  |  |  |
|  | Injury to eyes | 2010-2023 | +21.1% (+10.7 to +34.5) | 350 (211-553) |  |  |  |  |
|  | Fracture of foot bones except ankle | 2010-2023 | +21.3% (+11.0 to +35.6) | 120 (62-210) |  |  |  |  |
|  | Spinal cord lesion at neck level | 2010-2023 | +20.3% (+9.9 to +34.7) | 6 (3-12) |  |  |  |  |
|  | Fracture of patella, tibia or fibula, or ankle | 2010-2023 | +20.7% (+10.9 to +32.9) | 352 (213-521) |  |  |  |  |
|  | Multiple fractures, dislocations, crashes, wounds, pains, and strains | 2010-2023 | +20.4% (+10.4 to +33.0) | 201 (115-340) |  |  |  |  |
|  | Minor TBI | 2010-2023 | +20.4% (+9.6 to +33.4) | 215 (129-335) |  |  |  |  |
|  | Amputation of upper limb, unilateral | 2010-2023 | +21.4% (+10.2 to +35.3) | 4 (2-8) |  |  |  |  |
|  | Nerve injury | 2010-2023 | +21.4% (+12.1 to +33.8) | 98 (50-188) |  |  |  |  |
|  | Fracture of femur, other than femoral neck | 2010-2023 | +20.7% (+10.0 to +33.3) | 194 (101-343) |  |  |  |  |
|  | Foreign body in GI and urogenital system | 2010-2023 | +21.1% (+10.0 to +35.4) | 58 (27-112) |  |  |  |  |
|  | Amputation of thumb | 2010-2023 | +21.3% (+10.6 to +34.8) | 40 (17-87) |  |  |  |  |
|  | Fracture of vertebral column | 2010-2023 | +20.6% (+10.3 to +32.9) | 80 (38-150) |  |  |  |  |
|  | Amputation of toe/toes | 2010-2023 | +20.9% (+10.3 to +34.8) | 31 (14-65) |  |  |  |  |
|  | Foreign body in ear | 2010-2023 | +21.9% (+10.1 to +35.1) | 36 (15-71) |  |  |  |  |
|  | Dislocation of shoulder | 2010-2023 | +20.7% (+9.8 to +34.9) | 43 (20-83) |  |  |  |  |
|  | Fracture of clavicle, scapula, or humerus | 2010-2023 | +20.6% (+10.3 to +32.5) | 198 (113-334) |  |  |  |  |
|  | Foreign body in respiratory system | 2010-2023 | +20.9% (+10.4 to +34.3) | 46 (22-93) |  |  |  |  |
|  | Muscle and tendon injuries, including sprains and strains lesser dislocations | 2010-2023 | +21.0% (+10.7 to +34.1) | 694 (471-1,007) |  |  |  |  |
|  | Asphyxiation | 2010-2023 | +21.7% (+11.3 to +34.6) | 29 (11-63) |  |  |  |  |
|  | Fracture of radius and/or ulna | 2010-2023 | +20.5% (+10.9 to +33.4) | 331 (202-510) |  |  |  |  |
|  | Lower airway burns | 2010-2023 | +21.7% (+11.1 to +35.2) | 3 (1-6) |  |  |  |  |
|  | Dislocation of hip | 2010-2023 | +20.9% (+10.3 to +34.8) | 32 (13-62) |  |  |  |  |
|  | Drowning and nonfatal submersion | 2010-2023 | +21.7% (+10.0 to +36.4) | 33 (14-64) |  |  |  |  |
|  | Amputation of lower limbs, bilateral | 2010-2023 | +21.5% (+10.6 to +36.7) | 2 (1-4) |  |  |  |  |
|  | Superficial injury of any part of the body | 2010-2023 | +20.7% (+10.2 to +33.6) | 553 (365-797) |  |  |  |  |
|  | Dislocation of knee | 2010-2023 | +21.2% (+10.0 to +35.5) | 35 (15-71) |  |  |  |  |
|  | Amputation of lower limb, unilateral | 2010-2023 | +16.2% (+6.6 to +28.3) | 2 (1-3) |  |  |  |  |
|  | Effect of different environmental factors | 2010-2023 | +21.6% (+11.0 to +35.0) | 57 (24-116) |  |  |  |  |
|  | Burns, <20% total burned surface area without lower airway burns | 2010-2023 | +22.1% (+11.9 to +37.7) | 291 (169-485) |  |  |  |  |
|  | Contusion in any part of the body | 2010-2023 | +21.0% (+10.9 to +34.1) | 292 (188-449) |  |  |  |  |
|  | Complications following therapeutic procedures | 2010-2023 | +19.2% (+9.6 to +31.0) | 105 (63-165) |  |  |  |  |
|  | Fracture of pelvis | 2010-2023 | +19.9% (+9.3 to +34.2) | 25 (10-58) |  |  |  |  |
|  | Fracture of hip | 2010-2023 | +16.1% (+6.6 to +27.5) | 9 (4-18) |  |  |  |  |
|  | Burns, >=20% total burned surface area or >= 10% burned surface area if head/neck or hands/wrist involved w/o lower airway burns | 2010-2023 | +21.2% (+10.5 to +34.3) | 38 (23-56) |  |  |  |  |
|  | Poisoning requiring urgent care | 2010-2023 | +21.0% (+10.8 to +34.5) | 315 (196-489) |  |  |  |  |

## Ireland

| **Age** | **Category** | **Period** | **Male %Change** | **Male Incidence 2023** | **Female %Change** | **Female Incidence 2023** | **Both %Change** | **Both Incidence 2023** |
| --- | --- | --- | --- | --- | --- | --- | --- | --- |
| 5-14 years | Amputation of lower limb, unilateral | 2020-2023 | +11.5% (+1.5 to +23.0) | 0 (0-0) |  |  | +9.7% (+1.5 to +20.2) | 0 (0-0) |
|  | Moderate/Severe TBI | 2020-2023 | +10.8% (+1.0 to +22.8) | 7 (4-12) |  |  | +9.5% (+2.0 to +17.6) | 12 (7-20) |
|  | Severe chest Injury | 2020-2023 | +10.4% (+1.3 to +23.7) | 25 (12-43) |  |  | +9.4% (+2.1 to +18.7) | 35 (18-63) |
|  | Crush injury | 2020-2023 |  |  |  |  | +9.4% (+1.8 to +17.9) | 0 (0-1) |
|  | Internal hemorrhage in abdomen and pelvis | 2020-2023 | +10.4% (+0.1 to +22.4) | 32 (17-53) |  |  | +9.4% (+2.5 to +17.7) | 46 (23-76) |
|  | Fracture of skull | 2020-2023 | +10.3% (+0.9 to +21.8) | 43 (20-74) |  |  | +9.4% (+1.2 to +18.1) | 61 (28-109) |
|  | Fracture of sternum and/or fracture of one or more ribs | 2020-2023 | +10.4% (+0.1 to +21.7) | 12 (5-24) |  |  | +9.3% (+0.8 to +17.3) | 18 (7-40) |
|  | Spinal cord lesion at neck level | 2020-2023 | +10.7% (+1.5 to +22.4) | 0 (0-1) |  |  | +9.2% (+2.4 to +18.3) | 1 (0-2) |
|  | Fracture of face bones | 2020-2023 |  |  |  |  | +9.2% (+0.5 to +18.2) | 87 (41-148) |
|  | Amputation of upper limbs, bilateral | 2020-2023 | +10.7% (+0.0 to +21.6) | 0 (0-0) |  |  | +9.1% (+1.2 to +19.2) | 0 (0-1) |
|  | Fracture of hip | 2020-2023 | +11.1% (+0.8 to +23.7) | 1 (0-1) |  |  | +9.1% (+1.5 to +18.9) | 2 (1-4) |
|  | Amputation of upper limb, unilateral | 2020-2023 |  |  |  |  | +9.1% (+0.8 to +19.6) | 0 (0-1) |
|  | Spinal cord lesion below neck level | 2020-2023 | +10.4% (+0.6 to +22.9) | 1 (0-1) |  |  | +9.0% (+2.5 to +18.4) | 1 (0-3) |
|  | Fracture of hand (wrist and other distal part of hand) | 2020-2023 | +10.3% (+0.6 to +23.4) | 44 (20-79) |  |  | +9.0% (+1.5 to +18.1) | 73 (34-138) |
|  | Open wound(s) | 2020-2023 | +10.4% (+0.8 to +22.0) | 201 (108-322) |  |  | +9.0% (+2.0 to +18.4) | 350 (184-569) |
|  | Fracture of patella, tibia or fibula, or ankle | 2020-2023 |  |  |  |  | +8.9% (+2.4 to +17.5) | 31 (14-55) |
|  | Multiple fractures, dislocations, crashes, wounds, pains, and strains | 2020-2023 | +10.5% (+1.4 to +23.0) | 6 (3-12) |  |  | +8.9% (+2.0 to +17.9) | 11 (5-21) |
|  | Complications following therapeutic procedures | 2020-2023 | +10.9% (+1.7 to +21.0) | 1 (1-2) |  |  | +8.9% (+2.2 to +18.7) | 3 (1-5) |
|  | Minor TBI | 2020-2023 |  |  |  |  | +8.9% (+1.7 to +18.4) | 51 (25-94) |
|  | Injury to eyes | 2020-2023 | +10.3% (+0.2 to +22.4) | 38 (18-65) |  |  | +8.9% (+2.3 to +18.0) | 67 (30-119) |
|  | Amputation of fingers (excluding thumb) | 2020-2023 | +10.3% (+0.1 to +23.6) | 4 (1-8) |  |  | +8.9% (+1.1 to +18.7) | 7 (2-15) |
|  | Fracture of femur, other than femoral neck | 2020-2023 | +10.5% (+0.5 to +22.6) | 5 (2-11) |  |  | +8.9% (+1.5 to +18.4) | 10 (4-22) |
|  | Fracture of vertebral column | 2020-2023 |  |  |  |  | +8.8% (+1.5 to +17.7) | 12 (5-24) |
|  | Dislocation of shoulder | 2020-2023 |  |  |  |  | +8.8% (+0.8 to +18.9) | 12 (4-32) |
|  | Fracture of foot bones except ankle | 2020-2023 |  |  |  |  | +8.8% (+1.3 to +19.3) | 14 (6-31) |
|  | Nerve injury | 2020-2023 | +10.3% (+0.9 to +24.0) | 10 (4-20) |  |  | +8.8% (+1.8 to +19.4) | 18 (6-39) |
|  | Lower airway burns | 2020-2023 |  |  |  |  | +8.7% (+1.5 to +18.3) | 0 (0-1) |
|  | Foreign body in respiratory system | 2020-2023 |  |  |  |  | +8.6% (+2.1 to +18.8) | 6 (2-14) |
|  | Fracture of clavicle, scapula, or humerus | 2020-2023 | +10.5% (+0.6 to +22.3) | 17 (8-33) |  |  | +8.6% (+0.1 to +18.3) | 35 (15-69) |
|  | Fracture of radius and/or ulna | 2020-2023 | +10.5% (+0.1 to +23.5) | 12 (5-23) |  |  | +8.6% (+1.9 to +18.2) | 26 (11-49) |
|  | Dislocation of hip | 2020-2023 | +10.5% (+0.0 to +22.6) | 3 (1-6) |  |  | +8.6% (+0.5 to +19.0) | 6 (2-15) |
|  | Muscle and tendon injuries, including sprains and strains lesser dislocations | 2020-2023 | +10.4% (+0.6 to +22.9) | 30 (15-50) |  |  | +8.6% (+1.2 to +18.7) | 61 (32-108) |
|  | Amputation of lower limbs, bilateral | 2020-2023 | +10.3% (+1.1 to +22.0) | 0 (0-0) |  |  | +8.6% (+1.8 to +17.6) | 0 (0-1) |
|  | Amputation of toe/toes | 2020-2023 | +10.3% (+1.2 to +22.6) | 3 (1-7) |  |  | +8.6% (+1.2 to +19.3) | 6 (2-13) |
|  | Superficial injury of any part of the body | 2020-2023 |  |  |  |  | +8.6% (+0.9 to +18.5) | 243 (115-401) |
|  | Foreign body in GI and urogenital system | 2020-2023 | +10.1% (+0.9 to +22.7) | 4 (1-9) |  |  | +8.6% (+1.6 to +19.0) | 8 (2-18) |
|  | Fracture of pelvis | 2020-2023 | +10.5% (+0.7 to +24.3) | 4 (1-10) |  |  | +8.5% (+1.3 to +18.7) | 9 (2-24) |
|  | Drowning and nonfatal submersion | 2020-2023 |  |  |  |  | +8.5% (+1.8 to +17.8) | 9 (3-25) |
|  | Contusion in any part of the body | 2020-2023 | +10.4% (+0.9 to +21.9) | 87 (47-147) |  |  | +8.5% (+1.1 to +18.4) | 196 (103-347) |
|  | Effect of different environmental factors | 2020-2023 | +10.3% (+0.2 to +23.3) | 5 (2-10) |  |  | +8.5% (+1.1 to +18.3) | 10 (4-25) |
|  | Dislocation of knee | 2020-2023 |  |  |  |  | +8.5% (+1.7 to +18.4) | 9 (3-23) |
|  | Burns, >=20% total burned surface area or >= 10% burned surface area if head/neck or hands/wrist involved w/o lower airway burns | 2020-2023 | +10.6% (+1.3 to +21.7) | 0 (0-1) |  |  | +8.4% (+0.5 to +17.7) | 1 (0-2) |
|  | Amputation of thumb | 2020-2023 |  |  |  |  | +8.4% (+0.8 to +18.6) | 8 (2-19) |
|  | Foreign body in ear | 2020-2023 | +10.1% (+0.3 to +22.7) | 5 (2-10) |  |  |  |  |
|  | Asphyxiation | 2020-2023 | +10.0% (+0.1 to +23.7) | 5 (1-12) |  |  | +8.2% (+0.6 to +18.1) | 10 (3-28) |
|  | Poisoning requiring urgent care | 2020-2023 | +10.4% (+1.2 to +22.5) | 7 (3-13) |  |  | +8.2% (+0.9 to +19.1) | 19 (9-36) |
|  | Burns, <20% total burned surface area without lower airway burns | 2020-2023 | +10.0% (+0.3 to +23.8) | 13 (5-24) |  |  | +8.2% (+0.7 to +19.3) | 29 (11-56) |

## Italy

| **Age** | **Category** | **Period** | **Male %Change** | **Male Incidence 2023** | **Female %Change** | **Female Incidence 2023** | **Both %Change** | **Both Incidence 2023** |
| --- | --- | --- | --- | --- | --- | --- | --- | --- |
| <5 years | Severe chest Injury | 2020-2023 | +84.7% (+70.4 to +100.3) | 53 (29-89) | +68.1% (+55.7 to +78.5) | 24 (12-48) | +79.1% (+66.4 to +92.3) | 77 (41-135) |
|  | Fracture of skull | 2020-2023 | +84.7% (+69.6 to +100.7) | 94 (47-158) | +68.0% (+55.3 to +78.4) | 43 (20-86) | +79.0% (+65.4 to +93.3) | 137 (67-246) |
|  | Internal hemorrhage in abdomen and pelvis | 2020-2023 | +84.6% (+70.4 to +100.0) | 69 (41-112) | +68.0% (+56.0 to +78.0) | 33 (19-59) | +78.8% (+66.2 to +92.5) | 102 (60-168) |
|  | Fracture of face bones | 2020-2023 | +84.7% (+69.8 to +98.7) | 122 (66-202) | +68.1% (+55.4 to +78.0) | 70 (35-124) | +78.3% (+65.0 to +90.1) | 192 (102-331) |
|  | Fracture of sternum and/or fracture of one or more ribs | 2020-2023 | +84.6% (+69.2 to +100.6) | 23 (10-46) | +68.0% (+55.4 to +79.4) | 14 (6-30) | +78.0% (+64.4 to +91.3) | 37 (16-74) |
|  | Moderate/Severe TBI | 2020-2023 | +85.1% (+70.6 to +101.6) | 15 (9-23) | +68.4% (+56.2 to +79.2) | 10 (6-17) | +77.8% (+64.9 to +91.4) | 25 (15-39) |
|  | Crush injury | 2020-2023 | +85.2% (+70.9 to +100.9) | 0 (0-1) | +68.4% (+55.9 to +79.6) | 0 (0-1) | +77.6% (+65.1 to +90.3) | 1 (0-1) |
|  | Amputation of upper limbs, bilateral | 2020-2023 | +85.2% (+70.7 to +101.4) | 0 (0-1) | +68.5% (+56.0 to +79.1) | 0 (0-1) | +77.5% (+64.9 to +90.9) | 1 (0-2) |
|  | Spinal cord lesion below neck level | 2020-2023 | +85.4% (+70.8 to +99.8) | 2 (1-3) | +68.6% (+56.0 to +78.8) | 1 (0-3) | +77.4% (+64.9 to +89.2) | 3 (1-6) |
|  | Fracture of hand (wrist and other distal part of hand) | 2020-2023 | +84.6% (+70.4 to +100.9) | 92 (49-158) | +68.1% (+54.6 to +80.3) | 69 (33-123) | +77.2% (+64.3 to +90.6) | 161 (84-271) |
|  | Spinal cord lesion at neck level | 2020-2023 | +85.2% (+69.9 to +99.5) | 1 (0-2) | +68.4% (+55.7 to +77.9) | 1 (0-2) | +76.9% (+63.7 to +88.6) | 2 (1-4) |
|  | Open wound(s) | 2020-2023 | +84.7% (+70.5 to +100.3) | 419 (253-632) | +68.1% (+55.5 to +78.7) | 339 (192-530) | +76.9% (+64.0 to +89.4) | 758 (451-1,156) |
|  | Amputation of fingers (excluding thumb) | 2020-2023 | +84.7% (+70.0 to +102.0) | 8 (3-17) | +68.1% (+54.6 to +79.2) | 7 (2-17) | +76.8% (+64.0 to +90.1) | 15 (5-33) |
|  | Amputation of upper limb, unilateral | 2020-2023 | +85.2% (+70.7 to +101.1) | 0 (0-1) | +68.4% (+56.2 to +79.3) | 0 (0-1) | +76.6% (+64.2 to +89.4) | 1 (0-2) |
|  | Fracture of foot bones except ankle | 2020-2023 | +84.9% (+69.3 to +101.9) | 17 (8-35) | +68.1% (+55.1 to +79.5) | 15 (6-35) | +76.6% (+63.4 to +89.8) | 32 (14-69) |
|  | Injury to eyes | 2020-2023 | +84.6% (+69.1 to +101.4) | 82 (42-140) | +67.9% (+54.9 to +78.8) | 68 (31-126) | +76.6% (+63.6 to +89.0) | 150 (74-266) |
|  | Fracture of patella, tibia or fibula, or ankle | 2020-2023 | +84.7% (+70.6 to +99.4) | 35 (17-62) | +68.2% (+56.0 to +77.6) | 31 (15-58) | +76.5% (+64.4 to +87.5) | 66 (33-120) |
|  | Minor TBI | 2020-2023 | +84.7% (+69.9 to +100.1) | 57 (30-100) | +68.1% (+56.1 to +78.7) | 51 (24-93) | +76.5% (+63.8 to +87.9) | 107 (54-191) |
|  | Nerve injury | 2020-2023 | +84.9% (+70.5 to +100.4) | 21 (9-46) | +68.2% (+55.7 to +79.2) | 20 (8-48) | +76.4% (+63.7 to +88.4) | 41 (17-92) |
|  | Multiple fractures, dislocations, crashes, wounds, pains, and strains | 2020-2023 | +84.6% (+69.4 to +100.7) | 12 (6-22) | +67.9% (+55.6 to +78.7) | 11 (5-21) | +76.3% (+63.4 to +88.6) | 23 (11-42) |
|  | Foreign body in GI and urogenital system | 2020-2023 | +84.8% (+70.1 to +100.2) | 9 (3-18) | +68.2% (+56.0 to +78.5) | 9 (2-24) | +76.0% (+63.7 to +88.6) | 17 (6-44) |
|  | Fracture of femur, other than femoral neck | 2020-2023 | +84.7% (+70.7 to +101.4) | 11 (5-21) | +68.1% (+55.1 to +78.3) | 11 (4-26) | +75.9% (+64.0 to +87.4) | 22 (9-49) |
|  | Amputation of thumb | 2020-2023 | +84.7% (+69.2 to +101.8) | 9 (3-20) | +68.3% (+54.4 to +80.8) | 9 (2-23) | +75.9% (+62.4 to +90.5) | 18 (5-43) |
|  | Foreign body in ear | 2020-2023 | +84.7% (+70.3 to +100.6) | 11 (4-25) | +68.1% (+54.8 to +79.4) | 11 (3-31) | +75.7% (+62.8 to +88.5) | 22 (7-53) |
|  | Amputation of toe/toes | 2020-2023 | +84.5% (+69.9 to +101.4) | 6 (2-14) | +68.0% (+55.6 to +79.2) | 6 (2-17) | +75.7% (+63.6 to +88.9) | 12 (4-28) |
|  | Amputation of lower limbs, bilateral | 2020-2023 | +85.3% (+71.2 to +100.3) | 0 (0-1) | +68.5% (+55.8 to +79.1) | 1 (0-1) | +75.6% (+63.3 to +87.7) | 1 (0-2) |
|  | Lower airway burns | 2020-2023 | +85.3% (+69.8 to +101.5) | 1 (0-1) | +68.3% (+55.7 to +78.7) | 1 (0-2) | +75.5% (+63.2 to +88.8) | 1 (0-3) |
|  | Fracture of vertebral column | 2020-2023 | +84.4% (+70.1 to +99.9) | 12 (5-24) | +68.0% (+56.2 to +78.2) | 13 (5-28) | +75.5% (+63.7 to +88.6) | 24 (10-49) |
|  | Dislocation of shoulder | 2020-2023 | +84.6% (+70.1 to +100.1) | 12 (5-27) | +68.1% (+56.4 to +78.8) | 14 (4-37) | +75.5% (+64.2 to +87.6) | 26 (9-66) |
|  | Dislocation of knee | 2020-2023 | +85.0% (+70.6 to +99.7) | 9 (3-22) | +68.3% (+56.1 to +79.6) | 11 (3-32) | +75.4% (+63.9 to +86.4) | 20 (6-52) |
|  | Foreign body in respiratory system | 2020-2023 | +84.7% (+70.7 to +100.5) | 6 (2-14) | +68.1% (+55.7 to +78.4) | 7 (2-18) | +75.4% (+62.7 to +86.3) | 13 (4-32) |
|  | Amputation of lower limb, unilateral | 2020-2023 | +85.0% (+70.0 to +100.6) | 0 (0-0) | +68.2% (+55.2 to +78.8) | 0 (0-0) | +75.4% (+62.4 to +87.0) | 0 (0-0) |
|  | Fracture of clavicle, scapula, or humerus | 2020-2023 | +84.6% (+69.8 to +100.5) | 35 (17-67) | +68.0% (+55.4 to +78.8) | 40 (16-83) | +75.4% (+62.1 to +87.6) | 75 (33-149) |
|  | Asphyxiation | 2020-2023 | +84.9% (+70.4 to +99.6) | 11 (4-26) | +68.0% (+56.3 to +78.6) | 13 (4-36) | +75.3% (+63.8 to +86.8) | 23 (7-60) |
|  | Fracture of radius and/or ulna | 2020-2023 | +84.6% (+69.6 to +101.1) | 25 (12-44) | +68.0% (+55.1 to +78.3) | 30 (14-57) | +75.2% (+62.0 to +86.9) | 55 (25-103) |
|  | Muscle and tendon injuries, including sprains and strains lesser dislocations | 2020-2023 | +84.6% (+69.8 to +99.3) | 63 (35-110) | +68.0% (+55.1 to +79.0) | 74 (37-132) | +75.2% (+62.7 to +87.0) | 136 (72-240) |
|  | Superficial injury of any part of the body | 2020-2023 | +84.6% (+69.9 to +100.3) | 233 (131-370) | +68.1% (+56.3 to +78.7) | 289 (162-464) | +75.1% (+62.7 to +86.5) | 522 (294-834) |
|  | Dislocation of hip | 2020-2023 | +84.5% (+70.1 to +99.0) | 6 (2-14) | +67.9% (+56.4 to +79.3) | 7 (2-19) | +75.0% (+63.0 to +87.7) | 13 (4-32) |
|  | Drowning and nonfatal submersion | 2020-2023 | +84.5% (+70.6 to +99.9) | 10 (3-22) | +67.7% (+55.5 to +78.5) | 12 (3-34) | +74.9% (+62.0 to +86.7) | 22 (6-56) |
|  | Burns, <20% total burned surface area without lower airway burns | 2020-2023 | +84.8% (+68.9 to +100.0) | 31 (14-60) | +68.2% (+55.1 to +79.9) | 42 (17-90) | +74.9% (+61.2 to +86.4) | 73 (31-151) |
|  | Effect of different environmental factors | 2020-2023 | +84.7% (+68.8 to +100.6) | 10 (4-24) | +68.0% (+55.1 to +79.9) | 14 (5-36) | +74.8% (+61.6 to +87.0) | 24 (8-59) |
|  | Contusion in any part of the body | 2020-2023 | +84.7% (+69.9 to +100.7) | 184 (104-295) | +68.1% (+55.3 to +78.2) | 253 (141-424) | +74.7% (+62.3 to +86.1) | 437 (249-717) |
|  | Complications following therapeutic procedures | 2020-2023 | +84.5% (+68.9 to +100.5) | 2 (1-4) | +68.0% (+56.1 to +77.6) | 3 (2-6) | +74.5% (+61.8 to +86.5) | 6 (3-10) |
|  | Fracture of pelvis | 2020-2023 | +84.4% (+70.2 to +100.5) | 7 (2-18) | +67.9% (+55.5 to +78.5) | 10 (3-30) | +74.3% (+62.3 to +84.8) | 18 (5-48) |
|  | Burns, >=20% total burned surface area or >= 10% burned surface area if head/neck or hands/wrist involved w/o lower airway burns | 2020-2023 | +85.2% (+70.6 to +100.7) | 1 (0-1) | +68.3% (+56.5 to +78.9) | 1 (1-3) | +74.2% (+63.0 to +84.6) | 2 (1-4) |
|  | Fracture of hip | 2020-2023 | +84.5% (+69.1 to +100.2) | 1 (0-2) | +68.0% (+55.2 to +78.2) | 2 (1-4) | +73.9% (+61.6 to +85.2) | 3 (1-6) |
|  | Poisoning requiring urgent care | 2020-2023 | +84.7% (+69.1 to +99.0) | 15 (8-27) | +68.1% (+55.8 to +79.2) | 28 (13-53) | +73.7% (+60.7 to +85.2) | 43 (22-80) |
|  | Severe chest Injury | 2010-2023 | +24.3% (+18.6 to +30.6) | 53 (29-89) | +15.4% (+9.3 to +21.6) | 24 (12-48) | +21.3% (+17.1 to +26.4) | 77 (41-135) |
|  | Fracture of skull | 2010-2023 | +24.3% (+18.3 to +31.4) | 94 (47-158) | +15.3% (+9.2 to +22.2) | 43 (20-86) | +21.2% (+16.3 to +27.1) | 137 (67-246) |
|  | Internal hemorrhage in abdomen and pelvis | 2010-2023 | +24.2% (+18.6 to +30.9) | 69 (41-112) | +15.3% (+9.3 to +21.6) | 33 (19-59) | +21.1% (+16.3 to +26.5) | 102 (60-168) |
|  | Fracture of face bones | 2010-2023 | +24.3% (+19.1 to +30.8) | 122 (66-202) | +15.4% (+9.0 to +21.7) | 70 (35-124) | +20.8% (+16.2 to +25.7) | 192 (102-331) |
|  | Fracture of sternum and/or fracture of one or more ribs | 2010-2023 | +24.3% (+18.3 to +31.2) | 23 (10-46) | +15.4% (+9.0 to +21.9) | 14 (6-30) | +20.7% (+15.5 to +26.1) | 37 (16-74) |
|  | Fracture of hand (wrist and other distal part of hand) | 2010-2023 | +24.3% (+18.4 to +31.3) | 92 (49-158) | +15.4% (+9.0 to +22.2) | 69 (33-123) | +20.3% (+15.3 to +25.9) | 161 (84-271) |
|  | Moderate/Severe TBI | 2010-2023 | +24.1% (+17.9 to +33.3) | 15 (9-23) | +15.2% (+9.0 to +22.2) | 10 (6-17) | +20.2% (+15.9 to +25.8) | 25 (15-39) |
|  | Open wound(s) | 2010-2023 | +24.3% (+18.4 to +31.3) | 419 (253-632) | +15.3% (+9.4 to +21.9) | 339 (192-530) | +20.1% (+14.9 to +25.6) | 758 (451-1,156) |
|  | Crush injury | 2010-2023 | +24.1% (+18.5 to +30.9) | 0 (0-1) | +15.1% (+8.8 to +21.9) | 0 (0-1) | +20.1% (+14.9 to +25.7) | 1 (0-1) |
|  | Injury to eyes | 2010-2023 | +24.3% (+18.1 to +30.6) | 82 (42-140) | +15.4% (+8.7 to +22.5) | 68 (31-126) | +20.1% (+14.5 to +25.8) | 150 (74-266) |
|  | Amputation of upper limbs, bilateral | 2010-2023 | +24.1% (+18.7 to +30.7) | 0 (0-1) | +15.3% (+9.5 to +22.1) | 0 (0-1) | +20.0% (+15.4 to +25.3) | 1 (0-2) |
|  | Amputation of fingers (excluding thumb) | 2010-2023 | +24.2% (+17.9 to +32.2) | 8 (3-17) | +15.3% (+9.6 to +21.9) | 7 (2-17) | +20.0% (+15.3 to +25.5) | 15 (5-33) |
|  | Fracture of foot bones except ankle | 2010-2023 | +24.4% (+18.3 to +31.3) | 17 (8-35) | +15.4% (+9.0 to +23.0) | 15 (6-35) | +20.0% (+14.5 to +25.5) | 32 (14-69) |
|  | Fracture of patella, tibia or fibula, or ankle | 2010-2023 | +24.3% (+18.8 to +30.6) | 35 (17-62) | +15.4% (+9.7 to +21.2) | 31 (15-58) | +19.9% (+15.3 to +24.8) | 66 (33-120) |
|  | Minor TBI | 2010-2023 | +24.3% (+18.9 to +30.6) | 57 (30-100) | +15.4% (+9.5 to +21.8) | 51 (24-93) | +19.9% (+15.2 to +25.4) | 107 (54-191) |
|  | Multiple fractures, dislocations, crashes, wounds, pains, and strains | 2010-2023 | +24.3% (+18.4 to +31.6) | 12 (6-22) | +15.3% (+9.1 to +21.8) | 11 (5-21) | +19.9% (+15.1 to +25.5) | 23 (11-42) |
|  | Spinal cord lesion below neck level | 2010-2023 | +24.1% (+18.5 to +30.7) | 2 (1-3) | +15.1% (+8.7 to +21.5) | 1 (0-3) | +19.8% (+15.0 to +24.5) | 3 (1-6) |
|  | Nerve injury | 2010-2023 | +24.3% (+18.5 to +31.0) | 21 (9-46) | +15.3% (+8.9 to +22.3) | 20 (8-48) | +19.8% (+14.9 to +25.0) | 41 (17-92) |
|  | Spinal cord lesion at neck level | 2010-2023 | +24.1% (+17.8 to +31.0) | 1 (0-2) | +15.1% (+8.5 to +22.6) | 1 (0-2) | +19.7% (+14.7 to +25.4) | 2 (1-4) |
|  | Fracture of femur, other than femoral neck | 2010-2023 | +24.3% (+18.7 to +31.4) | 11 (5-21) | +15.4% (+9.3 to +21.6) | 11 (4-26) | +19.6% (+15.1 to +24.6) | 22 (9-49) |
|  | Foreign body in GI and urogenital system | 2010-2023 | +24.3% (+18.8 to +32.0) | 9 (3-18) | +15.3% (+9.4 to +22.7) | 9 (2-24) | +19.6% (+14.3 to +25.5) | 17 (6-44) |
|  | Amputation of upper limb, unilateral | 2010-2023 | +24.1% (+18.1 to +31.2) | 0 (0-1) | +15.1% (+9.3 to +22.4) | 0 (0-1) | +19.6% (+14.8 to +24.7) | 1 (0-2) |
|  | Fracture of vertebral column | 2010-2023 | +24.3% (+18.5 to +31.1) | 12 (5-24) | +15.3% (+9.7 to +21.5) | 13 (5-28) | +19.5% (+14.9 to +24.6) | 24 (10-49) |
|  | Amputation of toe/toes | 2010-2023 | +24.3% (+17.7 to +31.9) | 6 (2-14) | +15.3% (+9.1 to +22.0) | 6 (2-17) | +19.5% (+13.9 to +25.2) | 12 (4-28) |
|  | Amputation of thumb | 2010-2023 | +24.3% (+18.1 to +32.0) | 9 (3-20) | +15.3% (+8.1 to +22.9) | 9 (2-23) | +19.5% (+14.9 to +25.1) | 18 (5-43) |
|  | Foreign body in ear | 2010-2023 | +24.3% (+18.2 to +32.1) | 11 (4-25) | +15.3% (+8.9 to +21.6) | 11 (3-31) | +19.5% (+14.6 to +24.6) | 22 (7-53) |
|  | Dislocation of shoulder | 2010-2023 | +24.3% (+18.7 to +31.4) | 12 (5-27) | +15.4% (+9.6 to +21.7) | 14 (4-37) | +19.5% (+14.9 to +24.9) | 26 (9-66) |
|  | Fracture of clavicle, scapula, or humerus | 2010-2023 | +24.3% (+18.4 to +30.6) | 35 (17-67) | +15.4% (+9.0 to +22.3) | 40 (16-83) | +19.4% (+14.6 to +24.9) | 75 (33-149) |
|  | Foreign body in respiratory system | 2010-2023 | +24.3% (+18.8 to +30.5) | 6 (2-14) | +15.3% (+9.3 to +22.2) | 7 (2-18) | +19.4% (+14.8 to +24.6) | 13 (4-32) |
|  | Fracture of radius and/or ulna | 2010-2023 | +24.3% (+18.3 to +31.1) | 25 (12-44) | +15.4% (+9.0 to +22.4) | 30 (14-57) | +19.4% (+14.3 to +24.7) | 55 (25-103) |
|  | Asphyxiation | 2010-2023 | +24.4% (+19.1 to +30.9) | 11 (4-26) | +15.3% (+9.5 to +21.7) | 13 (4-36) | +19.3% (+14.6 to +24.2) | 23 (7-60) |
|  | Dislocation of knee | 2010-2023 | +24.4% (+19.0 to +30.2) | 9 (3-22) | +15.4% (+9.8 to +21.4) | 11 (3-32) | +19.3% (+14.7 to +24.0) | 20 (6-52) |
|  | Muscle and tendon injuries, including sprains and strains lesser dislocations | 2010-2023 | +24.3% (+18.7 to +30.6) | 63 (35-110) | +15.3% (+9.0 to +22.0) | 74 (37-132) | +19.3% (+14.3 to +24.3) | 136 (72-240) |
|  | Superficial injury of any part of the body | 2010-2023 | +24.3% (+18.9 to +31.1) | 233 (131-370) | +15.4% (+9.2 to +22.2) | 289 (162-464) | +19.3% (+14.6 to +24.4) | 522 (294-834) |
|  | Dislocation of hip | 2010-2023 | +24.3% (+18.8 to +31.5) | 6 (2-14) | +15.3% (+9.3 to +21.5) | 7 (2-19) | +19.2% (+14.5 to +24.5) | 13 (4-32) |
|  | Drowning and nonfatal submersion | 2010-2023 | +24.2% (+18.9 to +30.6) | 10 (3-22) | +15.3% (+9.8 to +22.8) | 12 (3-34) | +19.2% (+14.6 to +23.8) | 22 (6-56) |
|  | Amputation of lower limb, unilateral | 2010-2023 | +24.2% (+18.4 to +31.5) | 0 (0-0) | +15.3% (+9.4 to +22.1) | 0 (0-0) | +19.2% (+14.3 to +24.8) | 0 (0-0) |
|  | Effect of different environmental factors | 2010-2023 | +24.3% (+17.7 to +32.1) | 10 (4-24) | +15.4% (+9.6 to +22.9) | 14 (5-36) | +19.1% (+13.7 to +24.6) | 24 (8-59) |
|  | Lower airway burns | 2010-2023 | +24.1% (+17.1 to +30.5) | 1 (0-1) | +15.2% (+9.1 to +21.7) | 1 (0-2) | +19.0% (+14.2 to +24.4) | 1 (0-3) |
|  | Contusion in any part of the body | 2010-2023 | +24.3% (+18.9 to +30.3) | 184 (104-295) | +15.3% (+9.0 to +21.7) | 253 (141-424) | +19.0% (+14.2 to +24.0) | 437 (249-717) |
|  | Burns, <20% total burned surface area without lower airway burns | 2010-2023 | +24.3% (+18.2 to +31.2) | 31 (14-60) | +15.3% (+8.6 to +22.2) | 42 (17-90) | +19.0% (+13.9 to +24.4) | 73 (31-151) |
|  | Complications following therapeutic procedures | 2010-2023 | +24.3% (+18.4 to +30.7) | 2 (1-4) | +15.4% (+9.6 to +21.8) | 3 (2-6) | +19.0% (+14.2 to +24.0) | 6 (3-10) |
|  | Amputation of lower limbs, bilateral | 2010-2023 | +24.1% (+18.1 to +31.7) | 0 (0-1) | +15.1% (+8.9 to +21.6) | 1 (0-1) | +19.0% (+14.2 to +24.4) | 1 (0-2) |
|  | Fracture of pelvis | 2010-2023 | +24.3% (+18.3 to +31.2) | 7 (2-18) | +15.3% (+9.6 to +21.6) | 10 (3-30) | +18.9% (+14.3 to +23.8) | 18 (5-48) |
|  | Fracture of hip | 2010-2023 | +24.4% (+18.9 to +31.2) | 1 (0-2) | +15.5% (+9.5 to +22.2) | 2 (1-4) | +18.8% (+14.2 to +23.8) | 3 (1-6) |
|  | Poisoning requiring urgent care | 2010-2023 | +24.3% (+18.5 to +30.8) | 15 (8-27) | +15.3% (+9.1 to +22.1) | 28 (13-53) | +18.5% (+13.3 to +23.3) | 43 (22-80) |
|  | Burns, >=20% total burned surface area or >= 10% burned surface area if head/neck or hands/wrist involved w/o lower airway burns | 2010-2023 | +24.1% (+18.7 to +30.0) | 1 (0-1) | +15.1% (+9.2 to +21.7) | 1 (1-3) | +18.4% (+13.4 to +23.3) | 2 (1-4) |
| 5-14 years | Fracture of skull | 2020-2023 | +29.7% (+13.6 to +47.8) | 178 (82-310) | +23.9% (+6.8 to +39.7) | 54 (21-105) | +28.2% (+12.1 to +45.0) | 232 (108-402) |
|  | Severe chest Injury | 2020-2023 | +29.6% (+13.9 to +46.2) | 104 (49-191) | +23.9% (+6.8 to +39.9) | 31 (14-60) | +28.2% (+12.9 to +43.7) | 135 (63-253) |
|  | Internal hemorrhage in abdomen and pelvis | 2020-2023 | +29.6% (+14.6 to +46.1) | 134 (68-216) | +23.9% (+7.2 to +39.5) | 43 (19-74) | +28.2% (+13.6 to +43.6) | 176 (89-285) |
|  | Fracture of face bones | 2020-2023 | +29.6% (+12.8 to +46.9) | 237 (113-383) | +23.9% (+7.2 to +40.1) | 89 (39-163) | +28.0% (+12.3 to +43.5) | 327 (153-543) |
|  | Fracture of sternum and/or fracture of one or more ribs | 2020-2023 | +29.4% (+14.8 to +45.6) | 49 (19-98) | +23.7% (+7.2 to +38.9) | 20 (6-49) | +27.7% (+13.0 to +43.1) | 69 (27-148) |
|  | Open wound(s) | 2020-2023 | +29.7% (+13.8 to +45.2) | 836 (469-1,385) | +24.0% (+6.3 to +40.2) | 446 (231-746) | +27.6% (+10.8 to +42.3) | 1,283 (707-2,095) |
|  | Amputation of fingers (excluding thumb) | 2020-2023 | +29.7% (+12.5 to +47.7) | 16 (6-34) | +24.0% (+8.1 to +40.8) | 9 (3-21) | +27.6% (+11.8 to +43.5) | 25 (9-54) |
|  | Spinal cord lesion below neck level | 2020-2023 | +29.6% (+14.8 to +45.2) | 3 (1-6) | +24.1% (+6.5 to +40.1) | 2 (0-4) | +27.6% (+12.8 to +42.3) | 4 (1-10) |
|  | Fracture of hand (wrist and other distal part of hand) | 2020-2023 | +29.5% (+13.2 to +46.8) | 181 (86-326) | +23.8% (+6.5 to +40.5) | 89 (38-181) | +27.6% (+10.3 to +43.5) | 270 (124-493) |
|  | Fracture of foot bones except ankle | 2020-2023 | +29.8% (+13.0 to +47.9) | 32 (14-69) | +24.0% (+6.5 to +41.2) | 19 (6-45) | +27.6% (+10.5 to +44.5) | 51 (22-116) |
|  | Nerve injury | 2020-2023 | +29.8% (+12.9 to +45.2) | 40 (16-82) | +24.0% (+6.3 to +39.8) | 25 (9-62) | +27.5% (+11.9 to +42.6) | 64 (24-139) |
|  | Injury to eyes | 2020-2023 | +29.6% (+15.0 to +45.7) | 158 (77-276) | +23.9% (+6.9 to +40.3) | 87 (38-170) | +27.5% (+12.6 to +42.1) | 245 (115-436) |
|  | Asphyxiation | 2020-2023 | +30.1% (+14.0 to +46.2) | 19 (6-49) | +24.4% (+6.3 to +38.8) | 15 (4-48) | +27.5% (+11.8 to +42.1) | 35 (9-96) |
|  | Foreign body in GI and urogenital system | 2020-2023 | +29.8% (+12.9 to +46.7) | 16 (6-40) | +24.2% (+6.0 to +40.0) | 11 (3-30) | +27.4% (+10.0 to +43.0) | 28 (9-71) |
|  | Foreign body in ear | 2020-2023 | +29.9% (+14.8 to +47.1) | 19 (6-45) | +24.2% (+6.7 to +40.7) | 14 (3-37) | +27.4% (+11.6 to +44.3) | 33 (10-82) |
|  | Crush injury | 2020-2023 | +29.3% (+13.4 to +47.1) | 1 (0-2) | +23.8% (+6.4 to +39.2) | 0 (0-1) | +27.4% (+12.1 to +43.3) | 1 (0-3) |
|  | Minor TBI | 2020-2023 | +29.5% (+14.4 to +46.0) | 118 (59-217) | +24.0% (+6.6 to +40.3) | 69 (31-134) | +27.4% (+11.0 to +42.8) | 187 (90-357) |
|  | Fracture of patella, tibia or fibula, or ankle | 2020-2023 | +29.5% (+13.6 to +45.5) | 70 (35-126) | +23.9% (+6.9 to +39.1) | 41 (18-79) | +27.4% (+11.7 to +42.5) | 111 (52-199) |
|  | Amputation of thumb | 2020-2023 | +29.8% (+14.1 to +47.3) | 16 (5-37) | +24.0% (+6.8 to +40.1) | 11 (3-34) | +27.3% (+10.8 to +44.0) | 27 (8-69) |
|  | Dislocation of knee | 2020-2023 | +29.9% (+12.9 to +47.8) | 17 (6-40) | +24.3% (+7.5 to +41.1) | 14 (4-38) | +27.3% (+11.2 to +43.0) | 31 (10-77) |
|  | Burns, <20% total burned surface area without lower airway burns | 2020-2023 | +30.1% (+13.6 to +46.9) | 53 (23-100) | +24.3% (+5.8 to +40.0) | 48 (18-108) | +27.3% (+10.7 to +42.8) | 101 (41-199) |
|  | Amputation of toe/toes | 2020-2023 | +29.7% (+14.8 to +46.4) | 12 (4-27) | +24.0% (+5.9 to +38.2) | 8 (2-22) | +27.3% (+11.2 to +42.4) | 20 (6-48) |
|  | Fracture of femur, other than femoral neck | 2020-2023 | +29.6% (+13.0 to +44.6) | 22 (10-47) | +24.0% (+6.8 to +38.6) | 15 (6-34) | +27.3% (+9.9 to +43.5) | 37 (16-80) |
|  | Amputation of upper limbs, bilateral | 2020-2023 | +29.2% (+13.8 to +46.4) | 1 (0-2) | +23.6% (+6.6 to +39.6) | 0 (0-1) | +27.3% (+11.7 to +43.4) | 1 (0-3) |
|  | Drowning and nonfatal submersion | 2020-2023 | +30.1% (+15.3 to +47.9) | 18 (5-42) | +23.9% (+7.1 to +40.1) | 15 (3-43) | +27.2% (+12.2 to +43.8) | 32 (9-83) |
|  | Moderate/Severe TBI | 2020-2023 | +28.9% (+13.3 to +44.9) | 31 (17-51) | +23.5% (+6.2 to +38.5) | 14 (8-25) | +27.2% (+12.3 to +42.3) | 46 (25-78) |
|  | Fracture of clavicle, scapula, or humerus | 2020-2023 | +29.6% (+13.3 to +44.8) | 71 (32-137) | +24.0% (+6.1 to +40.5) | 54 (21-114) | +27.1% (+11.0 to +42.7) | 125 (53-252) |
|  | Multiple fractures, dislocations, crashes, wounds, pains, and strains | 2020-2023 | +29.4% (+14.7 to +45.6) | 25 (12-48) | +23.6% (+6.6 to +38.5) | 15 (6-29) | +27.1% (+11.9 to +41.8) | 40 (18-76) |
|  | Dislocation of shoulder | 2020-2023 | +29.7% (+14.2 to +46.0) | 25 (9-61) | +23.8% (+7.2 to +39.2) | 19 (5-52) | +27.1% (+11.5 to +41.1) | 44 (14-112) |
|  | Muscle and tendon injuries, including sprains and strains lesser dislocations | 2020-2023 | +29.6% (+13.9 to +45.4) | 123 (63-221) | +23.9% (+6.3 to +39.6) | 95 (46-178) | +27.0% (+9.6 to +42.3) | 218 (113-400) |
|  | Superficial injury of any part of the body | 2020-2023 | +29.6% (+13.4 to +46.8) | 471 (234-802) | +24.0% (+5.6 to +41.0) | 387 (171-701) | +27.0% (+10.0 to +43.2) | 858 (405-1,458) |
|  | Amputation of upper limb, unilateral | 2020-2023 | +29.2% (+11.9 to +45.5) | 1 (0-2) | +23.7% (+5.8 to +40.1) | 0 (0-1) | +27.0% (+11.0 to +43.2) | 1 (0-3) |
|  | Spinal cord lesion at neck level | 2020-2023 | +29.0% (+12.4 to +44.5) | 2 (1-4) | +23.7% (+6.4 to +39.5) | 1 (0-2) | +27.0% (+11.4 to +42.6) | 3 (1-6) |
|  | Fracture of radius and/or ulna | 2020-2023 | +29.5% (+12.4 to +44.9) | 52 (23-95) | +23.8% (+6.5 to +39.7) | 40 (16-79) | +27.0% (+9.8 to +41.6) | 92 (39-172) |
|  | Effect of different environmental factors | 2020-2023 | +29.7% (+13.7 to +47.3) | 19 (7-44) | +24.0% (+6.8 to +40.0) | 17 (6-43) | +26.9% (+11.0 to +43.3) | 36 (13-85) |
|  | Foreign body in respiratory system | 2020-2023 | +29.4% (+13.9 to +45.8) | 12 (4-26) | +23.8% (+6.6 to +39.4) | 9 (3-24) | +26.9% (+10.5 to +42.2) | 21 (7-49) |
|  | Amputation of lower limbs, bilateral | 2020-2023 | +29.5% (+13.7 to +45.0) | 1 (0-2) | +23.8% (+5.6 to +39.1) | 1 (0-2) | +26.9% (+11.0 to +41.8) | 1 (0-4) |
|  | Lower airway burns | 2020-2023 | +29.4% (+12.8 to +48.0) | 1 (0-2) | +23.8% (+7.2 to +38.5) | 1 (0-2) | +26.8% (+10.7 to +43.0) | 2 (0-4) |
|  | Dislocation of hip | 2020-2023 | +29.5% (+13.7 to +48.2) | 12 (4-28) | +23.6% (+7.0 to +41.3) | 9 (2-25) | +26.8% (+11.1 to +45.3) | 21 (6-51) |
|  | Contusion in any part of the body | 2020-2023 | +29.6% (+13.3 to +45.6) | 360 (194-615) | +23.9% (+7.2 to +40.0) | 328 (164-583) | +26.8% (+10.4 to +42.2) | 689 (361-1,180) |
|  | Fracture of vertebral column | 2020-2023 | +29.2% (+14.2 to +44.6) | 24 (11-52) | +23.4% (+5.9 to +40.0) | 17 (7-39) | +26.7% (+11.2 to +42.4) | 42 (18-88) |
|  | Fracture of pelvis | 2020-2023 | +29.2% (+12.5 to +45.5) | 16 (5-40) | +23.6% (+6.4 to +40.4) | 15 (4-43) | +26.4% (+9.6 to +41.8) | 31 (9-83) |
|  | Burns, >=20% total burned surface area or >= 10% burned surface area if head/neck or hands/wrist involved w/o lower airway burns | 2020-2023 | +29.4% (+12.9 to +46.7) | 2 (1-3) | +23.8% (+5.7 to +39.2) | 2 (1-3) | +26.4% (+10.1 to +42.1) | 3 (1-7) |
|  | Poisoning requiring urgent care | 2020-2023 | +29.6% (+13.2 to +47.0) | 30 (14-56) | +23.9% (+6.2 to +40.0) | 36 (17-72) | +26.4% (+8.6 to +42.3) | 66 (31-129) |
|  | Complications following therapeutic procedures | 2020-2023 | +29.0% (+13.9 to +44.2) | 5 (3-10) | +23.4% (+6.0 to +38.6) | 5 (2-10) | +26.2% (+9.3 to +40.6) | 11 (5-19) |
|  | Amputation of lower limb, unilateral | 2020-2023 | +28.0% (+12.0 to +44.2) | 0 (0-0) | +22.9% (+4.8 to +39.2) | 0 (0-0) | +25.7% (+8.7 to +42.5) | 0 (0-0) |
|  | Fracture of hip | 2020-2023 | +28.2% (+12.1 to +44.5) | 3 (1-7) | +22.9% (+5.4 to +38.6) | 3 (1-7) | +25.4% (+8.8 to +40.1) | 6 (3-14) |
|  | Severe chest Injury | 2010-2023 | +9.2% (+3.9 to +13.7) | 104 (49-191) | +6.2% (+1.9 to +10.4) | 31 (14-60) | +8.4% (+3.9 to +12.2) | 135 (63-253) |
|  | Fracture of skull | 2010-2023 | +9.2% (+4.0 to +13.8) | 178 (82-310) | +6.1% (+1.5 to +10.7) | 54 (21-105) | +8.4% (+3.9 to +11.9) | 232 (108-402) |
|  | Internal hemorrhage in abdomen and pelvis | 2010-2023 | +9.2% (+4.3 to +13.5) | 134 (68-216) | +6.2% (+1.7 to +10.5) | 43 (19-74) | +8.4% (+4.1 to +12.1) | 176 (89-285) |
|  | Moderate/Severe TBI | 2010-2023 | +9.3% (+4.3 to +14.0) | 31 (17-51) | +6.6% (+2.0 to +11.0) | 14 (8-25) | +8.4% (+4.4 to +11.9) | 46 (25-78) |
|  | Crush injury | 2010-2023 | +9.3% (+4.0 to +14.3) | 1 (0-2) | +6.5% (+2.0 to +11.1) | 0 (0-1) | +8.4% (+4.3 to +11.9) | 1 (0-3) |
|  | Amputation of lower limb, unilateral | 2010-2023 | +9.3% (+4.2 to +14.7) | 0 (0-0) | +7.2% (+2.6 to +12.2) | 0 (0-0) | +8.4% (+4.4 to +12.2) | 0 (0-0) |
|  | Fracture of face bones | 2010-2023 | +9.2% (+4.2 to +14.1) | 237 (113-383) | +6.2% (+1.5 to +10.3) | 89 (39-163) | +8.3% (+4.1 to +11.6) | 327 (153-543) |
|  | Fracture of sternum and/or fracture of one or more ribs | 2010-2023 | +9.1% (+4.3 to +14.1) | 49 (19-98) | +6.3% (+1.4 to +10.9) | 20 (6-49) | +8.3% (+4.2 to +11.7) | 69 (27-148) |
|  | Spinal cord lesion at neck level | 2010-2023 | +9.2% (+4.1 to +13.9) | 2 (1-4) | +6.5% (+2.2 to +11.2) | 1 (0-2) | +8.2% (+3.9 to +11.8) | 3 (1-6) |
|  | Amputation of upper limbs, bilateral | 2010-2023 | +9.1% (+4.0 to +14.4) | 1 (0-2) | +6.4% (+1.8 to +11.4) | 0 (0-1) | +8.2% (+4.1 to +11.8) | 1 (0-3) |
|  | Spinal cord lesion below neck level | 2010-2023 | +9.2% (+4.2 to +13.6) | 3 (1-6) | +6.3% (+1.6 to +11.0) | 2 (0-4) | +8.1% (+4.0 to +11.6) | 4 (1-10) |
|  | Fracture of hand (wrist and other distal part of hand) | 2010-2023 | +9.2% (+3.6 to +13.7) | 181 (86-326) | +6.2% (+1.6 to +10.6) | 89 (38-181) | +8.1% (+3.9 to +11.6) | 270 (124-493) |
|  | Open wound(s) | 2010-2023 | +9.2% (+4.2 to +13.8) | 836 (469-1,385) | +6.2% (+1.6 to +11.0) | 446 (231-746) | +8.1% (+4.0 to +11.6) | 1,283 (707-2,095) |
|  | Minor TBI | 2010-2023 | +9.2% (+4.3 to +13.8) | 118 (59-217) | +6.3% (+1.6 to +10.8) | 69 (31-134) | +8.1% (+4.0 to +11.4) | 187 (90-357) |
|  | Fracture of hip | 2010-2023 | +9.3% (+4.4 to +14.6) | 3 (1-7) | +6.9% (+2.5 to +11.5) | 3 (1-7) | +8.1% (+4.4 to +11.8) | 6 (3-14) |
|  | Multiple fractures, dislocations, crashes, wounds, pains, and strains | 2010-2023 | +9.2% (+4.1 to +13.7) | 25 (12-48) | +6.3% (+1.5 to +11.0) | 15 (6-29) | +8.1% (+4.1 to +11.4) | 40 (18-76) |
|  | Amputation of upper limb, unilateral | 2010-2023 | +9.2% (+4.1 to +14.3) | 1 (0-2) | +6.4% (+1.6 to +11.7) | 0 (0-1) | +8.1% (+4.0 to +11.9) | 1 (0-3) |
|  | Fracture of patella, tibia or fibula, or ankle | 2010-2023 | +9.2% (+4.2 to +13.8) | 70 (35-126) | +6.3% (+1.6 to +10.9) | 41 (18-79) | +8.1% (+3.7 to +11.3) | 111 (52-199) |
|  | Amputation of fingers (excluding thumb) | 2010-2023 | +9.2% (+3.6 to +13.9) | 16 (6-34) | +6.1% (+1.7 to +10.5) | 9 (3-21) | +8.0% (+4.1 to +11.3) | 25 (9-54) |
|  | Injury to eyes | 2010-2023 | +9.2% (+4.2 to +14.0) | 158 (77-276) | +6.2% (+1.5 to +10.4) | 87 (38-170) | +8.0% (+4.3 to +11.4) | 245 (115-436) |
|  | Fracture of femur, other than femoral neck | 2010-2023 | +9.2% (+3.8 to +14.2) | 22 (10-47) | +6.3% (+2.1 to +10.4) | 15 (6-34) | +8.0% (+3.7 to +11.1) | 37 (16-80) |
|  | Amputation of toe/toes | 2010-2023 | +9.2% (+3.8 to +13.8) | 12 (4-27) | +6.3% (+1.6 to +11.3) | 8 (2-22) | +8.0% (+3.7 to +11.0) | 20 (6-48) |
|  | Fracture of foot bones except ankle | 2010-2023 | +9.1% (+3.8 to +14.1) | 32 (14-69) | +6.1% (+0.8 to +10.6) | 19 (6-45) | +8.0% (+3.9 to +11.5) | 51 (22-116) |
|  | Foreign body in GI and urogenital system | 2010-2023 | +9.2% (+3.9 to +14.3) | 16 (6-40) | +6.3% (+1.4 to +11.4) | 11 (3-30) | +8.0% (+3.9 to +11.7) | 28 (9-71) |
|  | Complications following therapeutic procedures | 2010-2023 | +9.3% (+4.2 to +14.1) | 5 (3-10) | +6.6% (+2.0 to +10.8) | 5 (2-10) | +8.0% (+4.4 to +11.4) | 11 (5-19) |
|  | Nerve injury | 2010-2023 | +9.2% (+4.4 to +13.8) | 40 (16-82) | +6.0% (+1.6 to +11.2) | 25 (9-62) | +7.9% (+4.3 to +11.2) | 64 (24-139) |
|  | Fracture of vertebral column | 2010-2023 | +9.2% (+3.8 to +13.7) | 24 (11-52) | +6.2% (+1.7 to +11.0) | 17 (7-39) | +7.9% (+4.1 to +11.2) | 42 (18-88) |
|  | Foreign body in respiratory system | 2010-2023 | +9.2% (+4.0 to +14.1) | 12 (4-26) | +6.3% (+1.8 to +10.4) | 9 (3-24) | +7.9% (+4.1 to +11.2) | 21 (7-49) |
|  | Fracture of clavicle, scapula, or humerus | 2010-2023 | +9.2% (+3.7 to +14.0) | 71 (32-137) | +6.3% (+1.9 to +11.0) | 54 (21-114) | +7.9% (+3.5 to +11.5) | 125 (53-252) |
|  | Fracture of radius and/or ulna | 2010-2023 | +9.2% (+3.6 to +13.9) | 52 (23-95) | +6.3% (+2.0 to +10.9) | 40 (16-79) | +7.9% (+4.2 to +11.2) | 92 (39-172) |
|  | Dislocation of shoulder | 2010-2023 | +9.2% (+3.7 to +14.3) | 25 (9-61) | +6.2% (+1.9 to +10.4) | 19 (5-52) | +7.9% (+4.1 to +11.2) | 44 (14-112) |
|  | Lower airway burns | 2010-2023 | +9.2% (+3.8 to +13.9) | 1 (0-2) | +6.3% (+1.3 to +11.1) | 1 (0-2) | +7.9% (+3.8 to +11.5) | 2 (0-4) |
|  | Amputation of lower limbs, bilateral | 2010-2023 | +9.2% (+4.1 to +13.5) | 1 (0-2) | +6.3% (+2.0 to +10.5) | 1 (0-2) | +7.9% (+4.0 to +11.0) | 1 (0-4) |
|  | Fracture of pelvis | 2010-2023 | +9.2% (+3.6 to +14.4) | 16 (5-40) | +6.4% (+1.8 to +11.4) | 15 (4-43) | +7.8% (+3.8 to +11.9) | 31 (9-83) |
|  | Amputation of thumb | 2010-2023 | +9.2% (+3.7 to +14.4) | 16 (5-37) | +6.1% (+1.3 to +10.8) | 11 (3-34) | +7.8% (+3.6 to +11.3) | 27 (8-69) |
|  | Muscle and tendon injuries, including sprains and strains lesser dislocations | 2010-2023 | +9.2% (+4.1 to +13.8) | 123 (63-221) | +6.2% (+1.8 to +10.7) | 95 (46-178) | +7.8% (+3.9 to +11.2) | 218 (113-400) |
|  | Superficial injury of any part of the body | 2010-2023 | +9.2% (+4.2 to +14.1) | 471 (234-802) | +6.2% (+1.5 to +10.8) | 387 (171-701) | +7.8% (+3.7 to +11.2) | 858 (405-1,458) |
|  | Asphyxiation | 2010-2023 | +9.2% (+4.2 to +14.1) | 19 (6-49) | +6.1% (+1.8 to +10.5) | 15 (4-48) | +7.8% (+4.2 to +10.8) | 35 (9-96) |
|  | Foreign body in ear | 2010-2023 | +9.1% (+3.8 to +14.3) | 19 (6-45) | +6.0% (+1.4 to +11.1) | 14 (3-37) | +7.8% (+4.1 to +11.3) | 33 (10-82) |
|  | Drowning and nonfatal submersion | 2010-2023 | +9.2% (+4.3 to +13.7) | 18 (5-42) | +6.0% (+1.2 to +10.3) | 15 (3-43) | +7.8% (+4.0 to +11.1) | 32 (9-83) |
|  | Contusion in any part of the body | 2010-2023 | +9.2% (+4.2 to +14.2) | 360 (194-615) | +6.2% (+2.1 to +10.7) | 328 (164-583) | +7.7% (+3.5 to +11.3) | 689 (361-1,180) |
|  | Dislocation of hip | 2010-2023 | +9.1% (+4.1 to +13.7) | 12 (4-28) | +6.1% (+1.7 to +11.1) | 9 (2-25) | +7.7% (+3.5 to +11.2) | 21 (6-51) |
|  | Burns, >=20% total burned surface area or >= 10% burned surface area if head/neck or hands/wrist involved w/o lower airway burns | 2010-2023 | +9.2% (+4.2 to +13.6) | 2 (1-3) | +6.4% (+1.4 to +11.4) | 2 (1-3) | +7.7% (+3.9 to +11.1) | 3 (1-7) |
|  | Dislocation of knee | 2010-2023 | +9.1% (+4.1 to +13.8) | 17 (6-40) | +6.1% (+1.1 to +11.3) | 14 (4-38) | +7.7% (+4.0 to +11.0) | 31 (10-77) |
|  | Effect of different environmental factors | 2010-2023 | +9.2% (+4.0 to +14.3) | 19 (7-44) | +6.1% (+1.5 to +11.2) | 17 (6-43) | +7.7% (+3.8 to +11.0) | 36 (13-85) |
|  | Burns, <20% total burned surface area without lower airway burns | 2010-2023 | +9.1% (+3.9 to +13.7) | 53 (23-100) | +5.9% (+1.3 to +11.2) | 48 (18-108) | +7.6% (+3.7 to +10.8) | 101 (41-199) |
|  | Poisoning requiring urgent care | 2010-2023 | +9.2% (+4.3 to +13.9) | 30 (14-56) | +6.2% (+1.6 to +10.7) | 36 (17-72) | +7.6% (+3.5 to +10.8) | 66 (31-129) |
| <20 years | Burns, <20% total burned surface area without lower airway burns | 2020-2023 | +27.4% (+17.1 to +39.1) | 122 (57-242) | +31.2% (+18.3 to +43.9) | 111 (48-234) | +29.2% (+17.6 to +41.2) | 233 (106-473) |
|  | Foreign body in ear | 2020-2023 | +26.7% (+15.1 to +39.2) | 43 (15-95) | +30.8% (+18.7 to +43.9) | 31 (8-81) | +28.4% (+16.9 to +41.0) | 75 (24-176) |
|  | Asphyxiation | 2020-2023 | +26.7% (+15.4 to +37.8) | 44 (15-105) | +30.6% (+19.2 to +42.3) | 35 (10-107) | +28.4% (+16.9 to +39.3) | 79 (24-204) |
|  | Drowning and nonfatal submersion | 2020-2023 | +26.6% (+15.7 to +38.3) | 41 (13-92) | +30.3% (+19.1 to +43.0) | 33 (9-95) | +28.3% (+17.6 to +40.3) | 74 (22-188) |
|  | Dislocation of knee | 2020-2023 | +26.1% (+15.3 to +38.1) | 39 (13-85) | +30.2% (+18.9 to +42.6) | 32 (9-90) | +27.9% (+17.2 to +39.5) | 71 (23-173) |
|  | Effect of different environmental factors | 2020-2023 | +26.0% (+15.3 to +39.3) | 44 (17-92) | +30.1% (+18.5 to +43.1) | 39 (14-100) | +27.9% (+16.5 to +40.4) | 82 (33-185) |
|  | Amputation of thumb | 2020-2023 | +25.9% (+15.2 to +38.1) | 37 (13-88) | +30.1% (+17.7 to +43.9) | 26 (8-70) | +27.6% (+16.2 to +39.5) | 62 (22-156) |
|  | Nerve injury | 2020-2023 | +25.9% (+14.9 to +37.5) | 91 (42-190) | +30.0% (+18.1 to +42.4) | 57 (23-134) | +27.5% (+16.0 to +39.2) | 148 (63-321) |
|  | Fracture of foot bones except ankle | 2020-2023 | +25.8% (+14.4 to +38.5) | 74 (35-144) | +29.9% (+16.9 to +42.6) | 43 (17-94) | +27.3% (+15.4 to +40.1) | 118 (54-244) |
|  | Foreign body in GI and urogenital system | 2020-2023 | +25.5% (+14.8 to +37.1) | 38 (15-85) | +29.9% (+17.7 to +41.8) | 25 (7-70) | +27.2% (+15.8 to +39.0) | 63 (22-156) |
|  | Poisoning requiring urgent care | 2020-2023 | +24.8% (+13.5 to +35.7) | 70 (39-126) | +29.1% (+16.5 to +41.5) | 83 (42-155) | +27.1% (+15.2 to +38.7) | 153 (81-279) |
|  | Contusion in any part of the body | 2020-2023 | +25.1% (+14.6 to +36.2) | 830 (476-1,313) | +29.2% (+17.3 to +42.0) | 747 (413-1,217) | +27.0% (+16.3 to +38.7) | 1,576 (906-2,486) |
|  | Muscle and tendon injuries, including sprains and strains lesser dislocations | 2020-2023 | +25.1% (+14.1 to +36.3) | 282 (168-455) | +29.4% (+16.4 to +42.2) | 217 (114-372) | +26.9% (+15.6 to +38.6) | 499 (287-817) |
|  | Dislocation of hip | 2020-2023 | +25.1% (+13.4 to +38.1) | 27 (9-63) | +29.2% (+17.9 to +42.4) | 21 (5-56) | +26.9% (+15.5 to +39.9) | 48 (15-121) |
|  | Injury to eyes | 2020-2023 | +25.4% (+14.6 to +37.0) | 362 (189-571) | +29.6% (+17.2 to +42.2) | 198 (94-362) | +26.8% (+16.8 to +39.1) | 561 (286-912) |
|  | Amputation of fingers (excluding thumb) | 2020-2023 | +25.3% (+13.8 to +38.2) | 37 (16-73) | +29.5% (+17.7 to +42.8) | 20 (6-46) | +26.7% (+16.4 to +39.7) | 57 (22-116) |
|  | Amputation of toe/toes | 2020-2023 | +25.0% (+13.4 to +37.4) | 27 (10-61) | +29.2% (+17.4 to +41.5) | 19 (5-47) | +26.7% (+14.7 to +38.6) | 46 (15-102) |
|  | Lower airway burns | 2020-2023 | +24.5% (+12.9 to +37.6) | 2 (1-5) | +29.5% (+18.3 to +41.9) | 2 (0-5) | +26.7% (+16.0 to +39.0) | 4 (1-10) |
|  | Superficial injury of any part of the body | 2020-2023 | +24.7% (+13.8 to +37.3) | 1,084 (634-1,667) | +29.0% (+17.1 to +41.5) | 874 (475-1,390) | +26.6% (+15.4 to +37.8) | 1,958 (1,119-3,010) |
|  | Amputation of lower limbs, bilateral | 2020-2023 | +24.4% (+13.3 to +36.2) | 2 (1-4) | +29.4% (+18.2 to +41.4) | 2 (0-5) | +26.5% (+15.7 to +37.8) | 3 (1-9) |
|  | Foreign body in respiratory system | 2020-2023 | +24.7% (+13.7 to +36.2) | 27 (9-56) | +29.0% (+16.9 to +41.3) | 20 (6-50) | +26.5% (+15.9 to +37.5) | 47 (16-108) |
|  | Fracture of clavicle, scapula, or humerus | 2020-2023 | +24.7% (+13.3 to +35.9) | 164 (78-293) | +29.0% (+16.3 to +41.9) | 122 (55-241) | +26.4% (+15.9 to +38.4) | 286 (134-520) |
|  | Fracture of hand (wrist and other distal part of hand) | 2020-2023 | +25.1% (+14.2 to +36.8) | 417 (227-700) | +29.3% (+17.2 to +42.3) | 202 (106-383) | +26.4% (+16.2 to +38.9) | 618 (336-1,061) |
|  | Dislocation of shoulder | 2020-2023 | +24.6% (+13.8 to +36.7) | 58 (22-136) | +28.9% (+17.4 to +41.4) | 43 (13-111) | +26.4% (+15.5 to +38.6) | 101 (36-250) |
|  | Fracture of skull | 2020-2023 | +25.4% (+14.0 to +37.4) | 412 (229-687) | +29.6% (+17.3 to +42.0) | 125 (59-250) | +26.4% (+15.3 to +38.1) | 537 (289-921) |
|  | Spinal cord lesion below neck level | 2020-2023 | +24.7% (+13.7 to +35.7) | 7 (3-13) | +29.7% (+18.3 to +41.4) | 4 (1-9) | +26.4% (+16.2 to +37.0) | 10 (4-23) |
|  | Open wound(s) | 2020-2023 | +24.9% (+13.9 to +36.6) | 1,931 (1,202-2,748) | +29.2% (+17.0 to +41.7) | 1,013 (589-1,505) | +26.3% (+15.8 to +37.4) | 2,944 (1,837-4,224) |
|  | Burns, >=20% total burned surface area or >= 10% burned surface area if head/neck or hands/wrist involved w/o lower airway burns | 2020-2023 | +23.6% (+13.2 to +35.0) | 4 (2-7) | +28.8% (+17.2 to +41.2) | 4 (2-8) | +26.3% (+14.8 to +37.2) | 8 (4-14) |
|  | Fracture of face bones | 2020-2023 | +25.1% (+13.8 to +37.2) | 548 (301-861) | +29.4% (+17.3 to +41.5) | 203 (108-345) | +26.3% (+15.8 to +37.3) | 752 (411-1,191) |
|  | Fracture of femur, other than femoral neck | 2020-2023 | +24.5% (+13.7 to +37.6) | 51 (24-99) | +28.8% (+16.2 to +41.8) | 34 (15-73) | +26.2% (+15.1 to +38.3) | 86 (38-169) |
|  | Fracture of patella, tibia or fibula, or ankle | 2020-2023 | +24.5% (+13.8 to +36.4) | 162 (89-270) | +28.9% (+16.7 to +40.8) | 94 (50-173) | +26.1% (+15.8 to +37.0) | 256 (138-430) |
|  | Fracture of radius and/or ulna | 2020-2023 | +24.2% (+12.6 to +36.3) | 120 (61-205) | +28.6% (+16.1 to +41.2) | 92 (44-169) | +26.1% (+15.0 to +37.7) | 212 (105-368) |
|  | Internal hemorrhage in abdomen and pelvis | 2020-2023 | +25.1% (+14.2 to +36.7) | 310 (177-481) | +29.3% (+17.5 to +41.5) | 98 (51-166) | +26.0% (+15.7 to +36.7) | 408 (230-638) |
|  | Severe chest Injury | 2020-2023 | +24.7% (+13.6 to +35.6) | 242 (132-398) | +29.0% (+17.4 to +41.0) | 71 (37-128) | +25.6% (+15.3 to +36.6) | 314 (170-514) |
|  | Minor TBI | 2020-2023 | +23.9% (+12.9 to +35.3) | 275 (154-463) | +28.6% (+16.2 to +41.0) | 157 (78-293) | +25.6% (+15.4 to +37.3) | 432 (242-760) |
|  | Fracture of vertebral column | 2020-2023 | +23.8% (+12.9 to +35.4) | 57 (27-111) | +28.2% (+16.9 to +40.4) | 39 (16-82) | +25.6% (+15.0 to +37.8) | 96 (44-196) |
|  | Multiple fractures, dislocations, crashes, wounds, pains, and strains | 2020-2023 | +23.8% (+12.4 to +35.3) | 59 (30-103) | +28.2% (+16.9 to +40.7) | 34 (16-65) | +25.3% (+15.0 to +36.4) | 93 (46-168) |
|  | Amputation of upper limb, unilateral | 2020-2023 | +23.4% (+12.3 to +34.9) | 2 (1-4) | +28.6% (+17.6 to +41.3) | 1 (0-3) | +25.3% (+14.1 to +37.3) | 3 (1-7) |
|  | Amputation of upper limbs, bilateral | 2020-2023 | +23.6% (+13.0 to +35.5) | 2 (1-4) | +28.7% (+17.2 to +41.4) | 1 (0-2) | +25.2% (+14.6 to +36.7) | 3 (1-7) |
|  | Fracture of pelvis | 2020-2023 | +23.0% (+11.0 to +35.5) | 37 (13-84) | +27.5% (+15.4 to +40.4) | 34 (10-89) | +25.1% (+14.4 to +36.9) | 71 (23-171) |
|  | Fracture of sternum and/or fracture of one or more ribs | 2020-2023 | +23.7% (+12.9 to +34.9) | 114 (51-218) | +28.0% (+16.7 to +40.2) | 44 (17-95) | +24.8% (+14.9 to +36.0) | 158 (68-299) |
|  | Crush injury | 2020-2023 | +22.4% (+10.9 to +34.4) | 2 (1-4) | +27.9% (+15.7 to +39.9) | 1 (0-2) | +24.1% (+13.9 to +35.5) | 3 (1-6) |
|  | Spinal cord lesion at neck level | 2020-2023 | +22.1% (+11.3 to +32.4) | 4 (2-9) | +27.5% (+15.5 to +39.6) | 2 (1-6) | +23.9% (+14.0 to +34.7) | 7 (3-15) |
|  | Complications following therapeutic procedures | 2020-2023 | +21.6% (+10.7 to +32.4) | 13 (7-22) | +26.5% (+14.7 to +38.4) | 11 (6-21) | +23.8% (+13.3 to +34.6) | 25 (13-43) |
|  | Moderate/Severe TBI | 2020-2023 | +21.6% (+11.3 to +32.7) | 80 (48-117) | +27.0% (+15.2 to +39.6) | 34 (20-56) | +23.1% (+12.8 to +33.4) | 114 (68-170) |
|  | Fracture of hip | 2020-2023 | +17.8% (+5.7 to +29.4) | 8 (3-16) | +23.0% (+11.2 to +34.6) | 8 (3-17) | +20.3% (+9.5 to +32.1) | 15 (7-32) |
|  | Amputation of lower limb, unilateral | 2020-2023 | +16.9% (+5.5 to +28.3) | 0 (0-0) | +22.8% (+11.5 to +34.9) | 0 (0-0) | +19.2% (+8.7 to +30.4) | 0 (0-0) |
|  | Fracture of hip | 2010-2023 |  |  | +6.0% (+1.5 to +11.2) | 8 (3-17) | +5.1% (+0.4 to +9.8) | 15 (7-32) |
|  | Severe chest Injury | 2010-2023 |  |  | +4.2% (+0.0 to +8.5) | 71 (37-128) | +5.0% (+0.5 to +9.3) | 314 (170-514) |
|  | Fracture of skull | 2010-2023 |  |  |  |  | +5.0% (+0.6 to +9.2) | 537 (289-921) |
|  | Fracture of sternum and/or fracture of one or more ribs | 2010-2023 |  |  | +4.5% (+0.3 to +8.8) | 44 (17-95) | +5.0% (+0.7 to +9.1) | 158 (68-299) |
|  | Internal hemorrhage in abdomen and pelvis | 2010-2023 | +5.2% (+0.0 to +10.4) | 310 (177-481) | +4.1% (+0.2 to +8.6) | 98 (51-166) | +5.0% (+0.5 to +9.2) | 408 (230-638) |
|  | Fracture of face bones | 2010-2023 |  |  |  |  | +4.9% (+0.5 to +9.3) | 752 (411-1,191) |
|  | Complications following therapeutic procedures | 2010-2023 |  |  | +5.0% (+0.9 to +9.3) | 11 (6-21) | +4.9% (+1.0 to +8.7) | 25 (13-43) |
|  | Fracture of hand (wrist and other distal part of hand) | 2010-2023 |  |  |  |  | +4.9% (+1.1 to +9.0) | 618 (336-1,061) |
|  | Fracture of pelvis | 2010-2023 |  |  | +4.7% (+0.5 to +9.6) | 34 (10-89) | +4.9% (+1.0 to +9.1) | 71 (23-171) |
|  | Minor TBI | 2010-2023 |  |  | +4.4% (+0.6 to +8.8) | 157 (78-293) | +4.9% (+1.0 to +8.6) | 432 (242-760) |
|  | Open wound(s) | 2010-2023 | +5.2% (+0.1 to +10.0) | 1,931 (1,202-2,748) |  |  | +4.9% (+1.1 to +8.8) | 2,944 (1,837-4,224) |
|  | Fracture of patella, tibia or fibula, or ankle | 2010-2023 |  |  | +4.3% (+0.0 to +8.7) | 94 (50-173) | +4.9% (+1.0 to +8.8) | 256 (138-430) |
|  | Amputation of toe/toes | 2010-2023 |  |  | +4.2% (+0.2 to +8.3) | 19 (5-47) | +4.9% (+0.9 to +9.1) | 46 (15-102) |
|  | Multiple fractures, dislocations, crashes, wounds, pains, and strains | 2010-2023 |  |  | +4.4% (+0.2 to +8.8) | 34 (16-65) | +4.9% (+0.7 to +9.0) | 93 (46-168) |
|  | Injury to eyes | 2010-2023 |  |  | +4.1% (+0.1 to +8.8) | 198 (94-362) | +4.9% (+0.9 to +8.7) | 561 (286-912) |
|  | Fracture of femur, other than femoral neck | 2010-2023 |  |  | +4.3% (+0.3 to +8.9) | 34 (15-73) | +4.9% (+0.9 to +8.7) | 86 (38-169) |
|  | Dislocation of shoulder | 2010-2023 |  |  |  |  | +4.9% (+1.1 to +8.8) | 101 (36-250) |
|  | Fracture of clavicle, scapula, or humerus | 2010-2023 |  |  | +4.3% (+0.1 to +9.0) | 122 (55-241) | +4.8% (+1.0 to +8.9) | 286 (134-520) |
|  | Fracture of radius and/or ulna | 2010-2023 |  |  | +4.4% (+0.3 to +8.9) | 92 (44-169) | +4.8% (+1.3 to +8.8) | 212 (105-368) |
|  | Amputation of fingers (excluding thumb) | 2010-2023 |  |  |  |  | +4.8% (+0.6 to +9.3) | 57 (22-116) |
|  | Superficial injury of any part of the body | 2010-2023 |  |  |  |  | +4.8% (+1.1 to +8.6) | 1,958 (1,119-3,010) |
|  | Fracture of vertebral column | 2010-2023 |  |  | +4.4% (+0.4 to +9.2) | 39 (16-82) | +4.8% (+0.7 to +9.1) | 96 (44-196) |
|  | Foreign body in GI and urogenital system | 2010-2023 | +5.3% (+0.3 to +10.1) | 38 (15-85) |  |  | +4.8% (+1.0 to +8.6) | 63 (22-156) |
|  | Foreign body in respiratory system | 2010-2023 |  |  | +4.2% (+0.0 to +8.3) | 20 (6-50) | +4.8% (+0.7 to +8.8) | 47 (16-108) |
|  | Fracture of foot bones except ankle | 2010-2023 |  |  |  |  | +4.8% (+0.9 to +9.2) | 118 (54-244) |
|  | Muscle and tendon injuries, including sprains and strains lesser dislocations | 2010-2023 |  |  |  |  | +4.8% (+0.9 to +8.6) | 499 (287-817) |
|  | Nerve injury | 2010-2023 | +5.3% (+0.3 to +9.9) | 91 (42-190) |  |  | +4.7% (+1.2 to +8.7) | 148 (63-321) |
|  | Contusion in any part of the body | 2010-2023 |  |  | +4.1% (+0.1 to +8.6) | 747 (413-1,217) | +4.7% (+0.9 to +8.7) | 1,576 (906-2,486) |
|  | Amputation of thumb | 2010-2023 | +5.3% (+0.0 to +10.2) | 37 (13-88) |  |  | +4.7% (+0.8 to +8.5) | 62 (22-156) |
|  | Amputation of lower limb, unilateral | 2010-2023 |  |  | +5.9% (+1.5 to +11.5) | 0 (0-0) | +4.7% (+0.1 to +9.4) | 0 (0-0) |
|  | Asphyxiation | 2010-2023 | +5.4% (+0.2 to +10.2) | 44 (15-105) |  |  | +4.7% (+1.3 to +8.3) | 79 (24-204) |
|  | Dislocation of hip | 2010-2023 |  |  |  |  | +4.7% (+1.1 to +8.9) | 48 (15-121) |
|  | Dislocation of knee | 2010-2023 | +5.3% (+0.3 to +10.0) | 39 (13-85) | +3.9% (+0.1 to +8.2) | 32 (9-90) | +4.7% (+1.2 to +8.4) | 71 (23-173) |
|  | Drowning and nonfatal submersion | 2010-2023 | +5.4% (+0.3 to +10.1) | 41 (13-92) |  |  | +4.6% (+1.0 to +8.5) | 74 (22-188) |
|  | Poisoning requiring urgent care | 2010-2023 |  |  |  |  | +4.6% (+1.1 to +8.6) | 153 (81-279) |
|  | Effect of different environmental factors | 2010-2023 |  |  |  |  | +4.6% (+1.0 to +8.9) | 82 (33-185) |
|  | Foreign body in ear | 2010-2023 |  |  |  |  | +4.6% (+1.1 to +8.8) | 75 (24-176) |
|  | Crush injury | 2010-2023 |  |  | +4.5% (+0.1 to +9.3) | 1 (0-2) | +4.6% (+0.5 to +9.2) | 3 (1-6) |
|  | Moderate/Severe TBI | 2010-2023 |  |  | +4.6% (+0.5 to +9.1) | 34 (20-56) | +4.6% (+0.0 to +9.0) | 114 (68-170) |
|  | Spinal cord lesion at neck level | 2010-2023 |  |  | +4.5% (+0.5 to +8.8) | 2 (1-6) | +4.5% (+0.3 to +8.9) | 7 (3-15) |
|  | Spinal cord lesion below neck level | 2010-2023 |  |  |  |  | +4.5% (+0.5 to +8.5) | 10 (4-23) |
|  | Burns, <20% total burned surface area without lower airway burns | 2010-2023 | +5.4% (+0.2 to +10.5) | 122 (57-242) |  |  | +4.5% (+0.9 to +8.5) | 233 (106-473) |
|  | Amputation of upper limb, unilateral | 2010-2023 |  |  |  |  | +4.5% (+0.0 to +8.7) | 3 (1-7) |
|  | Amputation of upper limbs, bilateral | 2010-2023 |  |  |  |  | +4.5% (+0.4 to +8.8) | 3 (1-7) |
|  | Amputation of lower limbs, bilateral | 2010-2023 |  |  |  |  | +4.4% (+0.5 to +8.4) | 3 (1-9) |
|  | Burns, >=20% total burned surface area or >= 10% burned surface area if head/neck or hands/wrist involved w/o lower airway burns | 2010-2023 |  |  |  |  | +4.4% (+0.6 to +8.2) | 8 (4-14) |
|  | Lower airway burns | 2010-2023 |  |  |  |  | +4.4% (+0.5 to +8.2) | 4 (1-10) |

## Latvia

| **Age** | **Category** | **Period** | **Male %Change** | **Male Incidence 2023** | **Female %Change** | **Female Incidence 2023** | **Both %Change** | **Both Incidence 2023** |
| --- | --- | --- | --- | --- | --- | --- | --- | --- |
| 5-14 years | Fracture of skull | 2020-2023 |  |  |  |  | +13.1% (+1.6 to +22.1) | 7 (3-13) |
|  | Fracture of face bones | 2020-2023 |  |  |  |  | +13.1% (+1.6 to +22.0) | 14 (7-23) |
|  | Internal hemorrhage in abdomen and pelvis | 2020-2023 |  |  | +13.0% (+0.4 to +31.3) | 8 (4-14) | +13.0% (+0.7 to +21.9) | 25 (13-39) |
|  | Injury to eyes | 2020-2023 |  |  | +13.2% (+0.4 to +29.9) | 6 (3-12) | +13.0% (+1.9 to +21.6) | 13 (6-24) |
|  | Amputation of thumb | 2020-2023 |  |  |  |  | +13.0% (+2.5 to +23.1) | 2 (1-4) |
|  | Burns, <20% total burned surface area without lower airway burns | 2020-2023 |  |  |  |  | +13.0% (+1.9 to +21.9) | 13 (5-26) |
|  | Nerve injury | 2020-2023 |  |  |  |  | +13.0% (+1.9 to +22.6) | 4 (1-9) |
|  | Fracture of foot bones except ankle | 2020-2023 |  |  |  |  | +13.0% (+0.6 to +21.9) | 4 (2-9) |
|  | Foreign body in ear | 2020-2023 |  |  |  |  | +13.0% (+3.1 to +22.3) | 1 (1-4) |
|  | Severe chest Injury | 2020-2023 |  |  | +13.0% (+0.7 to +29.5) | 5 (2-9) | +13.0% (+1.4 to +21.6) | 15 (8-27) |
|  | Dislocation of hip | 2020-2023 |  |  |  |  | +12.9% (+0.9 to +23.1) | 1 (0-3) |
|  | Fracture of hand (wrist and other distal part of hand) | 2020-2023 |  |  |  |  | +12.9% (+1.2 to +21.8) | 10 (5-18) |
|  | Open wound(s) | 2020-2023 |  |  | +13.1% (+0.3 to +29.3) | 59 (33-95) | +12.9% (+2.8 to +21.3) | 125 (73-198) |
|  | Asphyxiation | 2020-2023 |  |  | +13.0% (+0.2 to +29.9) | 1 (0-2) | +12.9% (+2.2 to +21.8) | 1 (0-3) |
|  | Foreign body in GI and urogenital system | 2020-2023 |  |  | +13.1% (+0.3 to +31.5) | 1 (0-3) | +12.9% (+1.6 to +24.0) | 2 (1-5) |
|  | Muscle and tendon injuries, including sprains and strains lesser dislocations | 2020-2023 |  |  |  |  | +12.9% (+1.4 to +22.4) | 31 (17-53) |
|  | Amputation of fingers (excluding thumb) | 2020-2023 |  |  |  |  | +12.9% (+2.9 to +22.7) | 4 (1-8) |
|  | Foreign body in respiratory system | 2020-2023 |  |  |  |  | +12.9% (+2.0 to +22.1) | 2 (1-5) |
|  | Multiple fractures, dislocations, crashes, wounds, pains, and strains | 2020-2023 |  |  |  |  | +12.9% (+1.7 to +21.9) | 8 (4-14) |
|  | Fracture of sternum and/or fracture of one or more ribs | 2020-2023 |  |  | +12.9% (+1.4 to +27.3) | 1 (0-1) | +12.9% (+1.0 to +21.2) | 1 (0-3) |
|  | Drowning and nonfatal submersion | 2020-2023 |  |  |  |  | +12.9% (+1.8 to +21.5) | 1 (0-4) |
|  | Contusion in any part of the body | 2020-2023 |  |  | +13.1% (+0.5 to +29.9) | 8 (4-15) | +12.9% (+1.8 to +22.6) | 14 (7-25) |
|  | Fracture of femur, other than femoral neck | 2020-2023 |  |  | +13.0% (+0.3 to +28.6) | 4 (2-10) | +12.9% (+2.2 to +21.5) | 8 (4-17) |
|  | Effect of different environmental factors | 2020-2023 |  |  |  |  | +12.8% (+2.3 to +22.9) | 3 (1-6) |
|  | Dislocation of knee | 2020-2023 |  |  |  |  | +12.8% (+3.1 to +22.9) | 2 (0-4) |
|  | Fracture of patella, tibia or fibula, or ankle | 2020-2023 |  |  | +12.9% (+0.5 to +27.8) | 7 (3-12) | +12.8% (+1.6 to +21.4) | 14 (7-23) |
|  | Spinal cord lesion below neck level | 2020-2023 |  |  | +13.0% (+1.1 to +29.7) | 0 (0-0) | +12.8% (+1.8 to +21.1) | 0 (0-1) |
|  | Fracture of clavicle, scapula, or humerus | 2020-2023 |  |  |  |  | +12.8% (+2.6 to +21.5) | 9 (4-18) |
|  | Superficial injury of any part of the body | 2020-2023 |  |  | +13.0% (+0.1 to +30.1) | 15 (7-27) | +12.8% (+1.8 to +21.9) | 26 (13-46) |
|  | Dislocation of shoulder | 2020-2023 |  |  | +13.0% (+0.4 to +28.2) | 1 (0-3) | +12.8% (+2.2 to +21.2) | 2 (1-4) |
|  | Minor TBI | 2020-2023 |  |  | +13.0% (+0.2 to +30.5) | 4 (2-8) | +12.8% (+0.6 to +21.7) | 9 (4-16) |
|  | Amputation of lower limbs, bilateral | 2020-2023 |  |  |  |  | +12.8% (+1.6 to +21.6) | 0 (0-0) |
|  | Amputation of upper limbs, bilateral | 2020-2023 |  |  |  |  | +12.7% (+1.3 to +22.2) | 0 (0-0) |
|  | Fracture of vertebral column | 2020-2023 |  |  | +12.9% (+0.5 to +29.3) | 2 (1-4) | +12.7% (+2.0 to +20.9) | 3 (1-7) |
|  | Amputation of toe/toes | 2020-2023 |  |  |  |  | +12.7% (+1.7 to +21.9) | 1 (0-3) |
|  | Fracture of radius and/or ulna | 2020-2023 |  |  | +13.0% (+0.2 to +29.3) | 9 (4-16) | +12.7% (+1.8 to +21.9) | 15 (7-28) |
|  | Poisoning requiring urgent care | 2020-2023 |  |  | +13.0% (+0.6 to +27.5) | 12 (6-22) | +12.7% (+1.7 to +22.1) | 18 (9-32) |
|  | Crush injury | 2020-2023 |  |  | +12.9% (+0.0 to +29.1) | 0 (0-0) | +12.7% (+1.6 to +21.7) | 0 (0-1) |
|  | Moderate/Severe TBI | 2020-2023 |  |  | +12.9% (+0.3 to +28.0) | 4 (3-7) | +12.7% (+1.2 to +20.8) | 10 (6-16) |
|  | Burns, >=20% total burned surface area or >= 10% burned surface area if head/neck or hands/wrist involved w/o lower airway burns | 2020-2023 |  |  |  |  | +12.7% (+2.2 to +21.8) | 2 (1-3) |
|  | Spinal cord lesion at neck level | 2020-2023 |  |  |  |  | +12.7% (+1.2 to +20.9) | 0 (0-0) |
|  | Complications following therapeutic procedures | 2020-2023 |  |  | +13.0% (+1.1 to +28.2) | 4 (2-7) | +12.7% (+2.9 to +21.2) | 6 (3-11) |
|  | Lower airway burns | 2020-2023 |  |  |  |  | +12.7% (+1.1 to +21.9) | 0 (0-0) |
|  | Amputation of upper limb, unilateral | 2020-2023 |  |  |  |  | +12.7% (+1.3 to +23.0) | 0 (0-0) |
|  | Fracture of pelvis | 2020-2023 |  |  |  |  | +12.4% (+2.3 to +22.6) | 1 (0-3) |
|  | Fracture of hip | 2020-2023 |  |  |  |  | +12.3% (+1.8 to +21.3) | 1 (0-1) |
|  | Amputation of lower limb, unilateral | 2020-2023 |  |  |  |  | +12.1% (+0.7 to +21.2) | 0 (0-0) |

## Lithuania

| **Age** | **Category** | **Period** | **Male %Change** | **Male Incidence 2023** | **Female %Change** | **Female Incidence 2023** | **Both %Change** | **Both Incidence 2023** |
| --- | --- | --- | --- | --- | --- | --- | --- | --- |
| <5 years | Fracture of skull | 2020-2023 | +14.0% (+1.9 to +29.1) | 18 (10-30) |  |  | +9.0% (+0.9 to +21.8) | 25 (14-44) |
|  | Severe chest Injury | 2020-2023 | +14.0% (+1.6 to +28.5) | 36 (22-55) |  |  | +9.0% (+0.1 to +21.3) | 49 (29-80) |
|  | Internal hemorrhage in abdomen and pelvis | 2020-2023 | +13.9% (+1.9 to +29.2) | 61 (39-90) |  |  | +8.8% (+0.2 to +22.0) | 84 (53-125) |
|  | Fracture of face bones | 2020-2023 | +14.0% (+1.8 to +28.3) | 31 (19-49) |  |  |  |  |
|  | Fracture of sternum and/or fracture of one or more ribs | 2020-2023 | +13.9% (+1.5 to +28.5) | 3 (1-5) |  |  |  |  |
|  | Moderate/Severe TBI | 2020-2023 | +13.8% (+1.8 to +27.8) | 21 (15-29) |  |  |  |  |
|  | Fracture of hand (wrist and other distal part of hand) | 2020-2023 | +14.0% (+2.3 to +27.9) | 20 (12-31) |  |  |  |  |
|  | Crush injury | 2020-2023 | +13.9% (+2.6 to +28.1) | 1 (1-2) |  |  |  |  |
|  | Amputation of fingers (excluding thumb) | 2020-2023 | +14.0% (+1.2 to +28.5) | 7 (4-13) |  |  |  |  |
|  | Open wound(s) | 2020-2023 | +14.0% (+1.9 to +27.1) | 239 (166-325) |  |  |  |  |
|  | Amputation of upper limbs, bilateral | 2020-2023 | +13.7% (+1.8 to +28.0) | 0 (0-1) |  |  |  |  |
|  | Injury to eyes | 2020-2023 | +14.1% (+2.4 to +28.6) | 25 (15-41) |  |  |  |  |
|  | Multiple fractures, dislocations, crashes, wounds, pains, and strains | 2020-2023 | +14.1% (+2.0 to +27.2) | 14 (8-24) |  |  |  |  |
|  | Fracture of patella, tibia or fibula, or ankle | 2020-2023 | +14.1% (+2.4 to +27.3) | 25 (14-39) |  |  |  |  |
|  | Spinal cord lesion below neck level | 2020-2023 | +13.8% (+2.5 to +27.5) | 1 (0-1) |  |  |  |  |
|  | Minor TBI | 2020-2023 | +14.1% (+2.4 to +28.5) | 15 (9-24) |  |  |  |  |
|  | Fracture of foot bones except ankle | 2020-2023 | +14.0% (+2.8 to +28.2) | 9 (4-16) |  |  |  |  |
|  | Spinal cord lesion at neck level | 2020-2023 | +13.8% (+1.7 to +27.5) | 0 (0-1) |  |  |  |  |
|  | Nerve injury | 2020-2023 | +14.0% (+2.2 to +27.4) | 7 (3-13) |  |  |  |  |
|  | Amputation of upper limb, unilateral | 2020-2023 | +13.7% (+2.5 to +27.0) | 0 (0-1) |  |  |  |  |
|  | Fracture of femur, other than femoral neck | 2020-2023 | +14.1% (+2.3 to +27.5) | 14 (7-26) |  |  |  |  |
|  | Foreign body in GI and urogenital system | 2020-2023 | +14.1% (+1.3 to +29.0) | 4 (2-8) |  |  |  |  |
|  | Amputation of toe/toes | 2020-2023 | +14.0% (+1.7 to +28.4) | 2 (1-5) |  |  |  |  |
|  | Amputation of thumb | 2020-2023 | +14.1% (+1.0 to +31.6) | 3 (1-6) |  |  |  |  |
|  | Fracture of vertebral column | 2020-2023 | +14.0% (+3.3 to +27.9) | 6 (3-11) |  |  |  |  |
|  | Foreign body in ear | 2020-2023 | +14.0% (+1.7 to +30.0) | 3 (1-5) |  |  |  |  |
|  | Fracture of clavicle, scapula, or humerus | 2020-2023 | +14.1% (+2.3 to +28.7) | 14 (8-25) |  |  |  |  |
|  | Dislocation of shoulder | 2020-2023 | +14.0% (+2.2 to +29.3) | 3 (1-6) |  |  |  |  |
|  | Dislocation of hip | 2020-2023 | +14.1% (+2.4 to +28.2) | 2 (1-5) |  |  |  |  |
|  | Muscle and tendon injuries, including sprains and strains lesser dislocations | 2020-2023 | +14.0% (+2.0 to +27.3) | 50 (33-72) |  |  |  |  |
|  | Asphyxiation | 2020-2023 | +13.9% (+2.2 to +29.3) | 2 (1-5) |  |  |  |  |
|  | Foreign body in respiratory system | 2020-2023 | +14.0% (+3.1 to +28.1) | 3 (1-7) |  |  |  |  |
|  | Fracture of radius and/or ulna | 2020-2023 | +14.0% (+2.2 to +28.9) | 24 (14-37) |  |  |  |  |
|  | Lower airway burns | 2020-2023 | +13.8% (+2.0 to +28.5) | 0 (0-0) |  |  |  |  |
|  | Amputation of lower limb, unilateral | 2020-2023 | +13.9% (+2.2 to +28.8) | 0 (0-0) |  |  |  |  |
|  | Severe chest Injury | 2010-2023 | +16.1% (+0.3 to +30.5) | 36 (22-55) |  |  |  |  |
|  | Superficial injury of any part of the body | 2020-2023 | +14.0% (+2.3 to +28.1) | 39 (26-58) |  |  |  |  |
|  | Drowning and nonfatal submersion | 2020-2023 | +13.9% (+2.2 to +29.8) | 2 (1-5) |  |  |  |  |
|  | Dislocation of knee | 2020-2023 | +14.0% (+1.9 to +27.8) | 3 (1-5) |  |  |  |  |
|  | Fracture of skull | 2010-2023 | +16.1% (+1.0 to +29.9) | 18 (10-30) |  |  |  |  |
|  | Contusion in any part of the body | 2020-2023 | +14.0% (+2.4 to +26.7) | 21 (13-33) |  |  |  |  |
|  | Amputation of lower limbs, bilateral | 2020-2023 | +13.8% (+2.5 to +29.9) | 0 (0-0) |  |  |  |  |
|  | Effect of different environmental factors | 2020-2023 | +13.9% (+2.6 to +27.3) | 4 (2-8) |  |  |  |  |
|  | Complications following therapeutic procedures | 2020-2023 | +14.0% (+2.5 to +27.3) | 7 (4-12) |  |  |  |  |
|  | Burns, <20% total burned surface area without lower airway burns | 2020-2023 | +14.1% (+1.4 to +28.5) | 21 (12-36) |  |  |  |  |
|  | Fracture of pelvis | 2020-2023 | +14.0% (+2.4 to +29.0) | 2 (1-4) |  |  |  |  |
|  | Internal hemorrhage in abdomen and pelvis | 2010-2023 | +16.0% (+1.1 to +31.1) | 61 (39-90) |  |  |  |  |
|  | Fracture of hip | 2020-2023 | +14.1% (+2.2 to +29.9) | 1 (0-1) |  |  |  |  |
|  | Burns, >=20% total burned surface area or >= 10% burned surface area if head/neck or hands/wrist involved w/o lower airway burns | 2020-2023 | +13.8% (+1.9 to +29.0) | 3 (2-4) |  |  |  |  |
|  | Poisoning requiring urgent care | 2020-2023 | +14.1% (+3.0 to +27.3) | 23 (13-37) |  |  |  |  |
|  | Fracture of face bones | 2010-2023 | +16.2% (+0.6 to +31.7) | 31 (19-49) |  |  |  |  |
|  | Fracture of sternum and/or fracture of one or more ribs | 2010-2023 | +16.2% (+0.2 to +31.1) | 3 (1-5) |  |  |  |  |
|  | Moderate/Severe TBI | 2010-2023 | +15.6% (+0.4 to +30.0) | 21 (15-29) |  |  |  |  |
|  | Fracture of hand (wrist and other distal part of hand) | 2010-2023 | +16.1% (+0.1 to +30.6) | 20 (12-31) |  |  |  |  |
|  | Crush injury | 2010-2023 | +15.7% (+0.9 to +29.3) | 1 (1-2) |  |  |  |  |
|  | Open wound(s) | 2010-2023 | +16.2% (+0.3 to +30.4) | 239 (166-325) |  |  |  |  |
|  | Amputation of fingers (excluding thumb) | 2010-2023 | +16.3% (+0.2 to +30.4) | 7 (4-13) |  |  |  |  |
|  | Injury to eyes | 2010-2023 | +16.2% (+0.1 to +31.8) | 25 (15-41) |  |  |  |  |
|  | Multiple fractures, dislocations, crashes, wounds, pains, and strains | 2010-2023 | +16.2% (+0.2 to +30.6) | 14 (8-24) |  |  |  |  |
|  | Fracture of patella, tibia or fibula, or ankle | 2010-2023 | +16.2% (+0.3 to +31.0) | 25 (14-39) |  |  |  |  |
|  | Minor TBI | 2010-2023 | +16.3% (+0.7 to +30.4) | 15 (9-24) |  |  |  |  |
|  | Spinal cord lesion below neck level | 2010-2023 | +15.4% (+0.8 to +28.7) | 1 (0-1) |  |  |  |  |
|  | Fracture of foot bones except ankle | 2010-2023 | +16.1% (+1.3 to +30.3) | 9 (4-16) |  |  |  |  |
|  | Nerve injury | 2010-2023 | +16.1% (+0.3 to +28.8) | 7 (3-13) |  |  |  |  |
|  | Amputation of upper limb, unilateral | 2010-2023 | +15.5% (+0.8 to +29.0) | 0 (0-1) |  |  |  |  |
|  | Fracture of femur, other than femoral neck | 2010-2023 | +16.2% (+0.7 to +30.8) | 14 (7-26) |  |  |  |  |
|  | Foreign body in GI and urogenital system | 2010-2023 | +16.2% (+0.1 to +31.1) | 4 (2-8) |  |  |  |  |
|  | Amputation of toe/toes | 2010-2023 | +16.1% (+0.6 to +30.2) | 2 (1-5) |  |  |  |  |
|  | Fracture of vertebral column | 2010-2023 | +16.2% (+0.0 to +30.5) | 6 (3-11) |  |  |  |  |
|  | Foreign body in ear | 2010-2023 | +16.1% (+0.1 to +31.1) | 3 (1-5) |  |  |  |  |
|  | Fracture of clavicle, scapula, or humerus | 2010-2023 | +16.2% (+0.7 to +29.5) | 14 (8-25) |  |  |  |  |
|  | Foreign body in respiratory system | 2010-2023 | +16.1% (+0.2 to +30.6) | 3 (1-7) |  |  |  |  |
|  | Muscle and tendon injuries, including sprains and strains lesser dislocations | 2010-2023 | +16.1% (+0.1 to +31.3) | 50 (33-72) |  |  |  |  |
|  | Fracture of radius and/or ulna | 2010-2023 | +16.1% (+0.6 to +30.7) | 24 (14-37) |  |  |  |  |
|  | Dislocation of hip | 2010-2023 | +16.1% (+0.3 to +30.3) | 2 (1-5) |  |  |  |  |
|  | Superficial injury of any part of the body | 2010-2023 | +16.2% (+1.3 to +30.5) | 39 (26-58) |  |  |  |  |
|  | Dislocation of knee | 2010-2023 | +16.1% (+0.2 to +29.3) | 3 (1-5) |  |  |  |  |
|  | Drowning and nonfatal submersion | 2010-2023 | +16.0% (+1.3 to +30.6) | 2 (1-5) |  |  |  |  |
|  | Effect of different environmental factors | 2010-2023 | +16.0% (+0.6 to +30.8) | 4 (2-8) |  |  |  |  |
|  | Contusion in any part of the body | 2010-2023 | +16.1% (+1.7 to +30.2) | 21 (13-33) |  |  |  |  |
|  | Amputation of lower limbs, bilateral | 2010-2023 | +15.6% (+0.6 to +34.1) | 0 (0-0) |  |  |  |  |
|  | Complications following therapeutic procedures | 2010-2023 | +16.2% (+1.2 to +31.1) | 7 (4-12) |  |  |  |  |
|  | Fracture of pelvis | 2010-2023 | +16.2% (+0.9 to +32.0) | 2 (1-4) |  |  |  |  |
|  | Fracture of hip | 2010-2023 | +16.5% (+0.8 to +32.5) | 1 (0-1) |  |  |  |  |
|  | Poisoning requiring urgent care | 2010-2023 | +16.2% (+2.2 to +30.0) | 23 (13-37) |  |  |  |  |
| 5-14 years | Fracture of hip | 2020-2023 |  |  | +14.0% (+2.4 to +31.2) | 1 (0-1) | +13.8% (+5.2 to +26.7) | 1 (0-2) |
|  | Fracture of sternum and/or fracture of one or more ribs | 2020-2023 |  |  | +13.4% (+0.8 to +29.6) | 1 (0-2) | +13.8% (+4.2 to +25.8) | 2 (1-4) |
|  | Amputation of lower limb, unilateral | 2020-2023 |  |  | +13.8% (+3.5 to +33.2) | 0 (0-0) | +13.7% (+5.0 to +27.3) | 0 (0-0) |
|  | Crush injury | 2020-2023 |  |  | +13.3% (+1.8 to +30.2) | 0 (0-1) | +13.7% (+4.5 to +25.1) | 1 (0-1) |
|  | Severe chest Injury | 2020-2023 |  |  | +13.1% (+0.8 to +31.6) | 7 (3-14) | +13.7% (+2.6 to +25.7) | 21 (12-40) |
|  | Moderate/Severe TBI | 2020-2023 |  |  | +13.3% (+1.8 to +30.0) | 6 (4-10) | +13.7% (+4.3 to +24.5) | 15 (9-23) |
|  | Internal hemorrhage in abdomen and pelvis | 2020-2023 |  |  | +13.1% (+1.2 to +29.9) | 12 (6-21) | +13.6% (+3.4 to +24.5) | 36 (18-59) |
|  | Amputation of upper limb, unilateral | 2020-2023 |  |  | +13.3% (+1.7 to +33.1) | 0 (0-0) | +13.6% (+3.5 to +27.9) | 0 (0-1) |
|  | Spinal cord lesion at neck level | 2020-2023 | +13.8% (+0.9 to +26.0) | 0 (0-0) | +13.4% (+1.0 to +30.5) | 0 (0-0) | +13.6% (+5.9 to +25.0) | 0 (0-1) |
|  | Fracture of skull | 2020-2023 |  |  | +13.0% (+1.6 to +30.3) | 3 (1-7) | +13.6% (+2.9 to +24.8) | 10 (5-19) |
|  | Amputation of upper limbs, bilateral | 2020-2023 |  |  | +13.2% (+1.9 to +30.1) | 0 (0-0) | +13.6% (+4.2 to +23.8) | 0 (0-1) |
|  | Multiple fractures, dislocations, crashes, wounds, pains, and strains | 2020-2023 |  |  | +13.3% (+3.0 to +29.9) | 6 (3-11) | +13.5% (+3.7 to +24.9) | 12 (5-22) |
|  | Fracture of face bones | 2020-2023 |  |  | +13.0% (+0.6 to +29.4) | 8 (4-14) | +13.5% (+2.8 to +25.1) | 20 (10-33) |
|  | Dislocation of shoulder | 2020-2023 |  |  | +13.4% (+1.9 to +30.4) | 2 (0-4) | +13.5% (+5.4 to +26.4) | 3 (1-6) |
|  | Fracture of hand (wrist and other distal part of hand) | 2020-2023 |  |  | +13.2% (+1.6 to +30.2) | 6 (3-12) | +13.5% (+4.4 to +25.0) | 14 (7-27) |
|  | Amputation of fingers (excluding thumb) | 2020-2023 |  |  | +13.0% (+2.1 to +31.2) | 3 (1-6) | +13.5% (+4.7 to +26.8) | 5 (2-11) |
|  | Open wound(s) | 2020-2023 |  |  | +13.0% (+1.5 to +31.8) | 86 (50-135) | +13.5% (+3.7 to +25.5) | 183 (105-284) |
|  | Nerve injury | 2020-2023 |  |  | +13.2% (+2.4 to +31.8) | 3 (1-6) | +13.4% (+4.4 to +25.5) | 5 (2-12) |
|  | Spinal cord lesion below neck level | 2020-2023 | +13.7% (+0.1 to +26.6) | 0 (0-0) | +13.1% (+1.4 to +30.5) | 0 (0-0) | +13.4% (+5.1 to +25.3) | 0 (0-1) |
|  | Foreign body in ear | 2020-2023 |  |  | +13.1% (+1.2 to +31.3) | 1 (0-3) | +13.4% (+4.3 to +25.9) | 2 (1-5) |
|  | Lower airway burns | 2020-2023 |  |  | +13.3% (+1.5 to +31.8) | 0 (0-0) | +13.4% (+4.9 to +25.6) | 0 (0-0) |
|  | Fracture of foot bones except ankle | 2020-2023 |  |  | +13.1% (+1.2 to +32.2) | 3 (1-7) | +13.4% (+3.8 to +24.7) | 7 (3-13) |
|  | Effect of different environmental factors | 2020-2023 |  |  | +13.2% (+1.1 to +29.7) | 2 (1-6) | +13.4% (+4.4 to +26.1) | 4 (1-9) |
|  | Fracture of vertebral column | 2020-2023 |  |  | +13.1% (+1.9 to +30.2) | 3 (1-6) | +13.4% (+4.9 to +24.6) | 5 (2-10) |
|  | Fracture of femur, other than femoral neck | 2020-2023 |  |  | +13.2% (+1.2 to +31.5) | 6 (3-14) | +13.4% (+5.4 to +26.1) | 12 (5-25) |
|  | Dislocation of hip | 2020-2023 |  |  | +13.2% (+1.1 to +31.1) | 1 (0-3) | +13.4% (+5.1 to +28.2) | 2 (1-5) |
|  | Injury to eyes | 2020-2023 |  |  | +13.0% (+0.8 to +30.1) | 9 (4-18) | +13.4% (+3.9 to +25.0) | 19 (9-34) |
|  | Asphyxiation | 2020-2023 | +13.7% (+0.4 to +26.6) | 1 (0-2) | +13.3% (+2.1 to +30.5) | 1 (0-3) | +13.4% (+4.9 to +25.0) | 2 (1-5) |
|  | Fracture of radius and/or ulna | 2020-2023 |  |  | +13.2% (+1.7 to +31.1) | 13 (6-24) | +13.4% (+4.8 to +26.0) | 23 (10-42) |
|  | Fracture of patella, tibia or fibula, or ankle | 2020-2023 |  |  | +13.0% (+1.3 to +29.5) | 10 (4-17) | +13.4% (+5.0 to +24.6) | 20 (10-35) |
|  | Foreign body in respiratory system | 2020-2023 |  |  | +13.2% (+0.6 to +29.6) | 2 (1-4) | +13.4% (+5.2 to +24.8) | 3 (1-7) |
|  | Dislocation of knee | 2020-2023 |  |  | +13.3% (+2.1 to +31.0) | 1 (0-4) | +13.4% (+5.4 to +25.8) | 2 (1-6) |
|  | Complications following therapeutic procedures | 2020-2023 |  |  | +13.3% (+1.9 to +29.8) | 5 (2-10) | +13.3% (+5.9 to +25.5) | 8 (4-16) |
|  | Amputation of thumb | 2020-2023 |  |  | +13.1% (+1.4 to +32.4) | 1 (0-4) | +13.3% (+3.8 to +25.5) | 2 (1-6) |
|  | Minor TBI | 2020-2023 |  |  | +13.1% (+1.1 to +31.1) | 6 (3-12) | +13.3% (+4.5 to +25.0) | 13 (6-22) |
|  | Fracture of pelvis | 2020-2023 |  |  | +13.2% (+0.6 to +31.8) | 1 (0-3) | +13.3% (+4.1 to +27.3) | 2 (1-5) |
|  | Muscle and tendon injuries, including sprains and strains lesser dislocations | 2020-2023 |  |  | +13.1% (+2.8 to +29.7) | 26 (14-45) | +13.3% (+4.8 to +25.7) | 45 (25-79) |
|  | Amputation of toe/toes | 2020-2023 |  |  | +13.1% (+1.4 to +30.6) | 1 (0-3) | +13.3% (+4.9 to +24.7) | 2 (1-5) |
|  | Fracture of clavicle, scapula, or humerus | 2020-2023 |  |  | +13.1% (+1.3 to +32.1) | 7 (3-15) | +13.3% (+4.9 to +25.8) | 13 (6-26) |
|  | Foreign body in GI and urogenital system | 2020-2023 |  |  | +13.0% (+0.2 to +30.1) | 2 (1-5) | +13.3% (+3.7 to +25.3) | 3 (1-8) |
|  | Burns, >=20% total burned surface area or >= 10% burned surface area if head/neck or hands/wrist involved w/o lower airway burns | 2020-2023 |  |  | +13.2% (+2.1 to +28.5) | 2 (1-3) | +13.3% (+5.1 to +25.1) | 3 (1-5) |
|  | Superficial injury of any part of the body | 2020-2023 |  |  | +13.0% (+1.5 to +31.4) | 22 (10-39) | +13.3% (+4.8 to +26.6) | 38 (19-66) |
|  | Amputation of lower limbs, bilateral | 2020-2023 |  |  | +13.0% (+0.3 to +30.4) | 0 (0-0) | +13.2% (+4.6 to +25.5) | 0 (0-0) |
|  | Contusion in any part of the body | 2020-2023 |  |  | +13.1% (+1.8 to +30.5) | 12 (6-22) | +13.2% (+4.6 to +25.1) | 21 (10-36) |
|  | Drowning and nonfatal submersion | 2020-2023 |  |  | +13.0% (+2.2 to +31.7) | 1 (0-3) | +13.1% (+4.1 to +25.6) | 2 (1-6) |
|  | Poisoning requiring urgent care | 2020-2023 |  |  | +13.1% (+1.9 to +31.0) | 18 (8-31) | +13.1% (+4.9 to +27.3) | 27 (13-47) |
|  | Burns, <20% total burned surface area without lower airway burns | 2020-2023 |  |  | +12.8% (+1.1 to +31.6) | 11 (5-23) | +13.1% (+4.3 to +25.9) | 19 (8-38) |

## Luxembourg

| **Age** | **Category** | **Period** | **Male %Change** | **Male Incidence 2023** | **Female %Change** | **Female Incidence 2023** | **Both %Change** | **Both Incidence 2023** |
| --- | --- | --- | --- | --- | --- | --- | --- | --- |
| 5-14 years | Foreign body in respiratory system | 2020-2023 |  |  |  |  | +10.2% (+2.7 to +17.4) | 1 (0-1) |
|  | Amputation of upper limb, unilateral | 2020-2023 |  |  |  |  | +10.1% (+1.5 to +20.3) | 0 (0-0) |
|  | Dislocation of knee | 2020-2023 |  |  |  |  | +10.1% (+2.7 to +17.8) | 1 (0-2) |
|  | Foreign body in GI and urogenital system | 2020-2023 |  |  |  |  | +10.1% (+1.7 to +20.7) | 1 (0-2) |
|  | Amputation of upper limbs, bilateral | 2020-2023 |  |  |  |  | +10.1% (+1.5 to +20.4) | 0 (0-0) |
|  | Burns, <20% total burned surface area without lower airway burns | 2020-2023 |  |  |  |  | +10.1% (+1.2 to +19.6) | 3 (1-5) |
|  | Fracture of foot bones except ankle | 2020-2023 |  |  |  |  | +10.1% (+2.2 to +19.2) | 1 (1-3) |
|  | Effect of different environmental factors | 2020-2023 |  |  |  |  | +10.1% (+2.6 to +19.0) | 1 (0-2) |
|  | Fracture of face bones | 2020-2023 |  |  |  |  | +10.1% (+2.2 to +19.3) | 8 (4-14) |
|  | Fracture of pelvis | 2020-2023 |  |  |  |  | +10.1% (+1.7 to +19.1) | 1 (0-2) |
|  | Asphyxiation | 2020-2023 | +10.5% (+0.2 to +23.6) | 0 (0-1) |  |  | +10.1% (+2.1 to +19.1) | 1 (0-3) |
|  | Injury to eyes | 2020-2023 |  |  |  |  | +10.0% (+2.8 to +18.6) | 6 (3-11) |
|  | Amputation of fingers (excluding thumb) | 2020-2023 |  |  |  |  | +10.0% (+1.6 to +20.3) | 1 (0-1) |
|  | Moderate/Severe TBI | 2020-2023 |  |  |  |  | +10.0% (+2.7 to +18.7) | 1 (1-2) |
|  | Nerve injury | 2020-2023 |  |  |  |  | +10.0% (+1.2 to +19.3) | 2 (1-4) |
|  | Fracture of sternum and/or fracture of one or more ribs | 2020-2023 |  |  |  |  | +10.0% (+1.5 to +18.8) | 2 (1-4) |
|  | Fracture of vertebral column | 2020-2023 |  |  |  |  | +10.0% (+2.0 to +19.0) | 1 (0-2) |
|  | Amputation of thumb | 2020-2023 |  |  |  |  | +10.0% (+2.3 to +20.2) | 1 (0-2) |
|  | Crush injury | 2020-2023 |  |  |  |  | +10.0% (+2.1 to +19.2) | 0 (0-0) |
|  | Fracture of radius and/or ulna | 2020-2023 |  |  |  |  | +10.0% (+1.8 to +18.5) | 2 (1-4) |
|  | Minor TBI | 2020-2023 |  |  |  |  | +10.0% (+2.1 to +20.1) | 5 (2-9) |
|  | Spinal cord lesion at neck level | 2020-2023 |  |  |  |  | +10.0% (+3.2 to +19.5) | 0 (0-0) |
|  | Fracture of hand (wrist and other distal part of hand) | 2020-2023 |  |  |  |  | +10.0% (+2.2 to +19.8) | 7 (3-13) |
|  | Fracture of hip | 2020-2023 |  |  |  |  | +10.0% (+2.3 to +18.6) | 0 (0-0) |
|  | Burns, >=20% total burned surface area or >= 10% burned surface area if head/neck or hands/wrist involved w/o lower airway burns | 2020-2023 |  |  |  |  | +10.0% (+2.3 to +18.8) | 0 (0-0) |
|  | Open wound(s) | 2020-2023 |  |  |  |  | +10.0% (+2.2 to +19.1) | 33 (18-53) |
|  | Dislocation of hip | 2020-2023 |  |  |  |  | +10.0% (+1.6 to +19.2) | 1 (0-1) |
|  | Foreign body in ear | 2020-2023 |  |  |  |  | +10.0% (+2.5 to +19.2) | 1 (0-2) |
|  | Multiple fractures, dislocations, crashes, wounds, pains, and strains | 2020-2023 |  |  |  |  | +10.0% (+2.5 to +18.6) | 1 (0-2) |
|  | Amputation of lower limbs, bilateral | 2020-2023 |  |  |  |  | +10.0% (+0.9 to +19.6) | 0 (0-0) |
|  | Fracture of skull | 2020-2023 |  |  |  |  | +10.0% (+1.6 to +19.9) | 6 (3-10) |
|  | Amputation of lower limb, unilateral | 2020-2023 |  |  |  |  | +10.0% (+1.1 to +19.7) | 0 (0-0) |
|  | Superficial injury of any part of the body | 2020-2023 |  |  |  |  | +10.0% (+1.9 to +18.6) | 22 (11-38) |
|  | Muscle and tendon injuries, including sprains and strains lesser dislocations | 2020-2023 |  |  |  |  | +10.0% (+2.9 to +17.2) | 6 (3-10) |
|  | Fracture of femur, other than femoral neck | 2020-2023 |  |  |  |  | +10.0% (+2.2 to +18.3) | 1 (0-2) |
|  | Severe chest Injury | 2020-2023 |  |  |  |  | +10.0% (+0.9 to +20.2) | 3 (2-6) |
|  | Complications following therapeutic procedures | 2020-2023 | +10.3% (+0.1 to +22.4) | 0 (0-0) |  |  | +10.0% (+2.1 to +18.0) | 0 (0-1) |
|  | Internal hemorrhage in abdomen and pelvis | 2020-2023 |  |  |  |  | +10.0% (+2.3 to +18.3) | 4 (2-7) |
|  | Lower airway burns | 2020-2023 |  |  |  |  | +10.0% (+1.7 to +21.6) | 0 (0-0) |
|  | Fracture of clavicle, scapula, or humerus | 2020-2023 |  |  |  |  | +10.0% (+2.5 to +19.7) | 3 (1-6) |
|  | Fracture of patella, tibia or fibula, or ankle | 2020-2023 | +10.3% (+0.1 to +23.9) | 2 (1-3) |  |  | +10.0% (+3.1 to +19.2) | 3 (1-5) |
|  | Poisoning requiring urgent care | 2020-2023 |  |  |  |  | +10.0% (+1.8 to +18.0) | 2 (1-3) |
|  | Spinal cord lesion below neck level | 2020-2023 |  |  |  |  | +9.9% (+1.5 to +19.2) | 0 (0-0) |
|  | Contusion in any part of the body | 2020-2023 |  |  |  |  | +9.9% (+3.0 to +18.2) | 18 (10-31) |
|  | Amputation of toe/toes | 2020-2023 |  |  |  |  | +9.9% (+2.0 to +19.0) | 1 (0-1) |
|  | Dislocation of shoulder | 2020-2023 |  |  |  |  | +9.9% (+1.5 to +18.3) | 1 (0-3) |
|  | Drowning and nonfatal submersion | 2020-2023 |  |  |  |  | +9.8% (+1.1 to +18.8) | 1 (0-2) |
| 15-19 years | Fracture of skull | 2020-2023 |  |  |  |  | +9.6% (+0.2 to +20.8) | 3 (1-5) |
|  | Internal hemorrhage in abdomen and pelvis | 2020-2023 |  |  |  |  | +9.6% (+0.1 to +20.8) | 2 (1-3) |
|  | Moderate/Severe TBI | 2020-2023 |  |  |  |  | +9.2% (+0.2 to +20.5) | 1 (0-1) |
|  | Fracture of hand (wrist and other distal part of hand) | 2020-2023 |  |  |  |  | +9.1% (+0.4 to +19.4) | 3 (1-5) |
|  | Multiple fractures, dislocations, crashes, wounds, pains, and strains | 2020-2023 |  |  |  |  | +8.8% (+0.6 to +19.1) | 0 (0-1) |
|  | Nerve injury | 2020-2023 |  |  |  |  | +8.8% (+0.5 to +18.8) | 1 (0-2) |
|  | Complications following therapeutic procedures | 2020-2023 |  |  |  |  | +8.3% (+0.1 to +17.7) | 0 (0-0) |

## Malta

| **Age** | **Category** | **Period** | **Male %Change** | **Male Incidence 2023** | **Female %Change** | **Female Incidence 2023** | **Both %Change** | **Both Incidence 2023** |
| --- | --- | --- | --- | --- | --- | --- | --- | --- |
| 5-14 years | Fracture of skull | 2020-2023 | +12.1% (+0.4 to +25.7) | 3 (2-6) |  |  | +11.5% (+2.4 to +21.9) | 5 (2-9) |
|  | Dislocation of knee | 2020-2023 | +12.2% (+1.1 to +24.6) | 0 (0-1) |  |  | +11.5% (+2.6 to +22.9) | 1 (0-2) |
|  | Internal hemorrhage in abdomen and pelvis | 2020-2023 | +12.1% (+1.1 to +24.5) | 3 (1-4) |  |  | +11.5% (+2.7 to +21.2) | 4 (2-6) |
|  | Crush injury | 2020-2023 | +12.3% (+0.4 to +25.8) | 0 (0-0) |  |  | +11.5% (+2.4 to +22.1) | 0 (0-0) |
|  | Moderate/Severe TBI | 2020-2023 | +12.2% (+1.8 to +25.4) | 1 (0-1) |  |  | +11.5% (+2.1 to +20.7) | 1 (1-2) |
|  | Foreign body in GI and urogenital system | 2020-2023 |  |  |  |  | +11.5% (+2.1 to +22.8) | 1 (0-1) |
|  | Fracture of hand (wrist and other distal part of hand) | 2020-2023 | +12.2% (+1.0 to +25.9) | 3 (2-6) |  |  | +11.5% (+2.8 to +22.1) | 6 (3-11) |
|  | Severe chest Injury | 2020-2023 | +12.1% (+0.4 to +25.5) | 2 (1-3) |  |  | +11.5% (+1.6 to +21.4) | 3 (1-5) |
|  | Fracture of foot bones except ankle | 2020-2023 | +12.2% (+0.7 to +26.3) | 1 (0-1) |  |  | +11.5% (+2.7 to +23.2) | 1 (0-2) |
|  | Nerve injury | 2020-2023 |  |  |  |  | +11.5% (+2.1 to +21.7) | 1 (1-3) |
|  | Fracture of face bones | 2020-2023 | +12.1% (+0.4 to +24.8) | 5 (2-7) |  |  | +11.5% (+1.6 to +21.7) | 7 (3-12) |
|  | Fracture of patella, tibia or fibula, or ankle | 2020-2023 | +12.2% (+0.3 to +24.3) | 1 (1-2) |  |  | +11.4% (+2.8 to +21.5) | 2 (1-4) |
|  | Spinal cord lesion at neck level | 2020-2023 | +12.4% (+1.2 to +25.4) | 0 (0-0) |  |  | +11.4% (+2.5 to +22.2) | 0 (0-0) |
|  | Open wound(s) | 2020-2023 | +12.2% (+1.6 to +24.9) | 16 (9-26) |  |  | +11.4% (+2.2 to +21.4) | 28 (16-46) |
|  | Spinal cord lesion below neck level | 2020-2023 | +12.0% (+0.6 to +24.5) | 0 (0-0) |  |  | +11.4% (+2.0 to +20.5) | 0 (0-0) |
|  | Fracture of vertebral column | 2020-2023 |  |  |  |  | +11.4% (+1.4 to +21.9) | 1 (0-2) |
|  | Injury to eyes | 2020-2023 | +12.1% (+0.0 to +25.3) | 3 (1-5) |  |  | +11.4% (+2.3 to +21.3) | 5 (3-10) |
|  | Dislocation of shoulder | 2020-2023 | +12.2% (+1.3 to +24.3) | 0 (0-1) |  |  | +11.4% (+2.5 to +21.7) | 1 (0-3) |
|  | Asphyxiation | 2020-2023 | +12.1% (+0.5 to +24.0) | 0 (0-1) |  |  | +11.4% (+2.4 to +21.7) | 1 (0-2) |
|  | Amputation of upper limb, unilateral | 2020-2023 |  |  |  |  | +11.4% (+2.0 to +22.4) | 0 (0-0) |
|  | Multiple fractures, dislocations, crashes, wounds, pains, and strains | 2020-2023 |  |  |  |  | +11.4% (+1.8 to +21.6) | 1 (0-2) |
|  | Fracture of femur, other than femoral neck | 2020-2023 | +12.2% (+0.5 to +26.6) | 0 (0-1) |  |  | +11.4% (+2.7 to +22.4) | 1 (0-2) |
|  | Amputation of thumb | 2020-2023 |  |  |  |  | +11.4% (+2.4 to +22.9) | 1 (0-2) |
|  | Amputation of fingers (excluding thumb) | 2020-2023 |  |  |  |  | +11.3% (+1.9 to +21.8) | 1 (0-1) |
|  | Amputation of upper limbs, bilateral | 2020-2023 |  |  |  |  | +11.3% (+2.1 to +21.9) | 0 (0-0) |
|  | Fracture of sternum and/or fracture of one or more ribs | 2020-2023 |  |  |  |  | +11.3% (+2.8 to +22.4) | 1 (1-3) |
|  | Minor TBI | 2020-2023 |  |  |  |  | +11.3% (+1.6 to +21.2) | 4 (2-8) |
|  | Fracture of clavicle, scapula, or humerus | 2020-2023 |  |  |  |  | +11.3% (+1.7 to +22.7) | 3 (1-6) |
|  | Amputation of lower limb, unilateral | 2020-2023 | +12.3% (+0.6 to +26.0) | 0 (0-0) |  |  | +11.3% (+2.2 to +22.0) | 0 (0-0) |
|  | Muscle and tendon injuries, including sprains and strains lesser dislocations | 2020-2023 | +12.0% (+1.4 to +24.1) | 2 (1-4) |  |  | +11.3% (+2.4 to +20.6) | 5 (3-9) |
|  | Fracture of hip | 2020-2023 |  |  |  |  | +11.3% (+2.1 to +22.7) | 0 (0-0) |
|  | Fracture of radius and/or ulna | 2020-2023 | +12.1% (+0.2 to +26.6) | 1 (0-2) |  |  | +11.3% (+1.5 to +21.6) | 2 (1-4) |
|  | Foreign body in ear | 2020-2023 |  |  |  |  | +11.3% (+1.8 to +23.0) | 1 (0-2) |
|  | Burns, <20% total burned surface area without lower airway burns | 2020-2023 |  |  |  |  | +11.3% (+1.7 to +22.6) | 2 (1-5) |
|  | Foreign body in respiratory system | 2020-2023 | +12.2% (+0.1 to +26.7) | 0 (0-1) |  |  | +11.3% (+2.6 to +21.2) | 0 (0-1) |
|  | Superficial injury of any part of the body | 2020-2023 |  |  |  |  | +11.3% (+2.0 to +21.8) | 20 (9-32) |
|  | Fracture of pelvis | 2020-2023 |  |  |  |  | +11.3% (+0.9 to +22.2) | 1 (0-2) |
|  | Amputation of lower limbs, bilateral | 2020-2023 | +12.0% (+0.4 to +24.5) | 0 (0-0) |  |  | +11.3% (+2.6 to +22.1) | 0 (0-0) |
|  | Burns, >=20% total burned surface area or >= 10% burned surface area if head/neck or hands/wrist involved w/o lower airway burns | 2020-2023 | +12.3% (+0.8 to +26.0) | 0 (0-0) |  |  | +11.3% (+1.6 to +22.3) | 0 (0-0) |
|  | Contusion in any part of the body | 2020-2023 | +12.1% (+1.1 to +24.9) | 7 (4-12) |  |  | +11.3% (+2.7 to +22.4) | 16 (9-28) |
|  | Amputation of toe/toes | 2020-2023 | +12.0% (+0.9 to +26.2) | 0 (0-1) |  |  | +11.3% (+2.6 to +22.3) | 0 (0-1) |
|  | Complications following therapeutic procedures | 2020-2023 | +12.2% (+1.3 to +24.9) | 0 (0-0) |  |  | +11.2% (+1.3 to +21.7) | 0 (0-0) |
|  | Effect of different environmental factors | 2020-2023 | +12.1% (+1.1 to +26.7) | 0 (0-1) |  |  | +11.2% (+1.8 to +21.1) | 1 (0-2) |
|  | Poisoning requiring urgent care | 2020-2023 | +12.1% (+1.0 to +24.6) | 1 (0-1) |  |  | +11.2% (+2.5 to +21.5) | 2 (1-3) |
|  | Dislocation of hip | 2020-2023 | +11.9% (+0.3 to +27.8) | 0 (0-1) |  |  | +11.2% (+0.9 to +23.3) | 0 (0-1) |
|  | Drowning and nonfatal submersion | 2020-2023 | +12.0% (+0.8 to +26.0) | 0 (0-1) |  |  | +11.2% (+2.0 to +21.7) | 1 (0-2) |
|  | Lower airway burns | 2020-2023 |  |  |  |  | +11.1% (+1.8 to +21.8) | 0 (0-0) |

## Netherlands

| **Age** | **Category** | **Period** | **Male %Change** | **Male Incidence 2023** | **Female %Change** | **Female Incidence 2023** | **Both %Change** | **Both Incidence 2023** |
| --- | --- | --- | --- | --- | --- | --- | --- | --- |
| 5-14 years | Severe chest Injury | 2020-2023 |  |  |  |  | +10.7% (+1.2 to +18.5) | 106 (53-190) |
|  | Fracture of skull | 2020-2023 |  |  |  |  | +10.7% (+1.1 to +20.2) | 182 (84-322) |
|  | Internal hemorrhage in abdomen and pelvis | 2020-2023 | +11.1% (+0.4 to +22.7) | 95 (49-162) |  |  | +10.7% (+2.5 to +19.3) | 139 (70-235) |
|  | Spinal cord lesion at neck level | 2020-2023 | +11.2% (+0.2 to +22.8) | 1 (1-3) |  |  | +10.7% (+1.2 to +18.3) | 2 (1-5) |
|  | Fracture of hand (wrist and other distal part of hand) | 2020-2023 |  |  |  |  | +10.7% (+1.4 to +18.4) | 220 (106-406) |
|  | Moderate/Severe TBI | 2020-2023 | +11.1% (+0.8 to +21.3) | 22 (12-36) |  |  | +10.6% (+1.9 to +18.2) | 37 (20-59) |
|  | Amputation of lower limbs, bilateral | 2020-2023 |  |  |  |  | +10.6% (+1.2 to +18.6) | 1 (0-3) |
|  | Fracture of face bones | 2020-2023 |  |  |  |  | +10.6% (+1.9 to +18.8) | 260 (126-432) |
|  | Injury to eyes | 2020-2023 | +11.0% (+0.0 to +23.7) | 112 (56-193) |  |  | +10.6% (+1.8 to +18.5) | 201 (94-370) |
|  | Fracture of sternum and/or fracture of one or more ribs | 2020-2023 | +11.0% (+1.0 to +22.9) | 35 (14-71) |  |  | +10.6% (+1.9 to +19.5) | 55 (22-116) |
|  | Fracture of vertebral column | 2020-2023 |  |  | +9.9% (+0.3 to +21.9) | 18 (7-40) | +10.5% (+1.5 to +18.4) | 35 (15-76) |
|  | Multiple fractures, dislocations, crashes, wounds, pains, and strains | 2020-2023 | +11.0% (+0.1 to +22.8) | 18 (8-33) |  |  | +10.5% (+1.7 to +18.7) | 33 (14-62) |
|  | Spinal cord lesion below neck level | 2020-2023 | +11.1% (+0.9 to +21.2) | 2 (1-4) |  |  | +10.5% (+1.9 to +19.4) | 4 (1-8) |
|  | Minor TBI | 2020-2023 | +11.1% (+0.3 to +22.7) | 83 (43-147) |  |  | +10.5% (+2.0 to +19.1) | 154 (78-276) |
|  | Fracture of foot bones except ankle | 2020-2023 | +11.0% (+0.0 to +24.6) | 23 (10-49) |  |  | +10.5% (+0.6 to +19.1) | 42 (17-93) |
|  | Open wound(s) | 2020-2023 | +11.1% (+0.3 to +21.9) | 593 (336-955) |  |  | +10.5% (+1.9 to +17.9) | 1,050 (578-1,731) |
|  | Fracture of hip | 2020-2023 | +11.1% (+0.8 to +24.1) | 2 (1-4) |  |  | +10.5% (+0.6 to +19.1) | 5 (2-12) |
|  | Amputation of upper limbs, bilateral | 2020-2023 | +11.1% (+0.2 to +23.7) | 1 (0-1) |  |  | +10.5% (+1.6 to +19.2) | 1 (0-2) |
|  | Amputation of upper limb, unilateral | 2020-2023 |  |  |  |  | +10.5% (+0.6 to +19.2) | 1 (0-3) |
|  | Crush injury | 2020-2023 |  |  |  |  | +10.5% (+2.0 to +18.5) | 1 (0-2) |
|  | Fracture of femur, other than femoral neck | 2020-2023 | +11.0% (+0.5 to +22.1) | 16 (7-33) |  |  | +10.5% (+2.1 to +17.5) | 31 (13-68) |
|  | Fracture of clavicle, scapula, or humerus | 2020-2023 | +11.1% (+0.2 to +22.7) | 51 (23-96) |  |  | +10.5% (+1.0 to +18.3) | 106 (46-207) |
|  | Fracture of patella, tibia or fibula, or ankle | 2020-2023 |  |  |  |  | +10.5% (+1.9 to +18.3) | 92 (43-165) |
|  | Amputation of thumb | 2020-2023 |  |  |  |  | +10.5% (+1.1 to +19.4) | 23 (7-58) |
|  | Foreign body in ear | 2020-2023 |  |  |  |  | +10.5% (+0.4 to +18.6) | 28 (9-68) |
|  | Nerve injury | 2020-2023 |  |  |  |  | +10.5% (+0.4 to +18.1) | 53 (20-119) |
|  | Asphyxiation | 2020-2023 | +10.9% (+1.0 to +23.7) | 14 (4-34) |  |  | +10.5% (+1.0 to +18.5) | 30 (8-81) |
|  | Muscle and tendon injuries, including sprains and strains lesser dislocations | 2020-2023 | +11.1% (+0.1 to +22.3) | 87 (45-151) |  |  | +10.5% (+2.1 to +17.7) | 185 (96-329) |
|  | Amputation of lower limb, unilateral | 2020-2023 |  |  |  |  | +10.5% (+0.9 to +18.8) | 0 (0-0) |
|  | Amputation of fingers (excluding thumb) | 2020-2023 |  |  |  |  | +10.5% (+0.8 to +19.5) | 20 (7-46) |
|  | Fracture of radius and/or ulna | 2020-2023 | +11.0% (+1.0 to +24.2) | 37 (16-66) | +10.0% (+0.1 to +22.2) | 41 (17-80) | +10.4% (+1.4 to +19.3) | 78 (34-149) |
|  | Superficial injury of any part of the body | 2020-2023 |  |  |  |  | +10.4% (+0.7 to +18.5) | 730 (348-1,204) |
|  | Dislocation of shoulder | 2020-2023 | +10.9% (+0.2 to +22.2) | 18 (6-44) |  |  | +10.4% (+1.8 to +18.8) | 37 (12-95) |
|  | Drowning and nonfatal submersion | 2020-2023 | +11.1% (+0.3 to +21.6) | 13 (4-31) | +9.9% (+0.1 to +22.2) | 15 (4-44) | +10.4% (+1.4 to +18.6) | 28 (7-74) |
|  | Foreign body in GI and urogenital system | 2020-2023 |  |  |  |  | +10.4% (+1.7 to +19.0) | 23 (7-55) |
|  | Amputation of toe/toes | 2020-2023 |  |  |  |  | +10.4% (+0.7 to +18.2) | 17 (5-39) |
|  | Foreign body in respiratory system | 2020-2023 | +11.0% (+0.4 to +22.0) | 8 (3-18) |  |  | +10.4% (+1.4 to +18.4) | 17 (6-42) |
|  | Dislocation of knee | 2020-2023 |  |  |  |  | +10.4% (+0.3 to +18.1) | 27 (8-68) |
|  | Burns, >=20% total burned surface area or >= 10% burned surface area if head/neck or hands/wrist involved w/o lower airway burns | 2020-2023 | +11.1% (+0.0 to +22.9) | 1 (1-2) |  |  | +10.4% (+1.6 to +19.1) | 3 (1-6) |
|  | Dislocation of hip | 2020-2023 | +11.0% (+0.4 to +24.0) | 8 (3-19) |  |  | +10.4% (+1.1 to +18.7) | 18 (5-45) |
|  | Fracture of pelvis | 2020-2023 |  |  |  |  | +10.4% (+0.5 to +18.5) | 27 (8-70) |
|  | Lower airway burns | 2020-2023 | +11.0% (+0.2 to +24.6) | 1 (0-1) |  |  | +10.4% (+0.8 to +19.4) | 1 (0-3) |
|  | Burns, <20% total burned surface area without lower airway burns | 2020-2023 |  |  |  |  | +10.3% (+1.2 to +18.8) | 88 (35-172) |
|  | Complications following therapeutic procedures | 2020-2023 | +11.1% (+0.8 to +22.8) | 4 (2-7) |  |  | +10.3% (+1.8 to +18.2) | 9 (4-16) |
|  | Poisoning requiring urgent care | 2020-2023 | +11.0% (+0.0 to +23.0) | 21 (10-37) |  |  | +10.3% (+1.8 to +17.9) | 58 (27-108) |
|  | Effect of different environmental factors | 2020-2023 | +11.1% (+0.1 to +22.7) | 13 (5-30) |  |  | +10.3% (+1.4 to +18.5) | 31 (11-74) |
|  | Contusion in any part of the body | 2020-2023 | +11.0% (+0.6 to +23.6) | 256 (142-443) |  |  | +10.3% (+1.5 to +17.9) | 591 (317-1,059) |
| 15-19 years | Moderate/Severe TBI | 2020-2023 |  |  |  |  | +9.2% (+0.1 to +19.1) | 25 (13-40) |
|  | Nerve injury | 2020-2023 |  |  |  |  | +9.2% (+0.1 to +18.1) | 26 (10-55) |
|  | Fracture of vertebral column | 2020-2023 |  |  |  |  | +9.2% (+0.0 to +18.0) | 19 (8-38) |
|  | Superficial injury of any part of the body | 2020-2023 |  |  |  |  | +9.1% (+0.7 to +18.1) | 361 (192-627) |
|  | Amputation of lower limb, unilateral | 2020-2023 |  |  |  |  | +9.1% (+0.4 to +18.1) | 0 (0-0) |
|  | Fracture of clavicle, scapula, or humerus | 2020-2023 |  |  |  |  | +9.1% (+0.4 to +19.6) | 53 (24-104) |
|  | Multiple fractures, dislocations, crashes, wounds, pains, and strains | 2020-2023 |  |  |  |  | +9.1% (+0.6 to +20.1) | 18 (8-35) |
|  | Poisoning requiring urgent care | 2020-2023 |  |  |  |  | +9.0% (+0.0 to +18.7) | 28 (14-53) |
|  | Foreign body in respiratory system | 2020-2023 |  |  |  |  | +8.9% (+0.0 to +18.4) | 9 (3-20) |

## Poland

| **Age** | **Category** | **Period** | **Male %Change** | **Male Incidence 2023** | **Female %Change** | **Female Incidence 2023** | **Both %Change** | **Both Incidence 2023** |
| --- | --- | --- | --- | --- | --- | --- | --- | --- |
| <5 years | Fracture of hip | 2020-2023 | +15.1% (+9.7 to +20.0) | 5 (2-10) | +16.0% (+11.6 to +20.1) | 9 (4-20) | +15.7% (+11.6 to +18.9) | 14 (6-29) |
|  | Complications following therapeutic procedures | 2020-2023 | +15.1% (+9.3 to +19.4) | 58 (33-97) | +15.8% (+11.8 to +20.5) | 88 (45-165) | +15.5% (+11.7 to +19.0) | 146 (81-263) |
|  | Poisoning requiring urgent care | 2020-2023 | +15.0% (+9.4 to +19.7) | 182 (104-299) | +15.8% (+11.2 to +20.4) | 354 (185-603) | +15.5% (+11.5 to +18.8) | 536 (289-903) |
|  | Fracture of pelvis | 2020-2023 | +15.0% (+9.4 to +19.8) | 14 (6-35) | +15.8% (+11.7 to +20.3) | 22 (7-60) | +15.5% (+11.8 to +18.6) | 36 (13-93) |
|  | Dislocation of hip | 2020-2023 | +15.0% (+9.3 to +20.1) | 19 (7-41) | +15.9% (+11.4 to +20.2) | 25 (8-61) | +15.5% (+11.5 to +18.9) | 43 (15-100) |
|  | Dislocation of knee | 2020-2023 | +15.0% (+9.2 to +19.7) | 20 (8-45) | +15.8% (+11.0 to +20.1) | 28 (8-72) | +15.5% (+11.4 to +18.8) | 48 (16-115) |
|  | Contusion in any part of the body | 2020-2023 | +15.0% (+9.7 to +19.8) | 169 (104-266) | +15.8% (+11.6 to +20.2) | 249 (139-436) | +15.5% (+11.9 to +18.6) | 419 (244-690) |
|  | Superficial injury of any part of the body | 2020-2023 | +15.0% (+9.0 to +19.7) | 318 (190-478) | +15.8% (+11.8 to +19.9) | 432 (241-682) | +15.5% (+11.5 to +18.6) | 751 (425-1,159) |
|  | Amputation of thumb | 2020-2023 | +15.0% (+8.4 to +20.4) | 23 (10-49) | +15.9% (+11.0 to +21.1) | 27 (9-64) | +15.5% (+11.1 to +19.4) | 50 (19-111) |
|  | Burns, <20% total burned surface area without lower airway burns | 2020-2023 | +15.0% (+9.0 to +20.0) | 171 (88-305) | +15.8% (+11.2 to +21.0) | 251 (111-477) | +15.5% (+11.2 to +19.2) | 422 (208-754) |
|  | Foreign body in respiratory system | 2020-2023 | +15.0% (+8.9 to +19.5) | 26 (11-55) | +15.8% (+11.4 to +20.5) | 33 (11-79) | +15.5% (+11.2 to +18.9) | 60 (23-132) |
|  | Fracture of vertebral column | 2020-2023 | +15.0% (+9.1 to +19.9) | 46 (23-85) | +15.9% (+11.2 to +20.0) | 53 (20-111) | +15.5% (+11.7 to +18.9) | 99 (45-195) |
|  | Dislocation of shoulder | 2020-2023 | +15.0% (+9.1 to +20.0) | 24 (11-46) | +15.8% (+11.5 to +20.2) | 30 (11-68) | +15.5% (+12.1 to +19.2) | 55 (22-110) |
|  | Effect of different environmental factors | 2020-2023 | +14.9% (+9.2 to +19.7) | 33 (15-69) | +15.8% (+11.2 to +20.0) | 48 (19-117) | +15.4% (+11.7 to +18.5) | 81 (34-184) |
|  | Fracture of radius and/or ulna | 2020-2023 | +15.0% (+9.3 to +19.6) | 190 (107-315) | +15.8% (+11.6 to +20.4) | 245 (120-425) | +15.4% (+11.3 to +18.6) | 435 (229-728) |
|  | Muscle and tendon injuries, including sprains and strains lesser dislocations | 2020-2023 | +15.0% (+9.7 to +19.8) | 402 (253-600) | +15.8% (+11.6 to +20.3) | 513 (301-808) | +15.4% (+11.6 to +18.9) | 915 (547-1,392) |
|  | Fracture of femur, other than femoral neck | 2020-2023 | +15.0% (+9.3 to +20.1) | 112 (56-209) | +15.8% (+11.6 to +20.3) | 123 (52-244) | +15.4% (+11.6 to +19.2) | 235 (108-443) |
|  | Foreign body in GI and urogenital system | 2020-2023 | +15.0% (+9.0 to +19.8) | 34 (14-70) | +15.8% (+11.7 to +20.5) | 38 (12-99) | +15.4% (+11.4 to +18.9) | 71 (26-171) |
|  | Amputation of toe/toes | 2020-2023 | +15.0% (+9.6 to +19.9) | 18 (8-39) | +15.8% (+11.3 to +20.5) | 21 (7-50) | +15.4% (+11.2 to +19.1) | 39 (15-87) |
|  | Drowning and nonfatal submersion | 2020-2023 | +15.0% (+9.0 to +19.6) | 19 (8-40) | +15.7% (+11.3 to +20.2) | 26 (8-73) | +15.4% (+11.9 to +19.3) | 45 (16-108) |
|  | Asphyxiation | 2020-2023 | +15.0% (+9.6 to +19.5) | 17 (7-37) | +15.7% (+11.3 to +20.1) | 22 (7-60) | +15.4% (+11.2 to +18.9) | 39 (14-99) |
|  | Fracture of clavicle, scapula, or humerus | 2020-2023 | +15.0% (+9.5 to +19.7) | 114 (60-210) | +15.8% (+11.4 to +20.1) | 142 (66-274) | +15.4% (+11.3 to +18.8) | 256 (128-486) |
|  | Minor TBI | 2020-2023 | +15.0% (+9.1 to +19.7) | 123 (66-205) | +15.8% (+11.4 to +20.4) | 120 (55-214) | +15.4% (+11.1 to +19.0) | 243 (121-410) |
|  | Fracture of patella, tibia or fibula, or ankle | 2020-2023 | +15.0% (+9.8 to +20.0) | 202 (114-324) | +15.8% (+11.6 to +19.9) | 195 (100-325) | +15.4% (+12.0 to +18.9) | 397 (215-622) |
|  | Nerve injury | 2020-2023 | +15.0% (+9.3 to +19.7) | 57 (27-106) | +15.8% (+11.7 to +20.1) | 58 (23-133) | +15.4% (+11.7 to +18.7) | 115 (50-244) |
|  | Fracture of foot bones except ankle | 2020-2023 | +15.0% (+9.6 to +19.8) | 70 (36-129) | +15.7% (+11.4 to +19.8) | 67 (29-144) | +15.4% (+11.1 to +18.8) | 136 (67-272) |
|  | Multiple fractures, dislocations, crashes, wounds, pains, and strains | 2020-2023 | +15.0% (+9.1 to +19.5) | 115 (62-200) | +15.8% (+11.6 to +20.7) | 111 (54-194) | +15.4% (+11.5 to +19.1) | 226 (117-394) |
|  | Open wound(s) | 2020-2023 | +15.0% (+9.3 to +19.6) | 1,932 (1,302-2,649) | +15.8% (+11.6 to +20.2) | 1,702 (1,089-2,447) | +15.4% (+11.5 to +18.9) | 3,633 (2,394-5,102) |
|  | Amputation of fingers (excluding thumb) | 2020-2023 | +15.0% (+9.3 to +20.2) | 59 (27-112) | +15.8% (+11.2 to +20.5) | 53 (19-118) | +15.4% (+11.3 to +19.0) | 111 (46-230) |
|  | Injury to eyes | 2020-2023 | +15.0% (+9.2 to +19.9) | 203 (117-337) | +15.8% (+11.3 to +20.6) | 184 (93-346) | +15.4% (+11.0 to +19.0) | 387 (215-676) |
|  | Foreign body in ear | 2020-2023 | +14.9% (+9.5 to +19.9) | 21 (9-43) | +15.7% (+11.6 to +20.9) | 25 (8-60) | +15.3% (+11.7 to +19.3) | 46 (17-102) |
|  | Fracture of sternum and/or fracture of one or more ribs | 2020-2023 | +15.0% (+9.3 to +19.5) | 21 (9-41) | +15.8% (+11.4 to +20.5) | 14 (5-31) | +15.3% (+11.0 to +18.7) | 35 (14-72) |
|  | Fracture of hand (wrist and other distal part of hand) | 2020-2023 | +15.0% (+9.7 to +19.9) | 160 (96-268) | +15.8% (+11.2 to +20.3) | 131 (68-234) | +15.3% (+11.1 to +18.8) | 291 (163-499) |
|  | Amputation of lower limb, unilateral | 2020-2023 | +14.8% (+8.8 to +19.4) | 1 (1-2) | +15.7% (+11.1 to +19.8) | 1 (1-3) | +15.3% (+11.3 to +18.8) | 2 (1-4) |
|  | Fracture of face bones | 2020-2023 | +15.0% (+8.9 to +19.6) | 253 (149-402) | +15.8% (+11.3 to +19.9) | 156 (81-273) | +15.3% (+11.0 to +18.8) | 409 (231-683) |
|  | Burns, >=20% total burned surface area or >= 10% burned surface area if head/neck or hands/wrist involved w/o lower airway burns | 2020-2023 | +14.7% (+8.8 to +19.7) | 21 (12-32) | +15.5% (+11.6 to +19.8) | 37 (21-61) | +15.2% (+11.4 to +18.5) | 58 (33-94) |
|  | Internal hemorrhage in abdomen and pelvis | 2020-2023 | +15.0% (+9.4 to +19.8) | 490 (304-741) | +15.8% (+11.3 to +20.4) | 249 (143-424) | +15.2% (+11.0 to +18.7) | 739 (448-1,144) |
|  | Severe chest Injury | 2020-2023 | +15.0% (+9.2 to +19.9) | 292 (174-463) | +15.8% (+11.3 to +19.9) | 139 (73-254) | +15.2% (+10.8 to +19.1) | 432 (251-727) |
|  | Fracture of skull | 2020-2023 | +15.0% (+9.5 to +19.8) | 147 (80-245) | +15.8% (+11.7 to +20.0) | 72 (33-148) | +15.2% (+11.1 to +18.9) | 219 (114-380) |
|  | Amputation of lower limbs, bilateral | 2020-2023 | +14.7% (+9.5 to +19.9) | 1 (0-2) | +15.5% (+10.7 to +20.3) | 1 (0-4) | +15.2% (+11.1 to +18.9) | 3 (1-7) |
|  | Amputation of upper limb, unilateral | 2020-2023 | +14.7% (+9.0 to +19.5) | 2 (1-4) | +15.6% (+10.7 to +20.2) | 2 (1-6) | +15.2% (+10.4 to +18.8) | 4 (2-10) |
|  | Spinal cord lesion at neck level | 2020-2023 | +14.7% (+9.3 to +19.4) | 3 (2-7) | +15.6% (+11.0 to +20.1) | 3 (1-8) | +15.2% (+10.9 to +18.9) | 7 (3-15) |
|  | Lower airway burns | 2020-2023 | +14.7% (+9.1 to +19.8) | 2 (1-3) | +15.5% (+10.9 to +20.5) | 2 (1-5) | +15.2% (+11.1 to +19.1) | 4 (1-9) |
|  | Moderate/Severe TBI | 2020-2023 | +14.7% (+8.9 to +19.9) | 170 (115-237) | +15.6% (+11.3 to +20.4) | 123 (81-182) | +15.1% (+11.0 to +18.9) | 292 (195-420) |
|  | Crush injury | 2020-2023 | +14.7% (+9.0 to +19.3) | 8 (4-15) | +15.6% (+11.3 to +20.4) | 6 (3-13) | +15.1% (+11.0 to +18.6) | 14 (7-28) |
|  | Amputation of upper limbs, bilateral | 2020-2023 | +14.7% (+9.1 to +19.5) | 3 (1-7) | +15.5% (+10.7 to +20.8) | 3 (1-7) | +15.0% (+11.1 to +18.6) | 6 (2-14) |
|  | Spinal cord lesion below neck level | 2020-2023 | +14.7% (+9.0 to +19.0) | 5 (2-9) | +15.4% (+11.5 to +20.0) | 4 (1-9) | +15.0% (+11.0 to +18.3) | 8 (3-18) |
| 5-14 years | Burns, <20% total burned surface area without lower airway burns | 2020-2023 | +15.5% (+11.6 to +18.8) | 133 (62-256) | +15.7% (+12.1 to +19.5) | 167 (64-354) | +15.6% (+12.8 to +18.2) | 301 (123-603) |
|  | Dislocation of knee | 2020-2023 | +15.4% (+11.9 to +18.9) | 17 (6-40) | +15.7% (+10.8 to +19.3) | 20 (5-53) | +15.6% (+12.6 to +18.1) | 37 (12-86) |
|  | Nerve injury | 2020-2023 | +15.5% (+11.8 to +18.9) | 47 (19-103) | +15.6% (+11.2 to +19.3) | 41 (13-90) | +15.5% (+12.3 to +18.3) | 88 (32-191) |
|  | Foreign body in ear | 2020-2023 | +15.5% (+11.7 to +19.0) | 17 (7-38) | +15.6% (+11.5 to +19.7) | 17 (5-44) | +15.5% (+12.5 to +18.2) | 34 (12-81) |
|  | Foreign body in GI and urogenital system | 2020-2023 | +15.4% (+12.1 to +18.9) | 29 (11-60) | +15.6% (+11.8 to +19.1) | 27 (8-71) | +15.5% (+12.6 to +18.0) | 56 (19-126) |
|  | Drowning and nonfatal submersion | 2020-2023 | +15.5% (+12.1 to +18.8) | 16 (6-34) | +15.6% (+11.5 to +19.5) | 18 (5-52) | +15.5% (+12.7 to +18.1) | 33 (11-88) |
|  | Fracture of skull | 2020-2023 | +15.4% (+12.0 to +19.2) | 123 (57-215) | +15.5% (+11.8 to +18.9) | 52 (20-106) | +15.5% (+12.6 to +18.3) | 175 (79-311) |
|  | Fracture of face bones | 2020-2023 | +15.4% (+11.9 to +19.4) | 217 (106-357) | +15.6% (+11.8 to +19.2) | 114 (53-206) | +15.5% (+12.6 to +18.5) | 332 (162-546) |
|  | Internal hemorrhage in abdomen and pelvis | 2020-2023 | +15.4% (+11.7 to +18.6) | 420 (221-660) | +15.6% (+11.6 to +19.0) | 182 (84-307) | +15.5% (+12.7 to +18.2) | 601 (313-976) |
|  | Effect of different environmental factors | 2020-2023 | +15.4% (+11.8 to +19.1) | 27 (10-59) | +15.6% (+11.4 to +19.6) | 34 (11-84) | +15.5% (+12.6 to +18.1) | 61 (23-134) |
|  | Dislocation of hip | 2020-2023 | +15.4% (+11.9 to +19.2) | 16 (6-36) | +15.6% (+11.7 to +19.3) | 18 (5-47) | +15.5% (+12.2 to +18.3) | 34 (11-80) |
|  | Severe chest Injury | 2020-2023 | +15.4% (+12.0 to +18.9) | 254 (135-443) | +15.5% (+11.7 to +18.9) | 103 (49-194) | +15.5% (+12.6 to +18.2) | 358 (188-640) |
|  | Amputation of thumb | 2020-2023 | +15.5% (+11.4 to +18.8) | 19 (7-44) | +15.5% (+11.6 to +19.3) | 19 (6-55) | +15.5% (+12.4 to +18.2) | 39 (14-95) |
|  | Fracture of foot bones except ankle | 2020-2023 | +15.4% (+11.7 to +19.0) | 58 (24-114) | +15.5% (+11.6 to +19.0) | 47 (16-103) | +15.5% (+12.6 to +18.2) | 106 (40-213) |
|  | Asphyxiation | 2020-2023 | +15.5% (+11.5 to +19.4) | 14 (5-31) | +15.5% (+10.9 to +20.0) | 15 (4-41) | +15.5% (+12.3 to +18.2) | 29 (9-69) |
|  | Muscle and tendon injuries, including sprains and strains lesser dislocations | 2020-2023 | +15.4% (+11.8 to +18.7) | 348 (189-578) | +15.5% (+11.5 to +19.0) | 379 (196-671) | +15.5% (+12.4 to +18.0) | 727 (389-1,236) |
|  | Amputation of fingers (excluding thumb) | 2020-2023 | +15.4% (+11.7 to +19.0) | 51 (22-97) | +15.5% (+11.5 to +18.9) | 39 (14-89) | +15.5% (+12.1 to +18.2) | 89 (36-185) |
|  | Amputation of lower limbs, bilateral | 2020-2023 | +15.4% (+12.2 to +18.8) | 1 (0-2) | +15.6% (+11.3 to +19.2) | 1 (0-3) | +15.5% (+12.6 to +18.2) | 2 (1-4) |
|  | Fracture of patella, tibia or fibula, or ankle | 2020-2023 | +15.4% (+11.7 to +18.8) | 179 (88-304) | +15.6% (+11.6 to +19.2) | 147 (66-262) | +15.5% (+12.7 to +18.1) | 327 (155-552) |
|  | Injury to eyes | 2020-2023 | +15.4% (+12.0 to +18.9) | 173 (88-294) | +15.5% (+11.6 to +18.8) | 134 (61-264) | +15.5% (+12.5 to +18.0) | 308 (150-564) |
|  | Open wound(s) | 2020-2023 | +15.4% (+11.8 to +19.1) | 1,700 (934-2,581) | +15.5% (+11.5 to +19.2) | 1,274 (702-2,027) | +15.5% (+12.6 to +18.2) | 2,974 (1,621-4,637) |
|  | Fracture of hand (wrist and other distal part of hand) | 2020-2023 | +15.4% (+11.6 to +18.7) | 139 (68-255) | +15.5% (+11.4 to +19.2) | 96 (43-184) | +15.5% (+12.4 to +18.0) | 235 (113-439) |
|  | Fracture of sternum and/or fracture of one or more ribs | 2020-2023 | +15.4% (+11.8 to +18.4) | 20 (8-42) | +15.5% (+11.3 to +19.0) | 11 (3-27) | +15.5% (+12.7 to +18.2) | 31 (11-69) |
|  | Contusion in any part of the body | 2020-2023 | +15.4% (+11.7 to +19.0) | 147 (77-252) | +15.5% (+11.7 to +19.1) | 184 (85-337) | +15.4% (+12.3 to +18.2) | 330 (165-583) |
|  | Amputation of toe/toes | 2020-2023 | +15.4% (+12.3 to +18.9) | 16 (6-35) | +15.5% (+11.2 to +19.7) | 16 (4-39) | +15.4% (+12.4 to +18.2) | 31 (11-74) |
|  | Amputation of upper limbs, bilateral | 2020-2023 | +15.4% (+11.5 to +18.8) | 3 (1-6) | +15.5% (+11.7 to +19.0) | 2 (1-5) | +15.4% (+12.6 to +18.4) | 5 (2-11) |
|  | Superficial injury of any part of the body | 2020-2023 | +15.4% (+11.8 to +18.8) | 285 (142-479) | +15.5% (+11.6 to +19.0) | 329 (157-589) | +15.4% (+12.4 to +18.0) | 613 (299-1,085) |
|  | Fracture of femur, other than femoral neck | 2020-2023 | +15.3% (+11.8 to +18.6) | 100 (46-206) | +15.5% (+11.7 to +19.1) | 94 (40-210) | +15.4% (+12.6 to +18.1) | 193 (86-413) |
|  | Fracture of clavicle, scapula, or humerus | 2020-2023 | +15.4% (+11.9 to +18.7) | 102 (47-198) | +15.5% (+11.6 to +18.9) | 108 (45-226) | +15.4% (+12.6 to +17.9) | 210 (92-416) |
|  | Dislocation of shoulder | 2020-2023 | +15.4% (+12.0 to +19.1) | 22 (9-45) | +15.5% (+11.5 to +19.1) | 23 (7-57) | +15.4% (+12.5 to +18.1) | 45 (16-102) |
|  | Poisoning requiring urgent care | 2020-2023 | +15.4% (+12.0 to +18.7) | 158 (78-269) | +15.5% (+11.5 to +19.2) | 262 (124-475) | +15.4% (+12.3 to +17.9) | 420 (203-744) |
|  | Multiple fractures, dislocations, crashes, wounds, pains, and strains | 2020-2023 | +15.4% (+11.9 to +18.4) | 105 (52-185) | +15.5% (+11.8 to +19.0) | 86 (36-160) | +15.4% (+12.5 to +18.0) | 191 (88-331) |
|  | Minor TBI | 2020-2023 | +15.4% (+11.7 to +18.9) | 112 (55-198) | +15.5% (+11.6 to +19.1) | 93 (42-170) | +15.4% (+12.5 to +18.3) | 205 (101-363) |
|  | Spinal cord lesion below neck level | 2020-2023 | +15.3% (+11.9 to +18.8) | 4 (1-7) | +15.5% (+11.5 to +18.8) | 3 (1-7) | +15.4% (+12.6 to +18.0) | 6 (2-14) |
|  | Lower airway burns | 2020-2023 | +15.4% (+11.9 to +19.2) | 1 (0-3) | +15.4% (+11.2 to +19.2) | 1 (0-4) | +15.4% (+12.6 to +18.1) | 3 (1-6) |
|  | Fracture of radius and/or ulna | 2020-2023 | +15.4% (+11.6 to +18.5) | 172 (84-307) | +15.5% (+11.5 to +19.0) | 189 (81-348) | +15.4% (+12.2 to +18.1) | 361 (170-654) |
|  | Fracture of vertebral column | 2020-2023 | +15.3% (+11.9 to +18.8) | 42 (18-78) | +15.5% (+11.5 to +19.0) | 41 (16-85) | +15.4% (+12.3 to +18.1) | 82 (35-160) |
|  | Foreign body in respiratory system | 2020-2023 | +15.4% (+11.9 to +18.8) | 23 (9-50) | +15.4% (+11.7 to +19.1) | 25 (9-59) | +15.4% (+12.5 to +18.3) | 48 (18-109) |
|  | Burns, >=20% total burned surface area or >= 10% burned surface area if head/neck or hands/wrist involved w/o lower airway burns | 2020-2023 | +15.3% (+12.1 to +18.6) | 17 (9-29) | +15.5% (+11.4 to +19.1) | 26 (11-46) | +15.4% (+12.3 to +17.8) | 43 (20-74) |
|  | Amputation of upper limb, unilateral | 2020-2023 | +15.3% (+11.5 to +19.3) | 2 (1-4) | +15.4% (+11.5 to +19.3) | 2 (0-4) | +15.4% (+12.5 to +18.4) | 3 (1-8) |
|  | Fracture of pelvis | 2020-2023 | +15.3% (+11.7 to +19.2) | 13 (5-30) | +15.4% (+11.3 to +19.2) | 18 (5-50) | +15.3% (+12.2 to +18.1) | 32 (10-80) |
|  | Spinal cord lesion at neck level | 2020-2023 | +15.3% (+12.0 to +18.6) | 3 (1-6) | +15.4% (+11.4 to +18.8) | 2 (1-5) | +15.3% (+12.7 to +17.8) | 5 (2-11) |
|  | Crush injury | 2020-2023 | +15.3% (+11.9 to +18.5) | 7 (3-13) | +15.4% (+11.5 to +18.7) | 5 (2-10) | +15.3% (+12.3 to +18.0) | 12 (5-21) |
|  | Complications following therapeutic procedures | 2020-2023 | +15.2% (+12.0 to +18.4) | 59 (30-105) | +15.4% (+11.7 to +18.8) | 75 (35-147) | +15.3% (+12.5 to +17.6) | 135 (67-248) |
|  | Moderate/Severe TBI | 2020-2023 | +15.2% (+12.2 to +18.5) | 153 (89-243) | +15.4% (+11.7 to +18.8) | 94 (53-150) | +15.3% (+12.8 to +17.9) | 247 (144-388) |
|  | Fracture of hip | 2020-2023 | +15.0% (+11.8 to +18.7) | 6 (3-12) | +15.2% (+11.1 to +18.6) | 9 (4-21) | +15.1% (+12.0 to +17.8) | 16 (6-33) |
|  | Amputation of lower limb, unilateral | 2020-2023 | +15.0% (+11.7 to +18.2) | 1 (1-2) | +15.2% (+11.3 to +18.5) | 1 (1-3) | +15.1% (+12.1 to +17.5) | 3 (1-5) |
| 15-19 years | Poisoning requiring urgent care | 2020-2023 |  |  | +8.9% (+4.1 to +13.5) | 69 (30-131) | +7.4% (+3.2 to +11.2) | 131 (64-241) |
|  | Burns, >=20% total burned surface area or >= 10% burned surface area if head/neck or hands/wrist involved w/o lower airway burns | 2020-2023 |  |  | +8.9% (+4.6 to +13.3) | 8 (4-15) | +7.4% (+3.4 to +11.3) | 16 (8-28) |
|  | Fracture of hip | 2020-2023 |  |  | +8.9% (+4.0 to +13.4) | 4 (2-9) | +7.4% (+2.4 to +11.4) | 8 (3-17) |
|  | Fracture of pelvis | 2020-2023 | +6.0% (+0.3 to +10.6) | 6 (2-15) | +8.8% (+4.3 to +13.7) | 5 (2-14) | +7.3% (+3.0 to +10.7) | 11 (4-30) |
|  | Complications following therapeutic procedures | 2020-2023 | +5.9% (+0.3 to +10.8) | 28 (15-49) | +8.9% (+4.1 to +12.9) | 24 (11-46) | +7.3% (+3.6 to +11.0) | 53 (27-95) |
|  | Dislocation of knee | 2020-2023 |  |  | +8.9% (+4.3 to +13.2) | 5 (2-12) | +7.3% (+2.2 to +11.1) | 11 (4-25) |
|  | Effect of different environmental factors | 2020-2023 |  |  | +8.9% (+4.4 to +12.9) | 8 (3-21) | +7.3% (+2.7 to +10.8) | 18 (6-40) |
|  | Contusion in any part of the body | 2020-2023 |  |  | +8.9% (+4.5 to +13.2) | 47 (21-83) | +7.2% (+2.7 to +11.0) | 103 (51-170) |
|  | Dislocation of hip | 2020-2023 |  |  | +8.9% (+4.5 to +12.5) | 5 (1-12) | +7.2% (+2.3 to +10.8) | 11 (4-25) |
|  | Burns, <20% total burned surface area without lower airway burns | 2020-2023 |  |  | +8.9% (+4.0 to +13.1) | 39 (15-83) | +7.2% (+2.0 to +11.1) | 84 (37-172) |
|  | Superficial injury of any part of the body | 2020-2023 |  |  | +8.9% (+4.3 to +13.5) | 88 (44-159) | +7.2% (+2.7 to +11.2) | 201 (103-351) |
|  | Fracture of radius and/or ulna | 2020-2023 |  |  | +8.9% (+4.1 to +13.2) | 53 (23-101) | +7.2% (+3.0 to +11.3) | 123 (57-233) |
|  | Fracture of clavicle, scapula, or humerus | 2020-2023 |  |  | +8.9% (+4.2 to +13.0) | 29 (13-58) | +7.2% (+2.2 to +11.0) | 70 (32-122) |
|  | Dislocation of shoulder | 2020-2023 |  |  | +8.9% (+4.5 to +13.2) | 6 (2-16) | +7.2% (+2.5 to +10.9) | 15 (5-35) |
|  | Asphyxiation | 2020-2023 |  |  | +8.9% (+4.5 to +13.4) | 4 (1-10) | +7.2% (+2.8 to +11.1) | 9 (3-21) |
|  | Muscle and tendon injuries, including sprains and strains lesser dislocations | 2020-2023 |  |  | +8.9% (+4.0 to +13.6) | 99 (46-173) | +7.2% (+2.5 to +10.9) | 234 (120-394) |
|  | Amputation of lower limbs, bilateral | 2020-2023 |  |  | +9.0% (+4.0 to +13.1) | 0 (0-1) | +7.2% (+2.8 to +11.0) | 1 (0-2) |
|  | Drowning and nonfatal submersion | 2020-2023 |  |  | +8.8% (+4.6 to +12.6) | 4 (1-12) | +7.2% (+2.7 to +10.7) | 10 (3-25) |
|  | Lower airway burns | 2020-2023 |  |  | +8.9% (+3.7 to +13.9) | 0 (0-1) | +7.2% (+2.3 to +11.1) | 1 (0-2) |
|  | Amputation of lower limb, unilateral | 2020-2023 |  |  | +8.9% (+4.0 to +13.5) | 1 (0-1) | +7.2% (+2.5 to +11.0) | 2 (1-3) |
|  | Foreign body in respiratory system | 2020-2023 |  |  | +8.8% (+4.1 to +13.5) | 7 (2-16) | +7.2% (+2.0 to +11.9) | 16 (6-37) |
|  | Fracture of vertebral column | 2020-2023 |  |  | +8.9% (+4.3 to +13.8) | 11 (4-26) | +7.2% (+2.1 to +11.0) | 29 (11-60) |
|  | Foreign body in ear | 2020-2023 |  |  | +8.9% (+3.7 to +13.4) | 4 (1-10) | +7.1% (+1.5 to +11.2) | 10 (4-23) |
|  | Amputation of toe/toes | 2020-2023 |  |  | +8.9% (+4.4 to +13.2) | 4 (1-11) | +7.1% (+2.4 to +10.9) | 10 (4-24) |
|  | Foreign body in GI and urogenital system | 2020-2023 |  |  | +9.0% (+4.2 to +13.7) | 7 (2-18) | +7.1% (+2.1 to +10.7) | 18 (7-41) |
|  | Amputation of thumb | 2020-2023 |  |  | +8.8% (+3.5 to +13.5) | 5 (1-13) | +7.1% (+1.2 to +11.2) | 12 (4-29) |
|  | Fracture of femur, other than femoral neck | 2020-2023 |  |  | +8.9% (+3.6 to +13.3) | 25 (9-55) | +7.1% (+1.8 to +11.3) | 65 (28-129) |
|  | Amputation of upper limb, unilateral | 2020-2023 |  |  | +8.9% (+3.6 to +14.0) | 0 (0-1) | +7.1% (+1.6 to +12.3) | 1 (0-3) |
|  | Fracture of foot bones except ankle | 2020-2023 | +5.9% (+0.2 to +11.0) | 22 (9-45) | +8.9% (+4.3 to +13.4) | 12 (4-28) | +7.1% (+2.6 to +11.0) | 34 (13-73) |
|  | Minor TBI | 2020-2023 |  |  | +8.9% (+4.5 to +13.3) | 26 (11-50) | +7.0% (+1.6 to +11.1) | 73 (35-129) |
|  | Injury to eyes | 2020-2023 | +5.9% (+0.0 to +10.9) | 66 (34-116) | +8.9% (+4.6 to +13.3) | 34 (15-67) | +7.0% (+2.2 to +10.9) | 100 (50-183) |
|  | Nerve injury | 2020-2023 |  |  | +8.9% (+4.0 to +13.8) | 10 (4-22) | +7.0% (+2.1 to +11.0) | 28 (12-58) |
|  | Fracture of patella, tibia or fibula, or ankle | 2020-2023 |  |  | +8.9% (+4.3 to +12.6) | 40 (18-78) | +7.0% (+2.3 to +10.7) | 112 (57-193) |
|  | Multiple fractures, dislocations, crashes, wounds, pains, and strains | 2020-2023 |  |  | +8.9% (+4.5 to +13.1) | 24 (10-52) | +7.0% (+2.4 to +11.0) | 68 (32-132) |
|  | Amputation of fingers (excluding thumb) | 2020-2023 |  |  | +8.9% (+3.8 to +13.4) | 10 (4-24) | +7.0% (+1.9 to +11.4) | 30 (13-60) |
|  | Spinal cord lesion at neck level | 2020-2023 |  |  | +8.9% (+3.1 to +13.3) | 1 (0-2) | +7.0% (+1.9 to +11.0) | 2 (1-6) |
|  | Open wound(s) | 2020-2023 |  |  | +8.9% (+3.7 to +12.9) | 337 (179-580) | +7.0% (+1.7 to +11.0) | 1,005 (567-1,566) |
|  | Spinal cord lesion below neck level | 2020-2023 |  |  | +8.9% (+4.4 to +13.2) | 1 (0-2) | +7.0% (+1.9 to +10.8) | 2 (1-5) |
|  | Fracture of hand (wrist and other distal part of hand) | 2020-2023 |  |  | +8.9% (+3.7 to +13.5) | 26 (11-49) | +6.9% (+1.8 to +10.9) | 80 (40-139) |
|  | Crush injury | 2020-2023 | +5.9% (+0.1 to +10.9) | 4 (2-7) | +8.9% (+3.8 to +12.8) | 2 (1-3) | +6.9% (+2.1 to +11.0) | 5 (2-10) |
|  | Moderate/Severe TBI | 2020-2023 |  |  | +8.9% (+4.4 to +12.6) | 34 (17-60) | +6.9% (+1.5 to +10.7) | 114 (66-185) |
|  | Amputation of upper limbs, bilateral | 2020-2023 |  |  | +8.9% (+4.3 to +13.0) | 1 (0-2) | +6.9% (+1.6 to +11.3) | 2 (1-4) |
|  | Fracture of face bones | 2020-2023 |  |  | +8.9% (+3.9 to +13.3) | 30 (14-56) | +6.8% (+1.1 to +10.9) | 114 (57-193) |
|  | Fracture of sternum and/or fracture of one or more ribs | 2020-2023 |  |  | +8.9% (+4.5 to +12.6) | 3 (1-7) | +6.8% (+1.3 to +11.0) | 11 (4-24) |
|  | Internal hemorrhage in abdomen and pelvis | 2020-2023 |  |  | +8.9% (+4.2 to +12.9) | 47 (22-85) | +6.8% (+1.4 to +10.5) | 208 (113-343) |
|  | Severe chest Injury | 2020-2023 |  |  | +8.9% (+4.2 to +12.5) | 27 (11-50) | +6.7% (+1.1 to +10.6) | 127 (62-214) |
|  | Fracture of skull | 2020-2023 |  |  | +8.9% (+3.9 to +13.0) | 13 (5-29) | +6.7% (+1.2 to +11.1) | 59 (28-111) |
| <20 years | Fracture of hip | 2020-2023 | +11.3% (+7.5 to +14.2) | 15 (7-29) | +11.4% (+8.4 to +14.5) | 22 (10-49) | +11.3% (+8.4 to +13.9) | 37 (16-77) |
|  | Amputation of lower limb, unilateral | 2020-2023 | +11.0% (+7.4 to +13.9) | 3 (2-6) | +11.2% (+8.1 to +14.4) | 3 (2-6) | +11.1% (+8.2 to +13.5) | 7 (3-12) |
|  | Complications following therapeutic procedures | 2020-2023 | +10.4% (+6.7 to +13.4) | 146 (84-243) | +10.4% (+7.7 to +13.4) | 188 (99-343) | +10.4% (+7.7 to +12.4) | 334 (189-576) |
|  | Fracture of pelvis | 2020-2023 | +10.1% (+6.7 to +12.9) | 33 (13-80) | +10.2% (+7.2 to +13.5) | 46 (14-123) | +10.1% (+7.6 to +12.5) | 79 (27-201) |
|  | Fracture of sternum and/or fracture of one or more ribs | 2020-2023 | +10.0% (+6.3 to +12.9) | 49 (22-97) | +10.1% (+6.9 to +13.3) | 27 (10-64) | +10.1% (+7.1 to +12.4) | 77 (33-164) |
|  | Moderate/Severe TBI | 2020-2023 | +9.9% (+6.2 to +13.0) | 404 (275-541) | +10.0% (+7.2 to +13.2) | 250 (160-357) | +10.0% (+7.1 to +12.3) | 654 (433-887) |
|  | Minor TBI | 2020-2023 | +9.9% (+6.0 to +12.9) | 282 (163-452) | +10.0% (+6.6 to +13.1) | 239 (114-420) | +9.9% (+7.2 to +12.3) | 521 (275-869) |
|  | Multiple fractures, dislocations, crashes, wounds, pains, and strains | 2020-2023 | +9.9% (+6.2 to +13.0) | 264 (140-446) | +10.0% (+6.8 to +13.5) | 220 (101-390) | +9.9% (+7.0 to +12.4) | 484 (239-831) |
|  | Fracture of vertebral column | 2020-2023 | +9.9% (+6.3 to +13.0) | 105 (51-193) | +10.0% (+7.1 to +13.3) | 105 (43-219) | +9.9% (+7.2 to +12.3) | 209 (92-401) |
|  | Fracture of radius and/or ulna | 2020-2023 | +9.9% (+6.1 to +13.0) | 433 (235-692) | +9.9% (+7.0 to +13.0) | 486 (244-858) | +9.9% (+7.2 to +12.1) | 919 (486-1,524) |
|  | Spinal cord lesion at neck level | 2020-2023 | +9.8% (+6.0 to +12.6) | 8 (4-17) | +9.9% (+6.9 to +13.0) | 6 (3-15) | +9.9% (+6.9 to +12.2) | 15 (7-32) |
|  | Dislocation of shoulder | 2020-2023 | +9.8% (+6.2 to +12.9) | 55 (24-110) | +9.9% (+7.3 to +13.0) | 60 (22-131) | +9.9% (+7.4 to +12.1) | 115 (48-229) |
|  | Fracture of femur, other than femoral neck | 2020-2023 | +9.8% (+6.2 to +13.1) | 251 (122-470) | +9.9% (+7.0 to +12.9) | 242 (102-500) | +9.8% (+7.0 to +12.6) | 493 (229-966) |
|  | Superficial injury of any part of the body | 2020-2023 | +9.8% (+6.0 to +13.1) | 716 (438-1,075) | +9.9% (+6.9 to +13.1) | 849 (469-1,374) | +9.8% (+7.1 to +12.2) | 1,565 (892-2,415) |
|  | Fracture of patella, tibia or fibula, or ankle | 2020-2023 | +9.8% (+6.0 to +13.1) | 454 (271-683) | +9.9% (+7.0 to +13.2) | 382 (205-629) | +9.8% (+7.1 to +12.1) | 836 (473-1,317) |
|  | Fracture of clavicle, scapula, or humerus | 2020-2023 | +9.8% (+6.2 to +13.0) | 257 (136-458) | +9.9% (+6.8 to +13.1) | 279 (132-530) | +9.8% (+7.2 to +12.4) | 536 (272-992) |
|  | Open wound(s) | 2020-2023 | +9.7% (+6.1 to +13.0) | 4,299 (2,909-5,825) | +9.8% (+6.7 to +13.3) | 3,313 (2,152-4,581) | +9.8% (+7.1 to +12.3) | 7,612 (5,078-10,264) |
|  | Crush injury | 2020-2023 | +9.7% (+6.0 to +12.8) | 19 (10-33) | +9.8% (+7.1 to +13.0) | 12 (6-25) | +9.8% (+7.0 to +11.9) | 31 (16-57) |
|  | Foreign body in respiratory system | 2020-2023 | +9.8% (+5.9 to +12.8) | 59 (26-123) | +9.8% (+7.0 to +12.9) | 65 (24-156) | +9.8% (+6.9 to +12.2) | 124 (52-280) |
|  | Amputation of toe/toes | 2020-2023 | +9.7% (+6.1 to +12.8) | 40 (17-85) | +9.8% (+6.6 to +13.4) | 40 (12-95) | +9.8% (+7.0 to +12.2) | 81 (29-184) |
|  | Severe chest Injury | 2020-2023 | +9.7% (+5.9 to +12.9) | 646 (378-1,004) | +9.8% (+6.8 to +13.0) | 270 (141-493) | +9.8% (+6.6 to +12.6) | 916 (522-1,485) |
|  | Dislocation of hip | 2020-2023 | +9.7% (+5.8 to +13.2) | 41 (16-85) | +9.8% (+6.8 to +13.2) | 48 (15-117) | +9.8% (+6.9 to +12.3) | 89 (31-201) |
|  | Fracture of hand (wrist and other distal part of hand) | 2020-2023 | +9.7% (+5.8 to +13.0) | 353 (208-568) | +9.8% (+6.5 to +13.4) | 253 (138-436) | +9.7% (+6.9 to +12.2) | 605 (349-998) |
|  | Fracture of face bones | 2020-2023 | +9.6% (+5.6 to +12.9) | 554 (332-827) | +9.8% (+6.8 to +12.9) | 301 (169-515) | +9.7% (+6.9 to +12.2) | 855 (523-1,321) |
|  | Muscle and tendon injuries, including sprains and strains lesser dislocations | 2020-2023 | +9.7% (+6.2 to +12.8) | 884 (577-1,332) | +9.8% (+6.8 to +13.1) | 991 (592-1,574) | +9.7% (+7.1 to +12.2) | 1,876 (1,171-2,900) |
|  | Amputation of fingers (excluding thumb) | 2020-2023 | +9.6% (+6.0 to +13.3) | 129 (57-225) | +9.8% (+6.6 to +13.0) | 102 (39-226) | +9.7% (+7.2 to +12.3) | 230 (98-447) |
|  | Internal hemorrhage in abdomen and pelvis | 2020-2023 | +9.6% (+6.0 to +12.7) | 1,071 (656-1,550) | +9.7% (+6.7 to +13.1) | 478 (258-777) | +9.7% (+6.6 to +12.0) | 1,549 (919-2,318) |
|  | Contusion in any part of the body | 2020-2023 | +9.7% (+5.9 to +12.8) | 372 (228-554) | +9.8% (+7.0 to +12.8) | 480 (263-794) | +9.7% (+7.2 to +12.0) | 852 (500-1,373) |
|  | Poisoning requiring urgent care | 2020-2023 | +9.7% (+6.0 to +12.7) | 402 (229-619) | +9.8% (+6.7 to +13.1) | 685 (353-1,178) | +9.7% (+7.2 to +12.2) | 1,088 (597-1,837) |
|  | Foreign body in GI and urogenital system | 2020-2023 | +9.6% (+5.8 to +12.6) | 73 (32-141) | +9.7% (+6.8 to +13.3) | 72 (22-180) | +9.7% (+6.8 to +12.2) | 145 (55-321) |
|  | Injury to eyes | 2020-2023 | +9.6% (+5.6 to +12.9) | 442 (255-736) | +9.7% (+6.6 to +13.0) | 353 (170-654) | +9.7% (+6.9 to +12.0) | 795 (439-1,326) |
|  | Dislocation of knee | 2020-2023 | +9.6% (+5.8 to +12.8) | 44 (19-95) | +9.7% (+6.4 to +12.7) | 52 (15-140) | +9.6% (+7.0 to +11.8) | 96 (34-221) |
|  | Fracture of skull | 2020-2023 | +9.5% (+5.7 to +12.8) | 315 (173-522) | +9.6% (+6.8 to +13.0) | 137 (63-277) | +9.6% (+6.4 to +12.1) | 452 (237-795) |
|  | Amputation of thumb | 2020-2023 | +9.5% (+5.5 to +13.0) | 50 (21-108) | +9.6% (+6.1 to +13.3) | 51 (16-133) | +9.6% (+6.6 to +12.4) | 101 (38-240) |
|  | Fracture of foot bones except ankle | 2020-2023 | +9.5% (+5.7 to +12.7) | 150 (77-258) | +9.6% (+6.6 to +12.7) | 126 (54-268) | +9.6% (+6.8 to +11.8) | 276 (132-525) |
|  | Amputation of upper limb, unilateral | 2020-2023 | +9.5% (+5.6 to +13.1) | 5 (2-10) | +9.6% (+6.6 to +13.1) | 4 (1-10) | +9.5% (+6.5 to +12.2) | 9 (3-21) |
|  | Nerve injury | 2020-2023 | +9.5% (+5.6 to +12.7) | 122 (60-234) | +9.6% (+6.6 to +12.8) | 108 (45-244) | +9.5% (+7.0 to +12.2) | 230 (105-493) |
|  | Effect of different environmental factors | 2020-2023 | +9.4% (+5.6 to +12.7) | 71 (31-143) | +9.6% (+6.6 to +13.0) | 90 (32-213) | +9.5% (+6.7 to +12.2) | 161 (67-346) |
|  | Burns, >=20% total burned surface area or >= 10% burned surface area if head/neck or hands/wrist involved w/o lower airway burns | 2020-2023 | +9.5% (+5.6 to +12.6) | 46 (27-71) | +9.6% (+6.7 to +12.5) | 71 (39-117) | +9.5% (+6.8 to +11.9) | 118 (66-182) |
|  | Amputation of upper limbs, bilateral | 2020-2023 | +9.4% (+5.6 to +12.6) | 8 (3-15) | +9.5% (+6.3 to +12.8) | 5 (2-13) | +9.5% (+6.6 to +12.0) | 13 (5-29) |
|  | Asphyxiation | 2020-2023 | +9.4% (+5.9 to +12.6) | 36 (13-80) | +9.5% (+6.3 to +12.8) | 41 (12-102) | +9.4% (+6.7 to +11.9) | 76 (26-180) |
|  | Drowning and nonfatal submersion | 2020-2023 | +9.4% (+5.7 to +12.7) | 41 (16-81) | +9.5% (+6.6 to +13.0) | 48 (15-129) | +9.4% (+6.7 to +11.7) | 88 (31-208) |
|  | Amputation of lower limbs, bilateral | 2020-2023 | +9.3% (+5.7 to +12.6) | 2 (1-5) | +9.4% (+6.1 to +12.9) | 3 (1-7) | +9.4% (+6.6 to +12.1) | 5 (2-12) |
|  | Foreign body in ear | 2020-2023 | +9.3% (+5.7 to +12.7) | 44 (19-90) | +9.4% (+6.0 to +13.0) | 46 (15-112) | +9.4% (+6.7 to +11.9) | 91 (33-196) |
|  | Lower airway burns | 2020-2023 | +9.3% (+5.3 to +12.9) | 3 (1-7) | +9.3% (+6.4 to +12.9) | 4 (1-10) | +9.3% (+6.4 to +11.7) | 7 (2-17) |
|  | Burns, <20% total burned surface area without lower airway burns | 2020-2023 | +9.2% (+5.4 to +12.5) | 350 (182-635) | +9.4% (+6.0 to +12.8) | 457 (219-882) | +9.3% (+6.6 to +12.1) | 807 (409-1,513) |
|  | Spinal cord lesion below neck level | 2020-2023 | +9.2% (+5.3 to +12.1) | 10 (5-19) | +9.3% (+6.4 to +12.7) | 7 (3-17) | +9.3% (+6.6 to +11.6) | 17 (7-36) |

## Portugal

| **Age** | **Category** | **Period** | **Male %Change** | **Male Incidence 2023** | **Female %Change** | **Female Incidence 2023** | **Both %Change** | **Both Incidence 2023** |
| --- | --- | --- | --- | --- | --- | --- | --- | --- |
| 5-14 years | Poisoning requiring urgent care | 2020-2023 |  |  | +14.9% (+1.8 to +30.0) | 12 (5-22) | +13.2% (+3.8 to +22.6) | 19 (9-34) |
|  | Burns, <20% total burned surface area without lower airway burns | 2020-2023 |  |  | +15.3% (+0.2 to +31.1) | 16 (6-32) | +13.1% (+3.1 to +22.7) | 28 (12-54) |
|  | Burns, >=20% total burned surface area or >= 10% burned surface area if head/neck or hands/wrist involved w/o lower airway burns | 2020-2023 |  |  | +14.9% (+0.0 to +30.7) | 1 (0-1) | +13.0% (+2.7 to +22.7) | 1 (0-2) |
|  | Asphyxiation | 2020-2023 |  |  | +15.3% (+2.5 to +29.3) | 5 (1-15) | +13.0% (+4.6 to +21.9) | 10 (3-27) |
|  | Effect of different environmental factors | 2020-2023 |  |  |  |  | +12.9% (+2.7 to +23.4) | 10 (4-24) |
|  | Contusion in any part of the body | 2020-2023 |  |  | +15.0% (+2.7 to +30.8) | 106 (55-196) | +12.9% (+3.0 to +22.6) | 191 (102-333) |
|  | Drowning and nonfatal submersion | 2020-2023 |  |  |  |  | +12.9% (+3.8 to +21.6) | 9 (2-24) |
|  | Foreign body in ear | 2020-2023 |  |  | +15.2% (+1.1 to +33.0) | 4 (1-12) | +12.9% (+4.1 to +23.3) | 9 (3-22) |
|  | Dislocation of knee | 2020-2023 |  |  |  |  | +12.9% (+3.1 to +22.8) | 9 (3-22) |
|  | Dislocation of hip | 2020-2023 |  |  |  |  | +12.9% (+2.2 to +23.4) | 6 (2-14) |
|  | Muscle and tendon injuries, including sprains and strains lesser dislocations | 2020-2023 |  |  | +15.0% (+1.6 to +29.1) | 31 (15-58) | +12.8% (+4.1 to +21.9) | 60 (30-105) |
|  | Superficial injury of any part of the body | 2020-2023 |  |  | +14.9% (+1.0 to +30.8) | 126 (61-218) | +12.8% (+3.7 to +22.7) | 236 (114-393) |
|  | Amputation of thumb | 2020-2023 |  |  |  |  | +12.8% (+3.3 to +23.3) | 7 (2-19) |
|  | Foreign body in GI and urogenital system | 2020-2023 |  |  | +15.2% (+0.8 to +31.1) | 4 (1-9) | +12.8% (+1.8 to +22.8) | 7 (2-17) |
|  | Amputation of toe/toes | 2020-2023 |  |  |  |  | +12.8% (+3.6 to +22.8) | 5 (2-13) |
|  | Fracture of pelvis | 2020-2023 |  |  |  |  | +12.8% (+3.7 to +23.0) | 9 (3-23) |
|  | Amputation of lower limbs, bilateral | 2020-2023 |  |  | +14.8% (+0.2 to +32.0) | 0 (0-1) | +12.8% (+4.1 to +21.9) | 0 (0-1) |
|  | Lower airway burns | 2020-2023 |  |  | +15.0% (+2.5 to +31.5) | 0 (0-1) | +12.8% (+3.4 to +22.4) | 0 (0-1) |
|  | Foreign body in respiratory system | 2020-2023 |  |  | +15.0% (+1.8 to +29.4) | 3 (1-8) | +12.7% (+4.5 to +21.8) | 6 (2-13) |
|  | Dislocation of shoulder | 2020-2023 |  |  | +14.9% (+1.1 to +29.8) | 6 (2-16) | +12.7% (+3.1 to +21.7) | 12 (4-31) |
|  | Fracture of clavicle, scapula, or humerus | 2020-2023 |  |  | +15.0% (+0.1 to +29.4) | 17 (8-37) | +12.7% (+3.2 to +23.4) | 34 (14-67) |
|  | Complications following therapeutic procedures | 2020-2023 |  |  | +14.6% (+0.4 to +29.0) | 2 (1-3) | +12.7% (+2.9 to +21.9) | 3 (1-5) |
|  | Fracture of radius and/or ulna | 2020-2023 |  |  | +14.8% (+1.8 to +30.0) | 13 (6-26) | +12.7% (+3.9 to +22.4) | 25 (11-47) |
|  | Fracture of femur, other than femoral neck | 2020-2023 |  |  | +14.9% (+0.9 to +30.1) | 5 (2-11) | +12.6% (+3.6 to +22.1) | 10 (4-22) |
|  | Nerve injury | 2020-2023 |  |  | +15.1% (+1.7 to +30.5) | 8 (3-19) | +12.5% (+4.3 to +22.9) | 17 (7-38) |
|  | Fracture of vertebral column | 2020-2023 |  |  | +14.8% (+1.5 to +29.4) | 6 (2-12) | +12.5% (+3.5 to +22.2) | 11 (5-24) |
|  | Fracture of foot bones except ankle | 2020-2023 |  |  | +15.0% (+1.2 to +30.9) | 6 (2-14) | +12.4% (+4.0 to +22.9) | 14 (6-30) |
|  | Injury to eyes | 2020-2023 |  |  | +15.1% (+3.2 to +30.5) | 28 (12-56) | +12.4% (+4.2 to +21.3) | 65 (31-123) |
|  | Amputation of fingers (excluding thumb) | 2020-2023 |  |  | +15.0% (+1.3 to +30.7) | 3 (1-7) | +12.4% (+3.6 to +22.5) | 7 (2-15) |
|  | Fracture of patella, tibia or fibula, or ankle | 2020-2023 |  |  | +15.0% (+2.6 to +30.1) | 13 (6-26) | +12.4% (+3.3 to +21.1) | 30 (14-53) |
|  | Minor TBI | 2020-2023 |  |  | +14.9% (+0.5 to +29.4) | 22 (11-42) | +12.4% (+4.0 to +22.2) | 50 (24-92) |
|  | Amputation of upper limb, unilateral | 2020-2023 |  |  |  |  | +12.4% (+2.4 to +22.0) | 0 (0-1) |
|  | Spinal cord lesion below neck level | 2020-2023 |  |  | +15.0% (+2.7 to +31.6) | 0 (0-1) | +12.3% (+3.9 to +21.9) | 1 (0-3) |
|  | Open wound(s) | 2020-2023 | +10.5% (+0.2 to +24.0) | 196 (112-316) | +14.9% (+1.9 to +29.5) | 145 (79-245) | +12.3% (+3.7 to +21.3) | 340 (192-544) |
|  | Fracture of hip | 2020-2023 |  |  | +14.0% (+0.8 to +29.1) | 1 (0-2) | +12.3% (+3.2 to +22.4) | 2 (1-4) |
|  | Multiple fractures, dislocations, crashes, wounds, pains, and strains | 2020-2023 |  |  | +14.8% (+1.4 to +31.9) | 5 (2-10) | +12.3% (+5.0 to +21.3) | 11 (5-21) |
|  | Fracture of hand (wrist and other distal part of hand) | 2020-2023 |  |  | +15.0% (+1.5 to +29.2) | 29 (12-58) | +12.2% (+3.5 to +22.0) | 71 (34-132) |
|  | Spinal cord lesion at neck level | 2020-2023 |  |  | +14.6% (+1.9 to +29.3) | 0 (0-1) | +12.1% (+4.0 to +20.3) | 1 (0-2) |
|  | Amputation of upper limbs, bilateral | 2020-2023 |  |  | +14.6% (+0.1 to +31.8) | 0 (0-0) | +12.0% (+2.9 to +21.4) | 0 (0-1) |
|  | Amputation of lower limb, unilateral | 2020-2023 |  |  | +14.0% (+1.1 to +29.0) | 0 (0-0) | +12.0% (+2.8 to +21.5) | 0 (0-0) |
|  | Fracture of face bones | 2020-2023 |  |  | +15.1% (+1.0 to +29.2) | 29 (13-54) | +12.0% (+3.1 to +23.4) | 85 (40-143) |
|  | Fracture of sternum and/or fracture of one or more ribs | 2020-2023 |  |  |  |  | +12.0% (+2.6 to +22.1) | 18 (7-39) |
|  | Crush injury | 2020-2023 |  |  | +14.4% (+0.3 to +30.3) | 0 (0-0) | +12.0% (+4.3 to +22.8) | 0 (0-1) |
|  | Moderate/Severe TBI | 2020-2023 |  |  | +14.6% (+1.9 to +28.4) | 5 (3-8) | +11.8% (+4.0 to +21.5) | 12 (7-20) |
|  | Internal hemorrhage in abdomen and pelvis | 2020-2023 |  |  | +15.1% (+1.7 to +30.1) | 14 (7-24) | +11.8% (+2.2 to +22.6) | 45 (24-73) |
|  | Severe chest Injury | 2020-2023 |  |  | +15.0% (+0.9 to +29.6) | 10 (5-19) | +11.8% (+2.5 to +22.9) | 34 (17-61) |
|  | Fracture of skull | 2020-2023 |  |  | +15.1% (+2.7 to +31.2) | 18 (7-35) | +11.7% (+2.5 to +22.8) | 59 (27-106) |
| <20 years | Fracture of hip | 2020-2023 |  |  |  |  | +7.7% (+1.2 to +15.1) | 9 (4-18) |
|  | Fracture of pelvis | 2020-2023 |  |  |  |  | +7.4% (+0.4 to +15.4) | 49 (16-118) |
|  | Complications following therapeutic procedures | 2020-2023 |  |  |  |  | +7.4% (+0.8 to +15.0) | 16 (9-26) |
|  | Burns, >=20% total burned surface area or >= 10% burned surface area if head/neck or hands/wrist involved w/o lower airway burns | 2020-2023 |  |  |  |  | +7.3% (+0.3 to +15.5) | 6 (3-11) |
|  | Contusion in any part of the body | 2020-2023 |  |  |  |  | +7.3% (+0.6 to +14.9) | 1,150 (727-1,683) |
|  | Amputation of lower limb, unilateral | 2020-2023 |  |  |  |  | +7.3% (+1.0 to +14.1) | 0 (0-0) |
|  | Superficial injury of any part of the body | 2020-2023 |  |  |  |  | +7.2% (+1.0 to +15.0) | 1,384 (880-2,064) |
|  | Dislocation of hip | 2020-2023 |  |  |  |  | +7.2% (+0.5 to +14.2) | 35 (10-85) |
|  | Drowning and nonfatal submersion | 2020-2023 |  |  |  |  | +7.2% (+0.6 to +14.1) | 56 (18-143) |
|  | Asphyxiation | 2020-2023 |  |  |  |  | +7.2% (+0.3 to +14.8) | 60 (19-147) |
|  | Dislocation of knee | 2020-2023 |  |  |  |  | +7.2% (+0.5 to +14.6) | 53 (17-126) |
|  | Fracture of radius and/or ulna | 2020-2023 |  |  |  |  | +7.2% (+0.7 to +14.6) | 147 (82-245) |
|  | Foreign body in respiratory system | 2020-2023 |  |  |  |  | +7.1% (+0.1 to +14.5) | 33 (12-80) |
|  | Muscle and tendon injuries, including sprains and strains lesser dislocations | 2020-2023 |  |  |  |  | +7.1% (+0.5 to +14.7) | 357 (222-572) |
|  | Fracture of clavicle, scapula, or humerus | 2020-2023 |  |  |  |  | +7.1% (+0.0 to +14.7) | 199 (102-358) |
|  | Dislocation of shoulder | 2020-2023 |  |  |  |  | +7.1% (+0.5 to +14.7) | 70 (27-162) |
|  | Amputation of lower limbs, bilateral | 2020-2023 |  |  |  |  | +7.0% (+0.1 to +14.6) | 3 (1-6) |
|  | Foreign body in GI and urogenital system | 2020-2023 |  |  |  |  | +7.0% (+0.6 to +14.5) | 45 (17-105) |
|  | Fracture of femur, other than femoral neck | 2020-2023 |  |  |  |  | +7.0% (+0.6 to +13.5) | 59 (28-115) |
|  | Fracture of vertebral column | 2020-2023 |  |  |  |  | +7.0% (+0.4 to +15.6) | 66 (30-136) |
|  | Foreign body in ear | 2020-2023 |  |  |  |  | +6.9% (+0.2 to +14.0) | 56 (18-130) |
|  | Minor TBI | 2020-2023 |  |  |  |  | +6.9% (+0.7 to +14.4) | 286 (166-465) |
|  | Multiple fractures, dislocations, crashes, wounds, pains, and strains | 2020-2023 |  |  |  |  | +6.8% (+0.4 to +14.1) | 62 (34-107) |
|  | Fracture of patella, tibia or fibula, or ankle | 2020-2023 |  |  |  |  | +6.8% (+0.5 to +13.5) | 173 (100-276) |
|  | Nerve injury | 2020-2023 |  |  |  |  | +6.8% (+0.1 to +14.1) | 106 (49-220) |
|  | Open wound(s) | 2020-2023 |  |  |  |  | +6.8% (+0.7 to +13.7) | 1,979 (1,398-2,680) |
|  | Injury to eyes | 2020-2023 |  |  |  |  | +6.7% (+0.3 to +14.6) | 387 (208-618) |
|  | Fracture of foot bones except ankle | 2020-2023 |  |  |  |  | +6.7% (+0.9 to +14.5) | 83 (38-165) |
|  | Fracture of hand (wrist and other distal part of hand) | 2020-2023 |  |  |  |  | +6.6% (+0.2 to +13.5) | 416 (246-661) |
|  | Spinal cord lesion below neck level | 2020-2023 |  |  |  |  | +6.5% (+0.1 to +13.2) | 7 (3-17) |
|  | Crush injury | 2020-2023 |  |  |  |  | +6.5% (+0.2 to +13.5) | 2 (1-4) |
|  | Moderate/Severe TBI | 2020-2023 |  |  |  |  | +6.5% (+0.4 to +13.6) | 71 (47-98) |
|  | Fracture of sternum and/or fracture of one or more ribs | 2020-2023 |  |  |  |  | +6.5% (+0.8 to +14.2) | 98 (46-194) |

## Romania

| **Age** | **Category** | **Period** | **Male %Change** | **Male Incidence 2023** | **Female %Change** | **Female Incidence 2023** | **Both %Change** | **Both Incidence 2023** |
| --- | --- | --- | --- | --- | --- | --- | --- | --- |
| <5 years | Poisoning requiring urgent care | 1990-2023 | +63.4% (+41.4 to +87.5) | 402 (240-619) | +86.0% (+62.4 to +108.2) | 913 (505-1,532) | +77.9% (+55.5 to +96.0) | 1,315 (766-2,149) |
|  | Burns, >=20% total burned surface area or >= 10% burned surface area if head/neck or hands/wrist involved w/o lower airway burns | 1990-2023 | +63.6% (+41.1 to +88.9) | 48 (29-71) | +86.4% (+61.4 to +111.0) | 99 (58-155) | +77.8% (+54.5 to +96.2) | 148 (88-227) |
|  | Fracture of hip | 1990-2023 | +63.4% (+42.1 to +92.2) | 11 (5-21) | +85.4% (+62.5 to +113.2) | 22 (10-48) | +77.2% (+53.7 to +98.5) | 32 (15-68) |
|  | Fracture of pelvis | 1990-2023 | +63.5% (+41.2 to +92.2) | 31 (13-74) | +85.7% (+62.0 to +112.0) | 57 (18-141) | +76.8% (+54.1 to +97.1) | 88 (32-217) |
|  | Burns, <20% total burned surface area without lower airway burns | 1990-2023 | +63.6% (+39.4 to +93.3) | 380 (202-643) | +86.0% (+58.7 to +113.5) | 651 (290-1,178) | +76.8% (+51.6 to +97.4) | 1,031 (513-1,802) |
|  | Effect of different environmental factors | 1990-2023 | +63.5% (+41.7 to +91.4) | 74 (31-151) | +86.1% (+63.6 to +111.4) | 124 (49-301) | +76.7% (+55.0 to +95.2) | 198 (78-442) |
|  | Contusion in any part of the body | 1990-2023 | +63.4% (+42.5 to +89.2) | 373 (234-568) | +86.0% (+62.7 to +111.0) | 642 (357-1,082) | +76.6% (+56.3 to +96.5) | 1,014 (609-1,645) |
|  | Complications following therapeutic procedures | 1990-2023 | +63.5% (+41.6 to +89.1) | 128 (75-202) | +85.6% (+61.1 to +110.4) | 225 (121-397) | +76.6% (+54.1 to +95.5) | 353 (201-598) |
|  | Dislocation of knee | 1990-2023 | +63.7% (+39.4 to +90.3) | 45 (18-88) | +86.3% (+60.4 to +112.7) | 71 (21-171) | +76.6% (+54.2 to +94.5) | 116 (38-258) |
|  | Amputation of lower limbs, bilateral | 1990-2023 | +63.7% (+40.4 to +91.6) | 3 (1-5) | +86.5% (+60.0 to +113.4) | 4 (1-10) | +76.5% (+53.6 to +94.6) | 6 (2-16) |
|  | Lower airway burns | 1990-2023 | +63.7% (+41.0 to +91.6) | 4 (1-8) | +86.5% (+58.4 to +113.0) | 5 (2-14) | +76.4% (+55.3 to +96.4) | 9 (3-21) |
|  | Superficial injury of any part of the body | 1990-2023 | +63.5% (+41.2 to +90.9) | 701 (461-993) | +85.9% (+60.5 to +111.3) | 1,110 (665-1,696) | +76.2% (+55.0 to +95.1) | 1,811 (1,137-2,662) |
|  | Asphyxiation | 1990-2023 | +63.5% (+38.6 to +92.8) | 37 (15-81) | +86.2% (+60.5 to +111.3) | 57 (18-148) | +76.2% (+51.6 to +97.9) | 94 (33-227) |
|  | Muscle and tendon injuries, including sprains and strains lesser dislocations | 1990-2023 | +63.6% (+38.7 to +89.5) | 887 (577-1,277) | +86.0% (+61.5 to +110.9) | 1,323 (805-2,079) | +76.1% (+53.3 to +95.6) | 2,210 (1,389-3,337) |
|  | Amputation of lower limb, unilateral | 1990-2023 | +63.5% (+40.3 to +91.7) | 2 (1-4) | +86.0% (+59.9 to +112.7) | 4 (2-6) | +76.0% (+51.9 to +94.0) | 6 (3-10) |
|  | Drowning and nonfatal submersion | 1990-2023 | +63.2% (+42.5 to +89.1) | 43 (18-87) | +85.7% (+61.9 to +113.8) | 67 (21-175) | +76.0% (+56.1 to +94.6) | 110 (40-256) |
|  | Dislocation of hip | 1990-2023 | +63.3% (+42.5 to +87.8) | 41 (17-83) | +85.5% (+61.7 to +112.3) | 64 (20-151) | +75.9% (+54.8 to +93.3) | 105 (38-230) |
|  | Fracture of radius and/or ulna | 1990-2023 | +63.3% (+40.0 to +90.1) | 418 (257-634) | +85.7% (+61.8 to +110.2) | 631 (339-1,019) | +75.8% (+53.3 to +95.8) | 1,049 (601-1,649) |
|  | Foreign body in respiratory system | 1990-2023 | +63.5% (+40.2 to +93.3) | 58 (27-117) | +85.7% (+64.3 to +112.8) | 86 (31-206) | +75.8% (+54.9 to +94.4) | 144 (60-322) |
|  | Dislocation of shoulder | 1990-2023 | +63.6% (+40.9 to +90.9) | 54 (25-102) | +85.8% (+60.7 to +109.6) | 78 (29-183) | +75.8% (+53.0 to +93.8) | 132 (56-283) |
|  | Fracture of clavicle, scapula, or humerus | 1990-2023 | +63.3% (+41.2 to +87.5) | 251 (137-422) | +85.8% (+62.2 to +110.7) | 365 (180-674) | +75.7% (+54.5 to +94.1) | 616 (319-1,103) |
|  | Foreign body in ear | 1990-2023 | +63.4% (+41.0 to +94.1) | 47 (20-96) | +85.9% (+59.3 to +113.8) | 65 (22-150) | +75.6% (+52.5 to +95.0) | 112 (42-238) |
|  | Amputation of thumb | 1990-2023 | +63.5% (+40.8 to +92.8) | 52 (22-110) | +85.9% (+59.3 to +111.0) | 69 (23-176) | +75.5% (+54.2 to +94.6) | 121 (44-287) |
|  | Amputation of toe/toes | 1990-2023 | +63.5% (+42.6 to +90.1) | 40 (18-82) | +85.9% (+59.6 to +112.1) | 54 (19-136) | +75.5% (+56.3 to +94.5) | 94 (37-212) |
|  | Foreign body in GI and urogenital system | 1990-2023 | +63.5% (+40.4 to +91.7) | 74 (34-146) | +86.1% (+61.7 to +109.7) | 97 (33-237) | +75.5% (+53.8 to +95.4) | 172 (67-385) |
|  | Fracture of femur, other than femoral neck | 1990-2023 | +63.5% (+41.4 to +91.6) | 246 (128-434) | +86.1% (+61.5 to +111.3) | 317 (142-630) | +75.4% (+53.0 to +94.3) | 563 (275-1,062) |
|  | Fracture of vertebral column | 1990-2023 | +63.2% (+41.9 to +90.5) | 101 (47-194) | +85.8% (+61.5 to +111.1) | 136 (52-279) | +75.3% (+53.4 to +93.0) | 236 (105-467) |
|  | Amputation of upper limb, unilateral | 1990-2023 | +63.6% (+38.9 to +93.1) | 5 (2-10) | +86.7% (+62.6 to +115.0) | 6 (2-15) | +75.2% (+52.0 to +96.2) | 11 (4-25) |
|  | Nerve injury | 1990-2023 | +63.4% (+42.8 to +89.6) | 127 (64-229) | +86.3% (+62.0 to +113.6) | 150 (63-327) | +75.0% (+53.1 to +92.7) | 276 (130-567) |
|  | Minor TBI | 1990-2023 | +63.5% (+42.5 to +89.6) | 271 (164-432) | +85.8% (+60.8 to +113.3) | 308 (162-518) | +74.6% (+52.9 to +93.2) | 579 (327-937) |
|  | Fracture of patella, tibia or fibula, or ankle | 1990-2023 | +63.4% (+41.4 to +88.2) | 446 (270-672) | +85.9% (+61.3 to +110.1) | 502 (274-846) | +74.6% (+54.6 to +93.8) | 949 (558-1,495) |
|  | Spinal cord lesion at neck level | 1990-2023 | +63.4% (+40.0 to +89.9) | 8 (4-14) | +86.3% (+61.8 to +112.5) | 9 (4-19) | +74.6% (+49.8 to +94.9) | 16 (8-34) |
|  | Multiple fractures, dislocations, crashes, wounds, pains, and strains | 1990-2023 | +63.5% (+41.7 to +90.4) | 254 (148-420) | +85.7% (+60.0 to +112.5) | 284 (142-513) | +74.5% (+52.0 to +93.7) | 538 (290-912) |
|  | Spinal cord lesion below neck level | 1990-2023 | +63.7% (+41.5 to +88.4) | 10 (5-20) | +86.5% (+62.7 to +109.5) | 10 (3-24) | +74.5% (+54.6 to +91.9) | 21 (9-43) |
|  | Amputation of fingers (excluding thumb) | 1990-2023 | +63.6% (+41.1 to +90.2) | 129 (63-231) | +86.1% (+58.3 to +110.6) | 136 (55-289) | +74.4% (+53.3 to +92.5) | 265 (122-525) |
|  | Fracture of foot bones except ankle | 1990-2023 | +63.2% (+41.2 to +89.9) | 154 (78-272) | +85.8% (+59.8 to +113.7) | 173 (72-371) | +74.4% (+51.2 to +93.5) | 327 (157-624) |
|  | Injury to eyes | 1990-2023 | +63.5% (+40.2 to +91.2) | 448 (270-677) | +85.8% (+62.8 to +110.8) | 475 (240-799) | +74.3% (+51.3 to +92.8) | 923 (546-1,498) |
|  | Amputation of upper limbs, bilateral | 1990-2023 | +63.6% (+41.9 to +89.2) | 8 (3-16) | +86.6% (+64.8 to +111.0) | 7 (2-18) | +74.1% (+54.6 to +94.0) | 15 (6-32) |
|  | Open wound(s) | 1990-2023 | +63.5% (+41.7 to +92.1) | 4,258 (3,240-5,490) | +85.7% (+61.9 to +111.4) | 4,376 (3,065-5,922) | +74.1% (+52.2 to +93.8) | 8,634 (6,435-11,308) |
|  | Crush injury | 1990-2023 | +63.6% (+40.7 to +91.5) | 18 (10-32) | +86.6% (+62.4 to +110.5) | 17 (8-32) | +73.9% (+53.1 to +93.3) | 35 (18-63) |
|  | Fracture of hand (wrist and other distal part of hand) | 1990-2023 | +63.4% (+42.0 to +90.7) | 353 (218-530) | +86.1% (+61.3 to +111.1) | 337 (182-561) | +73.9% (+54.0 to +93.5) | 691 (403-1,072) |
|  | Moderate/Severe TBI | 1990-2023 | +63.5% (+41.8 to +93.8) | 385 (288-504) | +86.5% (+61.1 to +109.9) | 325 (233-445) | +73.5% (+50.9 to +92.8) | 710 (526-944) |
|  | Fracture of sternum and/or fracture of one or more ribs | 1990-2023 | +63.3% (+40.3 to +89.4) | 47 (22-88) | +86.0% (+60.3 to +112.3) | 35 (14-83) | +72.5% (+51.9 to +92.3) | 81 (34-169) |
|  | Fracture of face bones | 1990-2023 | +63.4% (+42.6 to +89.9) | 558 (361-858) | +85.9% (+57.7 to +112.0) | 403 (236-684) | +72.4% (+52.5 to +91.9) | 962 (599-1,547) |
|  | Fracture of skull | 1990-2023 | +63.5% (+41.3 to +90.0) | 324 (188-518) | +86.0% (+63.3 to +110.5) | 186 (91-372) | +71.5% (+51.3 to +90.5) | 510 (285-903) |
|  | Internal hemorrhage in abdomen and pelvis | 1990-2023 | +63.3% (+41.3 to +91.8) | 1,084 (733-1,499) | +86.0% (+60.7 to +110.7) | 645 (386-1,026) | +71.5% (+51.7 to +94.9) | 1,729 (1,112-2,502) |
|  | Severe chest Injury | 1990-2023 | +63.4% (+42.1 to +91.6) | 645 (396-963) | +85.9% (+61.5 to +112.1) | 360 (196-636) | +71.2% (+49.8 to +91.4) | 1,005 (600-1,535) |
|  | Poisoning requiring urgent care | 2010-2023 |  |  | +23.0% (+9.3 to +37.2) | 913 (505-1,532) | +12.7% (+3.0 to +22.5) | 1,315 (766-2,149) |
|  | Fracture of hip | 2010-2023 |  |  | +23.2% (+8.5 to +39.2) | 22 (10-48) | +12.4% (+3.5 to +23.5) | 32 (15-68) |
|  | Burns, >=20% total burned surface area or >= 10% burned surface area if head/neck or hands/wrist involved w/o lower airway burns | 2010-2023 |  |  | +22.6% (+7.9 to +36.5) | 99 (58-155) | +11.6% (+2.3 to +21.4) | 148 (88-227) |
|  | Fracture of pelvis | 2010-2023 |  |  | +23.0% (+8.9 to +38.9) | 57 (18-141) | +11.4% (+1.7 to +23.0) | 88 (32-217) |
|  | Complications following therapeutic procedures | 2010-2023 |  |  | +23.2% (+10.0 to +37.8) | 225 (121-397) | +11.1% (+2.6 to +22.5) | 353 (201-598) |
|  | Contusion in any part of the body | 2010-2023 |  |  | +23.0% (+9.6 to +38.0) | 642 (357-1,082) | +10.8% (+1.8 to +21.8) | 1,014 (609-1,645) |
|  | Burns, <20% total burned surface area without lower airway burns | 2010-2023 |  |  | +23.0% (+6.9 to +40.3) | 651 (290-1,178) | +10.8% (+1.4 to +21.8) | 1,031 (513-1,802) |
|  | Effect of different environmental factors | 2010-2023 |  |  | +23.0% (+8.3 to +37.8) | 124 (49-301) | +10.7% (+0.8 to +20.8) | 198 (78-442) |
|  | Dislocation of knee | 2010-2023 |  |  | +23.2% (+8.4 to +39.1) | 71 (21-171) |  |  |
|  | Superficial injury of any part of the body | 2010-2023 |  |  | +23.1% (+8.8 to +38.0) | 1,110 (665-1,696) | +10.4% (+1.5 to +21.4) | 1,811 (1,137-2,662) |
|  | Dislocation of hip | 2010-2023 |  |  | +22.9% (+8.5 to +39.7) | 64 (20-151) |  |  |
|  | Drowning and nonfatal submersion | 2010-2023 |  |  | +22.9% (+9.9 to +39.3) | 67 (21-175) | +10.0% (+0.9 to +20.8) | 110 (40-256) |
|  | Asphyxiation | 2010-2023 |  |  | +23.0% (+8.8 to +39.3) | 57 (18-148) |  |  |
|  | Muscle and tendon injuries, including sprains and strains lesser dislocations | 2010-2023 |  |  | +23.0% (+8.9 to +38.3) | 1,323 (805-2,079) | +9.9% (+1.1 to +21.8) | 2,210 (1,389-3,337) |
|  | Fracture of radius and/or ulna | 2010-2023 |  |  | +22.9% (+10.2 to +37.2) | 631 (339-1,019) | +9.8% (+1.3 to +20.7) | 1,049 (601-1,649) |
|  | Foreign body in respiratory system | 2010-2023 |  |  | +22.9% (+8.8 to +39.6) | 86 (31-206) | +9.8% (+0.3 to +19.3) | 144 (60-322) |
|  | Dislocation of shoulder | 2010-2023 |  |  | +23.0% (+8.9 to +37.3) | 78 (29-183) | +9.7% (+0.2 to +19.8) | 132 (56-283) |
|  | Fracture of clavicle, scapula, or humerus | 2010-2023 |  |  | +23.0% (+8.7 to +37.2) | 365 (180-674) | +9.7% (+1.3 to +20.0) | 616 (319-1,103) |
|  | Amputation of lower limb, unilateral | 2010-2023 |  |  | +22.8% (+9.0 to +37.6) | 4 (2-6) | +9.6% (+0.6 to +19.8) | 6 (3-10) |
|  | Amputation of lower limbs, bilateral | 2010-2023 |  |  | +22.7% (+7.3 to +40.8) | 4 (1-10) |  |  |
|  | Lower airway burns | 2010-2023 |  |  | +22.5% (+7.0 to +39.8) | 5 (2-14) |  |  |
|  | Foreign body in ear | 2010-2023 |  |  | +23.0% (+9.0 to +39.2) | 65 (22-150) |  |  |
|  | Amputation of thumb | 2010-2023 |  |  | +23.1% (+6.8 to +39.4) | 69 (23-176) |  |  |
|  | Fracture of vertebral column | 2010-2023 |  |  | +23.0% (+8.6 to +39.3) | 136 (52-279) | +9.1% (+0.3 to +18.6) | 236 (105-467) |
|  | Amputation of toe/toes | 2010-2023 |  |  | +22.8% (+8.7 to +38.3) | 54 (19-136) |  |  |
|  | Foreign body in GI and urogenital system | 2010-2023 |  |  | +22.9% (+6.3 to +38.8) | 97 (33-237) |  |  |
|  | Fracture of femur, other than femoral neck | 2010-2023 |  |  | +23.0% (+9.0 to +38.3) | 317 (142-630) |  |  |
|  | Nerve injury | 2010-2023 |  |  | +23.1% (+8.9 to +39.0) | 150 (63-327) |  |  |
|  | Minor TBI | 2010-2023 |  |  | +23.1% (+8.7 to +38.6) | 308 (162-518) |  |  |
|  | Fracture of patella, tibia or fibula, or ankle | 2010-2023 |  |  | +23.1% (+9.2 to +38.3) | 502 (274-846) |  |  |
|  | Multiple fractures, dislocations, crashes, wounds, pains, and strains | 2010-2023 |  |  | +23.0% (+8.1 to +38.5) | 284 (142-513) |  |  |
|  | Fracture of foot bones except ankle | 2010-2023 |  |  | +22.8% (+9.0 to +38.3) | 173 (72-371) |  |  |
|  | Amputation of upper limb, unilateral | 2010-2023 |  |  | +22.8% (+8.1 to +40.0) | 6 (2-15) |  |  |
|  | Injury to eyes | 2010-2023 |  |  | +22.9% (+9.7 to +38.4) | 475 (240-799) |  |  |
|  | Amputation of fingers (excluding thumb) | 2010-2023 |  |  | +23.1% (+8.6 to +38.4) | 136 (55-289) |  |  |
|  | Open wound(s) | 2010-2023 |  |  | +23.0% (+9.0 to +38.4) | 4,376 (3,065-5,922) |  |  |
|  | Spinal cord lesion at neck level | 2010-2023 |  |  | +22.9% (+7.5 to +37.2) | 9 (4-19) |  |  |
|  | Fracture of hand (wrist and other distal part of hand) | 2010-2023 |  |  | +23.1% (+8.3 to +38.2) | 337 (182-561) |  |  |
|  | Spinal cord lesion below neck level | 2010-2023 |  |  | +22.7% (+7.2 to +38.2) | 10 (3-24) |  |  |
|  | Amputation of upper limbs, bilateral | 2010-2023 |  |  | +22.8% (+8.4 to +37.5) | 7 (2-18) |  |  |
|  | Crush injury | 2010-2023 |  |  | +22.7% (+9.5 to +37.8) | 17 (8-32) |  |  |
|  | Moderate/Severe TBI | 2010-2023 |  |  | +22.8% (+9.1 to +38.1) | 325 (233-445) |  |  |
|  | Fracture of sternum and/or fracture of one or more ribs | 2010-2023 |  |  | +23.1% (+9.2 to +38.3) | 35 (14-83) |  |  |
|  | Fracture of face bones | 2010-2023 |  |  | +22.9% (+9.2 to +38.4) | 403 (236-684) |  |  |
|  | Internal hemorrhage in abdomen and pelvis | 2010-2023 |  |  | +23.0% (+9.6 to +37.8) | 645 (386-1,026) |  |  |
|  | Fracture of skull | 2010-2023 |  |  | +23.1% (+10.0 to +37.9) | 186 (91-372) |  |  |
|  | Severe chest Injury | 2010-2023 |  |  | +23.0% (+8.7 to +38.4) | 360 (196-636) |  |  |
| 5-14 years | Drowning and nonfatal submersion | 2020-2023 | +12.6% (+1.5 to +26.4) | 8 (3-17) |  |  | +12.8% (+4.1 to +23.7) | 16 (5-42) |
|  | Burns, <20% total burned surface area without lower airway burns | 2020-2023 | +12.8% (+0.8 to +26.2) | 67 (29-126) |  |  | +12.8% (+1.9 to +23.2) | 148 (66-294) |
|  | Foreign body in GI and urogenital system | 2020-2023 | +12.7% (+0.9 to +26.5) | 14 (5-29) |  |  | +12.7% (+1.9 to +24.2) | 28 (9-61) |
|  | Asphyxiation | 2020-2023 | +12.6% (+0.7 to +26.8) | 7 (2-15) |  |  | +12.7% (+1.7 to +23.5) | 14 (4-32) |
|  | Foreign body in ear | 2020-2023 | +12.9% (+0.2 to +25.3) | 8 (3-18) |  |  | +12.7% (+3.0 to +22.7) | 17 (6-40) |
|  | Lower airway burns | 2020-2023 | +12.5% (+0.4 to +27.1) | 1 (0-1) |  |  | +12.7% (+1.2 to +23.0) | 1 (0-3) |
|  | Injury to eyes | 2020-2023 | +12.6% (+1.2 to +25.5) | 87 (46-143) |  |  | +12.7% (+3.3 to +22.8) | 152 (78-268) |
|  | Effect of different environmental factors | 2020-2023 | +12.5% (+0.3 to +27.1) | 14 (5-29) |  |  | +12.6% (+2.3 to +23.9) | 30 (11-67) |
|  | Fracture of femur, other than femoral neck | 2020-2023 | +12.6% (+1.9 to +26.2) | 50 (23-98) |  |  | +12.6% (+2.8 to +22.8) | 95 (42-207) |
|  | Contusion in any part of the body | 2020-2023 | +12.6% (+1.2 to +25.2) | 73 (39-125) |  |  | +12.6% (+2.6 to +23.2) | 162 (84-277) |
|  | Nerve injury | 2020-2023 | +12.7% (+0.6 to +25.8) | 24 (10-48) |  |  | +12.6% (+3.4 to +23.5) | 43 (17-94) |
|  | Severe chest Injury | 2020-2023 | +12.6% (+1.8 to +26.4) | 127 (69-217) |  |  | +12.6% (+3.4 to +23.3) | 177 (93-321) |
|  | Internal hemorrhage in abdomen and pelvis | 2020-2023 | +12.6% (+1.6 to +24.9) | 209 (111-331) |  |  | +12.6% (+3.5 to +22.4) | 297 (153-483) |
|  | Muscle and tendon injuries, including sprains and strains lesser dislocations | 2020-2023 | +12.6% (+1.8 to +24.2) | 173 (97-286) |  |  | +12.6% (+2.4 to +22.8) | 358 (198-598) |
|  | Amputation of upper limb, unilateral | 2020-2023 |  |  |  |  | +12.6% (+2.4 to +23.2) | 2 (1-4) |
|  | Fracture of skull | 2020-2023 | +12.6% (+1.0 to +26.6) | 61 (30-108) | +12.6% (+0.6 to +24.9) | 25 (10-52) | +12.6% (+4.1 to +23.6) | 86 (41-157) |
|  | Amputation of thumb | 2020-2023 | +12.7% (+0.6 to +26.4) | 10 (4-21) |  |  | +12.6% (+2.2 to +22.7) | 19 (7-46) |
|  | Fracture of hand (wrist and other distal part of hand) | 2020-2023 | +12.5% (+1.0 to +26.5) | 69 (34-121) |  |  | +12.6% (+2.8 to +23.4) | 116 (57-204) |
|  | Fracture of patella, tibia or fibula, or ankle | 2020-2023 | +12.6% (+1.1 to +26.2) | 89 (44-155) |  |  | +12.5% (+2.7 to +22.5) | 161 (78-272) |
|  | Poisoning requiring urgent care | 2020-2023 | +12.5% (+1.8 to +26.5) | 79 (38-131) |  |  | +12.5% (+2.4 to +22.6) | 206 (101-362) |
|  | Dislocation of shoulder | 2020-2023 |  |  |  |  | +12.5% (+2.3 to +22.6) | 22 (8-50) |
|  | Foreign body in respiratory system | 2020-2023 | +12.5% (+1.8 to +24.9) | 12 (5-25) |  |  | +12.5% (+3.0 to +23.6) | 24 (9-53) |
|  | Dislocation of knee | 2020-2023 | +12.5% (+0.8 to +23.7) | 8 (3-19) |  |  | +12.5% (+3.3 to +21.9) | 18 (6-42) |
|  | Amputation of fingers (excluding thumb) | 2020-2023 | +12.5% (+1.0 to +25.9) | 25 (11-48) |  |  | +12.5% (+3.6 to +22.2) | 44 (18-92) |
|  | Open wound(s) | 2020-2023 | +12.5% (+1.8 to +24.7) | 847 (470-1,290) |  |  | +12.5% (+3.0 to +22.9) | 1,467 (840-2,238) |
|  | Fracture of face bones | 2020-2023 | +12.5% (+0.5 to +26.6) | 108 (53-175) |  |  | +12.5% (+3.5 to +23.7) | 164 (80-267) |
|  | Fracture of foot bones except ankle | 2020-2023 |  |  |  |  | +12.5% (+2.8 to +22.7) | 52 (21-105) |
|  | Multiple fractures, dislocations, crashes, wounds, pains, and strains | 2020-2023 | +12.4% (+1.6 to +25.3) | 52 (26-90) |  |  | +12.5% (+2.9 to +22.5) | 94 (44-164) |
|  | Fracture of radius and/or ulna | 2020-2023 | +12.5% (+1.4 to +26.7) | 86 (42-152) |  |  | +12.5% (+2.7 to +22.9) | 177 (82-313) |
|  | Fracture of clavicle, scapula, or humerus | 2020-2023 | +12.5% (+1.7 to +25.8) | 51 (23-96) |  |  | +12.5% (+2.3 to +22.6) | 103 (46-204) |
|  | Superficial injury of any part of the body | 2020-2023 | +12.5% (+0.8 to +25.5) | 142 (74-230) |  |  | +12.5% (+2.9 to +22.8) | 302 (148-513) |
|  | Amputation of upper limbs, bilateral | 2020-2023 | +12.3% (+0.8 to +27.3) | 1 (1-3) |  |  | +12.5% (+2.8 to +23.0) | 2 (1-5) |
|  | Burns, >=20% total burned surface area or >= 10% burned surface area if head/neck or hands/wrist involved w/o lower airway burns | 2020-2023 | +12.4% (+1.5 to +25.1) | 9 (4-14) |  |  | +12.5% (+1.5 to +21.8) | 21 (10-36) |
|  | Fracture of sternum and/or fracture of one or more ribs | 2020-2023 | +12.5% (+1.6 to +25.9) | 10 (4-20) | +12.4% (+0.0 to +24.8) | 5 (2-13) | +12.5% (+3.8 to +23.4) | 15 (6-34) |
|  | Amputation of toe/toes | 2020-2023 | +12.5% (+1.1 to +25.3) | 8 (3-18) |  |  | +12.5% (+3.0 to +21.4) | 15 (5-36) |
|  | Fracture of pelvis | 2020-2023 | +12.5% (+1.1 to +25.2) | 7 (2-16) |  |  | +12.5% (+3.0 to +22.1) | 16 (5-39) |
|  | Dislocation of hip | 2020-2023 | +12.5% (+0.8 to +27.8) | 8 (3-18) |  |  | +12.5% (+0.6 to +23.9) | 17 (6-42) |
|  | Fracture of vertebral column | 2020-2023 | +12.4% (+1.3 to +26.0) | 21 (9-39) |  |  | +12.4% (+3.9 to +23.1) | 41 (18-78) |
|  | Spinal cord lesion at neck level | 2020-2023 |  |  | +12.4% (+0.1 to +26.0) | 1 (0-3) | +12.4% (+3.2 to +21.6) | 3 (1-5) |
|  | Spinal cord lesion below neck level | 2020-2023 | +12.4% (+2.0 to +25.0) | 2 (1-4) | +12.5% (+0.1 to +26.7) | 1 (0-3) | +12.4% (+3.8 to +21.6) | 3 (1-7) |
|  | Crush injury | 2020-2023 | +12.5% (+0.9 to +25.3) | 3 (2-6) |  |  | +12.4% (+2.7 to +23.6) | 6 (3-11) |
|  | Amputation of lower limbs, bilateral | 2020-2023 | +12.4% (+1.1 to +26.3) | 0 (0-1) |  |  | +12.4% (+2.0 to +22.4) | 1 (0-2) |
|  | Minor TBI | 2020-2023 | +12.4% (+2.6 to +24.9) | 56 (28-95) |  |  | +12.4% (+2.6 to +23.6) | 101 (49-173) |
|  | Moderate/Severe TBI | 2020-2023 | +12.3% (+0.9 to +24.4) | 76 (46-120) |  |  | +12.3% (+3.6 to +21.6) | 122 (75-184) |
|  | Complications following therapeutic procedures | 2020-2023 | +12.3% (+1.4 to +23.0) | 30 (15-52) |  |  | +12.3% (+2.6 to +21.5) | 66 (32-122) |
|  | Amputation of lower limb, unilateral | 2020-2023 | +12.1% (+0.7 to +25.8) | 1 (0-1) |  |  | +12.0% (+2.2 to +22.6) | 1 (1-2) |
|  | Fracture of hip | 2020-2023 | +12.1% (+0.3 to +26.1) | 3 (1-6) |  |  | +12.0% (+1.9 to +21.9) | 8 (3-16) |
| <20 years | Burns, <20% total burned surface area without lower airway burns | 1990-2023 | +23.2% (+8.9 to +41.5) | 473 (262-800) | +51.1% (+30.9 to +74.4) | 758 (345-1,387) | +38.9% (+22.3 to +54.9) | 1,231 (640-2,214) |
|  | Drowning and nonfatal submersion | 1990-2023 | +21.6% (+8.1 to +37.1) | 54 (22-107) | +50.1% (+34.3 to +71.1) | 79 (24-203) | +36.9% (+22.7 to +51.4) | 133 (48-313) |
|  | Poisoning requiring urgent care | 1990-2023 | +18.8% (+6.4 to +34.2) | 517 (330-780) | +48.1% (+31.8 to +68.5) | 1,086 (597-1,795) | +36.9% (+22.3 to +53.1) | 1,603 (926-2,547) |
|  | Effect of different environmental factors | 1990-2023 | +20.9% (+6.4 to +37.5) | 93 (41-190) | +49.6% (+31.6 to +69.3) | 146 (55-350) | +36.8% (+22.3 to +50.3) | 239 (97-536) |
|  | Asphyxiation | 1990-2023 | +21.7% (+7.5 to +38.0) | 47 (18-101) | +50.2% (+32.6 to +72.1) | 66 (20-172) | +36.8% (+20.7 to +53.2) | 114 (39-269) |
|  | Foreign body in ear | 1990-2023 | +22.0% (+7.9 to +40.2) | 59 (25-118) | +50.3% (+31.1 to +72.0) | 76 (25-178) | +36.4% (+22.2 to +52.5) | 135 (52-287) |
|  | Dislocation of knee | 1990-2023 | +20.5% (+6.4 to +36.5) | 57 (24-116) | +49.4% (+31.0 to +70.1) | 84 (25-204) | +36.0% (+21.9 to +49.5) | 141 (48-318) |
|  | Burns, >=20% total burned surface area or >= 10% burned surface area if head/neck or hands/wrist involved w/o lower airway burns | 1990-2023 | +18.0% (+5.0 to +33.9) | 62 (38-90) | +48.3% (+30.8 to +69.0) | 117 (69-186) | +36.0% (+22.7 to +51.6) | 179 (107-272) |
|  | Lower airway burns | 1990-2023 | +19.9% (+8.1 to +37.0) | 4 (2-10) | +49.8% (+30.4 to +71.7) | 6 (2-17) | +35.6% (+21.8 to +50.8) | 11 (4-25) |
|  | Contusion in any part of the body | 1990-2023 | +19.2% (+6.7 to +34.1) | 479 (304-713) | +48.3% (+32.1 to +69.1) | 762 (429-1,222) | +35.4% (+22.1 to +50.3) | 1,241 (738-1,967) |
|  | Amputation of lower limbs, bilateral | 1990-2023 | +19.5% (+5.5 to +36.3) | 3 (1-7) | +49.4% (+30.3 to +71.3) | 5 (1-11) | +35.3% (+20.0 to +49.5) | 8 (3-19) |
|  | Amputation of thumb | 1990-2023 | +20.5% (+6.7 to +37.4) | 66 (27-145) | +49.2% (+30.6 to +69.5) | 82 (27-206) | +34.9% (+20.7 to +50.7) | 147 (55-345) |
|  | Nerve injury | 1990-2023 | +20.8% (+6.9 to +36.8) | 160 (82-290) | +49.7% (+31.7 to +72.1) | 176 (75-389) | +34.4% (+20.3 to +48.7) | 337 (161-682) |
|  | Muscle and tendon injuries, including sprains and strains lesser dislocations | 1990-2023 | +19.1% (+5.2 to +34.7) | 1,139 (774-1,638) | +48.2% (+31.3 to +67.7) | 1,572 (972-2,464) | +34.4% (+19.9 to +49.1) | 2,712 (1,750-4,114) |
|  | Dislocation of hip | 1990-2023 | +18.8% (+6.3 to +34.5) | 53 (22-106) | +47.7% (+30.0 to +68.1) | 76 (24-180) | +34.2% (+20.8 to +48.5) | 128 (46-283) |
|  | Foreign body in GI and urogenital system | 1990-2023 | +19.8% (+7.2 to +37.1) | 95 (43-187) | +48.8% (+31.5 to +70.6) | 115 (39-279) | +34.1% (+20.5 to +49.3) | 210 (82-463) |
|  | Superficial injury of any part of the body | 1990-2023 | +18.0% (+4.6 to +34.2) | 909 (616-1,301) | +47.3% (+31.0 to +67.3) | 1,328 (810-1,994) | +33.7% (+20.0 to +49.0) | 2,237 (1,446-3,216) |
|  | Fracture of foot bones except ankle | 1990-2023 | +20.3% (+6.1 to +36.3) | 196 (100-343) | +49.1% (+31.4 to +69.4) | 204 (88-442) | +33.5% (+19.8 to +48.7) | 400 (195-784) |
|  | Foreign body in respiratory system | 1990-2023 | +18.3% (+5.4 to +34.6) | 75 (36-151) | +47.4% (+32.0 to +67.8) | 103 (38-248) | +33.5% (+19.3 to +47.9) | 178 (76-397) |
|  | Amputation of toe/toes | 1990-2023 | +18.8% (+4.6 to +35.1) | 52 (23-106) | +47.7% (+29.8 to +67.7) | 64 (22-158) | +33.2% (+20.2 to +47.7) | 116 (46-257) |
|  | Fracture of clavicle, scapula, or humerus | 1990-2023 | +17.8% (+4.7 to +32.5) | 326 (182-547) | +47.2% (+29.8 to +67.6) | 437 (220-798) | +33.0% (+19.0 to +46.3) | 763 (413-1,359) |
|  | Spinal cord lesion below neck level | 1990-2023 | +20.1% (+7.6 to +34.4) | 13 (6-25) | +49.7% (+34.1 to +68.6) | 12 (4-28) | +32.9% (+19.0 to +46.2) | 25 (11-53) |
|  | Dislocation of shoulder | 1990-2023 | +17.7% (+4.7 to +34.7) | 70 (33-140) | +47.1% (+31.0 to +67.6) | 94 (36-218) | +32.8% (+18.4 to +47.5) | 164 (71-346) |
|  | Fracture of radius and/or ulna | 1990-2023 | +17.2% (+3.6 to +33.2) | 546 (326-858) | +46.6% (+29.9 to +67.0) | 757 (420-1,244) | +32.6% (+18.9 to +45.9) | 1,302 (753-2,107) |
|  | Injury to eyes | 1990-2023 | +19.7% (+6.5 to +35.1) | 573 (345-897) | +48.5% (+31.1 to +68.8) | 563 (280-951) | +32.5% (+19.3 to +45.2) | 1,136 (639-1,851) |
|  | Fracture of femur, other than femoral neck | 1990-2023 | +18.0% (+5.2 to +34.1) | 319 (169-557) | +47.4% (+29.9 to +68.2) | 379 (169-763) | +32.3% (+18.2 to +47.4) | 698 (340-1,317) |
|  | Amputation of fingers (excluding thumb) | 1990-2023 | +19.2% (+4.4 to +36.2) | 166 (80-294) | +48.4% (+29.9 to +68.4) | 161 (66-344) | +32.1% (+17.6 to +45.9) | 327 (153-623) |
|  | Amputation of upper limb, unilateral | 1990-2023 | +17.9% (+3.3 to +34.1) | 7 (3-13) | +48.3% (+30.6 to +69.1) | 7 (2-18) | +31.9% (+17.5 to +46.6) | 13 (5-31) |
|  | Fracture of pelvis | 1990-2023 | +14.9% (+2.6 to +30.2) | 41 (17-97) | +44.8% (+27.9 to +65.2) | 69 (23-172) | +31.8% (+18.1 to +45.5) | 110 (41-271) |
|  | Fracture of vertebral column | 1990-2023 | +17.0% (+3.8 to +32.5) | 132 (61-245) | +46.6% (+29.9 to +65.9) | 163 (62-338) | +31.6% (+17.9 to +45.4) | 295 (128-583) |
|  | Fracture of patella, tibia or fibula, or ankle | 1990-2023 | +18.0% (+5.9 to +32.1) | 578 (348-866) | +47.4% (+29.5 to +65.8) | 600 (321-1,000) | +31.4% (+19.2 to +44.7) | 1,178 (686-1,816) |
|  | Open wound(s) | 1990-2023 | +18.6% (+5.4 to +34.2) | 5,498 (4,154-7,176) | +47.6% (+30.5 to +67.2) | 5,215 (3,658-6,987) | +31.2% (+17.5 to +45.4) | 10,713 (7,857-13,916) |
|  | Fracture of hand (wrist and other distal part of hand) | 1990-2023 | +18.9% (+5.4 to +34.7) | 454 (278-689) | +48.2% (+31.0 to +69.4) | 401 (230-662) | +31.2% (+18.7 to +44.8) | 855 (515-1,326) |
|  | Amputation of upper limbs, bilateral | 1990-2023 | +18.3% (+4.8 to +33.8) | 10 (4-20) | +48.7% (+33.0 to +69.1) | 9 (3-22) | +31.0% (+18.3 to +46.7) | 19 (7-40) |
|  | Minor TBI | 1990-2023 | +16.9% (+4.4 to +32.5) | 355 (215-550) | +46.5% (+29.3 to +67.5) | 370 (192-624) | +30.4% (+17.2 to +45.4) | 724 (410-1,162) |
|  | Multiple fractures, dislocations, crashes, wounds, pains, and strains | 1990-2023 | +17.0% (+4.1 to +32.3) | 332 (194-543) | +46.4% (+29.3 to +66.8) | 342 (171-604) | +30.3% (+17.8 to +43.3) | 674 (376-1,142) |
|  | Fracture of face bones | 1990-2023 | +19.3% (+6.7 to +33.7) | 716 (472-1,070) | +48.3% (+31.4 to +69.6) | 478 (278-822) | +29.7% (+17.1 to +43.4) | 1,195 (759-1,874) |
|  | Fracture of skull | 1990-2023 | +20.4% (+7.1 to +35.9) | 412 (238-661) | +49.2% (+33.3 to +69.7) | 220 (107-441) | +29.4% (+16.2 to +42.3) | 632 (350-1,105) |
|  | Complications following therapeutic procedures | 1990-2023 |  |  | +42.8% (+27.3 to +62.6) | 277 (153-484) | +29.0% (+15.5 to +43.1) | 451 (263-755) |
|  | Internal hemorrhage in abdomen and pelvis | 1990-2023 | +19.5% (+6.6 to +37.4) | 1,388 (929-1,922) | +48.6% (+30.2 to +67.9) | 764 (452-1,225) | +28.7% (+15.5 to +43.0) | 2,152 (1,403-3,130) |
|  | Spinal cord lesion at neck level | 1990-2023 | +14.8% (+2.1 to +31.1) | 10 (5-20) | +45.8% (+29.6 to +68.0) | 10 (4-23) | +28.5% (+14.8 to +43.1) | 21 (10-44) |
|  | Crush injury | 1990-2023 | +15.6% (+3.3 to +30.1) | 24 (13-42) | +46.5% (+29.1 to +65.7) | 20 (9-38) | +28.0% (+14.6 to +40.8) | 44 (23-78) |
|  | Severe chest Injury | 1990-2023 | +18.9% (+6.2 to +34.9) | 830 (523-1,241) | +48.0% (+30.7 to +70.0) | 428 (235-746) | +27.8% (+15.1 to +41.0) | 1,258 (752-1,950) |
|  | Fracture of sternum and/or fracture of one or more ribs | 1990-2023 | +16.3% (+2.2 to +30.9) | 61 (28-117) | +46.1% (+29.5 to +67.0) | 42 (16-100) | +27.1% (+13.3 to +40.5) | 103 (44-211) |
|  | Moderate/Severe TBI | 1990-2023 |  |  | +45.1% (+28.8 to +64.7) | 393 (278-528) | +25.7% (+12.2 to +39.4) | 902 (666-1,175) |
|  | Fracture of hip | 1990-2023 |  |  | +34.3% (+19.9 to +53.6) | 29 (13-65) | +20.5% (+9.5 to +34.4) | 45 (21-93) |
|  | Amputation of lower limb, unilateral | 1990-2023 |  |  | +34.8% (+19.6 to +54.0) | 5 (2-8) | +17.9% (+6.5 to +30.4) | 8 (4-13) |

## Slovakia

| **Age** | **Category** | **Period** | **Male %Change** | **Male Incidence 2023** | **Female %Change** | **Female Incidence 2023** | **Both %Change** | **Both Incidence 2023** |
| --- | --- | --- | --- | --- | --- | --- | --- | --- |
| <5 years | Poisoning requiring urgent care | 1990-2023 | +15.4% (+2.2 to +29.6) | 81 (47-127) | +26.9% (+13.9 to +41.0) | 170 (95-285) | +22.9% (+11.8 to +34.5) | 251 (147-412) |
|  | Burns, >=20% total burned surface area or >= 10% burned surface area if head/neck or hands/wrist involved w/o lower airway burns | 1990-2023 | +15.3% (+2.3 to +29.7) | 10 (6-14) | +27.0% (+13.5 to +41.9) | 18 (11-30) | +22.7% (+12.5 to +33.4) | 28 (16-44) |
|  | Fracture of hip | 1990-2023 | +15.4% (+2.8 to +29.9) | 2 (1-4) | +26.8% (+12.2 to +42.9) | 4 (2-9) | +22.6% (+11.4 to +36.0) | 6 (3-13) |
|  | Fracture of pelvis | 1990-2023 | +15.4% (+1.0 to +31.1) | 6 (3-15) | +27.0% (+13.7 to +43.1) | 11 (3-29) | +22.4% (+11.2 to +34.9) | 17 (6-43) |
|  | Complications following therapeutic procedures | 1990-2023 | +15.4% (+3.3 to +30.4) | 26 (15-41) | +26.8% (+13.9 to +42.5) | 42 (22-75) | +22.1% (+12.8 to +33.7) | 68 (37-118) |
|  | Contusion in any part of the body | 1990-2023 | +15.3% (+3.6 to +30.1) | 75 (48-118) | +26.8% (+13.3 to +42.4) | 120 (66-207) | +22.1% (+12.5 to +33.2) | 195 (115-317) |
|  | Burns, <20% total burned surface area without lower airway burns | 1990-2023 | +15.3% (+2.9 to +31.0) | 76 (40-127) | +26.9% (+12.0 to +43.8) | 121 (57-213) | +22.1% (+10.8 to +33.9) | 197 (102-346) |
|  | Amputation of lower limbs, bilateral | 1990-2023 | +15.5% (+1.4 to +30.5) | 1 (0-1) | +27.3% (+12.8 to +43.9) | 1 (0-2) | +22.1% (+10.8 to +32.4) | 1 (0-3) |
|  | Effect of different environmental factors | 1990-2023 | +15.3% (+2.7 to +29.4) | 15 (7-29) | +26.8% (+12.0 to +41.8) | 23 (9-58) | +22.0% (+11.8 to +33.4) | 38 (16-84) |
|  | Dislocation of knee | 1990-2023 | +15.6% (+1.3 to +28.4) | 9 (3-18) | +26.9% (+11.5 to +44.2) | 13 (4-32) | +22.0% (+11.1 to +32.7) | 22 (7-49) |
|  | Lower airway burns | 1990-2023 | +15.2% (+2.2 to +32.1) | 1 (0-2) | +27.4% (+12.3 to +44.9) | 1 (0-3) | +22.0% (+10.9 to +33.7) | 2 (1-4) |
|  | Drowning and nonfatal submersion | 1990-2023 | +15.4% (+3.2 to +28.1) | 9 (4-17) | +26.9% (+13.4 to +43.6) | 12 (4-30) | +21.9% (+11.8 to +33.5) | 21 (8-47) |
|  | Superficial injury of any part of the body | 1990-2023 | +15.4% (+2.7 to +29.1) | 141 (90-206) | +26.8% (+13.1 to +42.7) | 207 (121-312) | +21.9% (+12.5 to +34.2) | 348 (212-517) |
|  | Dislocation of hip | 1990-2023 | +15.3% (+2.3 to +29.3) | 8 (4-17) | +26.9% (+12.6 to +42.2) | 12 (4-30) | +21.9% (+11.4 to +33.6) | 20 (7-45) |
|  | Foreign body in respiratory system | 1990-2023 | +15.5% (+3.1 to +29.2) | 12 (5-24) | +27.0% (+13.2 to +41.8) | 16 (6-38) | +21.9% (+11.2 to +32.6) | 28 (11-61) |
|  | Amputation of lower limb, unilateral | 1990-2023 | +15.4% (+1.7 to +30.7) | 0 (0-1) | +26.9% (+12.9 to +41.9) | 1 (0-1) | +21.8% (+12.1 to +32.8) | 1 (1-2) |
|  | Fracture of radius and/or ulna | 1990-2023 | +15.3% (+2.4 to +30.1) | 84 (50-131) | +26.9% (+11.8 to +44.0) | 118 (64-193) | +21.8% (+12.4 to +32.6) | 202 (116-318) |
|  | Asphyxiation | 1990-2023 | +15.3% (+2.5 to +29.6) | 8 (3-17) | +26.9% (+13.1 to +43.0) | 11 (3-28) | +21.7% (+11.1 to +34.4) | 18 (6-44) |
|  | Muscle and tendon injuries, including sprains and strains lesser dislocations | 1990-2023 | +15.3% (+1.8 to +30.3) | 178 (118-255) | +26.8% (+11.8 to +43.7) | 247 (150-380) | +21.7% (+10.9 to +33.2) | 425 (272-630) |
|  | Fracture of clavicle, scapula, or humerus | 1990-2023 | +15.3% (+3.2 to +29.4) | 51 (27-91) | +26.9% (+13.3 to +43.9) | 68 (33-124) | +21.7% (+12.7 to +33.5) | 119 (63-215) |
|  | Amputation of thumb | 1990-2023 | +15.4% (+1.3 to +31.5) | 10 (4-22) | +27.1% (+11.6 to +43.1) | 13 (4-32) | +21.6% (+10.7 to +33.4) | 23 (9-52) |
|  | Dislocation of shoulder | 1990-2023 | +15.2% (+1.7 to +30.3) | 11 (5-21) | +26.8% (+12.8 to +41.6) | 15 (5-33) | +21.6% (+11.7 to +32.2) | 25 (11-54) |
|  | Foreign body in ear | 1990-2023 | +15.3% (+0.9 to +30.1) | 9 (4-19) | +26.9% (+11.2 to +41.3) | 12 (4-27) | +21.6% (+10.0 to +33.3) | 22 (8-45) |
|  | Amputation of toe/toes | 1990-2023 | +15.4% (+2.1 to +31.1) | 8 (4-16) | +26.8% (+12.8 to +44.0) | 10 (3-26) | +21.5% (+11.2 to +33.0) | 18 (7-40) |
|  | Fracture of femur, other than femoral neck | 1990-2023 | +15.5% (+2.2 to +30.6) | 49 (26-90) | +27.0% (+13.0 to +41.8) | 59 (27-118) | +21.5% (+12.3 to +32.7) | 108 (54-209) |
|  | Fracture of vertebral column | 1990-2023 | +15.3% (+1.9 to +29.6) | 20 (10-39) | +26.8% (+13.8 to +42.8) | 25 (10-54) | +21.4% (+10.9 to +32.9) | 46 (20-93) |
|  | Foreign body in GI and urogenital system | 1990-2023 | +15.4% (+1.2 to +29.9) | 15 (7-28) | +26.8% (+11.9 to +42.5) | 18 (6-46) | +21.4% (+12.2 to +33.7) | 33 (13-77) |
|  | Nerve injury | 1990-2023 | +15.4% (+3.2 to +29.3) | 25 (13-47) | +27.0% (+12.0 to +43.4) | 28 (12-64) | +21.2% (+12.5 to +32.0) | 53 (24-110) |
|  | Amputation of upper limb, unilateral | 1990-2023 | +15.2% (+2.7 to +30.4) | 1 (0-2) | +27.1% (+13.3 to +44.7) | 1 (0-3) | +21.0% (+11.2 to +32.8) | 2 (1-5) |
|  | Fracture of patella, tibia or fibula, or ankle | 1990-2023 | +15.4% (+2.4 to +30.6) | 90 (54-138) | +26.9% (+13.7 to +43.7) | 94 (49-156) | +21.0% (+11.8 to +32.0) | 184 (106-282) |
|  | Minor TBI | 1990-2023 | +15.4% (+2.8 to +29.5) | 55 (33-88) | +26.8% (+12.5 to +42.6) | 57 (29-99) | +21.0% (+11.5 to +32.7) | 112 (61-185) |
|  | Fracture of foot bones except ankle | 1990-2023 | +15.4% (+2.7 to +31.2) | 31 (16-56) | +26.9% (+11.9 to +44.1) | 32 (14-68) | +21.0% (+9.7 to +32.2) | 63 (31-124) |
|  | Multiple fractures, dislocations, crashes, wounds, pains, and strains | 1990-2023 | +15.3% (+3.5 to +29.5) | 51 (29-83) | +26.8% (+13.4 to +43.3) | 53 (26-95) | +20.9% (+11.6 to +32.0) | 104 (55-178) |
|  | Spinal cord lesion below neck level | 1990-2023 | +15.4% (+4.0 to +29.0) | 2 (1-4) | +27.3% (+13.8 to +43.2) | 2 (1-5) | +20.8% (+11.3 to +31.1) | 4 (2-8) |
|  | Injury to eyes | 1990-2023 | +15.4% (+2.5 to +29.8) | 90 (54-141) | +26.9% (+12.1 to +43.6) | 89 (47-155) | +20.8% (+11.2 to +31.8) | 179 (105-290) |
|  | Amputation of fingers (excluding thumb) | 1990-2023 | +15.4% (+1.5 to +30.4) | 26 (13-47) | +27.0% (+11.6 to +44.4) | 25 (10-53) | +20.8% (+10.4 to +32.0) | 51 (24-100) |
|  | Spinal cord lesion at neck level | 1990-2023 | +15.2% (+1.0 to +31.2) | 2 (1-3) | +26.9% (+12.6 to +44.3) | 2 (1-4) | +20.8% (+10.6 to +31.8) | 3 (1-7) |
|  | Open wound(s) | 1990-2023 | +15.3% (+2.7 to +29.4) | 856 (618-1,115) | +26.8% (+13.5 to +43.6) | 817 (557-1,147) | +20.7% (+11.8 to +30.9) | 1,673 (1,199-2,226) |
|  | Amputation of upper limbs, bilateral | 1990-2023 | +15.3% (+1.6 to +29.5) | 2 (1-3) | +27.1% (+12.4 to +43.4) | 1 (0-3) | +20.6% (+9.9 to +33.0) | 3 (1-7) |
|  | Fracture of hand (wrist and other distal part of hand) | 1990-2023 | +15.3% (+3.6 to +30.6) | 71 (41-108) | +26.9% (+13.2 to +42.4) | 63 (33-104) | +20.5% (+10.8 to +32.0) | 134 (75-209) |
|  | Crush injury | 1990-2023 | +15.4% (+2.0 to +28.8) | 4 (2-6) | +27.0% (+13.0 to +44.2) | 3 (1-6) | +20.4% (+10.8 to +30.5) | 7 (3-12) |
|  | Moderate/Severe TBI | 1990-2023 | +15.3% (+2.6 to +29.6) | 77 (57-103) | +26.9% (+13.2 to +43.2) | 60 (41-83) | +20.2% (+10.9 to +31.5) | 137 (100-188) |
|  | Fracture of sternum and/or fracture of one or more ribs | 1990-2023 | +15.3% (+2.2 to +28.7) | 9 (4-17) | +26.8% (+13.4 to +43.3) | 6 (2-15) | +19.8% (+10.9 to +31.5) | 16 (7-32) |
|  | Fracture of face bones | 1990-2023 | +15.4% (+2.7 to +30.1) | 112 (69-171) | +26.9% (+13.1 to +42.9) | 75 (41-126) | +19.7% (+10.4 to +31.3) | 188 (115-300) |
|  | Internal hemorrhage in abdomen and pelvis | 1990-2023 | +15.4% (+2.2 to +30.1) | 218 (137-312) | +26.8% (+13.2 to +43.1) | 120 (72-197) | +19.2% (+10.1 to +30.4) | 338 (207-495) |
|  | Fracture of skull | 1990-2023 | +15.3% (+2.2 to +31.5) | 65 (37-106) | +26.9% (+14.5 to +42.9) | 35 (17-66) | +19.2% (+9.6 to +31.2) | 100 (54-177) |
|  | Severe chest Injury | 1990-2023 | +15.3% (+1.1 to +30.8) | 130 (80-198) | +26.8% (+12.8 to +42.8) | 67 (37-118) | +19.0% (+8.4 to +30.6) | 197 (118-302) |
| 5-14 years | Poisoning requiring urgent care | 2020-2023 |  |  |  |  | +11.9% (+1.5 to +21.7) | 51 (25-92) |
|  | Burns, >=20% total burned surface area or >= 10% burned surface area if head/neck or hands/wrist involved w/o lower airway burns | 2020-2023 |  |  |  |  | +11.7% (+2.6 to +19.7) | 5 (3-9) |
|  | Fracture of hip | 2020-2023 |  |  |  |  | +11.7% (+1.4 to +20.8) | 2 (1-4) |
|  | Dislocation of knee | 2020-2023 |  |  |  |  | +11.7% (+2.2 to +19.5) | 4 (1-11) |
|  | Fracture of pelvis | 2020-2023 |  |  |  |  | +11.7% (+2.2 to +20.4) | 4 (1-10) |
|  | Burns, <20% total burned surface area without lower airway burns | 2020-2023 |  |  |  |  | +11.7% (+1.8 to +19.8) | 37 (16-71) |
|  | Superficial injury of any part of the body | 2020-2023 |  |  |  |  | +11.6% (+1.5 to +20.0) | 75 (36-131) |
|  | Complications following therapeutic procedures | 2020-2023 |  |  |  |  | +11.6% (+2.6 to +19.5) | 16 (8-30) |
|  | Contusion in any part of the body | 2020-2023 |  |  |  |  | +11.6% (+1.6 to +20.4) | 40 (21-70) |
|  | Amputation of lower limbs, bilateral | 2020-2023 |  |  |  |  | +11.6% (+2.1 to +19.7) | 0 (0-1) |
|  | Drowning and nonfatal submersion | 2020-2023 |  |  |  |  | +11.6% (+1.7 to +19.8) | 4 (1-10) |
|  | Lower airway burns | 2020-2023 |  |  |  |  | +11.6% (+0.8 to +20.0) | 0 (0-1) |
|  | Foreign body in respiratory system | 2020-2023 |  |  |  |  | +11.6% (+1.7 to +19.5) | 6 (2-13) |
|  | Effect of different environmental factors | 2020-2023 |  |  |  |  | +11.6% (+1.3 to +19.9) | 8 (3-17) |
|  | Dislocation of hip | 2020-2023 |  |  |  |  | +11.6% (+0.7 to +21.0) | 4 (1-10) |
|  | Fracture of femur, other than femoral neck | 2020-2023 |  |  |  |  | +11.6% (+2.0 to +20.1) | 24 (10-51) |
|  | Muscle and tendon injuries, including sprains and strains lesser dislocations | 2020-2023 |  |  |  |  | +11.5% (+1.8 to +19.2) | 89 (48-149) |
|  | Fracture of radius and/or ulna | 2020-2023 |  |  |  |  | +11.5% (+1.5 to +19.9) | 44 (21-81) |
|  | Amputation of toe/toes | 2020-2023 |  |  |  |  | +11.5% (+1.4 to +19.9) | 4 (1-9) |
|  | Fracture of clavicle, scapula, or humerus | 2020-2023 |  |  |  |  | +11.5% (+2.1 to +20.1) | 26 (11-50) |
|  | Foreign body in ear | 2020-2023 |  |  |  |  | +11.5% (+2.0 to +20.4) | 4 (1-10) |
|  | Asphyxiation | 2020-2023 |  |  |  |  | +11.5% (+1.5 to +19.6) | 4 (1-8) |
|  | Amputation of lower limb, unilateral | 2020-2023 |  |  |  |  | +11.5% (+1.3 to +19.8) | 0 (0-1) |
|  | Minor TBI | 2020-2023 |  |  |  |  | +11.4% (+1.1 to +19.4) | 25 (12-45) |
|  | Amputation of thumb | 2020-2023 |  |  |  |  | +11.4% (+0.8 to +20.1) | 5 (2-12) |
|  | Fracture of patella, tibia or fibula, or ankle | 2020-2023 |  |  |  |  | +11.4% (+2.0 to +19.3) | 40 (19-70) |
|  | Foreign body in GI and urogenital system | 2020-2023 |  |  |  |  | +11.4% (+0.9 to +19.9) | 7 (2-16) |
|  | Amputation of fingers (excluding thumb) | 2020-2023 |  |  |  |  | +11.4% (+1.2 to +19.8) | 11 (4-23) |
|  | Fracture of vertebral column | 2020-2023 |  |  |  |  | +11.4% (+1.3 to +19.8) | 10 (4-20) |
|  | Dislocation of shoulder | 2020-2023 |  |  |  |  | +11.4% (+1.2 to +18.7) | 6 (2-12) |
|  | Injury to eyes | 2020-2023 |  |  |  |  | +11.4% (+1.7 to +19.1) | 38 (19-69) |
|  | Spinal cord lesion at neck level | 2020-2023 |  |  |  |  | +11.3% (+1.4 to +18.3) | 1 (0-1) |
|  | Spinal cord lesion below neck level | 2020-2023 |  |  |  |  | +11.3% (+1.5 to +17.7) | 1 (0-2) |
|  | Nerve injury | 2020-2023 |  |  |  |  | +11.3% (+1.5 to +19.7) | 11 (4-24) |
|  | Open wound(s) | 2020-2023 |  |  |  |  | +11.3% (+0.7 to +19.3) | 366 (204-563) |
|  | Multiple fractures, dislocations, crashes, wounds, pains, and strains | 2020-2023 |  |  |  |  | +11.3% (+1.3 to +18.6) | 23 (11-44) |
|  | Fracture of foot bones except ankle | 2020-2023 |  |  |  |  | +11.3% (+1.8 to +20.4) | 13 (5-27) |
|  | Amputation of upper limb, unilateral | 2020-2023 |  |  |  |  | +11.3% (+0.4 to +20.0) | 0 (0-1) |
|  | Fracture of hand (wrist and other distal part of hand) | 2020-2023 |  |  |  |  | +11.2% (+1.5 to +19.6) | 29 (14-53) |
|  | Crush injury | 2020-2023 |  |  |  |  | +11.2% (+1.6 to +18.2) | 1 (1-3) |
|  | Moderate/Severe TBI | 2020-2023 |  |  |  |  | +11.1% (+1.4 to +18.4) | 30 (18-47) |
|  | Amputation of upper limbs, bilateral | 2020-2023 |  |  |  |  | +11.1% (+0.6 to +18.4) | 1 (0-1) |
|  | Fracture of face bones | 2020-2023 |  |  |  |  | +11.0% (+0.5 to +18.5) | 41 (20-67) |
|  | Fracture of skull | 2020-2023 |  |  |  |  | +11.0% (+0.4 to +19.4) | 22 (10-39) |
|  | Fracture of sternum and/or fracture of one or more ribs | 2020-2023 |  |  |  |  | +11.0% (+1.0 to +17.7) | 4 (1-8) |
|  | Internal hemorrhage in abdomen and pelvis | 2020-2023 |  |  |  |  | +11.0% (+1.0 to +17.8) | 74 (39-118) |
|  | Severe chest Injury | 2020-2023 |  |  |  |  | +10.9% (+0.9 to +18.7) | 44 (23-81) |
| <20 years | Burns, <20% total burned surface area without lower airway burns | 1990-2023 |  |  | +19.2% (+7.3 to +32.1) | 147 (72-266) | +12.3% (+3.7 to +21.3) | 246 (124-438) |
|  | Drowning and nonfatal submersion | 1990-2023 |  |  | +18.6% (+7.4 to +32.4) | 15 (5-38) | +11.3% (+2.5 to +21.5) | 27 (10-62) |
|  | Poisoning requiring urgent care | 1990-2023 |  |  | +17.2% (+6.6 to +29.5) | 212 (116-358) | +11.1% (+2.4 to +20.4) | 320 (186-523) |
|  | Effect of different environmental factors | 1990-2023 |  |  | +18.0% (+6.9 to +30.6) | 28 (11-68) | +11.0% (+2.1 to +20.4) | 48 (20-106) |
|  | Asphyxiation | 1990-2023 |  |  | +18.4% (+7.5 to +31.1) | 13 (4-34) | +11.0% (+2.1 to +20.7) | 23 (8-54) |
|  | Foreign body in ear | 1990-2023 |  |  | +18.7% (+6.1 to +30.6) | 15 (5-34) | +11.0% (+2.0 to +20.1) | 27 (11-58) |
|  | Dislocation of knee | 1990-2023 |  |  | +18.0% (+5.4 to +30.2) | 16 (5-40) | +10.7% (+1.9 to +20.0) | 28 (10-64) |
|  | Burns, >=20% total burned surface area or >= 10% burned surface area if head/neck or hands/wrist involved w/o lower airway burns | 1990-2023 |  |  | +17.2% (+6.3 to +29.0) | 23 (13-36) | +10.5% (+1.6 to +19.3) | 35 (21-55) |
|  | Lower airway burns | 1990-2023 |  |  | +18.4% (+6.3 to +29.7) | 1 (0-3) | +10.4% (+1.4 to +18.8) | 2 (1-5) |
|  | Amputation of lower limbs, bilateral | 1990-2023 |  |  | +18.1% (+6.9 to +30.0) | 1 (0-2) | +10.2% (+1.2 to +18.9) | 2 (1-4) |
|  | Contusion in any part of the body | 1990-2023 |  |  | +17.3% (+6.1 to +29.4) | 149 (82-248) | +10.2% (+2.0 to +18.9) | 249 (150-391) |
|  | Amputation of thumb | 1990-2023 |  |  | +18.1% (+6.1 to +29.7) | 16 (5-41) | +10.0% (+0.6 to +20.0) | 30 (11-68) |
|  | Nerve injury | 1990-2023 |  |  | +18.2% (+6.8 to +30.6) | 34 (15-77) | +9.8% (+1.4 to +18.1) | 68 (32-139) |
|  | Dislocation of hip | 1990-2023 |  |  | +17.2% (+6.1 to +30.4) | 15 (5-37) | +9.7% (+1.2 to +18.2) | 26 (9-58) |
|  | Muscle and tendon injuries, including sprains and strains lesser dislocations | 1990-2023 |  |  | +17.2% (+6.0 to +29.4) | 307 (190-473) | +9.6% (+1.8 to +18.7) | 546 (348-811) |
|  | Foreign body in GI and urogenital system | 1990-2023 |  |  | +17.5% (+6.4 to +30.0) | 22 (7-55) | +9.5% (+0.7 to +18.9) | 42 (16-97) |
|  | Fracture of foot bones except ankle | 1990-2023 |  |  | +17.8% (+6.5 to +30.4) | 40 (17-85) | +9.3% (+0.9 to +18.3) | 81 (40-157) |
|  | Foreign body in respiratory system | 1990-2023 |  |  | +16.9% (+6.6 to +28.3) | 20 (8-48) | +9.2% (+1.1 to +17.2) | 36 (15-80) |
|  | Superficial injury of any part of the body | 1990-2023 |  |  | +16.7% (+5.4 to +28.9) | 260 (154-387) | +9.2% (+1.3 to +18.4) | 451 (274-645) |
|  | Spinal cord lesion below neck level | 1990-2023 |  |  | +18.4% (+7.0 to +30.3) | 2 (1-6) | +8.9% (+0.1 to +17.1) | 5 (2-11) |
|  | Amputation of toe/toes | 1990-2023 |  |  | +16.9% (+5.4 to +29.9) | 12 (4-32) |  |  |
|  | Fracture of clavicle, scapula, or humerus | 1990-2023 |  |  | +16.6% (+5.5 to +29.6) | 85 (43-158) | +8.8% (+0.9 to +17.2) | 154 (82-278) |
|  | Injury to eyes | 1990-2023 |  |  | +17.5% (+5.9 to +30.2) | 110 (57-192) | +8.7% (+0.6 to +17.0) | 230 (134-382) |
|  | Dislocation of shoulder | 1990-2023 |  |  | +16.5% (+4.9 to +29.5) | 18 (7-41) |  |  |
|  | Fracture of radius and/or ulna | 1990-2023 |  |  | +16.3% (+5.2 to +28.5) | 148 (81-253) | +8.6% (+0.0 to +17.6) | 263 (152-420) |
|  | Fracture of femur, other than femoral neck | 1990-2023 |  |  | +16.7% (+5.8 to +29.0) | 74 (34-150) |  |  |
|  | Amputation of fingers (excluding thumb) | 1990-2023 |  |  | +17.4% (+5.1 to +30.9) | 31 (13-67) | +8.4% (+0.0 to +17.0) | 66 (31-125) |
|  | Amputation of upper limb, unilateral | 1990-2023 |  |  | +17.2% (+5.2 to +30.1) | 1 (0-3) |  |  |
|  | Fracture of pelvis | 1990-2023 |  |  | +15.2% (+4.0 to +27.4) | 14 (4-37) |  |  |
|  | Fracture of vertebral column | 1990-2023 |  |  | +16.2% (+6.0 to +28.7) | 32 (13-68) | +8.0% (+0.0 to +16.8) | 60 (26-122) |
|  | Fracture of patella, tibia or fibula, or ankle | 1990-2023 |  |  | +16.8% (+5.4 to +29.2) | 117 (63-193) |  |  |
|  | Open wound(s) | 1990-2023 |  |  | +16.9% (+5.8 to +29.4) | 1,018 (712-1,393) |  |  |
|  | Fracture of hand (wrist and other distal part of hand) | 1990-2023 |  |  | +17.2% (+6.3 to +30.3) | 78 (43-127) |  |  |
|  | Amputation of upper limbs, bilateral | 1990-2023 |  |  | +17.4% (+5.3 to +30.0) | 2 (1-4) |  |  |
|  | Minor TBI | 1990-2023 |  |  | +16.1% (+5.1 to +28.8) | 72 (37-122) |  |  |
|  | Multiple fractures, dislocations, crashes, wounds, pains, and strains | 1990-2023 |  |  | +16.1% (+5.8 to +27.8) | 67 (33-118) |  |  |
|  | Fracture of face bones | 1990-2023 |  |  | +17.4% (+7.1 to +29.8) | 93 (52-160) |  |  |
|  | Fracture of skull | 1990-2023 |  |  | +17.9% (+7.7 to +30.3) | 43 (20-84) |  |  |
|  | Internal hemorrhage in abdomen and pelvis | 1990-2023 |  |  | +17.4% (+5.5 to +29.7) | 149 (88-238) |  |  |
|  | Complications following therapeutic procedures | 1990-2023 |  |  | +13.7% (+3.7 to +25.2) | 55 (29-97) |  |  |
|  | Spinal cord lesion at neck level | 1990-2023 |  |  | +15.5% (+4.3 to +28.1) | 2 (1-4) |  |  |
|  | Severe chest Injury | 1990-2023 |  |  | +17.0% (+6.1 to +29.4) | 83 (46-145) |  |  |
|  | Crush injury | 1990-2023 |  |  | +15.9% (+5.2 to +29.4) | 4 (2-7) |  |  |
|  | Fracture of sternum and/or fracture of one or more ribs | 1990-2023 |  |  | +15.7% (+5.0 to +28.6) | 8 (3-19) |  |  |
|  | Moderate/Severe TBI | 1990-2023 |  |  | +15.0% (+4.6 to +26.7) | 76 (51-104) |  |  |

## Slovenia

| **Age** | **Category** | **Period** | **Male %Change** | **Male Incidence 2023** | **Female %Change** | **Female Incidence 2023** | **Both %Change** | **Both Incidence 2023** |
| --- | --- | --- | --- | --- | --- | --- | --- | --- |
| 15-19 years | Internal hemorrhage in abdomen and pelvis | 2020-2023 | +11.9% (+0.3 to +24.3) | 9 (4-15) |  |  |  |  |
|  | Fracture of face bones | 2020-2023 | +11.9% (+0.4 to +25.9) | 5 (2-8) |  |  | +10.2% (+0.2 to +21.2) | 6 (3-10) |
|  | Moderate/Severe TBI | 2020-2023 | +11.8% (+0.1 to +24.0) | 4 (2-7) |  |  | +10.0% (+0.3 to +20.3) | 6 (3-10) |
|  | Crush injury | 2020-2023 |  |  |  |  | +10.0% (+0.0 to +20.3) | 0 (0-0) |
|  | Fracture of hand (wrist and other distal part of hand) | 2020-2023 |  |  |  |  | +9.9% (+0.8 to +20.9) | 4 (2-7) |
|  | Open wound(s) | 2020-2023 |  |  |  |  | +9.8% (+0.1 to +21.1) | 52 (27-85) |
|  | Injury to eyes | 2020-2023 | +11.8% (+0.1 to +26.0) | 4 (2-6) |  |  | +9.7% (+0.3 to +21.4) | 5 (2-9) |
|  | Multiple fractures, dislocations, crashes, wounds, pains, and strains | 2020-2023 | +11.9% (+0.0 to +26.1) | 2 (1-5) |  |  | +9.7% (+0.9 to +19.5) | 3 (2-7) |
|  | Fracture of foot bones except ankle | 2020-2023 |  |  |  |  | +9.7% (+0.0 to +21.3) | 2 (1-4) |
|  | Fracture of patella, tibia or fibula, or ankle | 2020-2023 | +11.9% (+0.9 to +27.1) | 4 (2-7) |  |  | +9.7% (+1.1 to +21.4) | 6 (3-10) |
|  | Minor TBI | 2020-2023 | +11.8% (+0.2 to +25.6) | 3 (1-5) |  |  | +9.6% (+0.4 to +20.7) | 4 (2-7) |
|  | Amputation of toe/toes | 2020-2023 |  |  |  |  | +9.6% (+0.6 to +20.7) | 1 (0-1) |
|  | Dislocation of shoulder | 2020-2023 | +12.0% (+0.6 to +25.1) | 0 (0-1) |  |  |  |  |
|  | Amputation of thumb | 2020-2023 |  |  |  |  | +9.4% (+0.2 to +21.9) | 1 (0-1) |
|  | Fracture of clavicle, scapula, or humerus | 2020-2023 | +11.9% (+0.2 to +24.8) | 2 (1-4) |  |  | +9.3% (+0.3 to +20.4) | 4 (2-6) |
|  | Drowning and nonfatal submersion | 2020-2023 | +11.8% (+0.1 to +27.4) | 0 (0-1) |  |  |  |  |
|  | Contusion in any part of the body | 2020-2023 |  |  |  |  | +9.1% (+0.4 to +20.2) | 5 (2-9) |
|  | Fracture of pelvis | 2020-2023 | +12.1% (+0.3 to +27.3) | 0 (0-1) |  |  | +9.1% (+0.2 to +18.5) | 1 (0-1) |
|  | Complications following therapeutic procedures | 2020-2023 |  |  |  |  | +9.1% (+0.1 to +20.2) | 3 (1-5) |
|  | Dislocation of hip | 2020-2023 |  |  |  |  | +9.1% (+0.0 to +20.5) | 1 (0-1) |
|  | Burns, >=20% total burned surface area or >= 10% burned surface area if head/neck or hands/wrist involved w/o lower airway burns | 2020-2023 |  |  |  |  | +8.9% (+0.0 to +21.3) | 1 (0-1) |
|  | Poisoning requiring urgent care | 2020-2023 |  |  |  |  | +8.7% (+0.8 to +19.6) | 7 (3-12) |

## Spain

| **Age** | **Category** | **Period** | **Male %Change** | **Male Incidence 2023** | **Female %Change** | **Female Incidence 2023** | **Both %Change** | **Both Incidence 2023** |
| --- | --- | --- | --- | --- | --- | --- | --- | --- |
| 5-14 years | Internal hemorrhage in abdomen and pelvis | 2020-2023 |  |  |  |  | +10.4% (+0.6 to +21.6) | 283 (144-479) |
|  | Fracture of skull | 2020-2023 |  |  |  |  | +10.4% (+2.0 to +20.1) | 372 (170-665) |
|  | Severe chest Injury | 2020-2023 |  |  |  |  | +10.4% (+0.7 to +20.2) | 216 (106-389) |
|  | Fracture of sternum and/or fracture of one or more ribs | 2020-2023 |  |  |  |  | +10.2% (+0.8 to +20.8) | 112 (43-250) |
|  | Fracture of face bones | 2020-2023 |  |  |  |  | +10.2% (+0.9 to +20.2) | 531 (250-905) |
|  | Moderate/Severe TBI | 2020-2023 | +11.7% (+0.4 to +24.3) | 45 (26-72) |  |  | +10.1% (+1.1 to +19.4) | 75 (41-121) |
|  | Amputation of upper limbs, bilateral | 2020-2023 |  |  |  |  | +10.1% (+1.2 to +19.5) | 2 (1-5) |
|  | Crush injury | 2020-2023 |  |  |  |  | +10.0% (+0.0 to +19.5) | 2 (1-4) |
|  | Spinal cord lesion at neck level | 2020-2023 |  |  |  |  | +9.9% (+0.3 to +19.7) | 5 (2-10) |
|  | Open wound(s) | 2020-2023 | +11.5% (+0.5 to +24.3) | 1,215 (642-1,948) |  |  | +9.9% (+1.2 to +19.4) | 2,140 (1,134-3,495) |
|  | Amputation of fingers (excluding thumb) | 2020-2023 |  |  |  |  | +9.9% (+0.6 to +20.4) | 42 (14-94) |
|  | Spinal cord lesion below neck level | 2020-2023 |  |  |  |  | +9.9% (+0.2 to +19.6) | 7 (2-17) |
|  | Amputation of upper limb, unilateral | 2020-2023 | +11.6% (+0.3 to +24.4) | 1 (0-3) |  |  | +9.9% (+1.3 to +20.3) | 2 (1-5) |
|  | Fracture of patella, tibia or fibula, or ankle | 2020-2023 |  |  |  |  | +9.8% (+0.5 to +18.5) | 187 (84-339) |
|  | Foreign body in GI and urogenital system | 2020-2023 |  |  |  |  | +9.8% (+0.2 to +20.2) | 47 (15-130) |
|  | Amputation of lower limb, unilateral | 2020-2023 |  |  |  |  | +9.8% (+0.5 to +19.8) | 0 (0-0) |
|  | Injury to eyes | 2020-2023 |  |  |  |  | +9.8% (+0.5 to +18.9) | 410 (192-761) |
|  | Minor TBI | 2020-2023 |  |  |  |  | +9.8% (+1.2 to +18.9) | 314 (151-598) |
|  | Multiple fractures, dislocations, crashes, wounds, pains, and strains | 2020-2023 |  |  |  |  | +9.7% (+1.7 to +19.2) | 68 (29-126) |
|  | Amputation of toe/toes | 2020-2023 | +11.6% (+1.0 to +25.7) | 17 (6-41) |  |  | +9.7% (+0.5 to +20.9) | 34 (10-81) |
|  | Dislocation of hip | 2020-2023 |  |  |  |  | +9.7% (+0.6 to +19.5) | 36 (11-91) |
|  | Fracture of femur, other than femoral neck | 2020-2023 |  |  |  |  | +9.7% (+0.8 to +20.2) | 64 (26-138) |
|  | Nerve injury | 2020-2023 | +11.4% (+0.0 to +24.1) | 58 (23-120) |  |  | +9.7% (+1.2 to +18.7) | 109 (39-244) |
|  | Fracture of vertebral column | 2020-2023 |  |  |  |  | +9.7% (+0.6 to +19.7) | 71 (29-148) |
|  | Lower airway burns | 2020-2023 | +11.6% (+0.0 to +25.7) | 1 (0-3) |  |  | +9.7% (+1.4 to +19.9) | 3 (1-7) |
|  | Amputation of thumb | 2020-2023 |  |  |  |  | +9.6% (+0.7 to +20.6) | 47 (14-114) |
|  | Asphyxiation | 2020-2023 |  |  |  |  | +9.6% (+1.2 to +18.6) | 60 (16-171) |
|  | Complications following therapeutic procedures | 2020-2023 |  |  |  |  | +9.5% (+1.2 to +18.6) | 18 (8-34) |
|  | Dislocation of knee | 2020-2023 |  |  |  |  | +9.5% (+0.6 to +18.3) | 54 (16-144) |
|  | Fracture of radius and/or ulna | 2020-2023 |  |  |  |  | +9.5% (+1.2 to +18.7) | 159 (69-303) |
|  | Superficial injury of any part of the body | 2020-2023 |  |  |  |  | +9.5% (+0.4 to +19.2) | 1,487 (701-2,501) |
|  | Muscle and tendon injuries, including sprains and strains lesser dislocations | 2020-2023 | +11.5% (+0.2 to +24.2) | 178 (89-311) |  |  | +9.5% (+1.0 to +18.8) | 376 (193-664) |
|  | Foreign body in respiratory system | 2020-2023 |  |  |  |  | +9.5% (+0.0 to +19.1) | 35 (11-86) |
|  | Foreign body in ear | 2020-2023 |  |  |  |  | +9.5% (+0.2 to +18.0) | 56 (17-138) |
|  | Dislocation of shoulder | 2020-2023 |  |  |  |  | +9.4% (+0.3 to +19.1) | 76 (24-179) |
|  | Effect of different environmental factors | 2020-2023 |  |  |  |  | +9.4% (+0.1 to +18.6) | 63 (22-151) |
|  | Fracture of hip | 2020-2023 |  |  |  |  | +9.4% (+1.4 to +18.4) | 11 (4-25) |
|  | Contusion in any part of the body | 2020-2023 |  |  |  |  | +9.4% (+1.6 to +18.3) | 1,205 (657-2,138) |
|  | Amputation of lower limbs, bilateral | 2020-2023 |  |  |  |  | +9.3% (+0.5 to +19.7) | 2 (1-6) |
|  | Burns, >=20% total burned surface area or >= 10% burned surface area if head/neck or hands/wrist involved w/o lower airway burns | 2020-2023 |  |  |  |  | +9.3% (+0.7 to +18.9) | 6 (2-12) |
|  | Drowning and nonfatal submersion | 2020-2023 | +11.3% (+0.4 to +25.1) | 26 (8-62) |  |  | +9.2% (+0.7 to +20.2) | 56 (15-150) |
|  | Poisoning requiring urgent care | 2020-2023 | +11.5% (+0.6 to +24.4) | 44 (21-77) |  |  | +9.0% (+1.5 to +18.9) | 119 (55-211) |

## Sweden

| **Age** | **Category** | **Period** | **Male %Change** | **Male Incidence 2023** | **Female %Change** | **Female Incidence 2023** | **Both %Change** | **Both Incidence 2023** |
| --- | --- | --- | --- | --- | --- | --- | --- | --- |
| 5-14 years | Internal hemorrhage in abdomen and pelvis | 2020-2023 |  |  |  |  | +9.9% (+0.4 to +19.3) | 75 (40-123) |
|  | Severe chest Injury | 2020-2023 |  |  |  |  | +9.9% (+0.4 to +19.5) | 57 (29-104) |
|  | Fracture of skull | 2020-2023 |  |  |  |  | +9.9% (+1.0 to +19.1) | 99 (46-176) |
|  | Fracture of sternum and/or fracture of one or more ribs | 2020-2023 |  |  |  |  | +9.8% (+0.6 to +20.0) | 30 (12-65) |
|  | Fracture of face bones | 2020-2023 |  |  |  |  | +9.8% (+0.3 to +19.5) | 141 (68-240) |
|  | Amputation of toe/toes | 2020-2023 |  |  |  |  | +9.8% (+1.1 to +18.8) | 9 (3-22) |
|  | Spinal cord lesion below neck level | 2020-2023 |  |  |  |  | +9.7% (+0.7 to +19.5) | 2 (1-4) |
|  | Fracture of hand (wrist and other distal part of hand) | 2020-2023 |  |  |  |  | +9.6% (+0.2 to +19.1) | 118 (57-223) |
|  | Crush injury | 2020-2023 | +10.6% (+0.2 to +23.1) | 0 (0-1) |  |  | +9.6% (+0.3 to +20.3) | 1 (0-1) |
|  | Spinal cord lesion at neck level | 2020-2023 |  |  |  |  | +9.6% (+1.2 to +19.2) | 1 (0-3) |
|  | Amputation of upper limbs, bilateral | 2020-2023 |  |  |  |  | +9.6% (+1.3 to +19.4) | 1 (0-1) |
|  | Open wound(s) | 2020-2023 |  |  |  |  | +9.6% (+1.5 to +19.4) | 563 (321-897) |
|  | Minor TBI | 2020-2023 |  |  |  |  | +9.6% (+0.3 to +19.1) | 82 (41-152) |
|  | Fracture of patella, tibia or fibula, or ankle | 2020-2023 |  |  |  |  | +9.6% (+0.5 to +18.5) | 49 (23-88) |
|  | Fracture of foot bones except ankle | 2020-2023 |  |  |  |  | +9.6% (+0.6 to +21.2) | 23 (9-49) |
|  | Dislocation of knee | 2020-2023 |  |  |  |  | +9.5% (+0.7 to +20.2) | 14 (4-37) |
|  | Nerve injury | 2020-2023 |  |  |  |  | +9.5% (+0.5 to +17.6) | 29 (11-63) |
|  | Amputation of fingers (excluding thumb) | 2020-2023 |  |  |  |  | +9.5% (+0.1 to +20.3) | 11 (4-24) |
|  | Injury to eyes | 2020-2023 |  |  |  |  | +9.5% (+0.8 to +18.9) | 108 (51-196) |
|  | Multiple fractures, dislocations, crashes, wounds, pains, and strains | 2020-2023 |  |  |  |  | +9.5% (+0.5 to +19.2) | 18 (8-33) |
|  | Fracture of femur, other than femoral neck | 2020-2023 |  |  |  |  | +9.5% (+0.6 to +18.8) | 17 (7-36) |
|  | Amputation of lower limbs, bilateral | 2020-2023 |  |  |  |  | +9.5% (+1.1 to +20.6) | 1 (0-2) |
|  | Amputation of upper limb, unilateral | 2020-2023 |  |  |  |  | +9.5% (+0.6 to +20.4) | 1 (0-1) |
|  | Muscle and tendon injuries, including sprains and strains lesser dislocations | 2020-2023 |  |  |  |  | +9.5% (+0.9 to +19.2) | 98 (51-174) |
|  | Amputation of thumb | 2020-2023 |  |  |  |  | +9.4% (+0.9 to +18.3) | 12 (4-30) |
|  | Effect of different environmental factors | 2020-2023 |  |  |  |  | +9.4% (+0.3 to +19.0) | 16 (6-39) |
|  | Dislocation of hip | 2020-2023 |  |  |  |  | +9.4% (+0.2 to +19.7) | 10 (3-24) |
|  | Superficial injury of any part of the body | 2020-2023 |  |  |  |  | +9.4% (+0.2 to +18.7) | 387 (182-646) |
|  | Fracture of clavicle, scapula, or humerus | 2020-2023 |  |  |  |  | +9.4% (+0.5 to +18.7) | 56 (24-106) |
|  | Lower airway burns | 2020-2023 |  |  |  |  | +9.4% (+1.1 to +20.5) | 1 (0-2) |
|  | Fracture of pelvis | 2020-2023 |  |  |  |  | +9.3% (+0.6 to +18.7) | 14 (4-39) |
|  | Dislocation of shoulder | 2020-2023 |  |  |  |  | +9.3% (+0.4 to +19.1) | 20 (7-49) |
|  | Fracture of radius and/or ulna | 2020-2023 |  |  |  |  | +9.3% (+0.4 to +19.5) | 41 (18-77) |
|  | Complications following therapeutic procedures | 2020-2023 |  |  |  |  | +9.3% (+0.1 to +18.3) | 5 (2-9) |
|  | Drowning and nonfatal submersion | 2020-2023 |  |  |  |  | +9.3% (+0.6 to +18.6) | 15 (4-39) |
|  | Poisoning requiring urgent care | 2020-2023 |  |  |  |  | +9.2% (+0.3 to +19.1) | 31 (15-56) |
|  | Burns, >=20% total burned surface area or >= 10% burned surface area if head/neck or hands/wrist involved w/o lower airway burns | 2020-2023 |  |  |  |  | +9.2% (+0.3 to +18.2) | 1 (1-3) |
|  | Fracture of hip | 2020-2023 |  |  |  |  | +9.1% (+0.8 to +18.8) | 3 (1-6) |
